# Exploiting the Cryptic αD Pocket of Casein Kinase 2α (CK2α) to Deliver Highly Potent and Selective Type 1 Inhibitors

**DOI:** 10.1021/acs.jmedchem.5c01807

**Published:** 2025-10-14

**Authors:** Paul A. Glossop, Paul Brear, Susanne Wright, Neil Flanagan, Melanie S. Glossop, Charlotte A. L. Lane, Richard P. Butt, David R. Spring, Marko Hyvönen, Darren Cawkill

**Affiliations:** † Sandexis Medicinal Chemistry Ltd, Discovery Park, Ramsgate Road, Sandwich, Kent CT13 9FF, U.K.; ‡ Department of Biochemistry, 2152 University of Cambridge, 80 Tennis Court Road, Cambridge CB2 1GA, U.K.; § Yusuf Hamied Department of Chemistry, 2152 University of Cambridge, Lensfield Road, Cambridge CB2 1EW, U.K.; ∥ Apollo Therapeutics Ltd, 50-60 Station Road, Cambridge CB1 2JH, U.K.

## Abstract

Casein kinase 2α (CK2α) is an oncology drug target that acts as a positive regulator of many tumorigenic signaling pathways. We previously reported that CK2α has a unique cryptic binding site, the αD pocket, that offers the potential for inhibitors with improved kinase selectivity. The prototype bivalent molecule CAM4066 (**6**) confirmed that improved selectivity could be achieved while binding in both the ATP-binding site and the αD pocket. A drug discovery project to develop a new series of bivalent CK2α inhibitors with increased cell potency and selectivity identified **61f** (APL-5125), a highly potent, ATP-competitive CK2α inhibitor with exquisite kinase selectivity and cellular potency. Compound **61f** demonstrates *in vivo* inhibition of p-AKT S129 in tumors (HCT116) following once-daily oral administration and shows a clear PK–PD relationship with unbound drug exposure. **61f** has a superior preclinical profile to existing CK2α inhibitors and is currently under evaluation in patients with advanced solid tumors.

## Graphical abstract

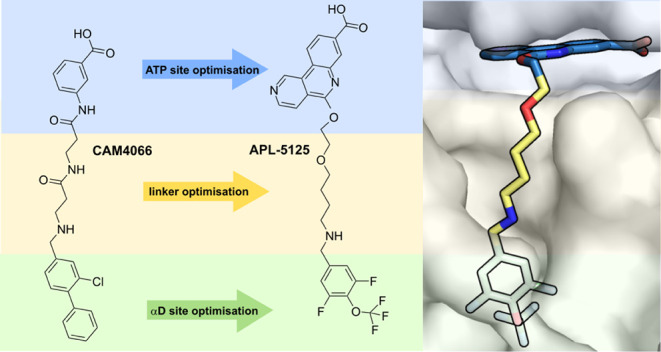

## Introduction

Casein kinase 2 (CK2) is a serine/threonine kinase composed of two catalytic (α and/or α′) subunits and a dimer of regulatory (β) subunits.^1^ The monomeric catalytic subunits are also constitutively active and known to process hundreds of protein substrates.^2,3^ As such, CK2 is a key regulator of many cellular processes, in particular, those related to cellular proliferation and antiapoptotic mechanisms.^4^ The CK2α isoform has been identified as a positive regulator of many tumorigenic signaling pathways, including the Wnt pathway.^5–7^ Inhibition of CK2α has been shown to prevent tumor cell line growth that is driven by different mutations in the Wnt pathway.^8^ Consequently, CK2α has emerged as a relevant therapeutic target for the treatment of numerous tumor types, including colorectal cancer (CRC) and cholangiocarcinoma (CCA).^9–11^

Prior to the work described here, several small-molecule CK2α inhibitors had been described in the literature but only one has since entered clinical development. Silmitasertib (CX-4945, **1**) (Chart [Fig chart1]), is undergoing clinical trials in patients with CCA^12^ and met its primary end point for improvement of progression-free survival during an interim analysis of a Phase 2 study.^13^ Silmitasertib is a tricyclic compound with potent biochemical activity (CK2α IC_50_ = 1 nM in radiometric kinase assay) but weak antiproliferative cell activity (IC_50_ > 1 μM across multiple cancer cell lines) and low levels of kinase selectivity, particularly against CLK2, DAPK3 and HIPK3.^14^ This broad off-target kinase inhibition is linked to silmitasertib being a Type 1 kinase inhibitor; i.e., binding only in the highly conserved ATP-binding site.

**1 chart1:**
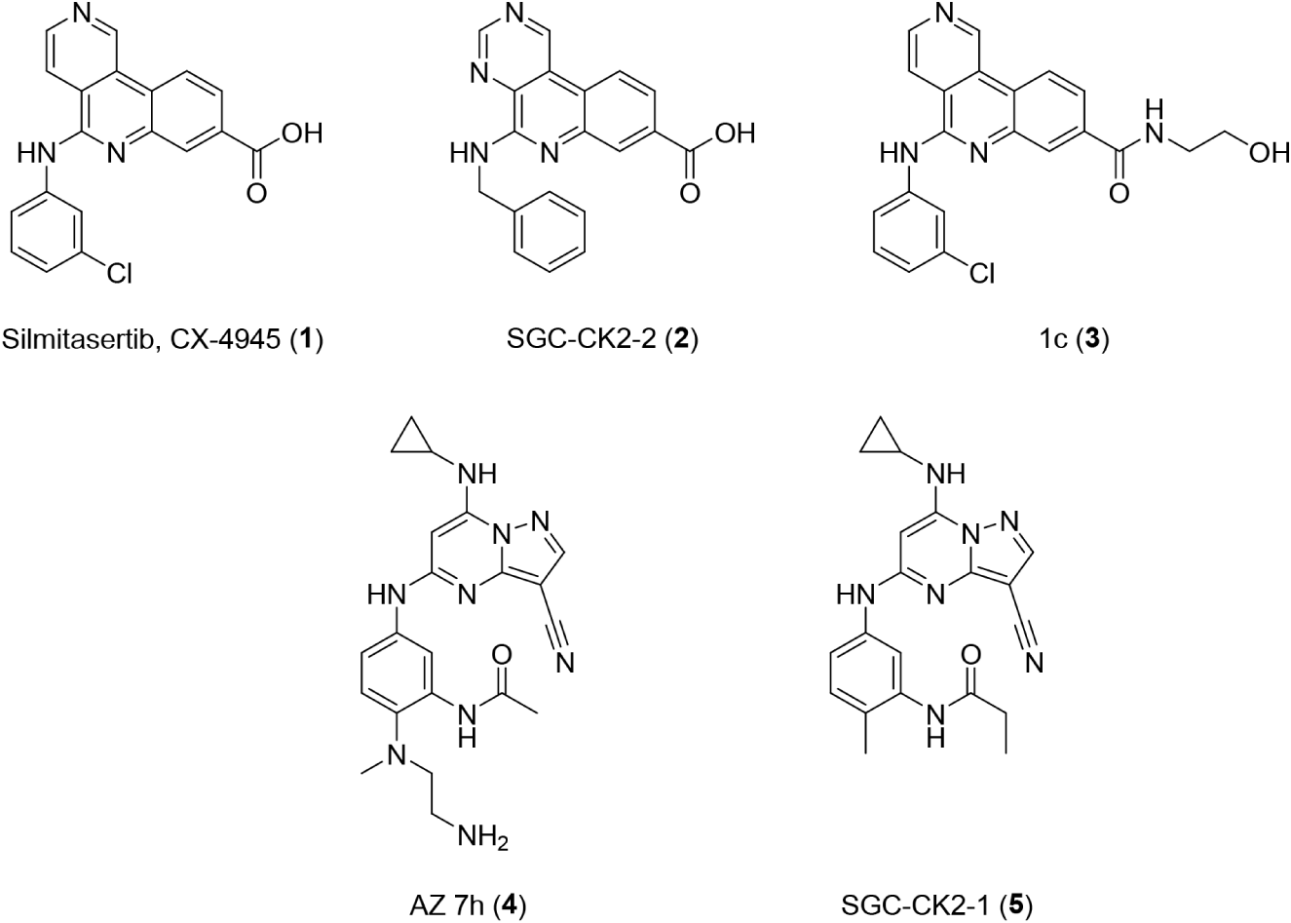
Chemical Structures of Selected Type 1 CK2α Inhibitors

Other published Type 1 CK2α inhibitors are mostly based on bicyclic scaffolds that bind to the hinge region of the ATP-binding site. Researchers from AstraZeneca have reported a series of pyrazolo­[1,5-*a*]­pyrimidines that are highly potent inhibitors of CK2α.^8,15,16^ Compound AZ 7h (**4**) (Chart [Fig chart1]) is highly potent in a biochemical assay (CK2α IC_50_ < 3 nM in mobility shift assay) and in cellular assays (GI_50_ = 5 nM in SW620 cancer cell line) and shows *in vivo* activity in tumor-bearing mice (SW620 xenograft).^8^ However, **4** also inhibits the off-target kinases DAPK3, HIPK3 and DYRK2.^8^ Separately, related series of pyrazolo­[1,5-*a*]­[1,3,5]­triazines,^17,18^ imidazo­[2,1-*f*]­[1,2,4]­triazines^19^ and imidazo­[1,2-*b*]­[1,2,4]­pyridazines^20^ have been published as CK2α inhibitors, but have no evidence for an improved selectivity profile.

During this research, descriptions of additional Type 1 CK2α inhibitors were published. Chemical probes with improved selectivity have been identified with modifications to both the tricyclic series (e.g., SGC-CK2-2, **2**)^21,22^ and pyrazolo­[1,5-*a*]­pyrimidines (e.g., SGC-CK2-1, **5**)^23^ (Chart [Fig chart1]). However, both **2** and **5** are less potent inhibitors of CK2α in a cellular NanoBRET assay compared to the original inhibitors from each of these templates (IC_50_ 240 nM (**1**) vs 920 nM (**2**) and 5 nM (**4**) vs 36 nM (**5**)).^21,23^ Another recent analogue (1c, **3**) (Chart [Fig chart1]) of silmitasertib has also been reported with improved overall kinase selectivity, but weak cellular potency (IC_50_ > 3 μM) against a panel of six human cancer cell lines.^24^ Possible allosteric inhibitors of CK2α have also been disclosed based on a 2-aminothiazole scaffold^25,26^ but subsequent protein–ligand X-ray crystallographic studies by us and other groups suggest they are classic orthosteric inhibitors that bind only in the ATP-binding site.^27,28^

Overall, the *in vitro* pharmacology profile of silmitasertib and other published CK2α inhibitors show there is scope for a significantly improved CK2α inhibitor. In particular, there is a need for an orally bioavailable inhibitor that combines both cellular potency and kinome selectivity. We have previously reported that CK2α has a unique cryptic αD pocket that lies beyond the ATP-binding site, providing an opportunity to design CK2α inhibitors with increased selectivity over the human kinome.^29,30^ This resulted in the identification of CAM4066 (**6**) (Chart [Fig chart2]), which binds in a bivalent manner to both the ATP-binding site (orthosteric) and the αD pocket (cryptosteric). While potency is modest compared to reported inhibitors such as **1** and **4**, interaction with the αD pocket led to increased selectivity.^29,30^ Since the publication of this work, other research groups have developed bivalent CK2α inhibitors that simultaneously occupy those binding sites.^31–33^ Compounds such as KN2^31^ (**7**) and AB668^32^ display biochemical CK2α inhibition (*K*_i_ = 6 and 41 nM, respectively) and cell potency (HEK293 IC_50_ = 16 and 0.6 μM, respectively), with improved kinase selectivity, consistent with our findings. During the writing of this manuscript, two related articles have been published that describe further inhibitors occupying both the ATP and αD binding sites.^34,35^ The first describes KDX1381 (**8**) as a bivalent CK2 inhibitor developed from AB668, and reports that KDX1381 (**8**) retains the selectivity profile of AB668 while further improving cell potency (18-fold) in the renal cell carcinoma cell line (786-O) used for screening.^34^ However, the physicochemical properties of KDX1381 (**8**) lead to high clearance and low oral bioavailability. The second describes Biv5 as a bivalent CK2 inhibitor developed from CX-4945 (**1**), and reports that Biv5 further improves biochemical potency (13-fold) and selectivity against a limited kinase panel of 16 targets.^35^ The cellular potency and *in vivo* properties of Biv5 are not reported.

**2 chart2:**
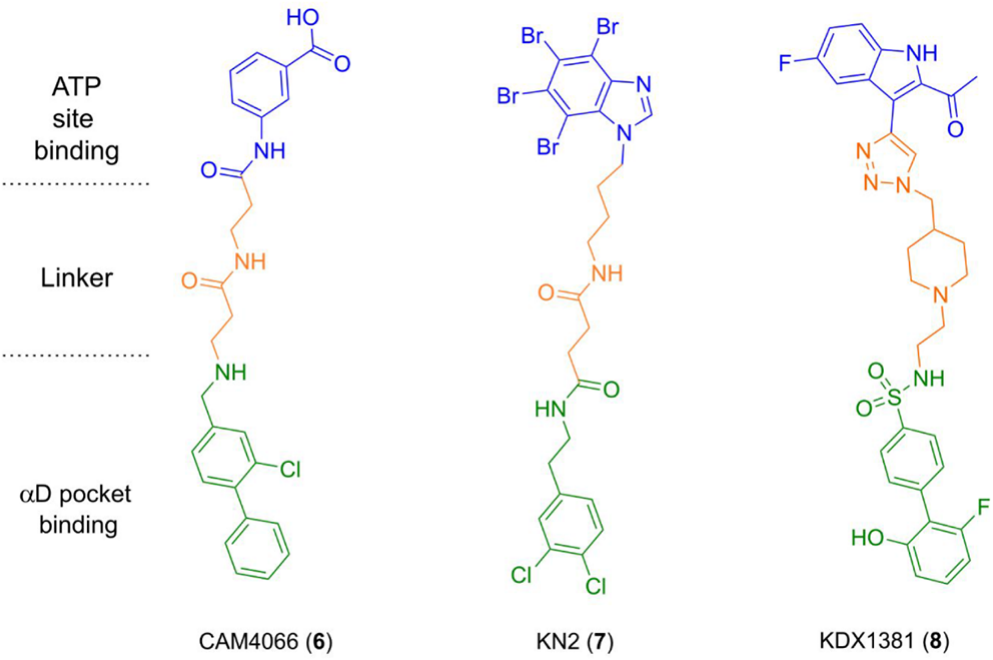
Chemical Structures of Selected Bivalent CK2α Inhibitors

Continuing our bivalent design hypothesis, we now report an extensive optimization campaign that led to the discovery of APL-5125 (**61f**), a highly potent and selective ATP-competitive CK2α inhibitor currently under evaluation as an oral treatment in patients with solid tumors, including CRC.^36^

### Chemistry

Literature compounds **1**, **4** and **6** were prepared according to published procedures.^8,14,29^

Pyrazole **12** was prepared according to Scheme 1. Reductive amination of 3-chloro-4-phenylbenzaldehyde^30^ with aminopropanoic acid followed by Boc-protection gave acid **9**. Subsequent amide coupling with 3,3-diethoxypropan-1-amine followed by deprotection yielded aldehyde **10**. Separately, methyl 3-amino-4-bromobenzoate underwent Pd-mediated Suzuki coupling to afford aniline **11**. Reductive amination between **10** and **11**, followed by deprotection, gave hit compound **12**.

**Scheme 1:**
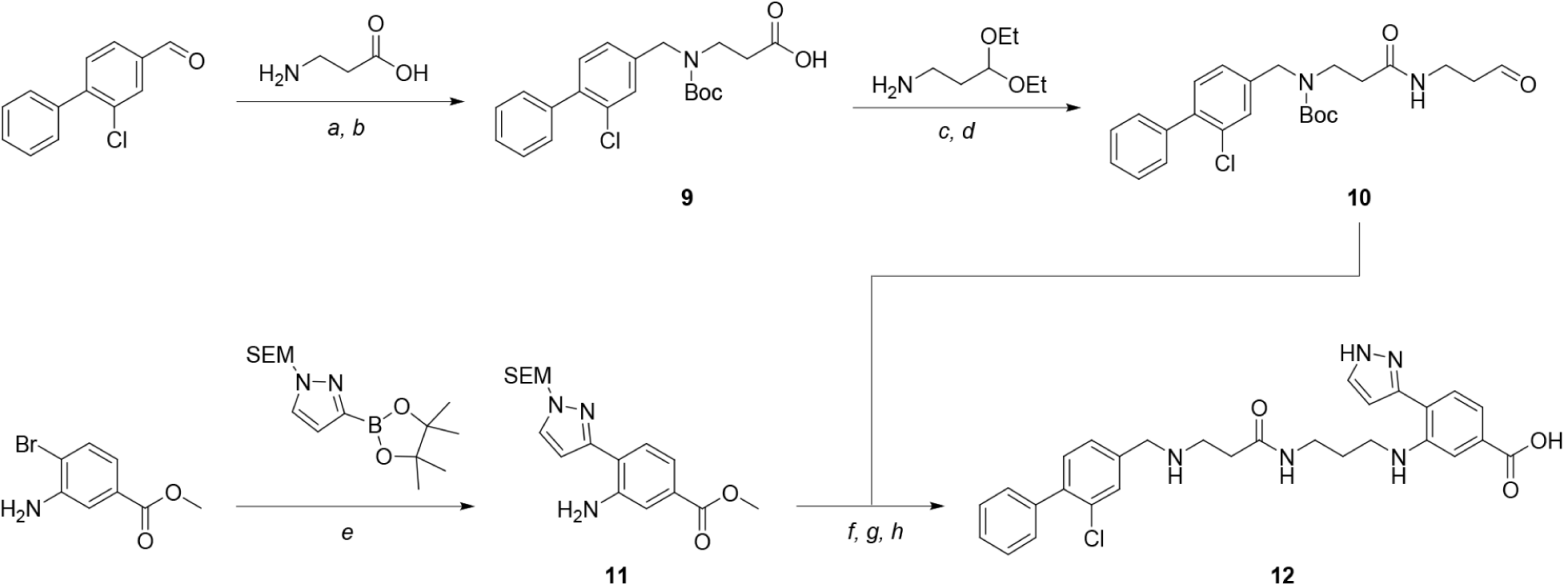
Synthesis of Hit Compound **12** ^a^

a Reagents: (a) (1) MeOH, rt, 1 h; (2) NaBH­(OAc)_3_, rt, 14 h. (b) Boc_2_O, NaHCO_3_, THF/H_2_O, rt, 2 h. (c) T_3_P (50% in EtOAc), TEA, THF, 0 °C, 2 h. (d) AcOH, H_2_O, rt, 2 h. (e) Pd­(dppf)­Cl_2_, K_3_PO_4_, toluene/EtOH/H_2_O, 90 °C, 4 h. (f) MeOH, AcOH, NaBH_3_CN, rt, 48 h. (g) HCl in MeOH (4 M), rt, 1 h. (h) NaOH (aq., 2 M), MeOH, rt, 4 h.

Indazole **14** was prepared according to Scheme 2 ^37^ Methyl 4-bromo-1H-indazole-6-carboxylate was THP-protected then converted to the corresponding 4-amino-indazole derivative **13**. Subsequent reductive amination with aldehyde **10**, followed by deprotection, gave hit compound **14**.

**Scheme 2:**
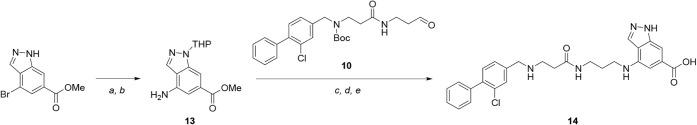
Synthesis of Hit Compound **14** ^a^

a Reagents: (a) 3,4-dihydro-2H-pyran, PTSA, THF, 50 °C, 12 h. (b) NH_4_OH, CuI, K_2_CO_3_, (2*S*,4*S*)-4-hydroxypyrrolidine-2-carboxylic acid, DMSO, 90 °C, 16 h. (c) NaBH­(OAc)_3_, DCE, rt, 16 h. (d) HCl in MeOH (4 M), rt, 1 h. (e) NaOH (aq., 2 M), MeOH, rt, 4 h.

Compounds from our lead tricyclic series were synthesized according to the routes illustrated in Schemes 3–10, described below.^38,39^

**Scheme 3:**
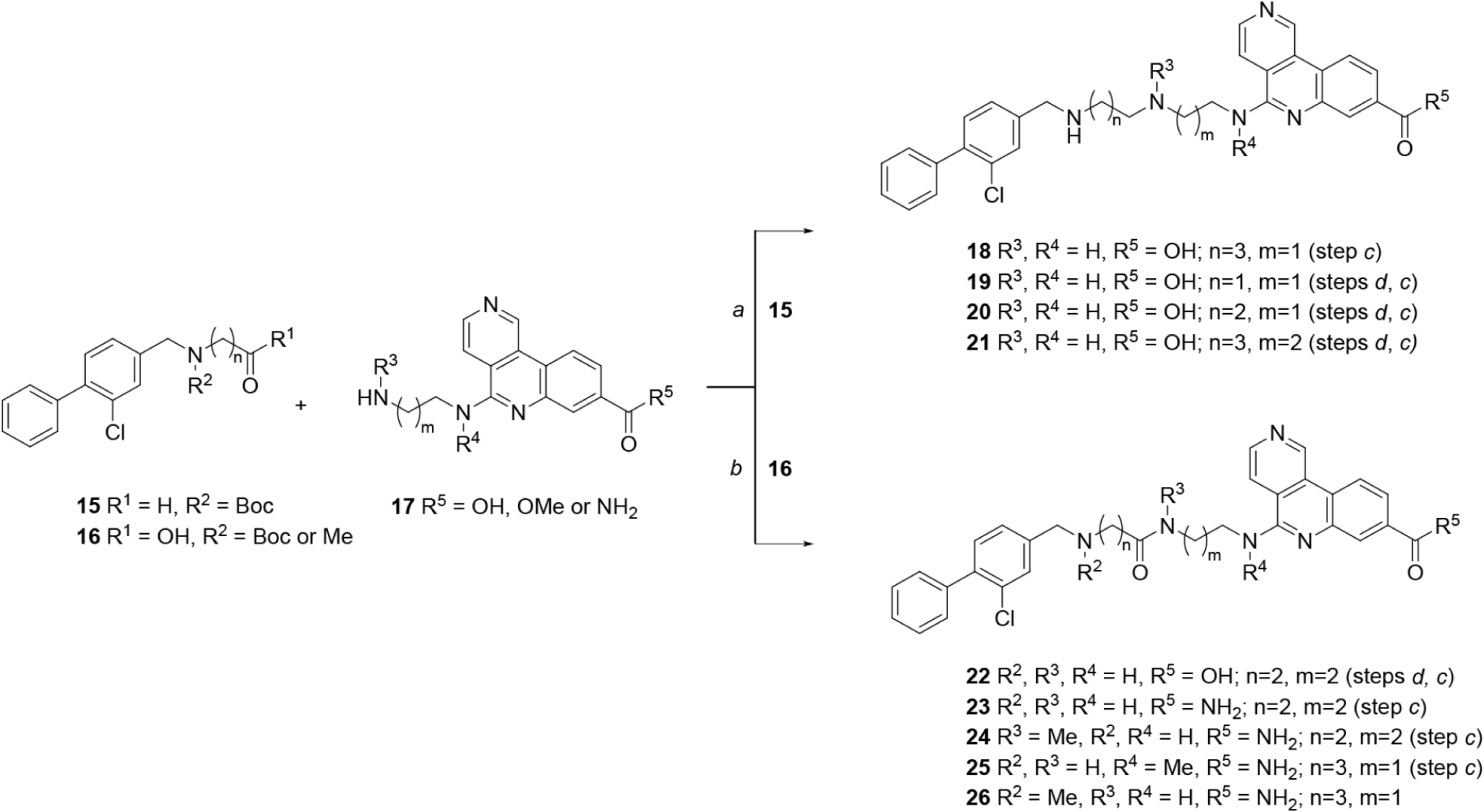
General Synthetic Routes to Compounds **18**–**26**, Incorporating Initial Changes to the Linker^a^

a Reagents: (a) NaBH_3_CN, MeOH, 70 °C, 2 h; or (1) NaOAc, MeOH/DCE, rt, 1 h; (2) NaBH_3_CN, rt, 15 h. (b) T_3_P (50% in EtOAc), TEA, DCM, 0 °C → rt, 15 h; or HATU, DIPEA, DMF/THF, rt, 2–12 h. (c) HCl in EtOAc (4 M), rt, 2 h; or TFA, DCM, rt, 0.5–16 h. (d) LiOH, THF/H_2_O, rt, 1–8 h; or LiOH, MeOH, 70 °C, 1 h.

Compounds with initial modifications to the substitution and length of the linker group were synthesized by the two general strategies shown in Scheme 3. A selection of reactants represented by structures **15**, **16** and **17** were synthesized using similar methods to those for **27** and **28** (Scheme 4). A reductive amination strategy using Boc-protected aldehydes **15** and tricyclic amines **17** (containing a tricyclic acid or ester) gave bivalent molecules with a central amine linker. Subsequent hydrolysis (as needed) and Boc-deprotection gave the initial hit tricyclic acid **18** and analogues **19**–**21**. An amide coupling strategy between *N*-substituted acids **16** and tricyclic amines **17** (containing a tricyclic ester or amide) afforded compounds with a central amide linker. As required, subsequent hydrolysis and/or Boc-deprotection gave tricyclic acid **22** and primary carboxamides **23**–**26**.

**Scheme 4:**
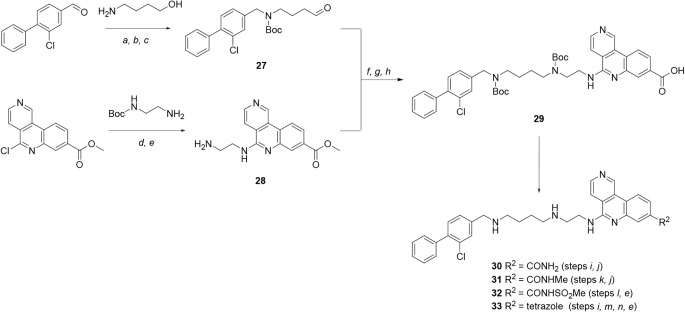
Synthesis of Compounds **30**–**33**, Containing Carboxylic Acid Isosteres^a^

a Reagents: (a) (1) MeOH, 4Å MS, rt, 18 h; (2) NaBH_3_CN, rt, 4 h. (b) (Boc)_2_O, THF/H_2_O, NaHCO_3_, rt, 18 h. (c) DMP, DCM, rt, 15 h. (d) DIPEA, DMSO, 70 °C, 17 h. (e) HCl in 1,4-dioxane (4 M), rt, 2–5 h. (f) (1) NaOAc, MeOH, rt, 1 h; (2) NaBH_3_CN, rt, 17 h. (g) (Boc)_2_O, DMAP, TEA, THF, reflux, 18 h. (h) LiOH, THF/H_2_O, rt, 8 h. (i) NH_4_Cl, HOBt, EDCI, DIPEA, DMF, 70 °C, 8 h. (j) TFA, DCM, rt, 2–8 h. (k) (1) HATU, DMF, TEA, rt, 30 min; (2) MeNH_3_Cl, rt, 30 min. (l) methane sulfonamide, DCC, DMAP, DCM, rt, 20 h. (m) TFAA, TEA, DCM, rt, 12 h. (n) NaN_3_, NH_4_Cl, DMF, 100 °C, 23 h.

The synthetic route to tricyclic compounds containing a tricyclic acid or acid isostere is shown in Scheme 4. Reductive amination of 3-chloro-4-phenylbenzaldehyde^30^ with 4-aminobutan-1-ol, followed by Boc-protection and oxidation, gave aldehyde **27**. Nucleophilic displacement of methyl 5-chlorobenzo­[*c*]­2,6-naphthyridine-8-carboxylate^14^ with *tert*-butyl (2-aminoethyl)­carbamate gave amine **28**, following Boc-deprotection. Subsequent reductive amination between **27** and **28**, followed by Boc-protection and ester hydrolysis gave tricyclic acid **29** as a key intermediate for the synthesis of acid isosteres. For example, amide coupling reactions followed by Boc-deprotection gave amides **30**–**31** and acyl sulfonamide **32**. The Boc-protected primary carboxamide intermediate was also used to synthesize the C-linked tetrazole **33** via dehydration to the nitrile, cyclization with sodium azide and Boc-deprotection.

Syntheses of tricyclic compounds containing either a central urea or sulfonamide are shown in Scheme 5. Fmoc-protected diamines **34** were synthesized from 3-chloro-4-phenylbenzaldehyde^30^ by reductive amination, Fmoc-protection and Boc-deprotection. Separately, tricyclic amine **35** was obtained via simple derivatization of the precursor to **28** (Scheme 4). Amine **34** (*n* = 1) was treated with CDI followed by tricyclic amine **35** to afford urea **36**. Amine **34** (*n* = 2) was converted to amino sulfonamide **37**, followed by S_N_Ar reaction with methyl 5-chlorobenzo­[*c*]­2,6-naphthyridine-8 ^14^ and subsequent treatment with ammonia (ester → amide conversion) to give sulfonamide **38**.

**Scheme 5:**
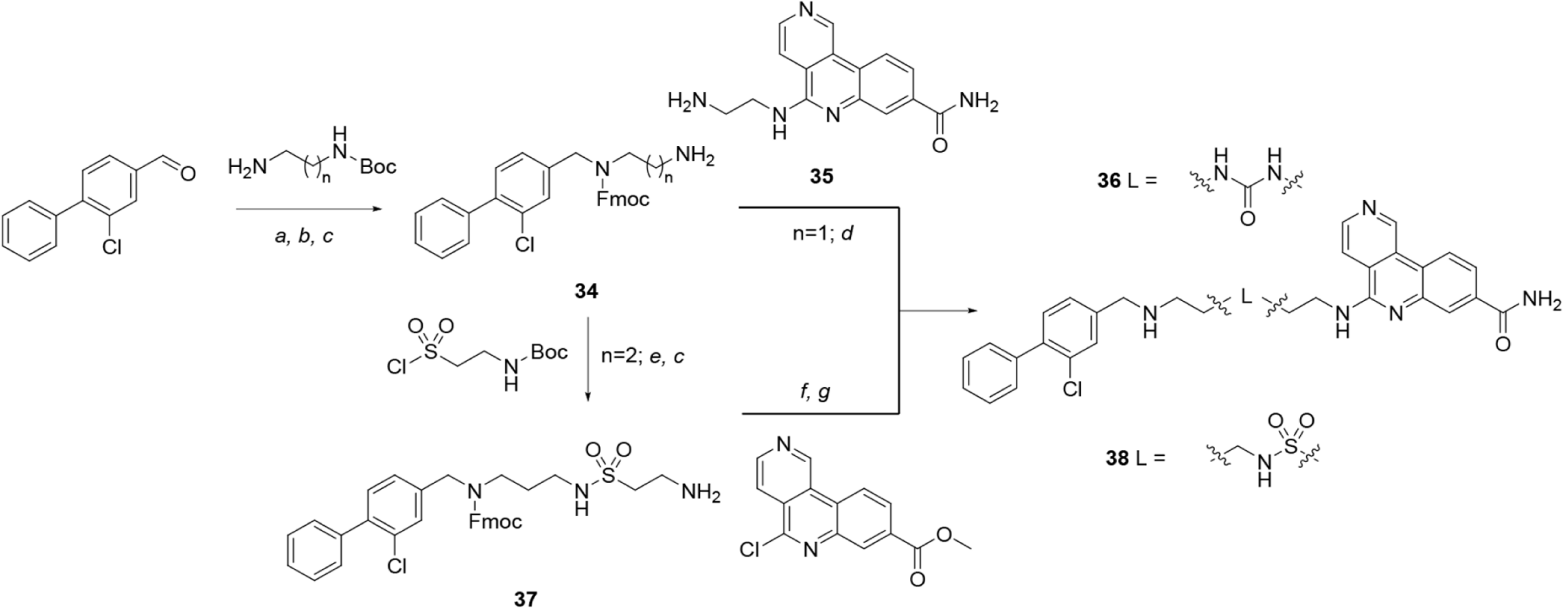
Synthesis of Compounds **36** and **38**, Incorporating Initial Changes to the Linker^a^

a Reagents: (a) (1) MeOH, rt, 2 h; (2) NaBH­(OAc)_3_, rt, 12 h. (b) Fmoc-Cl, DIPEA, DCM, rt, 1 h. (c) HCl in 1,4-dioxane (4 M), rt, 1 h. (d) (1) **34**, CDI, TEA, DMF, rt, 1 h; (2) **35**, DMF, rt, 12 h. (e) DIPEA, DMF, 15 °C, 30 min. (f) DIPEA, DMSO, 80 °C, 12 h. (g) NH_3_ in MeOH (7 M), 80 °C, sealed tube, 14 h.

The synthesis of molecules containing a central ether linker is shown in Scheme 6. These compounds were conveniently synthesized from suitably protected diamino ethers by adding the tricyclic moiety first, followed by the biaryl group, or vice versa, accompanied by suitable protection strategies (e.g., Boc, Fmoc) and functional group interconversions (e.g., ester → amide). Thus, diamino ethers **39**–**40** underwent S_N_Ar reaction with methyl 5-chlorobenzo­[*c*]­2,6-naphthyridine-8 ^14^ to give **41**–**42**, followed by reduction amination with 3-chloro-4-phenylbenzaldehyde^30^ to give **44**–**45**. Alternatively, diamino ether **39** was first subjected to the reductive amination step to give **43**, followed by the S_N_Ar reaction to yield **46**.

**Scheme 6:**
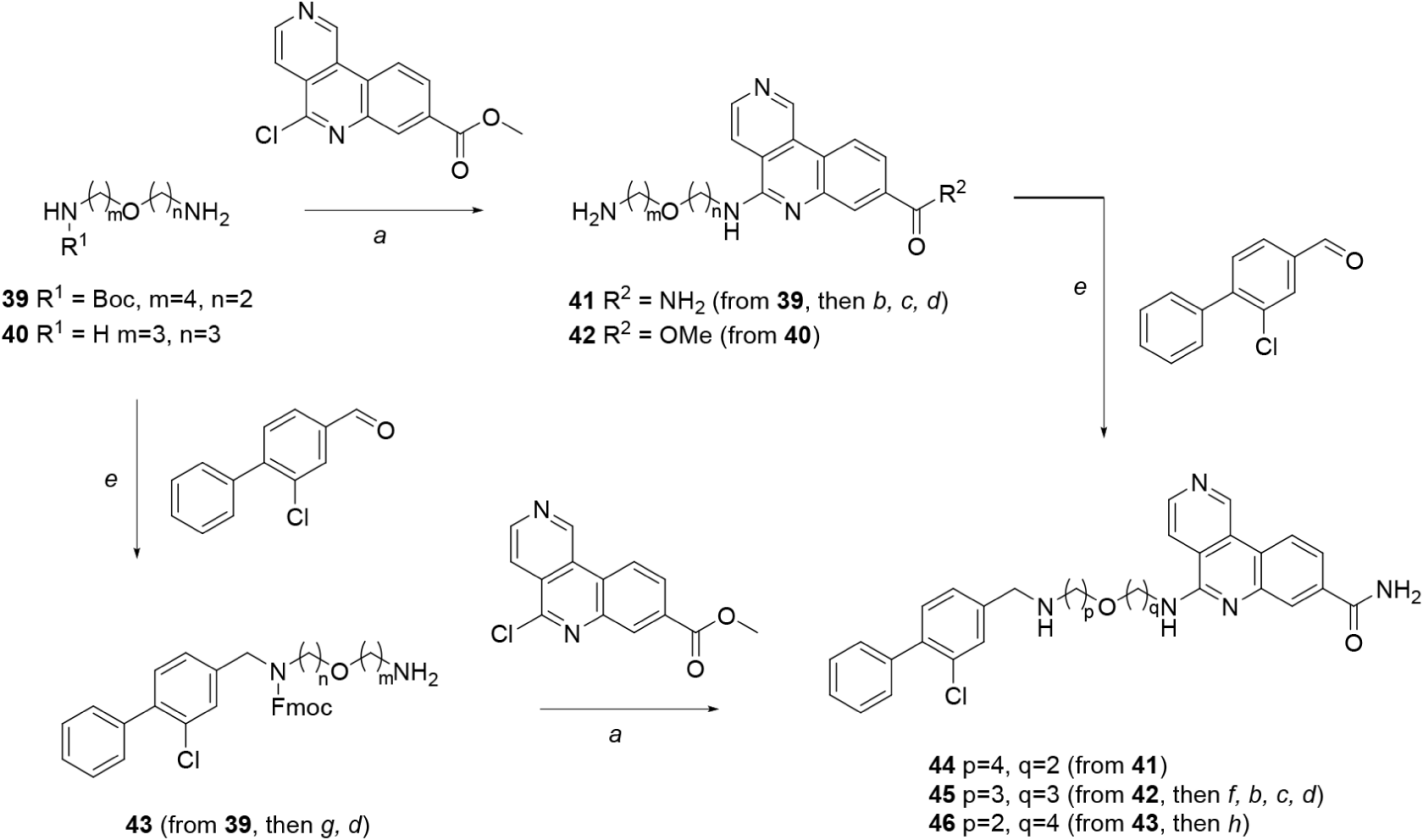
Synthesis of Compounds **44**–**46**, Containing an Ether Linker^a^

a Reagents: (a) DIPEA, DMSO, 65–75 °C, 12–16 h. (b) LiOH or NaOH, THF/MeOH/H_2_O, rt, 4–20 h. (c) NH_4_Cl, HATU, DIPEA, THF, rt, 14 h; or NH_4_Cl, EDCI, HOBt, DIPEA, DMF, rt 3 h. (d) TFA, DCM, rt, 1 h; or HCl in 1,4-dioxane (4 M), rt, 1 h; or HCl in MeOH (4 M), rt, 2 h. (e) (1) MeOH, DIPEA, rt, 3–12 h; (2) NaBH_3_CN, rt, 3–12 h. (f) Boc_2_O, TEA, DCM, rt, 1 h. (g) Fmoc-Cl, NaHCO_3_, 1,4-dioxane/H_2_O, rt, 1 h. (h) NH_3_ in MeOH (7 M), 80 °C, sealed tube, 14–16 h.

Synthetic routes to compounds with replacements for the benzylic amine of the biaryl unit are shown in Scheme 7. Requisite acids **47** were synthesized using standard methods from their corresponding reactants (benzoic acid, benzylamine or benzyl alcohol). Amide coupling reactions between amine **35** and acids **47** yielded benzylic amides **48**–**49** and benzylic ether **50**.

**Scheme 7:**
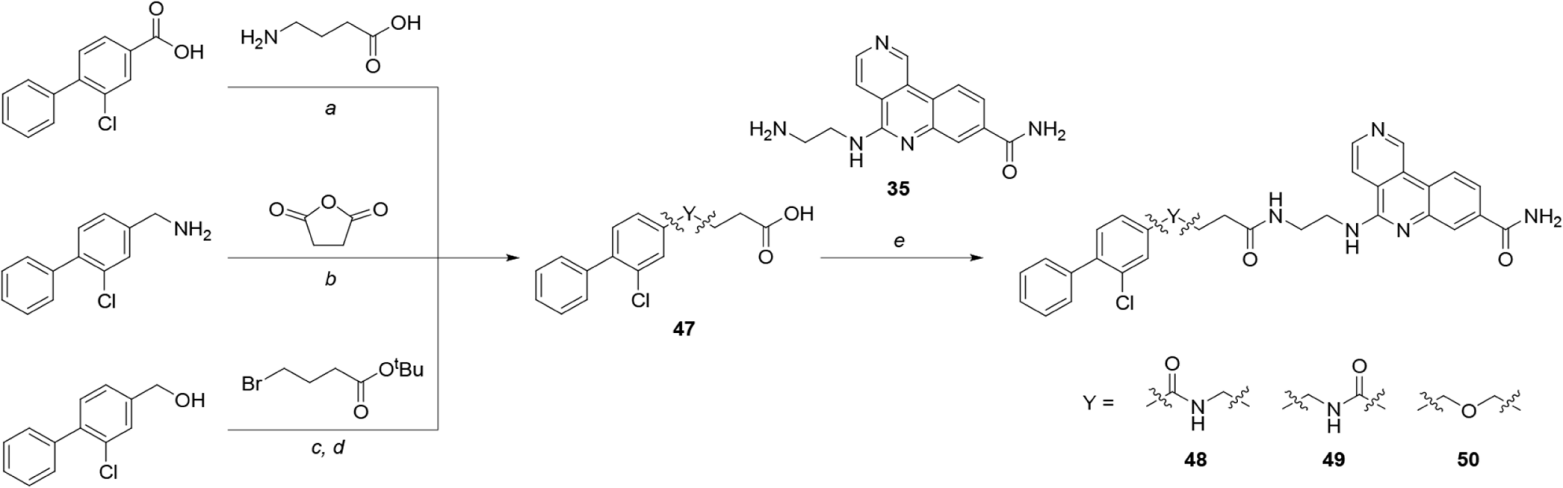
Synthesis of Compounds **48**–**50**, Incorporating Alternatives to the Benzylic Amine^a^

a Reagents: (a) (1) HATU, TEA, DCM, 0 °C, 5 h; (2) 4-aminobutanoic acid, rt, 30 min. (b) TEA, THF, rt, 1 h. (c) TBAI, Ag_2_O, MeCN, 50 °C, 12 h. (d) TFA, DCM, rt, 1 h. (e) T_3_P (50% in EtOAc), TEA, DCM, rt, 12 h; or HATU, DIPEA, THF, rt, 2–12 h.

Modification of the biaryl group was achieved efficiently by synthesis and derivatization of late-stage intermediates, as shown in Scheme 8. Tert-butyl (4-hydroxybutyl)­carbamate was alkylated with 2-bromoacetonitrile and hydrogenated to give Boc-protected diamino ether **39**. Subsequent S_N_Ar reaction with methyl 5-chlorobenzo­[*c*]­2,6-naphthyridine-8 ^14^ gave intermediate **51** that was Boc-deprotected either directly, to give tricyclic ester **53**, or following ester derivatization, to give amines **41** (tricyclic amide) and **52** (tricyclic acid). With these amino intermediates in hand, reductive amination with a variety of benzaldehydes (followed by deprotection, as needed) gave tricyclic amides **54a**–**m** and tricyclic acids **55a**–**b**.

**Scheme 8:**
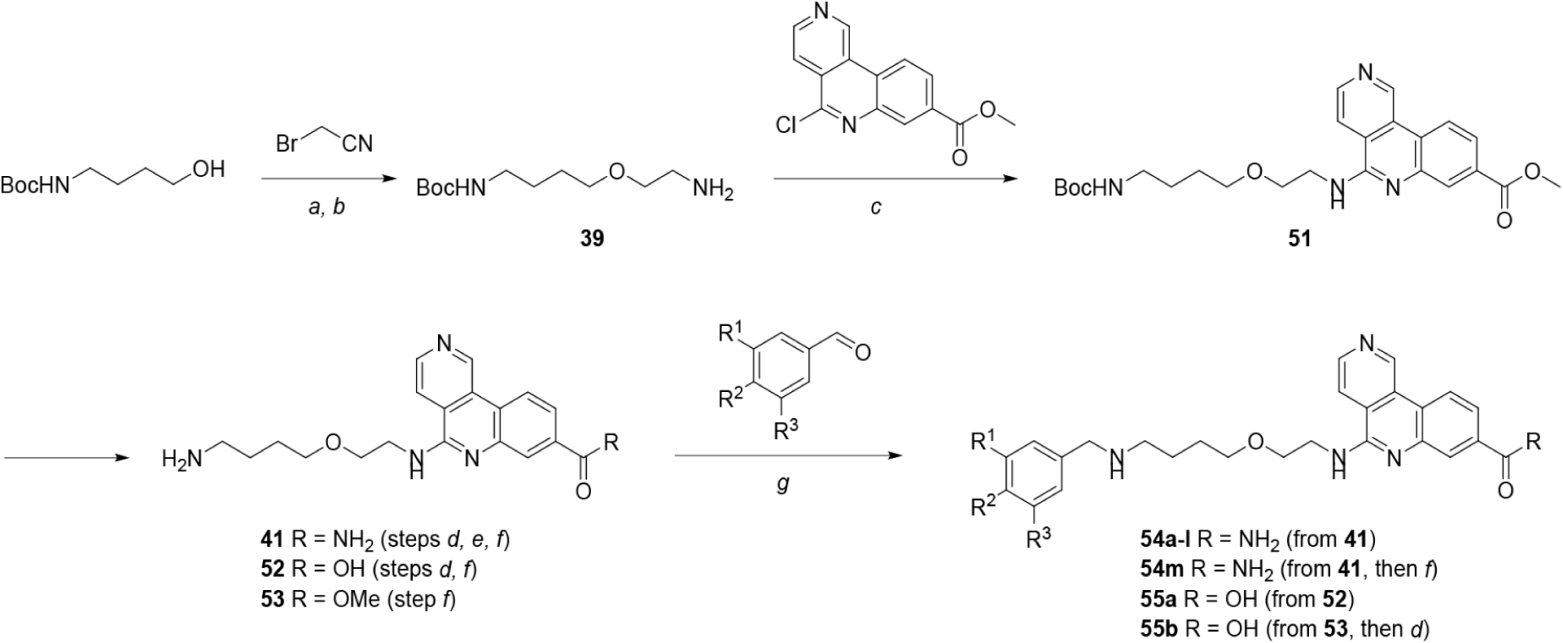
Synthesis of Compounds **54a**–**m** and **55a**–**b**, Incorporating Substitution of the αD Fragment^a^

a Reagents: (a) TBAI, Ag_2_O, DCM, rt, 16 h. (b) NH_4_OH (aq), Raney Ni, MeOH, H_2_, 45 psi, rt, 16 h. (c) DIPEA, DMSO, 75 °C, 12 h. (d) NaOH or LiOH, THF/MeOH/H_2_O, rt, 4 h. (e) NH_4_Cl, EDCI, HOBt, DIPEA, DMF, rt 3 h. (f) HCl in MeOH (4 M), rt, 2 h; or HCl in 1,4-dioxane (4 M), rt, 1–16 h; or TFA, DCM, rt, 30 min. (g) (1) DIPEA or NaOAc, MeOH, rt, 2–16 h; (2) NaBH­(OAc)_3_ or NaBH_3_CN, rt, 0.5–12 h.

Molecules containing substitution/rigidification of the linker adjacent to the tricyclic group were synthesized as outlined in Scheme 9. Appropriately substituted amino-ether linkers **56a**–**g** underwent S_N_Ar reaction with methyl 5-chlorobenzo­[*c*]­2,6-naphthyridine-8 ^14^ using standard thermal conditions. Corresponding hydroxy-ether linkers **56h**–**i** were deprotonated using sodium hydride prior to addition of methyl 5-chlorobenzo­[*c*]­2,6-naphthyridine-8 ^14^ at room temperature. Deprotection of these products afforded amino-ester intermediates **57a**–**i**. Reductive amination with 3-chloro-4-(trifluoromethoxy)­benzaldehyde and ester hydrolysis yielded tricyclic acids **58a**–**i**.

**Scheme 9:**
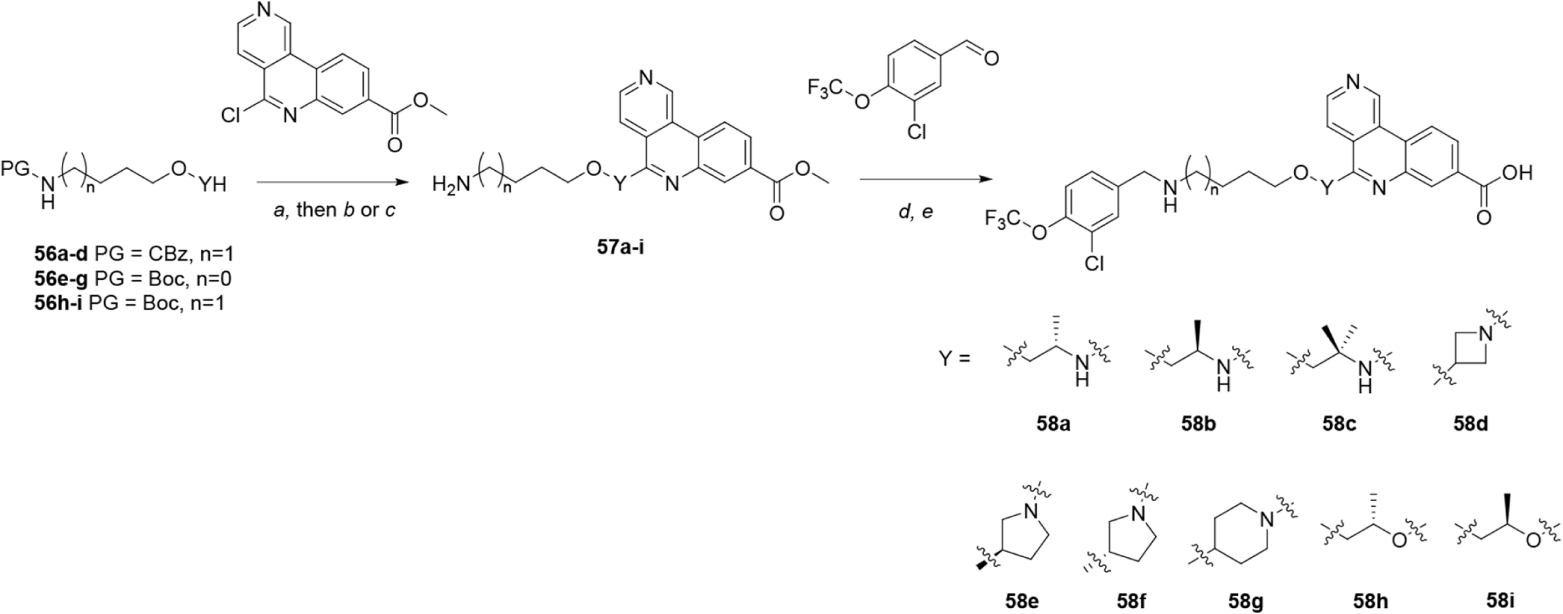
Synthesis of Compounds **58a**–**i**, Containing Changes to the Ether Linker^a^

a Reagents: (a) **56a**–**g**: DIPEA, DMSO, 80–120 °C, 2–20 h; **56h**–**i**: (1) NaH (60% in oil), THF, rt, 30 min; (2) methyl 5-chlorobenzo­[*c*]­2,6-naphthyridine-8-carboxylate, rt, 2 h. (b) NH_4_OH (aq), 10% Pd/C, MeOH, H_2_, 15 psi, rt, 1–16 h. (c) HCl in MeOH (4 M), rt, 1 h; or HCl in 1,4-dioxane (4 M), rt, 0.5–1.5 h. (d) (1) DIPEA or NaOAc, MeOH, rt, 1–16 h; (2) NaBH_3_CN or NaBH­(OAc)_3_, rt, 0.5–3.5 h. (e) LiOH, THF/MeOH/H_2_O, rt, 1–20 h.

Further modification of the pendant aryl group was performed on a late-stage intermediate, as shown in Scheme 10. Tert-butyl *N*-(4-hydroxybutyl)­carbamate was converted to hydroxy-ether **59** via alkylation with allyl bromide and oxidative cleavage/reduction using ozone and sodium borohydride. Using a similar method to Scheme 9, hydroxy-ether **59** was deprotonated with NaH and reacted with methyl 5-chlorobenzo­[*c*]­2,6-naphthyridine-8 ^14^ at elevated temperature; concomitant hydrolysis of the ester occurred during the reaction. Boc-deprotection then provided amino acid **60**. Finally, reductive amination of amine **60** with a range of substituted benzaldehydes and sodium triacetoxyborohydride yielded bivalent tricyclic acids **61a**–**j**. HPLC analysis of **61f** is shown in Figure S1.

**Scheme 10:**
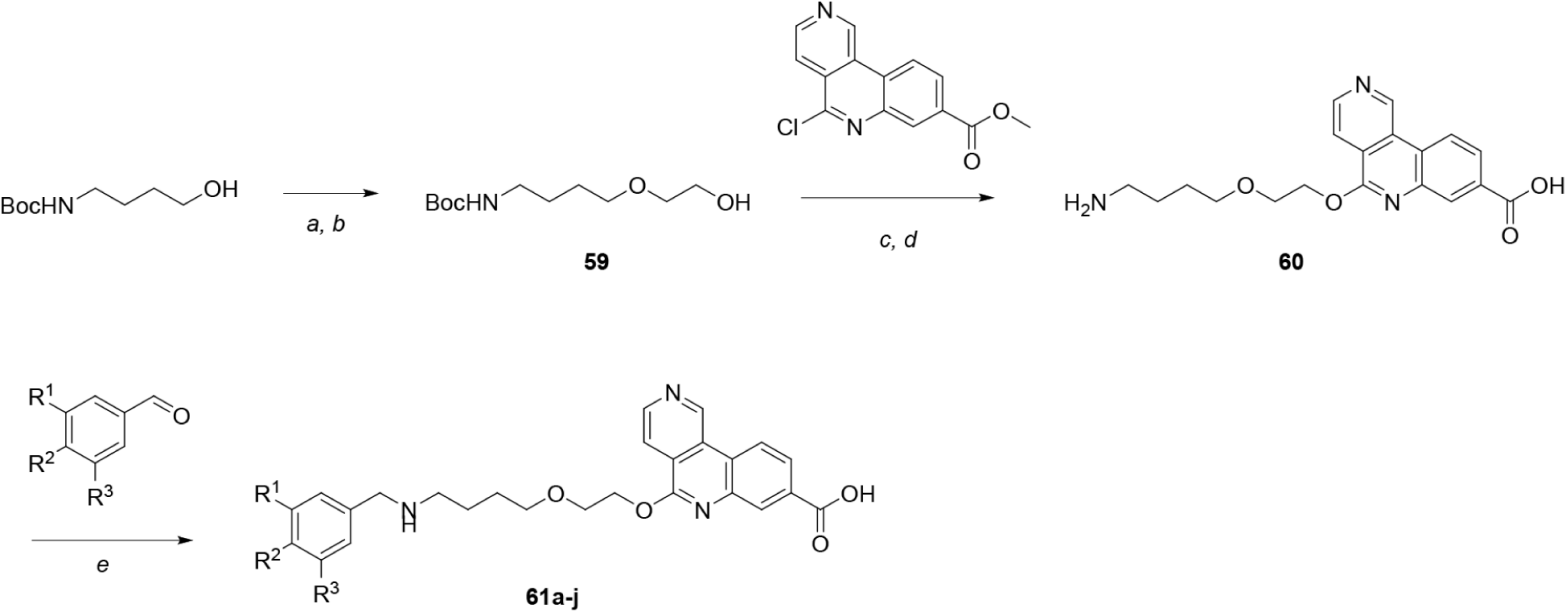
Synthesis of Compounds **61a**–**j**, Incorporating Substitution of the αD Fragment^a^

a Reagents: (a) allyl bromide, NaOH, 1,4-dioxane, 70 °C, 12 h. (b) (1) O_3_, DCM, −78 °C → 0 °C; (2) NaBH_4_, 0 °C → rt, 12 h. (c) NaH (60% in oil), DMF, 0 °C → 80 °C, 12 h. (d) TFA, DCM, rt, 30 min. (e) (1) DIPEA, MeOH, rt, 1 h; (2) NaBH­(OAc)_3_, rt, 11 h.

## Results and Discussion

Although the previous discovery of CAM4066 (**6**) was encouraging,^29,30^ it was clear that significant increases (e.g., 100–1000-fold) in both enzymatic and cellular potency would be required to deliver inhibition that would result in a therapeutic effect. In parallel, this would need to be achieved while retaining the selectivity advantages that engagement with the αD pocket had provided and was key to our overall strategy. **6** contains three structural motifs that were considered as initial starting points for optimization: 1) the benzoic acid that binds in the ATP-binding site; 2) the biaryl fragment originally discovered to bind in the αD pocket; 3) the flexible linker unit required to span these two binding sites (Chart [Fig chart1]). Based on knowledge of the binding mode and interactions of **6** with CK2α,^29,30^ we hypothesized that optimization of binding interactions within the ATP-binding site could offer the highest likelihood of successfully increasing potency while retaining drug-like physicochemical properties.^40,41^ Previous work to identify **6** focused on minimizing interactions in the ATP-binding site due to its highly conserved nature among other kinases.^29,30^ However, we felt that knowledge of the overall binding mode now enabled us to explore whether increasing interactions in the ATP-binding site could drive potency while utilizing the unique αD pocket to retain selectivity. We believed that the other two structural motifs in **6** were unlikely to be the most suitable starting points, given the large increase in potency that was required.

Exposed by the displacement of the flexible αD helix (Asp120 to Thr127), the αD pocket is largely hydrophobic in nature making it unattractive as a starting point to increase potency and control drug-like properties, especially as **6** already occupies this region with a lipophilic biphenyl group. The linker motif spans the two binding sites that are approximately 9–10 Å apart and is long and flexible with limited polar interactions to the protein, also making this an unfavorable approach to increase potency in the first instance. In fact, the preferred strategy for these two motifs was to reduce lipophilicity and rotatable bonds where possible to give improved drug-like physicochemical properties.^40,41^

Following a design strategy to increase potency via the number of protein–ligand interactions made between the benzoic acid of **6** and the ATP-binding site, an opportunity was identified to grow toward and engage with residues Glu114 to Val116 in the hinge region of CK2α. This was supported by the observation that these residues are involved in the binding of **1** (PDB: 3PE1) and **4** (PDB: 5H8E), both of which are highly potent, ATP-competitive inhibitors of CK2α.^8,14^ Furthermore, they demonstrated that crystal structures available in the Protein Data Bank (PDB) provided a significant opportunity to optimize specific binding interactions in the ATP-binding site. Consequently, all kinase crystal structures within the PDB were interrogated against the published crystal structure of **6** bound to CK2α (PDB: 5CU4).^29,30^ The results of these overlays were analyzed to identify ligands (of any kinase) that appeared to have functional groups with the correct vectors and protein interactions to build onto or hybridize with the benzoic acid of **6**. Following this analysis, approximately 200 compounds were initially designed, further triaged to a smaller set of 28 varied compounds to test the hypothesis that potency and selectivity could be combined and were not mutually exclusive. All these initial compounds retained the biaryl αD fragment present in **6** but with a slightly revised amine-based linker (i.e., without the amide carbonyls) for two reasons: 1) to reduce potential clashes with new substituents on the benzoic acid; 2) to maximize linker flexibility and likelihood of new designs binding simultaneously in both pockets.

This compound subset covering several design ideas was tested in ADP-Glo^TM^ kinase assays against CK2α and two initial kinase selectivity targets, CLK2 and DAPK3, with all assays being performed at an ATP concentration equivalent to or <*K*_M_ (Table S1). Both selectivity targets are potently inhibited by silmitasertib (**1**) and were considered to be a relevant early assessment of the selectivity profile of new compounds and our design hypothesis. Compounds that showed evidence of increased potency relative to **6** and improved selectivity relative to **1** were subsequently tested in ADP-Glo^TM^ selectivity assays (ATP concentration at or <*K*_M_) against HIPK3 and DYRK2 (also inhibited by **1**) and in cellular assays (HCT116 cell line) to assess cellular target engagement using NanoBRET technology and inhibition of proliferation. Overall, data from this initial set of compounds identified three new potential series of CK2α inhibitors: Series 1) tricycle, exemplified by **18**; Series 2) pyrazole, exemplified by **12**; Series 3) indazole, exemplified by **14** (Table 1). Series 1 contained a known CK2α hinge-binding motif, but Series 2 and Series 3 contained hinge-binding motifs not previously reported in CK2 inhibitors, which validated the approach of mining all kinase structures to design the initial screening set. All three series showed evidence of increased CK2α potency compared with **6**, and increased kinase selectivity compared with **1** (Table 1). In particular, Series 1 (**18**) showed very potent inhibition of CK2α (IC_50_ = 0.386 nM), encouraging kinase selectivity (59-fold to >129,000-fold), which was an immediate improvement over the selectivity profile of **1**, and early evidence of cellular activity (in contrast to **6**). Crystal structure of **18** bound to CK2α was determined by X-ray crystallography, confirming our hypothesis of a bivalent binding mode in both the αD pocket and ATP-binding site, plus additional interactions with the hinge region of CK2α (Figure 1A and B). The key ATP-binding site interactions include: retention of the salt-bridge interaction between the carboxylic acid of **18** to Lys68 and the through-water interaction to the acid of Glu81; new interactions with the hinge region including the hydrogen-bonding interaction between the pyridine of the tricycle and the N–H of Val116, and aromatic C–H interactions with the backbone C=O groups of Glu114 and Val116. The linker exits the ATP-binding site from the central ring of the tricycle, turns 90° and extends toward the αD pocket. The protonated secondary benzylic amine makes H-bond interactions to backbone C=O groups of Val162 and Pro159; subsequent data show that these interactions are critical to anchor the molecule and project the biaryl deep into the lipophilic αD pocket. Superposition of the X-ray structures of **1** (PDB: 3PE1), **6** (PDB: 5CU4) and **18** further illustrate that the design strategy successfully delivered the intended binding mode and is different to **4** (PDB: 5H8E) (Figure 1B). CK2α crystal structures were also generated with hit compounds **12** and **14** that contain novel CK2α hinge-binding motifs (Figure 1C). As expected, the pyrazole of **12** extends to the hinge region and makes one H-bond interaction with the C=O of Val116, whereas the indazole of **14** makes two H-bond interactions with the N–H of Val116 and C=O of Glu114. It is notable that the interaction of **14** with the hinge region drags the carboxylic acid too far away from Lys68 to make a direct salt-bridge interaction but this is replaced by a similar through-water interaction.

**Table 1:**
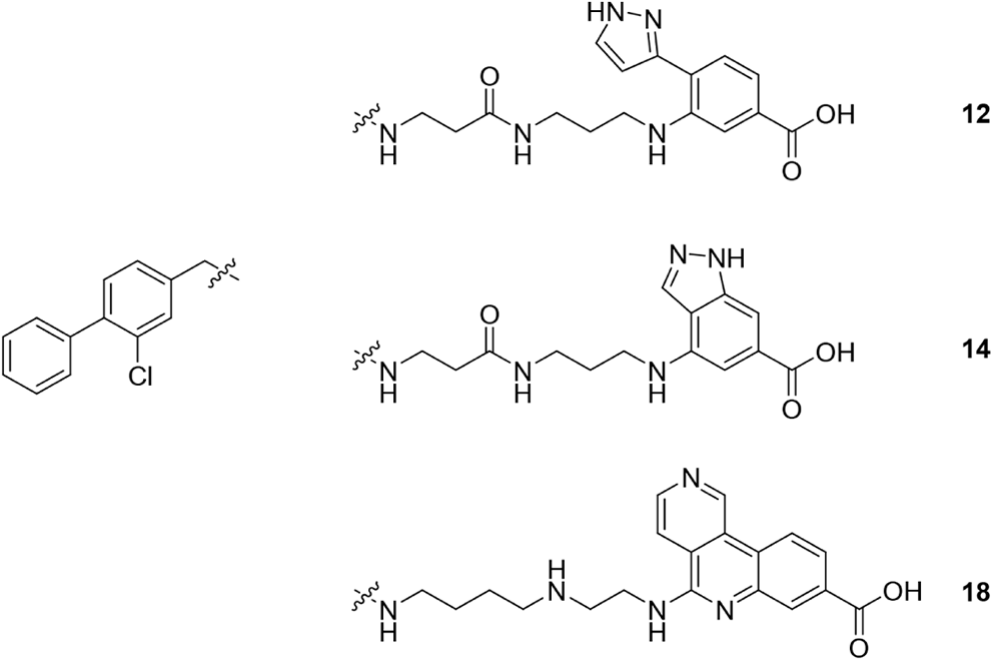
*In Vitro* CK2α Potency, Kinase Selectivity and Cellular Activity of Hit Compounds **12**, **14** and **18** ^a^

	Kinase Inhibition (ADP-Glo^TM^ Assay)					Cellular Assays (HCT116)	
	IC_50_ [nM]	IC_50_ [nM]/Selectivity [Fold]				IC_50_ [nM]	
Compound	CK2α	CLK2	DAPK3	HIPK3	DYRK2	NanoBRET	Proliferation
**1** (CX-4945, Silmitasertib)	0.678 ± 0.196^b^	4.92 ± 1.44/7.3^b^	5.11 ± 1.27/7.5^b^	23.3 ± 7.2/34^b^	36.6 ± 9.1/54^b^	107 ± 45^b^	3,722 ± 730^b^
**4** (AZ 7h)	0.348 ± 0.096^c^	2,678 ± 1,139/7,695^c^	5.23 ± 0.81/15^c^	48.9 ± 7.0/140^c^	39.7 ± 8.3/11^c^	5.37 ± 1.09^c^	3.05 ± 0.27^c^
**6** (CAM4066)	300 ± 80	>50,000/>166^c^	43,550/145^c^	22,350 ± 3,231/75^c^	27,340/91	>50,000^c^	>50,000
**12**	28.8± 5.11^c^	>50,000/2,475	32,950 ± 2,205/1631	>50,000/2,475	8,536/423	ND^d^	>50,000
**14**	132	21,840 ± 9,527/165	1,383 ± 574/11	29,440/223	12,250/93	>50,000	>50,000
**18**	0.386 ± 0.730^c^	216 ± 33/560^c^	22.6 ± 4.0/59	894/2,316	>50,000/129,534		19,200

a All kinase assays were performed at ATP concentration equivalent to or <*K*_M_. All data were generated *n* = 1 initially. For compounds with further repeats, data are shown as the mean ± SD from *n* = 2.

b For compounds with further repeats, data are shown as the mean ± SD from *n* ≥ 10.

c For compounds with further repeats, data are shown as the mean ± SD from *n* = 3–9.

d ND: not determined.

**Figure 1:**
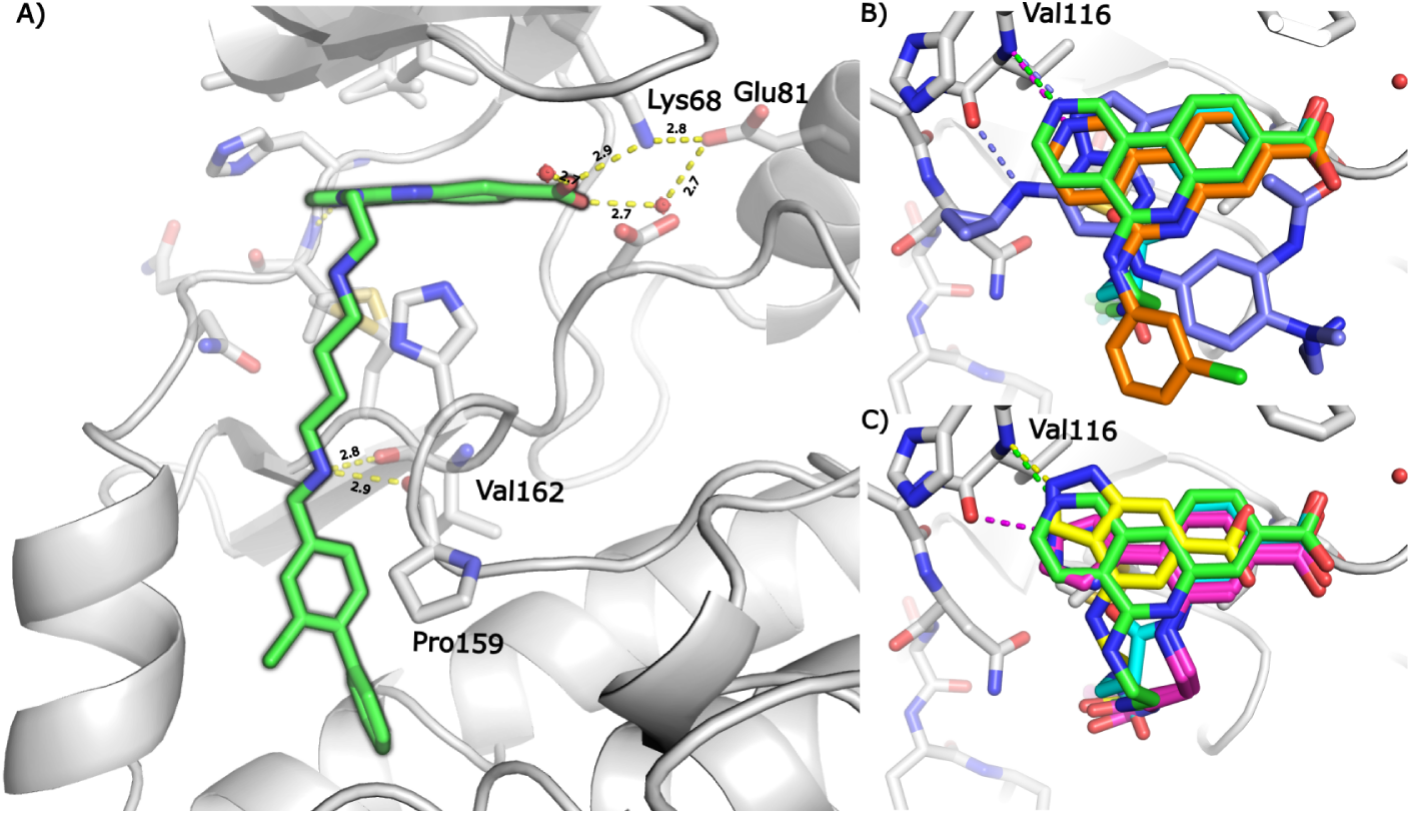
Binding mode of **18** to CK2α compared to the binding modes of established CK2α inhibitors. A) The binding mode of **18** (green, PDB: 7I8O) to CK2α. The hydrogen bonding networks of the carboxylic acid and the benzylic nitrogen are shown as dotted lines. B) The binding mode of **18** (green) in the ATP-binding site is compared to the binding modes of **1** (CX-4945, silmitasertib) (orange, PDB: 3PE1), **4** (AZ 7h) (purple, PDB: 5H8E) and **6** (CAM4066) (blue, PDB: 5CU4) with H-bonds to the hinge region shown as dotted lines in their respective colors. C) The binding mode of **18** (green) in the ATP-binding site is compared to the binding modes of **6** (blue), **12** (pink, PDB: 7I8N) and **14** (yellow, PDB: 7I8M) with H-bonds to the hinge region shown as dotted lines in their respective colors.

The conclusion of this initial phase of the project validated our design strategy: i.e., extending molecules deeper into the ATP-binding site and engaging the hinge region while simultaneously binding in the αD pocket was able to deliver CK2α inhibitors with increased potency and selectivity. Consequently, optimization of Series 1 was initiated as a high priority due to its overall *in vitro* profile relative to Series 2 and Series 3. Optimization of Series 2 and Series 3 also identified compounds with potent CK2α inhibition (IC_50_ < 1 nM), good kinase selectivity (>100-fold to >10000-fold) and cellular activity (IC_50_ < 500 nM). This work will be published separately.

Optimization of the tricycle series focused on two key areas in parallel: 1) isosteres of the carboxylic acid to attenuate the zwitterionic character of **18** and the associated risk of reduced permeability and oral absorption (due to a predominantly charged state at physiological pH);^42,43^ 2) modifications to the linker to remove basic centers introduced during hit identification. Both activities were related to understanding SAR and controlling physicochemical properties as close to drug-like space as possible since we were aware that a bivalent binding mode with large, flexible molecules would most likely result in physicochemical properties close to, or beyond, published guidelines for oral drugs.^40,41^ For these two initial optimization objectives, the biaryl αD fragment was retained but it was recognized that this would subsequently need to be modified.

The first compound synthesized to replace the carboxylic acid was the primary amide (**30**), which retained excellent CK2α potency (IC_50_ = 0.461 nM) but also possessed very high levels of selectivity (>3000-fold) and improved cellular activity (Table 2). The crystal structure of **30** (PDB: 7I7Y) showed that although the direct interaction with Lys68 was lost, it was replaced by a through-water interaction from the −NH_2_ of the primary amide, slightly shifting the position of flexible Lys68 side chain (Figure 2E). The through-water interaction to the acid of Glu81 was retained via the amide carbonyl (Figure 2A and E). All other protein interactions and the overall binding pose of **30 (**PDB: 7I7Y) were identical to **18**. In contrast, the -NMe amide (**31**) had almost 100-fold weaker potency, suggesting that this region of the binding site is sterically sensitive to substitution (Figure 2B and F). This was supported by the acyl sulfonamide (**32**), which despite being a known isostere for a carboxylic acid,^42^ also showed weaker inhibition of CK2α (Figure 2D and H). The tetrazole analogue (**33**) was also synthesized as one of the most-commonly used acid isosteres;^42^ excellent potency and selectivity were observed consistent with its crystal structure that showed **33 (**PDB: 7I8O) retained the same direct salt-bridge interaction to Lys68 as **18** (Figure 2C and G). However, cellular activity was weak, indicating reduced cell permeability, presumably due to increased polarity of the tetrazole.^42^

**Table 2:**
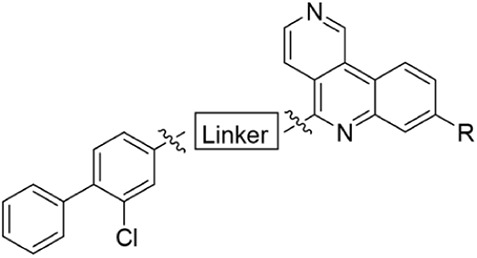

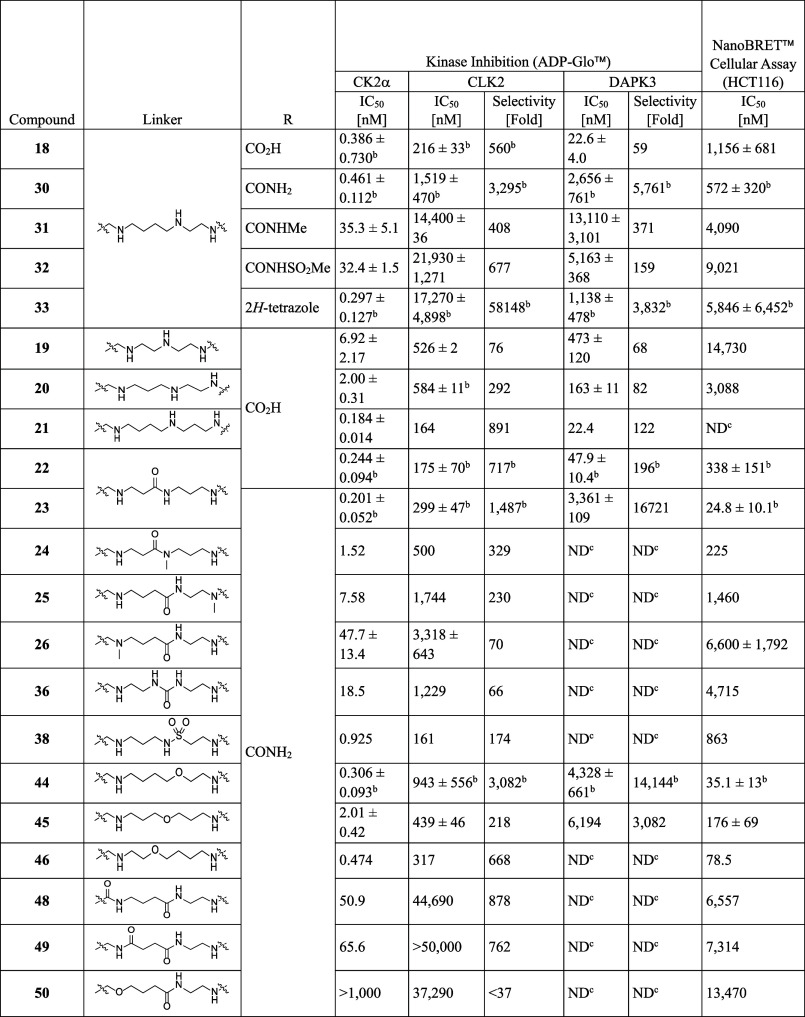
*In Vitro* CK2α Potency, Kinase Selectivity and Cellular Activity of Compounds with Acid Isosteres (**30**–**33**) and Linker Modifications (**19**–**26**, **36**, **38**, **44**–**46** and **49**–**50**)­^a^

a All kinase assays were performed at ATP concentration equivalent to or < K_M_. All data were generated *n* = 1 initially. For compounds with duplicate measurements, data are shown as the mean ± SD from *n* = 2.

b For compounds with further repeats, data are shown as the mean ± SD from *n* = 3–6.

c ND: not determined.

**Figure 2:**
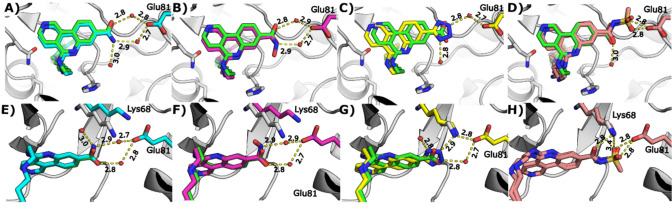
Crystal structures of compounds that show the hydrogen bonding networks of carboxylic acid isosteres. A) and E) The binding mode of **30** (blue, PDB: 7I7Y) compared to **18** (green). B) and F) The binding mode of **31** (pink, PDB: 7I7Z) compared to **18** (green). C) and G) The binding mode of **33** (yellow, PDB: 7I83) compared to **18** (green). D) and H) The binding mode of **32** (salmon pink, PDB: 7I84) compared to **18** (green). Hydrogen bonds are shown as dotted lines.

Initially retaining the carboxylic acid, exploration of the linker length revealed that the distance between the −NH moieties of the tricycle and the benzylic αD fragment was most effectively spanned by 7 (**18**) or 8 atoms (**21**), resulting in high levels of potency and selectivity (Table 2). Linkers containing 5 (**19**) or 6 atoms (**20**) led to 5–18-fold weaker potency, compared with **18**. X-ray crystallography showed that these molecules were anchored by the tricycle in the ATP-binding site, resulting in shorter linkers being unable to reach far enough to optimally position the benzylic NH and bury the biaryl motif as deeply into the αD pocket (Figure 3A–C, E and F). Although longer linkers such as **21** possessed excellent potency, they did not position the αD fragment any further into pocket (Figure 3A, D and G) and as such, contain extra bonds that are tolerated (due to their flexibility) but not required. Based on these observations, further changes to the linker focused on the use of 7 linear atoms (8 bonds) to optimally span the distance between the two binding sites.

**Figure 3:**
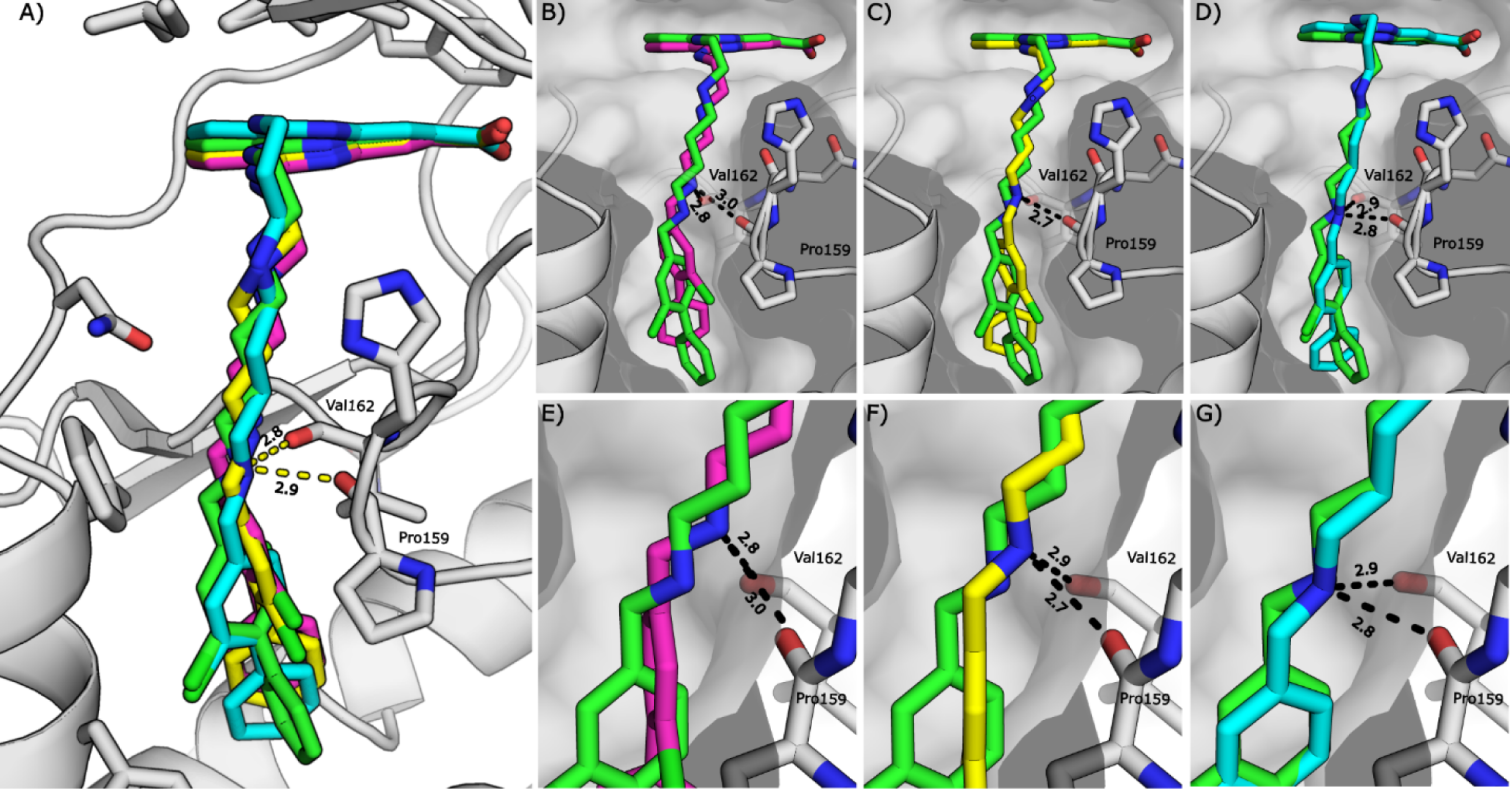
Crystal structures showing compounds with various linker lengths. A) The binding mode of **18** (green) compared to **19** (pink, PDB: 7I80), **20** (yellow, PDB: 7I82) and **21** (blue, PDB: 7I8P). Hydrogen bonds between **18** and Val162 and Pro159 are shown as dotted lines. B) and E) The binding mode of **18** (green) compared to **19** (pink). Hydrogen bonds between **19** and Val162 and Pro159 are shown as dotted lines. C) and F) The binding mode of **18** (green) compared to **20** (yellow). Hydrogen bonds between **20** and Val162 and Pro159 are shown as dotted lines. D) and G) The binding mode of **18** (green) compared to **21** (blue). Hydrogen bonds between **21** and Val162 and Pro159 are shown as dotted lines.

Reintroduction of a secondary amide in the center of the linker (present in **6**) gave **22** as a highly potent and selective compound with good cellular activity. The corresponding compound containing the primary amide (**23**) to replace the carboxylic acid on the tricycle had a very encouraging overall profile that included potent cellular activity (IC_50_ = 25 nM) in the NanoBRET target-engagement assay. Alternative functionality in the linker was also explored to modulate physicochemical properties, for example, incorporation of a central O-atom, sulfonamide or urea, and *N*-methylation along the linker. Of these changes, the O-linked compounds were the most successful, especially **44** which had an excellent overall profile; this approach also had the benefit of removing a basic center and H-bond donor from the center of the linker.

Interesting SAR was observed for the benzylic NH region: all tested modifications led to >100-fold loss in potency, e.g., *N*-methylation (**26**), amide analogues (**48–49**), and replacement with an oxygen atom (**50**). Other changes to the benzylic NH that gave weaker potency included the aniline analogue with transposed CH_2_ and NH groups (not shown). These SAR are explained by the above crystal structure of **18** (Figure 1A) which suggests the benzylic amine is protonated and makes two H-bond interactions to backbone C=O groups of Val162 and Pro159. These interactions anchor the molecule adjacent to the αD pocket – other functionality is unable to do this as effectively. Changes that were tolerated in this region include addition of chiral methyl groups either side of the benzylic amine (not shown), but ultimately these provided no benefit in terms of pharmacology or broader properties.

Having completed initial exploration of the carboxylic acid and linker to produce improved compounds, the next stage of optimization focused on the biaryl motif that bound in the αD pocket. Despite being a largely lipophilic pocket, surrounded by amino acid residues such as leucine, isoleucine, valine, methionine and tyrosine, we were keen to introduce polarity into this region of the molecule – or at least reduce lipophilicity to tune the intrinsic properties that were likely to influence pharmacokinetic (PK) parameters.^40^ Initially, the terminal phenyl was removed completely (**54a**) as a benchmark; measured logD was reduced by 1.4 log units while retaining good potency and excellent selectivity (Table 3). Initially suspecting that only lipophilic substituents would be tolerated, small lipophilic groups were added to the chloro-phenyl ring to probe SAR and minimize impact on logD. In general, good potency, selectivity and cell activity were obtained (e.g., **54b**–**d**, **54f**) combined with reduced logD. The crystal structures of **54e** and **54f** (Figure 4B and C) revealed that the 4-OCF_3_-phenyl occupies the αD pocket in a different orientation to the chloro-biaryl group in **18** (Figure 4A). Loss of the second phenyl group causes Met225 to fill the space and move closer to the ligand. A similar binding mode was observed with the 4-cPr-phenyl analogue **54b** (Figure 4D). The *in vitro* metabolic stability of these compounds was assessed in human liver microsomes (HLM) but despite reductions in logD, rapid metabolism was observed, e.g.: **54f**, logD = 2.3, Cl_int_ = 95 μL/min/mg; **54d**, logD = 1.2, Cl_int_ = 78 μL/min/mg.

**Table 3:**
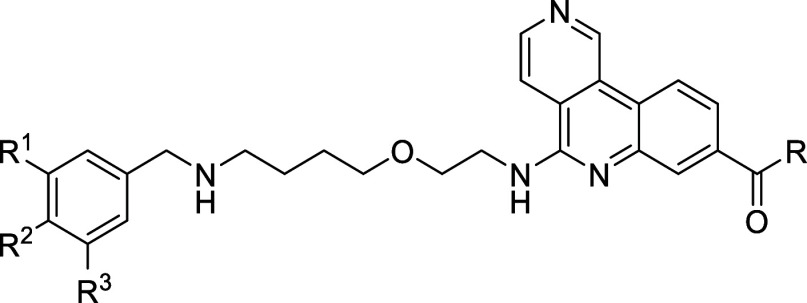

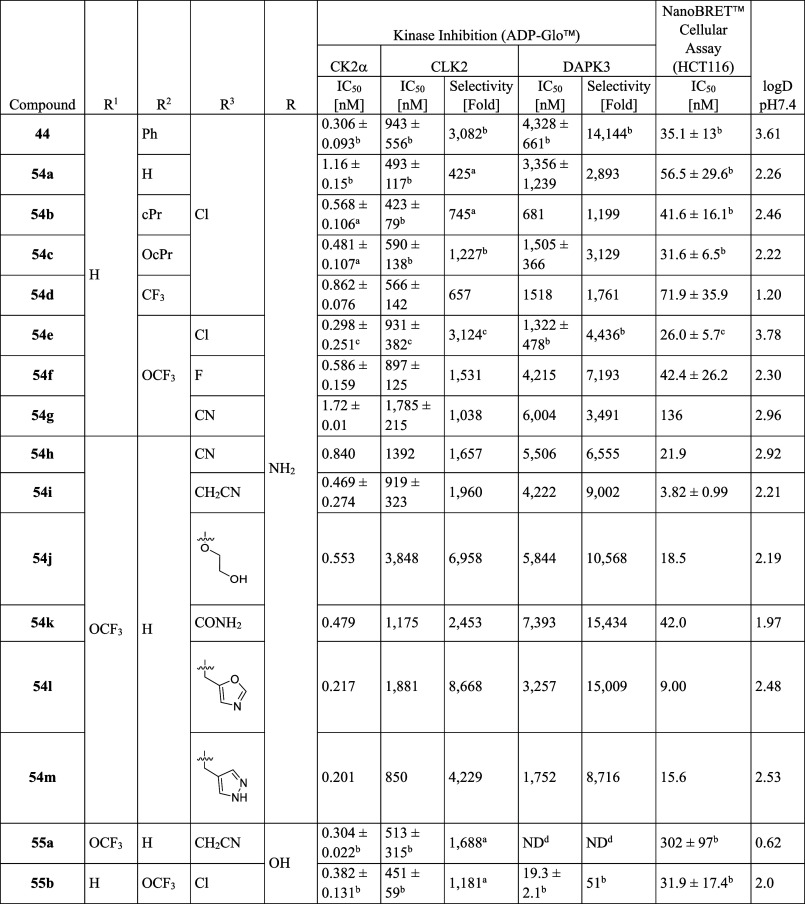
*In Vitro* CK2α Potency, Kinase Selectivity, Cellular Activity and logD of Compounds with Modifications to the αD Fragment (**54a**–**m** and **55a**–**b**)­^a^

a All kinase assays were performed at ATP concentration equivalent to or <*K*_M_. All data were generated *n* = 1 initially. For compounds with duplicate measurements, data are shown as the mean ± SD from *n* =2.

b For compounds with further repeats, data are shown as the mean ± SD from *n* = 3–9.

c For compounds with ten or more repeats, data are shown as the mean ± SD from *n* ≥ 10.

d ND: not determined

**Figure 4:**
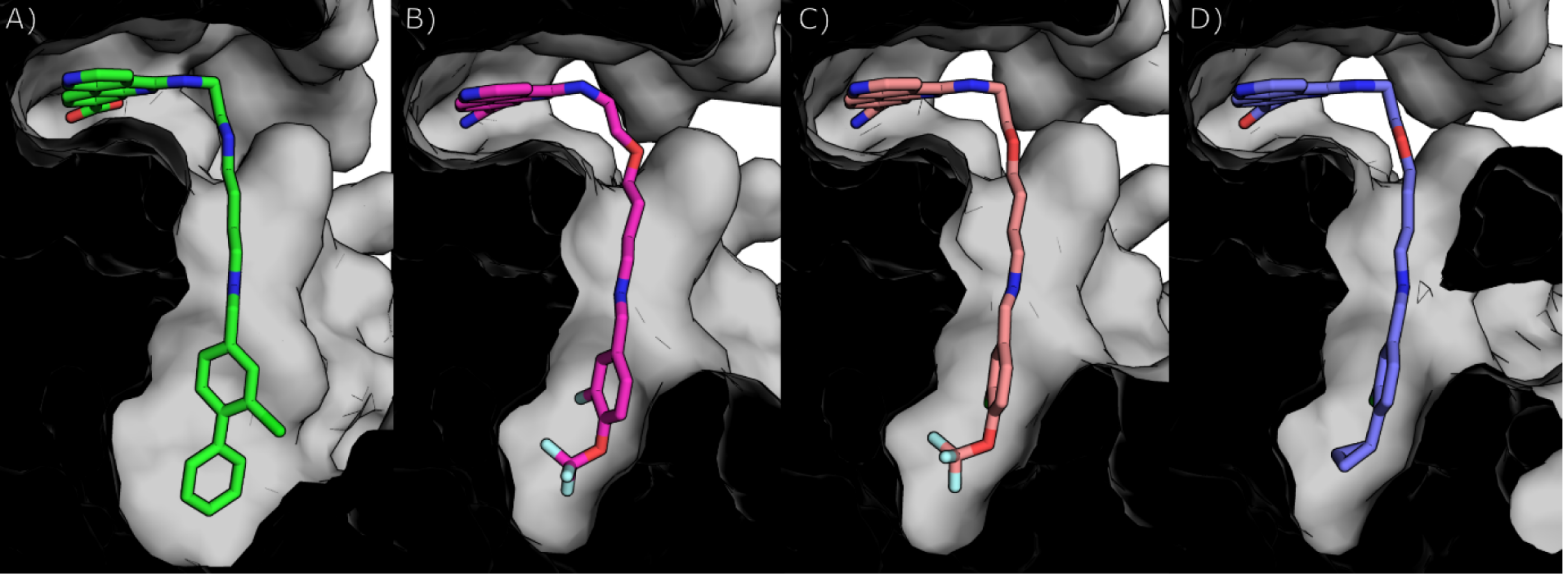
Crystal structures of compounds binding in the αD pocket. Cross-section of the binding modes of: A) **18** (green); B) **54f** (pink, PDB: 7I84); C) **54e** (salmon pink, PDB: 7I89); D) **54b** (purple, PDB: 7I8B).

To improve metabolic stability, the αD fragment was substituted with polar groups. Initially, this strategy led to a slight loss in potency, for example the 3-cyano, 4-OCF_3_ analogue (**54g**) had IC_50_ = 136 nM in the cellular NanoBRET assay, compared to 72 nM for **54d** without 3-cyano modification. However, moving the −OCF_3_ group to the 3-position successfully allowed polar substituents to be introduced at the 5-position to reduce logD and retain excellent levels of potency, selectivity and cellular activity. For example, the pair of cyano-analogues **54g** and **54h** show the effect of this change on cell potency in the NanoBRET assay. A variety of other polar groups were successfully incorporated in this position; a selection of those synthesized are shown in Table 3, including −OCH_2_CH_2_OH (**54j**), −CONH_2_ (**54k**) and heterocycles such as oxazole (**54l**) and pyrazole (**54m**). In particular, substitution with −CH_2_CN (**54i**) exhibited very potent cell activity combined with high kinase selectivity and logD of 2.21. Overall, these compounds illustrated that some polarity is tolerated in the αD pocket, which can drive very potent and selective CK2α pharmacology. To understand how these polar groups were accommodated within the binding pocket, crystal structures were obtained for several analogues, including **54k**–**m**. Structural data for **54l** revealed that the 3-OCF_3_ group occupies a similar position to that seen for the 4-OCF_3_ group in **54f** (Figure 5A vs 5B and 5E vs 5F). To do so, the phenyl group must twist, projecting the 5-CH_2_–oxazole substituent to the other side of the αD pocket, displacing Tyr125. This enlarges the αD pocket and positions the oxazole adjacent to a water network which is present in all CK2α crystal structures but not usually accessible from the αD pocket because it is blocked by Tyr125, which forms a H-bond with the first water molecule of the network. When **54l** is bound, the oxazole N atom makes the H-bond with the first water of that network. This new binding mode in the αD pocket was also observed with **54k** and **54m** (Figure 5C and D) and provides a rationale for the high level of CK2α potency exhibited by compounds that contain polar groups with this aromatic substitution pattern. The metabolic stability of these more polar compounds was assessed *in vitro* in HLM but despite reductions in logD, intrinsic clearance remained very high, ranging from 115 to 736 μL/min/mg. Despite encouraging pharmacology, it was clear that a different strategy was required to provide compounds with good metabolic stability.

**Figure 5:**
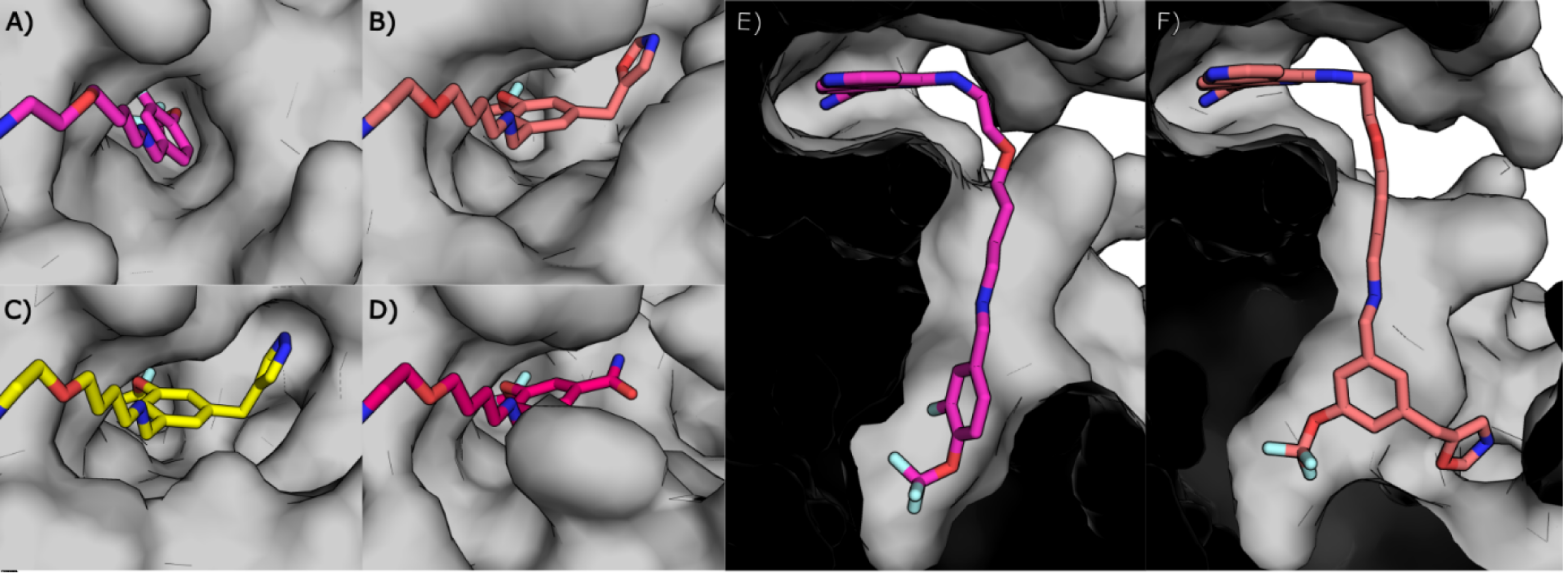
Crystal structures of compounds that bind in the αD pocket and grow into the adjacent water channel. αD pocket with a closed water channel: A) The binding mode of **54f** (pink, PDB: 7I8A); αD pockets with an open water channel:B) The binding mode of **54l** (salmon pink, PDB: 7I8Q); C) The binding mode of **54m** (yellow, PDB: 7I8R); D) The binding mode of **54k** (dark red, PDB: 9QQX); E) Cross-section of the binding mode of **54f** with a closed water channel ( pink, PDB: 7I8A); F) Cross-section of the binding mode of **54l** with an open water channel (salmon pink, PDB: 7I8Q).

Further optimization efforts within the tricyclic series containing our preferred primary amide in the ATP-binding site were not able to combine potent activity with good *in vitro* metabolic stability. Several unsuccessful strategies were attempted, for example: a) replacing phenyl with heterocycles in the αD fragment to reduce logD (→ weaker enzyme/cell potency); b) substituting adjacent to the benzylic amine in an attempt block metabolic cleavage of the αD fragment, which was a major route of metabolism in human hepatocytes (→ poor metabolic stability); c) incorporation of polar heterocycles in the linker to reduce logD (→ weaker cell potency); d) alternative tricycles with additional heteroatoms to reduce logD (→ weaker cell potency). Consequently, it was decided to reevaluate tricycles containing a carboxylic acid to reduce logD more effectively, while recognizing that this would return compounds to zwitterionic property space and potential permeability issues.^40,41^ Compounds were initially synthesized with the O-containing linker and a variety of polar and nonpolar αD fragments. Compounds with polar groups immediately gave excellent enzyme potency with low logD but cellular potency was compromised, presumably due to impaired cell penetration; e.g., acid **55a** exhibited ∼100-fold weaker cell potency (NanoBRET) than corresponding amide **54i**. However, compounds containing an acid and a less-polar αD fragment were able to deliver excellent enzyme potency and retain potent cellular activity; e.g., acid **55b** exhibited similar cell potency (NanoBRET IC_50_ = 32 nM) to corresponding amide **54e**, indicating that cell permeability could be achieved for a zwitterion with logD of 2.0. Encouragingly, **55b** also demonstrated much-improved metabolic stability in HLM compared to amide analogue **54e**; Cl_int_ = 24 vs 142 μL/min/mg, respectively. This was an important breakthrough for the project, indicating that the desired combination of potent cell activity and good metabolic stability was achievable in this scaffold. Consequently, further efforts were focused on optimization of this zwitterionic series.

Further investigation of the linker was conducted to optimize physicochemical properties for cell penetration and potency. Particular effort was made to rigidify the linker and reduce properties such as TPSA and H-bond donors/acceptors.^40,41^ A broad set of compounds were synthesized to assess the impact of these changes in different areas of the linker; selected compounds are shown in Table 4. Addition of a single methyl group adjacent to the tricyclic NH maintained excellent potency (e.g., **58a**–**b**) and good metabolic stability in both stereochemical configurations. Accordingly, dimethyl analogue **58c** was also very potent, especially in cells, but much higher logD led to high turnover in HLM. Incorporation of a heterocyclic ring in the linker gave several compounds with good CK2α enzyme potency, for example, azetidine **58d**, pyrrolidines **58e**–**f** and piperidine **58g**. Crystal structures were obtained for a selection of compounds (**58b** and **58d**–**f** vs **55b**), which confirmed that adding rigidity to the linker still allowed it to make the required 90° turn toward the αD pocket (Figure S2). However, despite rigidification and removal of an NH that were designed to enhance cell permeability, these analogues displayed slightly weaker cell potency. Stability in HLM was improved in some cases, e.g., azetidine **58d**, which was completely stable and had reduced logD (1.72) despite the addition of a CH_2_ group and removal of an NH (compared with **55b**). Replacement of the tricyclic NH with an oxygen atom gave **61a**, which was more potent in cells than corresponding NH-analogue **55b**, with similar metabolic stability. Monomethylation adjacent to the oxygen atom gave compounds **58h**–**i** with similar potency but increased logD and metabolism in HLM. Overall, **61a** appeared to give the best combination of cell potency, logD and stability in HLM, although selectivity against DAPK3 was compromised, which seemed to partly be a feature of moving back into the zwitterionic series.

**Table 4:**
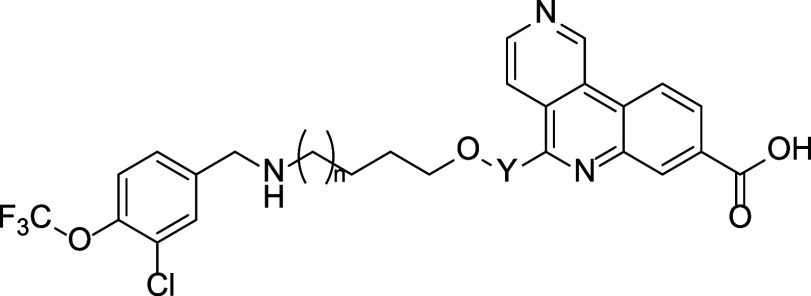

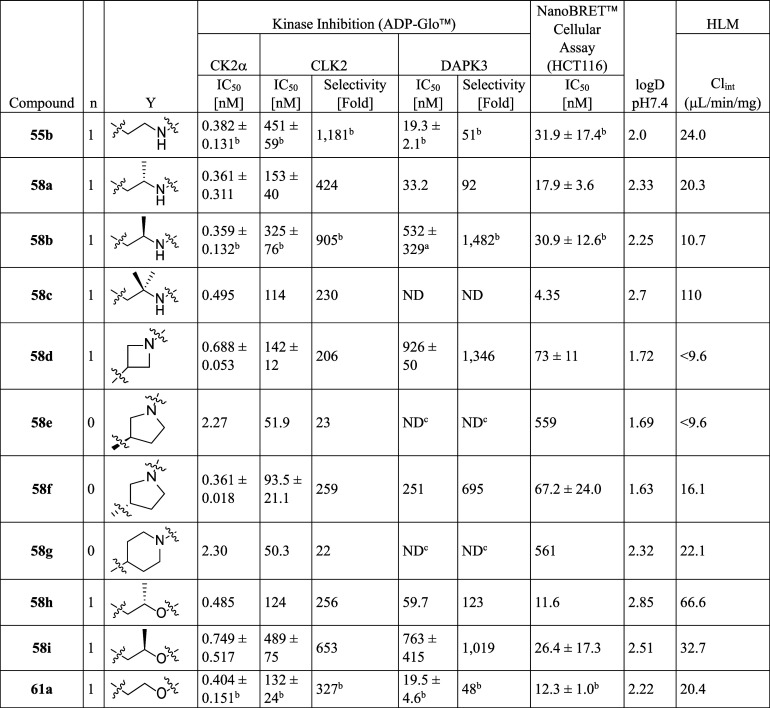
*In Vitro* CK2α Potency, Kinase Selectivity, Cellular Activity, logD and HLM Stability of Zwitterionic Compounds Incorporating Changes to the Ether Linker (**58a**–**i** and **61a**)­^a^

a All kinase assays were performed at ATP concentration equivalent to or <*K*_M_. All data were generated *n* = 1 initially. For compounds with further repeats, data are shown as the mean ± SD from*n* = 2.

b Data are shown as the mean ± SD from *n* = 3–10.

c ND: not determined.

Finally, additional optimization of the αD fragment through small modifications to **61a** were performed to identify lead compounds from the series with the best balance of metabolic stability, cellular potency and selectivity. Small changes were made to the substitution pattern of the aromatic ring, while retaining the −OCF_3_ motif (Table 5). Replacement of chloro with fluoro (**61b**) or methyl (**61c**) gave compounds with similar potency to **61a**, improved DAPK3 selectivity and complete stability in HLM. The 3,5-difluoro-4-OCF_3_ analogue (**61f**) exhibited the most potent cell activity (NanoBRET IC_50_ = 5.56 nM) combined with high kinase selectivity (>350-fold) and good stability in HLM. Compounds were also synthesized containing the regioisomeric 3-OCF_3_ group combined with small substituents in the 4- or 5-position. These analogues (e.g., **61g**–**i**) had excellent cell potency but slightly higher clearance in HLM. More polar substituents such as methoxy (**61d**) or −CH_2_CN (**61j**) had potent enzyme activity, low logD and stability in HLM, but suffered from weaker cell potency, again illustrating that polar αD fragments combined with the tricyclic acid were likely too polar and did not quite deliver the required profile. Overall, **61f** was determined to have the best *in vitro* profile and was advanced to further evaluation, including PK studies and broader profiling.

**Table 5:**
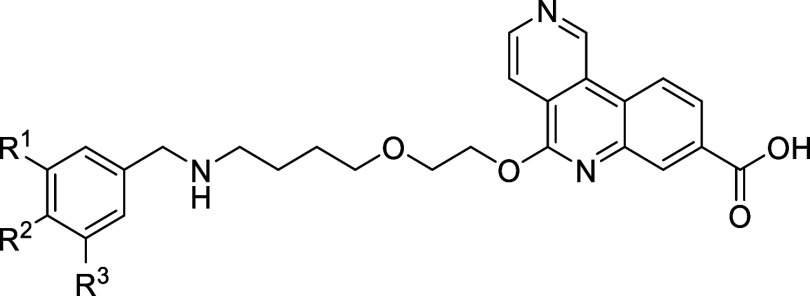
*In Vitro* CK2α Potency, Kinase Selectivity, Cellular Activity, logD and HLM Stability of Zwitterionic Compounds with Modifications to the αD Fragment (**61b**–**j**)­^a^

				Kinase Inhibition (ADP-Glo^TM^ Assay)							HLM
				CK2α	CLK2		DAPK3		NanoBRETCellular Assay (HCT116)		
Compound	R^1^	R^2^	R^3^	IC_50_ [nM]	IC_50_ [nM]	Selectivity [Fold]	IC_50_ [nM]	Selectivity [Fold]	IC_50_ [nM]	logD pH 7.4	Cl_int_ (μL/min/mg)
**61a**	H	OCF_3_	Cl	0.404 ± 0.151^b^	132 ± 24^b^	327^b^	19.5 ± 4.6^b^	48^b^	12.3 ± 1.0^b^	2.22	20.4
**61b**			F	0.354 ± 0.149^b^	161 ± 82^b^	455^b^	48.8 ± 15^b^	138^b^	13.8 ± 3.0^b^	1.8	<9.6
**61c**			Me	0.370 ± 0.057^b^	190 ± 109^b^	514^b^	64.2 ± 37	174	15.2 ± 1.2	1.8	<9.6
**61d**			OMe	0.403	283	702	n.d.	n.d.	164	1.42	<9.6
**61e**			cPr	0.432 ± 0.069	288 ± 232	667	42.6	99	15.3	2.16	31
**61f**	F	OCF_3_	F	0.348 ± 0.127^c^	363 ± 187^c^	1043^c^	123 ± 50^c^	353^c^	5.56 ± 1.75^c^	2.12	14.2
**61g**	H	Cl	OCF_3_	0.409 ± 0.123^b^	124 ± 23^b^	303^b^	33.1 ± 7.9	81	6.30 ± 0.28	2.07	25.6
**61h**	OCF_3_	H	Cl	0.342 ± 0.112	82 ± 51	241	126	368	8.64	2.09	39.3
**61i**			cPr	0.366 ± 0.213^b^	284 ± 256^b^	776^b^	89.7 ± 32^b^	245^b^	9.39 ± 6.25^b^	2.37	23.2
**61j**			CH_2_CN	0.354	202	571	n.d.	n.d.	71.4	1.04	14.2

a All kinase assays were performed at ATP concentration equivalent to or <*K*_M_. All data were generated *n* = 1 initially. For compounds with duplicate measurements, data are shown as the mean ± SD from *n* = 2.

b For compounds with further repeats, data are shown as the mean ± SD from *n* = 3–9.

c For compounds with further repeats, data are shown as the mean ± SD from *n* = 10.

d ND: not determined.

The IC_50_ for many of the compounds made during this project were at the tight binding limit of the CK2α kinase assay. This did not negatively impact SAR assessment since the nanoBRET assay results were most critical for decision-making and compound progression. However, to confirm absolute potency and selectivity of **61f** and silmitasertib (**1**), *K*_i_ values were determined across the ADP-Glo^TM^ assays. This confirmed very potent inhibition of CK2α by **61f** (0.095 nM) and high levels of selectivity (632–10547-fold) over the four other kinases profiled, which was far superior to silmitasertib (**1**) (Table 6). The binding kinetics of **61f** to CK2α were assessed using surface plasmon resonance (SPR), showing a potent *K*_d_ value (1.4 nM) and, in line with this, a slow rate of dissociation (*K*_off_ = 1.1 × 10^–3^ s^–1^) resulting in a long CK2α residence time (*t*_R_ = 15.2 min).^44,45^ The off-rate of **61f** was three times slower than that observed for **1** (Table 6, Figure S4), most likely due to its bivalent binding mode, which was confirmed by the crystal structure of **61f** bound to CK2α (Figure 6). Key ATP-binding site interactions are retained, including the salt-bridge interaction between the carboxylic acid of **61f** and Lys68 (Figure 6F), and the H-bond between the tricycle pyridine and the N–H of Val116 in the hinge region (Figure 6C). The linker exits the ATP-binding site with a 90° turn and extends toward the αD pocket (Figure 6A), retaining the key H-bond interactions between the benzylic amine and backbone C=O groups of Val162 and Pro159 (Figure 6E). These interactions anchor the molecule and bury the aryl group deep into the αD pocket (Figure 6D). A summary of the binding interactions between **61f** and CK2α is illustrated in Figure 6G.

**Table 6:** *In Vitro* Profile of **61f** in CK2α Biochemical, Biophysical and Cellular Assays, and Selectivity versus Key Kinases^a^

	Kinase Inhibition (ADP-Glo^TM^ Assay)					Binding Kinetics (SPR)			Cellular Assays (HCT116)		
	CK2α	CLK2	DAPK3	HIPK3	DYRK2	CK2α			NanoBRET	Proliferation	p-AKT S129
Compound	*K*_i_ [nM]	*K*_i_ [nM]/Selectivity [Fold]				*K*_d_ [nM]	*K*_on_ [M s^–1^]	*K*_off_ [s^–1^]	IC_50_ [nM]		
**61f**	0.095 ± 0.052^b^	129 ± 67^b^/1358^b^	60 ± 25^b^/632^b^	1,002 ± 550/10,547	833 ± 305/8,768	1.4 ± 0.65	1.1 × 10^6^	1.1 × 10^–3^	5.56 ± 1.75	325 ± 110	27.8 ± 9.1
**1** (CX-4945, Silmitasertib)	0.263 ± 0.090^c^	1.8 ± 0.5^c^/19^c^	2.5 ± 0.6^b^/26^b^	15 ± 4.5^b^/152^b^	16 ± 3.9^b^/166^b^	7.6 ± 1.2	1.5 × 10^6^	3.8 × 10^–3^	107 ± 45^c^	3.722 ± 730	4.390 ± 3.968

a All kinase assays were performed at ATP concentration equivalent to or <*K*_M_. Data are the mean ± SD from *n* = 5–19.

b Data are the mean ± SD from *n* = 21–99.

c Data are the mean ± SD from *n* > 100.

**Figure 6:**
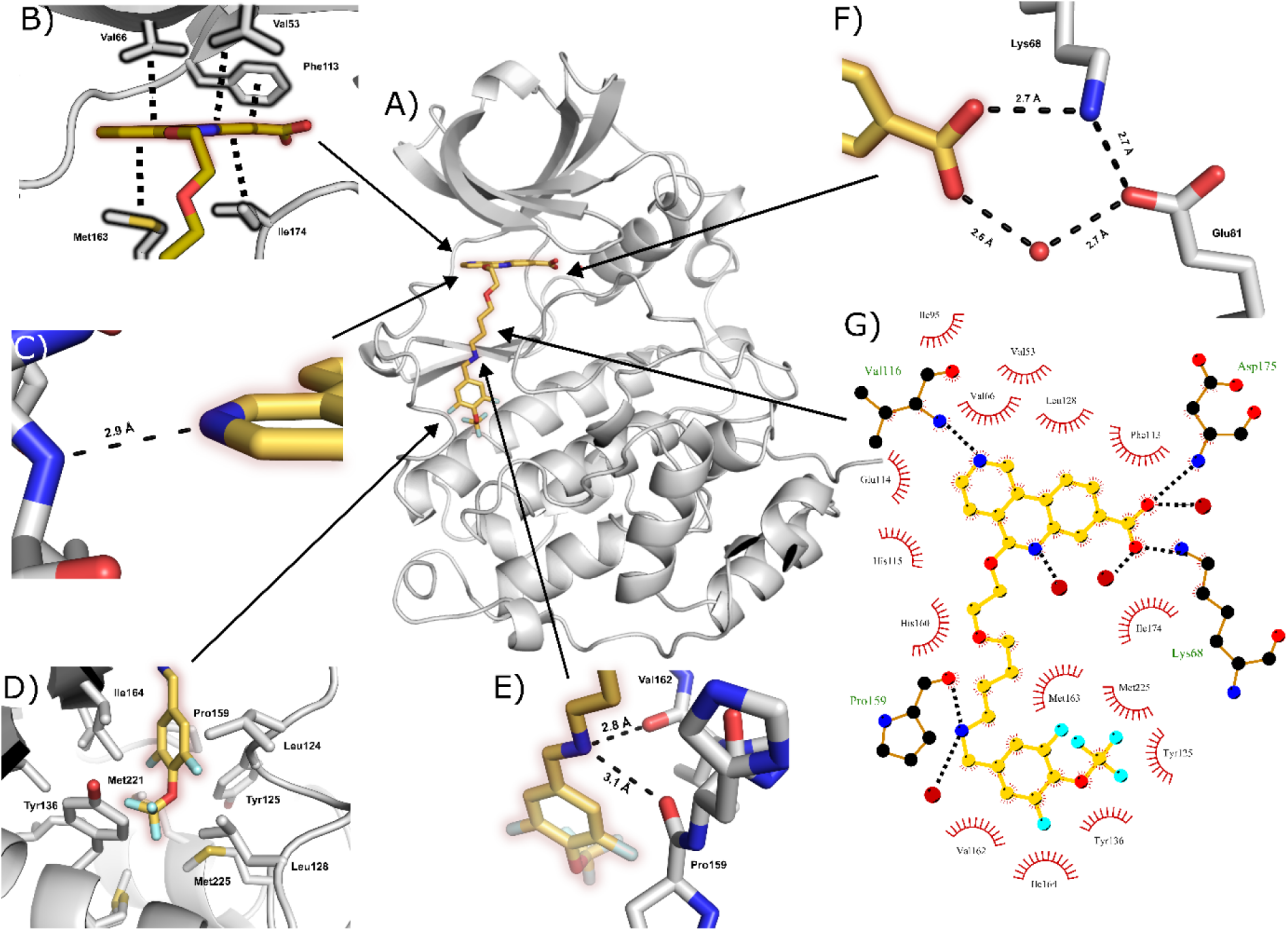
Binding mode of **61f** to CK2α. A) The crystal structure of **61f** bound to CK2α (PDB: 7I8K). B) The interactions of **61f** within the ATP-binding site. C) The hydrogen bonding interactions between **61f** and the hinge region. D) The hydrophobic interactions between **61f** and the αD pocket. E) The hydrogen bonds between the benzylamine and CK2α. F) The hydrogen bonding network between the carboxylic acid group of **61f**, Lys68, Glu81 and a water molecule. G) Two-dimensional map of the interactions between CK2α and **61f** (gold) with hydrogen bonds shown as dotted lines, and hydrophobic interactions depicted with spiked arcs (or “eyelashes”).

Further profiling in the HCT116 cell line was conducted to assess levels of phospho-AKT (p-AKT) Ser129 (S129) as a direct product of phosphorylation by CK2α on S129 of AKT. These data confirmed very potent cellular inhibition of CK2α that was >150-fold more potent than silmitasertib (**1**) (Table 6). To fully assess its kinase selectivity profile, **61f** was screened at 100 nM (i.e., 1000-fold CK2α K_i_) against 468 kinases using the KINOME*scan* ^TM^ scanMAX panel (Figure 7). The panel screen results confirmed potent activity of **61f** against both CK2α and CK2α′, *K*_d_ values for the two isoforms were within 3-fold of each other. Excluding CK2α/CK2α’ as hits, only 5/403 nonmutant kinases showed >65% inhibition, resulting in an excellent selectivity S-score of *S*(35) = 0.012. Follow-up determination of *K*_d_ values confirmed that **61f** had >175-fold selectivity over the nearest kinases DAPK1 and DAPK3, which was consistent with in-house data (Table 6). **61f** was also screened at 1 μM against a panel of 87 enzymes, ion channels, receptors and transporters (SafetyScreen87^TM^) but did not show activity >30% against any target.

**Figure 7:**
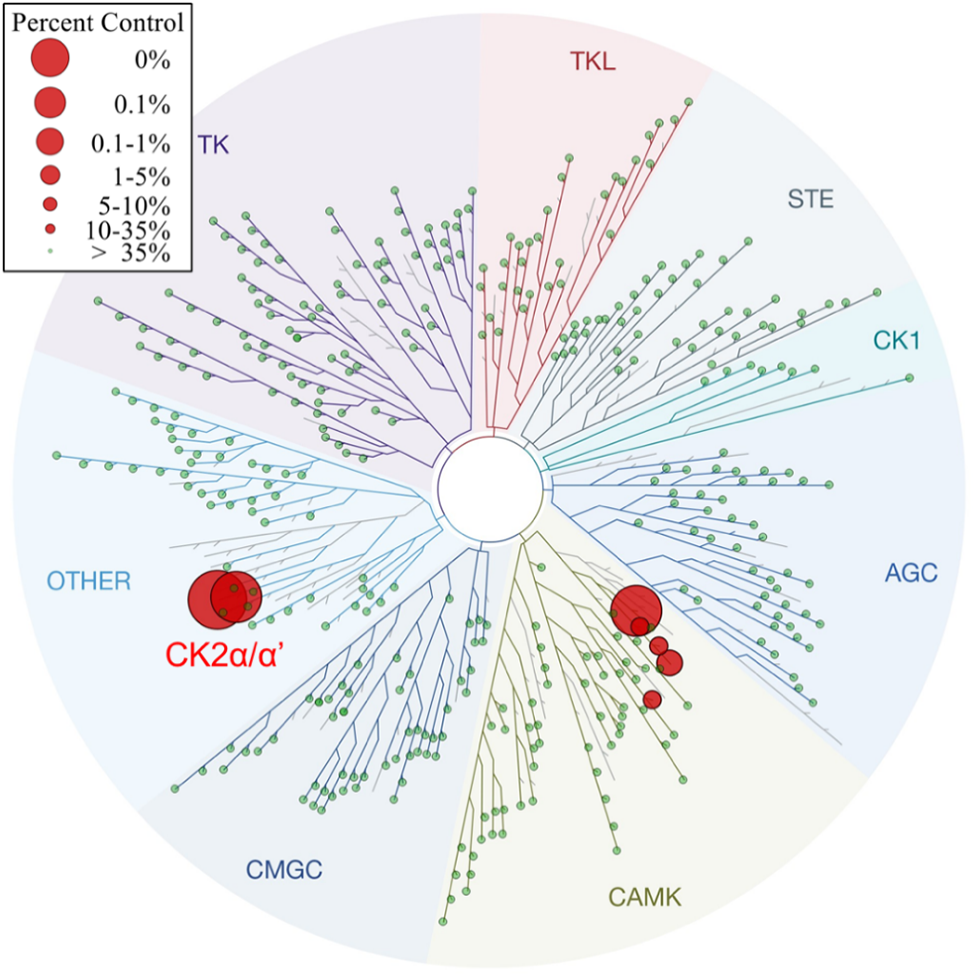
Profile of **61f** in the KINOME*scan* ^TM^ scanMAX panel. The human kinome is represented as a phylogenetic tree. Each kinase is shown as a sphere with size and color indicating the level of inhibition of each kinase at 100 nM APL-5125, as per legend on the top left corner.

The *in vitro* ADME profile of **61f** is summarized in Table 7. **61f** possessed good stability in human and dog liver microsomes and moderate stability in rodent. A similar profile was observed in hepatocytes, except for dog, which showed higher clearance in hepatocytes than microsomes. Overall, metabolism of **61f** in human hepatocytes included oxidation, dealkylation of the αD fragment and linker, and glucuronidation; the major metabolite was a direct glucuronide, that was presumed to form on the carboxylic acid. The human cytochrome P450 (CYP) enzymes responsible for oxidative metabolism were mostly CYP3A4 and, to a lesser extent, CYP2C8. Glucuronidation was mediated mainly by human UDP-glucuronosyltransferases (UGT) UGT1A1 and UGT1A3. This combination of Phase 1 and Phase 2 metabolism indicates that **61f** is unlikely to be the victim of a drug–drug interaction (DDI) if coadministered with other drugs. In Caco-2 cells, **61f** had moderate passive permeability with an efflux ratio of 9.1, suggesting the involvement of efflux transporters such as P-glycoprotein (P-gp) or breast cancer resistance protein (BCRP) – previous data from cellular pharmacology assays indicated that passive permeability was not an issue. Caco-2 data generated for a range of compounds showed that this profile was a general feature of the pharmacophore and was present in compounds with and without a carboxylic acid (e.g., **54e**, **54i**). Additional studies in Caco-2 cells in the presence of verapamil (P-gp inhibitor) or novobiocin (BCRP inhibitor) indicated that **61f** was a substrate for P-gp. Permeability data in MDCK-MDR1 cells (Madin-Darby canine kidney (MDCK) cells transfected with the human multidrug resistance 1 (*MDR1*) gene (*ABCB1*) encoding P-gp)^46^ confirmed **61f** was a substrate for P-gp (*P*_app_ A–B/B–A = 5.0/22.6 × 10^–6^ cm/s; ER = 4.5). Evaluation in a panel of additional transporters (e.g., organic anion transporters [OAT], organic cation transporters [OCT], bile salt export pump [BSEP]) showed that **61f** was not a substrate of these transporters. Thermodynamic solubility of **61f** was generally low at physiological pH, in line with its zwitterionic character (measured p*K*_a_ = 3.71 and 8.04) and physicochemical properties beyond the Rule-of-5 (MW = 565, cLogP = 5.3).^40^ To understand the impact of low solubility and permeability on oral absorption, **61f** was progressed to *in vivo* PK studies.

**Table 7:** *In Vitro* ADME Profile of 61f^a^

	Metabolic Stability (Cl_int_)^b^				Permeability (Caco-2 Cells)^c^			Thermodynamic Solubility [μg/mL]				
	Liver Microsomes [μL/min/mg]/Hepatocytes [μL/min/10^6^ cells]				*P*_app_ [× 10^–6^ cm/s]			PBS; pH			FaSSIF	FeSSIF
Compound	Human	Mouse	Rat	Dog	A–B	B–A	Efflux Ratio (ER)	1.0	7.4	10.0	6.5	5.0
**61f**	14.2/11.8	45.7/44.9	36.8/75.3	<9.6/57.7	2.8	25.7	9.1	256	<0.9	<0.9	1.6	10.7

a FaSSIF: Fasted state simulated intestinal fluid; FeSSIF: Fed state simulated intestinal fluid; PBS: phosphate buffered saline.

b Cl_int_ values in human liver microsomes and human hepatocytes were derived from the mean of *n* = 2 experiments; for all other species, Cl_int_ values were determined from *n* = 1.

c *P*_app_ and ER were determined from the mean of *n* = 2 experiments in Caco-2 cells with each experiment containing duplicate wells per test condition.

In all species, **61f** displayed a low volume of distribution (typical for drugs containing carboxylic acids),^47,48^ low clearance and moderate half-life (Table 8). Oral absorption was initially assessed as a solution in rats using a standard vehicle. Oral bioavailability of 16% and 36% was observed in male and female rats, respectively (Table 9), correlating with oral absorption (FaFg) of 20–40%. These data illustrated that oral absorption was achievable despite concerns relating to poor solubility and transporter-mediated efflux. To evaluate the potential for increased oral absorption, additional PK studies were conducted in rats with a variety of oral formulations selected for their potential to enhance solubilization and/or permeability (Table 9). The best result was observed with a formulation containing Kolliphor (Cremaphor) EL, a clinically used enhancer of solubility and permeation (as a P-gp inhibitor).^49^ In male rats, this formulation gave a 4-fold improvement, resulting in high oral bioavailability and absorption of 66% and 78%, respectively. Single-dose oral PK studies were conducted in dogs using capsules containing 100 mg of **61f** (per dog) in formulations with and without Kolliphor EL. Similar to the results seen in rats, oral exposure of **61f** was improved with formulations containing Kolliphor EL: oral bioavailability improved 3.5-fold from 22% (**61f** alone) to 76%, representing 88% oral absorption, with a *T*_max of_ 1.2 h. IV and oral PK studies in rats and dogs also showed that excretion of unchanged **61f** into feces was a significant route of clearance, consistent with hepatobiliary excretion of drugs containing carboxylic acids.^47^ In rats, 62% and 37% of **61f** was recovered in feces, following IV and oral administration, respectively; in dogs, these values were 27% (IV) and 39% (oral). Renal clearance was detectable but minimal. Plasma protein binding of **61f** was high in all species (e.g., human = 99.8%), again consistent with compound physicochemistry.

**Table 8:** IV Pharmacokinetic Parameters of **61f** in Mouse, Rat and Dog^a^

Species (Breed)	Sex	Dose [mg/kg]	Cl [mL/min/kg]	Vd_ss_ [L/kg]	*T*_1/2_ [h]
Mouse (BALB/c)	F	3	12.6	0.32	1.6
Rat (SD)	F	3	5.1	0.33	2.8
	M	3	8.8	0.44	2.3
Dog (Beagle)	M	3	2.8	0.35	3.8

a Pharmacokinetic studies were performed with *n* = 3 animals per group. Vehicle was 10% DMSO and hydroxypropyl-beta cyclodextrin 20% in water (2:98, v/v).

**Table 9:** Oral Pharmacokinetic Parameters of **61f** in Rat, Using Different Formulations^a^

Species (Breed)	Sex	Dose [mg/kg]/Concentration [mg/mL]	Vehicle	*T*_max_ [h]	*F* [%]	FaFg [%]
Rat (SD)	M	3/0.3	10% DMSO, 18% HP-β-cyclodextrin, 72% water (solution)	0.25–1.0	16.5	20
	F	5/0.6		0.5–1.0	36.1	40
	M	1/0.1	20% HP-β-cyclodextrin in water, pH 5 (solution)	0.5	27.4	33
	M	10/1		0.5	24.3	29
	M	10/1	30% PEG 400, 20% Kolliphor EL, 50% water, pH 4 (solution)	0.25	65.6	78
	M	10/1	0.5% (w/v) methylcellulose, 0.2% (v/v) Tween 80 in water (suspension)	0.25–0.5	12.7	15

a Pharmacokinetic studies were performed with *n* = 3 animals per group.

The *in vivo* pharmacology of **61f** was assessed in mice bearing tumors grown following inoculation with the human CRC HCT116 cell line. Once-daily oral administration of 10, 30, and 100 mg/kg **61f** was performed for 21 days using a nonoptimized formulation. After the final day of dosing, statistically significant tumor-growth inhibition (TGI, 45%, *p* = 0.0027) was observed at 100 mg/kg. Pharmacokinetic-pharmacodynamic (PK–PD) analysis of samples taken at 2- and 8 h postdose, showed that mean unbound plasma concentrations of **61f** were 46 and 5 nM, respectively, correlating with mean 72% (*p* < 0.0001) and 46% (*p* = 0.0015) inhibition of p-AKT S129 in tumor samples. Analysis of unbound concentrations of **61f** in tumor samples showed similar levels to plasma, which gave an *in vivo* IC_50_ of 2.9 nM (Figure 8). Overall, these data for **61f** demonstrated an excellent *in vitro*-*in vivo* translation and a clear relationship between unbound plasma exposure (PK), unbound tumor exposure, inhibition of tumor p-AKT S129 (PD) and TGI (efficacy). Furthermore, **61f** was shown to potently inhibit p-AKT S129 (IC_50_ = 2 nM) *in vitro* in human peripheral blood mononuclear cells (PBMC), providing a clinically relevant PK–PD biomarker of CK2α inhibition in humans (data not shown).

**Figure 8:**
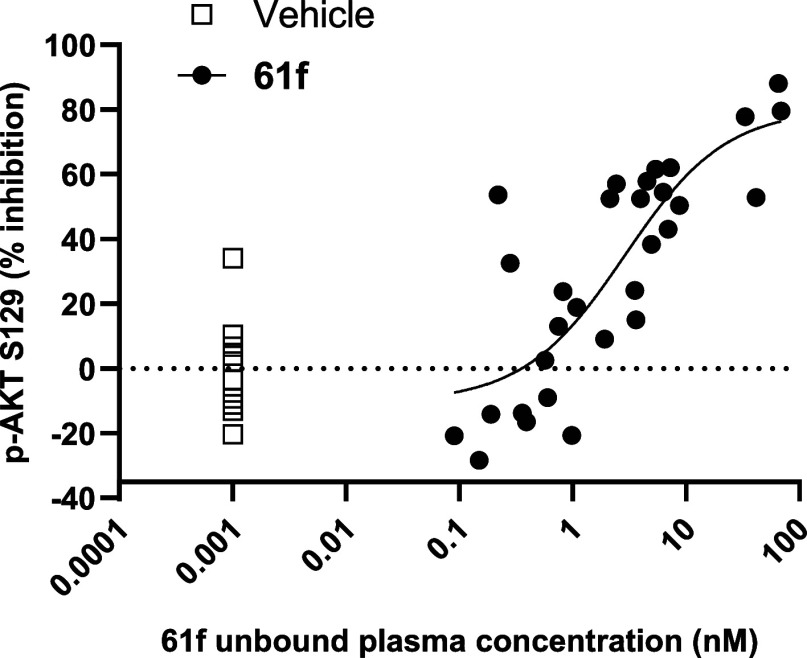
*In vivo* inhibition of p-AKT S129 in HCT116-tumors in mice following oral administration of **61f**. Concentration–response curve for p-AKT S129 inhibition in tumor was generated using the unbound concentration of **61f** in plasma from all individual mice in each dose group (10, 30, 100 mg/kg) at either 2 or 8 h postfinal dose, and the corresponding p-AKT S129 measurement from tumor tissue determined by Western blotting.

**61f** was shown to have a clean safety profile when tested in a variety of additional *in vitro* assays: no direct or time-dependent inhibition (IC_50_ > 25 μM) or induction of CYP enzymes, indicating a low risk of causing DDI if coadministered with other drugs; no pharmacologically relevant inhibition of the hERG cardiac ion channel (IC_50_ = 28 μM) in a GLP patch-clamp electrophysiology assay. Furthermore, **61f** has completed GLP *in vivo* toxicology studies in rats and dogs. **61f** is a highly crystalline zwitterion with suitable material properties for further development and has been synthesized up to 4 kg under GMP (good manufacturing practice) conditions. In humans, **61f** is predicted to have a low volume of distribution, low clearance and suitable half-life to support once-daily or twice-daily oral dosing.

## Conclusion

Capitalising on the earlier discovery of the cryptic αD pocket^29,30^ we have developed a series of highly potent and highly selective CK2α inhibitors. Compounds from this series bind to CK2α in a bivalent manner, simultaneously binding to the ATP-binding site (potency) and αD pocket (selectivity), to afford the desired *in vitro* profile. Protein–ligand X-ray crystallography was used extensively (238 structures) and was pivotal to the success of the overall project strategy. In particular, this crystallography-driven approach enabled: a) identification of the most attractive starting points and hit molecules; b) interpretation of SAR and optimization of interactions in both binding sites. The design of bivalent molecules results in relatively large, flexible molecules with physicochemical properties beyond recommended guidelines for oral drugs,^40,41^ bringing associated concerns regarding permeability and solubility. Despite these challenges, by exploring extensive SAR across the bivalent scaffold and modulating physicochemical properties where tolerated, we have identified compounds with potent cellular activity and good *in vitro* ADME profiles. Of these molecules, **61f** (APL-5125) emerged as a candidate molecule that demonstrates potent *in vivo* inhibition of p-AKT S129 in tumors in mice following once-daily oral administration. This *in vivo* effect correlates with unbound drug concentration in both tumor and plasma, demonstrating a clear PK–PD relationship. Furthermore, **61f** potently inhibits p-AKT S129 in human PBMC, providing a clinical biomarker of CK2α inhibition in humans.

Overall, **61f** is a highly potent, exquisitely selective, ATP-competitive inhibitor of CK2α with an optimized bivalent binding mode extending to the cryptic αD pocket. **61f** has a superior preclinical profile to existing CK2α inhibitors, including silmitasertib (**1**), and has completed regulatory GLP safety studies. **61f** (APL-5125) has the potential to be the best-in-class CK2α inhibitor and is currently under evaluation as an oral treatment in a Phase 1/2 study in patients with advanced solid tumors (e.g., CRC).^36^

## Experimental Section

### Chemistry

All commercially available solvents and chemicals were used as purchased without purification. Thin-layer chromatography was performed on precoated Merck silica gel plates (with fluorescence indicator UV_254_). Column chromatography was performed using Merck silica gel (40–63 μm). Proton (^1^H) NMR spectra were acquired on a Bruker Avance III 400 spectrometer in the deuterated solvents specified. Spectra were processed using interpretation software ACD Spectrus or equivalent. Chemical shifts are reported in parts per million (ppm) (δ relative to residual nondeuterated solvent peak). Exchangeable protons that appeared distinct from solvent peaks were also reported. LC–MS/MS spectra were acquired at wavelengths of 220 and 254 nm using the below analytical methods (AM).

Method 1 (AM1): Instrument: Agilent 1100/G1956A. Column: Kinetex EVO C18 30 × 2.1 mm × 5 μm. Run time: 1.5 min. Flow rate: 1.5 mL/min, 50 °C. Solvents: A) 0.0375% TFA in H_2_O (v/v); B) 0.01875% TFA in acetonitrile (v/v). Gradient: 5–95% B with A, 0.8 min; hold at 95% B to 1.2 min; 5% B from 1.21 to 1.5 min.

Method 2 (AM2): Instrument: Agilent 1200/G6110A. Column: Kinetex EVO C18 30 × 2.1 mm × 5 μm. Run time: 1.5 min. Flow rate: 1.5 mL/min, 50 °C. Solvents: A) 0.0375% TFA in H_2_O (v/v); B) 0.01875% TFA in acetonitrile (v/v). Gradient: 5–95% B with A, 0.8 min; hold at 95% B to 1.2 min; 5% B from 1.21 to 1.5 min.

Method 3 (AM3): Instrument: Shimadzu LCMS-2020. Column: Kinetex EVO C18 30 × 2.1 mm × 5 μm. Run time: 1.55 min. Flow rate: 1.5 mL/min, 50 °C. Solvents: A) 0.0375% TFA in H_2_O (v/v); B) 0.01875% TFA in acetonitrile (v/v). Gradient: 5–95% B with A, 0.8 min; hold at 95% B to 1.2 min; 5% B from 1.21 to 1.55 min.

Method 4 (AM4): Instrument: Agilent 1200 LC/G1956A MSD. Column: Kinetex EVO C18 30 × 2.1 mm × 5 μm. Run time: 1.5 min. Flow rate: 1.5 mL/min, 50 °C. Solvents: A) 0.0375% TFA in H_2_O (v/v); B) 0.01875% TFA in acetonitrile (v/v). Gradient: 5–95% B with A, 0.8 min; hold at 95% B to 1.2 min; 5% B from 1.21 to 1.5 min.

Method 5 (AM5): Instrument: Shimadzu LCMS-2020. Column: Kinetex EVO C18 30 × 2.1 mm × 5 μm. Run time: 1.55 min. Flow rate: 1.5 mL/min, 50 °C. Solvents: A) 0.0375% TFA in H_2_O (v/v); B) 0.01875% TFA in acetonitrile (v/v). Gradient: 0–60% B with A, 0.8 min; hold at 60% B to 1.2 min; 0% B from 1.21 to 1.55 min.

Method 6 (AM6): Instrument: Shimadzu LCMS-2020. Column: Kinetex EVO C18 30 × 2.1 mm × 5 μm. Run time: 4.0 min. Flow rate: 1.5 mL/min, 50 °C. Solvents: A) 0.0375% TFA in H_2_O (v/v); B) 0.01875% TFA in acetonitrile (v/v). Gradient: 5–95% B with A, 3.6 min; hold at 95% B to 3.7 min; 5% B from 3.71 to 4.0 min.

Method 7 (AM7): Instrument: Shimadzu LCMS-2020. Column: Kinetex EVO C18 30 × 2.1 mm × 5 μm. Run time: 1.5 min. Flow rate: 1.5 mL/min, 40 °C. Solvents: A) 0.025% NH_3_ in H_2_O (v/v); B) acetonitrile. Gradient: 5–95% B with A, 0.8 min; hold at 95% B to 1.2 min; 5% B from 1.21 to 1.5 min.

The purity of all test compounds was ≥ 95%.

Literature compounds **1**, **4** and **6** were prepared according to published procedures.^8,14,29^

#### *tert*-Butyl (4-(Allyloxy)­butyl)­carbamate

To a solution of NaOH (2.11 g, 52.8 mmol) in 1,4-dioxane (176 mL), was added tert-butyl *N*-(4-hydroxybutyl)­carbamate (10.0 g, 52.8 mmol) and 3-bromoprop-1-ene (12.8 g, 105 mmol) at room temperature. The mixture was heated to 70 °C and stirred for 12 h. The mixture was diluted with water (100 mL) and extracted with ethyl acetate (100 mL × 3). The combined organic layers were washed with brine (80 mL × 2), dried (Na_2_SO_4_), filtered and concentrated *in vacuo*. The residue was purified by column chromatography on silica gel eluting with petroleum ether/ethyl acetate (5:1) to afford the title compound (5.5 g, 45% yield) as a light-yellow oil.

^1^H NMR (400 MHz, CHCl_3_-*d*) δ: 5.93–5.82 (m, 1H), 5.27–5.20 (m, 1H), 5.16–5.11 (m, 1H), 4.70 (br, s, 1H), 3.93–3.91 (m, 2H), 3.43–3.39 (t, 2H), 3.12–3.08 (m, 2H), 1.62–1.49 (m, 4H), 1.40 (s, 9H).

#### *tert*-Butyl (4-(2-Hydroxyethoxy)­butyl)­carbamate (**59**)

Ozone was bubbled through a solution of tert-butyl (4-(allyloxy)­butyl)­carbamate (5.50 g, 24.0 mmol) in DCM (50 mL) at −78 °C until the mixture turned blue, then the reaction mixture was warmed to 0 °C and NaBH_4_ (1.77 g, 46.8 mmol) was added slowly. The reaction mixture was warmed to room temperature and stirred for 12 h. The reaction was quenched with water (50 mL) and extracted with DCM (80 mL × 2). The combined organic layers were washed with brine (80 mL × 2), dried over (Na_2_SO_4_), filtered and concentrated *in vacuo*. The residue was purified by column chromatography on silica gel eluting with petroleum ether/ethyl acetate (2:1) to afford title compound **59** (2.65 g, 47% yield) as a colorless oil.

^1^H NMR (400 MHz, CHCl_3_-*d*) δ: 4.78 (br s, 1H), 3.72–3.71 (m, 2H), 3.53–3.51 (t, 2H), 3.51–3.46 (t, 2H), 3.13–3.12 (m, 2H), 2.41 (br s, 1H), 1.66–1.50 (m, 4H), 1.42 (s, 9H).

#### 5-(2-(4-((*tert*-Butoxycarbonyl)­amino)­butoxy)­ethoxy)­benzo­[*c*]­[2,6]­naphthyridine-8-carboxylic acid

To a mixture of alcohol **59** (428 mg, 1.83 mmol) in DMF (10 mL) was added NaH (110 mg, 2.75 mmol, 60% w/w dispersion in oil) in one portion followed by methyl 5-chlorobenzo­[*c*]­2,6-naphthyridine-8 carboxylate^14^ (500 mg, 1.83 mmol) under a nitrogen atmosphere at 0 °C. The mixture was heated to 80 °C and stirred for 12 h. The mixture was diluted with water (50 mL) and extracted with ethyl acetate (50 mL × 2). The combined organic phases were washed with brine (50 mL), dried (Na_2_SO_4_) and concentrated *in vacuo*. The residue was purified by reverse-phase HPLC (column: Phenomenex Luna C18 250 × 50 mm × 10 μm; eluents: A) 0.1% TFA in H_2_O (v/v), B) acetonitrile; gradient: 22–42% B, 10 min) to afford the title compound (300 mg, 35% yield) as a light-yellow solid.

**LC–MS (AM3)**: rt = 0.903 min, (456.3 [M + H]^+^).

#### 5-(2-(4-Aminobutoxy)­ethoxy)­benzo­[*c*]­[2,6]­naphthyridine-8-carboxylic acid (**60**)

To a solution of 5-(2-(4-((*tert*-butoxycarbonyl)­amino)­butoxy)­ethoxy)­benzo­[*c*]­[2,6]­naphthyridine-8-carboxylic acid (100 mg, 220 mmol) in DCM (5 mL) was added TFA (1.00 mL, 13.5 mmol) and the mixture was stirred at room temperature for 0.5 h. The mixture was concentrated *in vacuo* to afford title compound **60** (100 mg, 97% yield, TFA salt) as a brown solid, which was used in the next step without purification.

### General Procedure for the Synthesis of **61a**–**J**

To a solution of amine **60** (1 equiv) and substituted benzaldehyde (1 equiv) in methanol (3–50 mL) was added DIPEA (2–3 equiv) at room temperature and the mixture stirred for 1 h. NaBH­(OAc)_3_ (1–5 equiv) was added and the mixture was stirred at room temperature for 11 h. The reaction mixture was concentrated *in vacuo* and the residue was purified by the method indicated to afford the title compound.

#### 5-(2-(4-((3-Chloro-4-(trifluoromethoxy)­benzyl)­amino)­butoxy)­ethoxy)­benzo­[*c*]­[2,6]­naphthyridine-8-carboxylic acid (**61a**)

**Aldehyde:** 3-Chloro-4-(trifluoromethoxy)­benzaldehyde.

Product purified by reverse-phase HPLC (column: Phenomenex Synergi C18 150 × 25 mm × 10 μm; eluents: A) 0.225% FA in H_2_O (v/v), B) acetonitrile; gradient: 28–58% B, 8.5 min) to afford title compound **61a** (29 mg, 24% yield) as a brown solid.

**LC–MS (AM3):** rt = 0.857 min, (564.2 [M + H]^+^).

^1^H NMR (400 MHz, MeOH-*d*_*4*_) δ: 9.88 (s, 1H), 8.76 (d, *J* = 5.6 Hz, 1H), 8.57 (d, *J* = 8.4 Hz, 1H), 8.37 (s, 1H), 8.11–8.05 (m, 2H), 7.71 (d, *J* = 1.6 Hz, 1H), 7.51–7.45 (m, 2H), 4.76 (t, *J* = 4.8 Hz, 2H), 4.16 (s, 2H), 3.95 (t, *J* = 4.8 Hz, 2H), 3.68 (t, *J* = 6.0 Hz, 2H), 3.08 (t, *J* = 7.8 Hz, 2H), 1.89–1.81 (quin, 2H), 1.74–1.67 (quin, 2H).

#### 5-(2-(4-((3-Fluoro-4-(trifluoromethoxy)­benzyl)­amino)­butoxy)­ethoxy)­benzo­[*c*]­[2,6]­naphthyridine-8-carboxylic acid (**61b**)

**Aldehyde:** 3-Fluoro-4-(trifluoromethoxy)­benzaldehyde

Product purified by reverse-phase HPLC (column: Welch Xtimate C18 150 × 30 mm × 5 μm; eluents: A) 0.05% NH_4_OH in H_2_O (v/v), B) acetonitrile; gradient: 14–44% B, 11.5 min) to afford title compound **61b** (123 mg, 31% yield) as an off-white solid.

**LC–MS (AM7):** rt = 0.753 min, (548.3 [M + H]^+^).

^1^H NMR (400 MHz, MeOH-*d*_*4*_) δ: 9.69 (s, 1H), 8.65 (d, *J* = 5.6 Hz, 1H), 8.39 (d, *J* = 8.4 Hz, 1H), 8.28 (d, *J* = 1.2 Hz, 1H), 8.02 (dd, *J* = 8.4, 1.6 Hz, 1H), 7.87 (d, *J* = 5.6 Hz, 1H), 7.52 (dd, *J* = 10.8, 2.0 Hz, 1H), 7.46–7.37 (m, 2H), 4.64 (t, *J* = 4.8 Hz, 2H), 4.16 (s, 2H), 3.89 (t, *J* = 4.8 Hz, 2H), 3.66 (t, *J* = 6.0 Hz, 2H), 3.05 (t, *J* = 7.8 Hz, 2H), 1.90–1.83 (quin, 2H), 1.74–1.67 (quin, 2H).

#### 5-(2-(4-((3-Methyl-4-(trifluoromethoxy)­benzyl)­amino)­butoxy)­ethoxy)­benzo­[*c*]­[2,6]­naphthyridine-8-carboxylic acid (**61c**)

**Aldehyde:** 3-Methyl-4-(trifluoromethoxy)­benzaldehyde.

Product purified by reverse-phase HPLC (column: Waters Xbridge 150 × 25 mm × 5 μm; eluents: A) 0.05% NH_4_OH in H_2_O, B) acetonitrile; gradient: 18–48% B, 9 min) to afford title compound **61c** (39 mg, 14% yield) as a yellow solid.

**LC-MS (AM7):**rt = 0.763 min, (544.3 [M + H]^+^).

^1^H NMR (400 MHz, MeOH-*d*_*4*_) δ: 9.73 (s, 1H), 8.66 (d, *J* = 5.6 Hz, 1H), 8.41 (d, *J* = 8.4 Hz, 1H), 8.30 (br s, 1H), 8.03 (d, *J* = 8.4 Hz, 1H), 7.90 (d, *J* = 5.6 Hz, 1H), 7.42 (d, *J* = 2.0 Hz, 1H), 7.36 (dd, *J* = 8.4, 2.0 Hz, 1H), 7.24 (dd, *J* = 8.4, 1.2 Hz, 1H), 4.66 (t, *J* = 4.8 Hz, 2H), 4.09 (s, 2H), 3.89 (t, *J* = 4.8 Hz, 2H), 3.65 (t, *J* = 6.0 Hz, 2H), 3.04 (t, *J* = 7.6 Hz, 2H), 2.27 (s, 3H), 1.89–1.81 (quin, 2H), 1.73–1.64 (quin, 2H).

#### 5-(2-(4-((3-Methoxy-4-(trifluoromethoxy)­benzyl)­amino)­butoxy)­ethoxy)­benzo­[*c*]­[2,6]­naphthyridine-8-carboxylic acid (**61d**)

**Aldehyde:** 3-Methoxy-4-(trifluoromethoxy)­benzaldehyde.

Product purified by reverse-phase HPLC (column: Phenomenex Gemini-NX C18 75 × 30 mm × 3 μm; eluents: A) 0.05% NH_4_OH in H_2_O (v/v), B) acetonitrile; gradient: 10–40% B, 7 min) to afford title compound **61d** (37 mg, 13% yield) as a yellow gum.

**LC–MS (AM3):** rt = 0.829 min, (560.1 [M + H]^+^).

^1^H NMR (400 MHz, MeOH-*d*_*4*_) δ: 9.73 (s, 1H), 8.67 (d, *J* = 5.6 Hz, 1H), 8.41 (d, *J* = 8.4 Hz, 1H), 8.31 (d, *J* = 1.6 Hz, 1H), 8.04 (dd, *J* = 8.4, 1.6 Hz, 1H), 7.91 (d, *J* = 5.6 Hz, 1H), 7.30 (d, *J* = 2.0 Hz, 1H), 7.24 (dd, *J* = 8.4, 1.2 Hz, 1H), 7.03 (dd, *J* = 8.4, 2.0 Hz, 1H), 4.67 (t, *J* = 4.8 Hz, 2H), 4.08 (s, 2H), 3.90 (t, *J* = 4.8 Hz, 2H), 3.86 (s, 3H), 3.66 (t, *J* = 6.0 Hz, 2H), 3.01 (t, *J* = 7.6 Hz, 2H), 1.88–1.81 (m, 2H), 1.74–1.67 (m, 2H).

#### 5-(2-(4-((3-Cyclopropyl-4-(trifluoromethoxy)­benzyl)­amino)­butoxy)­ethoxy)­benzo­[*c*]­[2,6]­naphthyridine-8-carboxylic acid (**61e**)

**Aldehyde:** 3-Cyclopropyl-4-(trifluoromethoxy)­benzaldehyde.^38^

Product purified by reverse-phase HPLC (column: Waters Xbridge 150 × 25 mm × 5 μm; eluents: A) 0.05% NH_4_OH in H_2_O, B) acetonitrile; gradient: 21–51% B, 9 min) to afford title compound **61e** (42.6 mg, 15% yield) as a yellow solid.

**LC–MS (AM7):** rt = 0.777 min, (570.3 [M + H]^+^).

^1^H NMR (400 MHz, MeOH-*d*_*4*_) δ: 9.82 (s, 1H), 8.71 (d, *J* = 5.6 Hz, 1H), 8.50 (d, *J* = 8.4 Hz, 1H), 8.36 (s, 1H), 8.08 (d, *J* = 8.4 Hz, 1H), 8.01 (d, *J* = 5.6 Hz, 1H), 7.31–7.24 (m, 2H), 7.12 (s, 1H), 4.73 (t, *J* = 4.8 Hz, 2H), 4.08 (s, 2H), 3.92 (t, *J* = 4.8 Hz, 2H), 3.67 (t, *J* = 6.0 Hz, 2H), 3.03 (t, *J* = 7.6 Hz, 2H), 2.13–2.06 (m, 1H), 1.87–1.80 (quin, 2H), 1.72–1.65 (quin, 2H), 1.03–0.98 (m, 2H), 0.75–0.71 (m, 2H).

#### 5-(2-(4-((3,5-Difluoro-4-(trifluoromethoxy)­benzyl)­amino)­butoxy)­ethoxy)­benzo­[*c*]­[2,6]­naphthyridine-8-carboxylic acid (**61f**)

**Aldehyde:** 3,5-Difluoro-4-(trifluoromethoxy)­benzaldehyde.

Product purified by reverse-phase HPLC (column: Phenomenex Luna C18 150 × 40 mm × 5 μm; eluents: A) 0.1% formic acid in water, B) acetonitrile; gradient: 40–65%, 15 min). The eluant was adjusted to pH 7–8 by the addition of ammonium hydroxide (25 wt %) and concentrated in vacuo to enable precipitation. The precipitate was collected and dried to afford the title compound as a white solid (25 g, 14% yield).

**LC–MS (AM7):** rt = 0.769 min, (566.3 [M + H]^+^).

HPLC trace of **61f** is shown in Figure S1.

^1^H NMR (400 MHz, MeOH-*d*_4_) δ: 9.69 (s, 1H), 8.65 (d, *J* = 5.6 Hz, 1H), 8.37 (d, *J* = 8.4 Hz, 1H), 8.27 (d, *J* = 1.6 Hz, 1H), 8.01 (dd, *J* = 8.4, 1.6 Hz, 1H), 7.87 (d, *J* = 5.2 Hz, 1H), 7.38 (d, *J* = 8.4 Hz, 2H), 4.64 (t, *J* = 4.8 Hz, 2H), 4.16 (s, 2H), 3.90 (t, *J* = 4.8 Hz, 2H), 3.68 (t, *J* = 6.0 Hz, 2H), 3.04 (t, *J* = 7.6 Hz, 2H), 1.91–1.84 (quin, 2H), 1.76–1.69 (quin, 2H).

#### 5-(2-(4-((4-Chloro-3-(trifluoromethoxy)­benzyl)­amino)­butoxy)­ethoxy)­benzo­[*c*]­[2,6]­naphthyridine-8-carboxylic Acid (**61g**)

**Aldehyde:** 4-Chloro-3-(trifluoromethoxy)­benzaldehyde.

Product purified by reverse-phase HPLC (column: Waters Xbridge 150 × 50 mm × 10 μm; eluents: A) 10 mM NH_4_HCO_3_ in H_2_O, B) acetonitrile; gradient: 20–50% B, 11 min) to afford title compound **61g** (104 mg, 36% yield) as a yellow solid.

**LC–MS (AM7):** rt = 0.768 min, (564.2 [M + H]^+^).

^1^H NMR (400 MHz, MeOH-*d*_4_) δ: 9.75 (s, 1H), 8.69 (d, *J* = 5.6 Hz, 1H), 8.45 (d, *J* = 8.4 Hz, 1H), 8.31 (d, *J* = 1.6 Hz, 1H), 8.04 (dd, *J* = 8.4, 1.6 Hz, 1H), 7.92 (d, *J* = 5.2 Hz, 1H), 7.60–7.58 (m, 2H), 7.47 (dd, *J* = 8.4, 2.0 Hz, 1H), 4.68 (t, *J* = 4.8 Hz, 2H), 4.15 (s, 2H), 3.91 (t, *J* = 4.8 Hz, 2H), 3.66 (t, *J* = 6.0 Hz, 2H), 3.04 (t, *J* = 7.8 Hz, 2H), 1.89–1.82 (quin, 2H), 1.74–1.67 (quin, 2H).

#### 5-(2-(4-((3-Chloro-5-(trifluoromethoxy)­benzyl)­amino)­butoxy)­ethoxy)­benzo­[*c*]­[2,6]­naphthyridine-8-carboxylic Acid (**61h**)

**Aldehyde:** 3-Chloro-5-(trifluoromethoxy)­benzaldehyde.

Product purified by reverse-phase HPLC (column: Waters Xbridge 150 × 50 mm × 10 μm; eluents: A) 10 mM NH_4_HCO_3_ in H_2_O, B) acetonitrile; gradient: 22–52% B, 11 min) to afford title compound **61h** (96 mg, 33% yield) as a light-yellow solid.

**LC–MS (AM7):** rt = 0.767 min, (564.2 [M + H]^+^).

^1^H NMR (400 MHz, MeOH-*d*_*4*_) δ: 9.79 (s, 1H), 8.71 (d, *J* = 5.2 Hz, 1H), 8.47 (d, *J* = 8.4 Hz, 1H), 8.34 (s, 1H), 8.06 (d, *J* = 8.4 Hz, 1H), 7.98 (d, *J* = 4.4 Hz, 1H), 7.54 (s, 1H), 7.40–7.33 (m, 2H), 4.70 (t, *J* = 4.8 Hz, 2H), 4.14 (s, 2H), 3.92 (t, *J* = 4.4 Hz, 2H), 3.67 (t, *J* = 5.6 Hz, 2H), 3.03 (t, *J* = 7.8 Hz, 2H), 1.88–1.81 (m, 2H), 1.74–1.66 (m, 2H).

#### 5-(2-(4-((3-Cyclopropyl-5-(trifluoromethoxy)­benzyl)­amino)­butoxy)­ethoxy)­benzo­[*c*]­[2,6]­naphthyridine-8-carboxylic Acid (**61i**)

**Aldehyde:** 3-Cyclopropyl-5-(trifluoromethoxy)­benzaldehyde.^38^

Product purified by reverse-phase HPLC (column: Waters Xbridge 150 × 50 mm × 10 μm; eluents: A) 10 mM NH_4_HCO_3_ in H_2_O, B) acetonitrile; gradient: 22–52% B, 11 min) to afford title compound **61i** (98 mg, 33% yield) as a yellow solid.

**LC–MS (AM7):** rt = 0.785 min, (570.3 [M + H]^+^).

^1^H NMR (400 MHz, MeOH-*d*_*4*_) δ: 9.82 (s, 1H), 8.71 (d, *J* = 5.2 Hz, 1H), 8.50 (d, *J* = 8.4 Hz, 1H), 8.36 (d, *J* = 1.6 Hz, 1H), 8.08 (dd, *J* = 8.4, 1.6 Hz, 1H), 8.00 (d, *J* = 5.2 Hz, 1H), 7.16 (s, 2H), 6.98 (s, 1H), 4.73 (t, *J* = 4.8 Hz, 2H), 4.09 (s, 2H), 3.93 (t, *J* = 4.8 Hz, 2H), 3.67 (t, *J* = 6.0 Hz, 2H), 3.03 (t, *J* = 7.2 Hz, 2H), 1.96–1.91 (m, 1H), 1.89–1.81 (quin, 2H), 1.74–1.66 (quin, 2H), 1.04–0.98 (m, 2H), 0.74–0.69 (m, 2H).

#### 5-(2-(4-((3-(Cyanomethyl)-5-(trifluoromethoxy)­benzyl)­amino)­butoxy)­ethoxy)­benzo­[*c*]­[2,6]­naphthyridine-8-carboxylic Acid (**61j**)

**Aldehyde:** 2-(3-Formyl-5-(trifluoromethoxy)­phenyl)­acetonitrile.^38^

Product purified by reverse-phase HPLC (column: Waters Xbridge 150 × 50 mm × 10 μm; eluents: A) 10 mM NH_4_HCO_3_ in H_2_O, B) acetonitrile; gradient: 16–46% B, 11 min) to afford title compound **61j** (651 mg, 26% yield) as a yellow gum.

**LC–MS (AM7):** rt = 0.751 min, (569.3 [M + H]^+^).

^1^H NMR (400 MHz, DMSO-*d*_*6*_) δ: 10.11 (s, 1H), 8.89 (d, *J* = 5.2 Hz, 1H), 8.82 (d, *J* = 8.4 Hz, 1H), 8.32 (s, 1H), 8.08–8.02 (m, 2H), 7.38–7.33 (m, 2H), 7.21 (s, 1H), 4.70 (t, *J* = 4.4 Hz, 2H), 4.09 (s, 2H), 3.87 (t, *J* = 4.0 Hz, 2H), 3.79 (s, 2H), 3.53 (t, *J* = 6.0 Hz, 2H), 2.56 (t, *J* = 6.0 Hz, 2H), 1.62–1.48 (m, 4H).

### Computational PDB Mining

All X-ray structures in the Protein Data Bank (PDB) were processed using a proprietary workflow to generate a database of nonredundant protein sequences. This database was searched with BLAST^50^ to identify all PDB entries with sequence similarity to CK2α. All resultant PDB structures were overlaid onto the CAM4066 (**6**) structure (PDB: 5CU4) by rms fitting of the equivalent backbone atoms from all residues in the sequence alignment that were within 4 Å of the binding cavity containing CAM4066. The overlap was carried out using an iterative procedure in which the corresponding atoms with the worst fit were successively removed from the alignment and the superposition recalculated until the largest distance between superimposed atoms was no more than 1.5 Å. This approach prevented outlier residues from causing translational shifts in the overlap. The resultant rotation matrices were applied to the ligands from the PDB files, and any non-ATP ligands occupying the binding pocket were compared with CAM4066 to identify any bonds that overlapped, using a 0.5 Å rms threshold. Any compounds meeting these criteria and also binding to the hinge region were manually inspected to ascertain the potential for hybridizing with CAM4066. This inspection focused on assessing the quality of fit of the overlapping bonds and whether or not the hybrid product (or an idea that it inspired, e.g., heteroatom switches) was novel, synthetically tractable and looked like it could fit sterically into the CK2α binding pocket.

### Biology

#### ADP-Glo^TM^ Kinase Activity Assay

Purified recombinant wild-type CK2 (residues 2–329) was prepared as described previously.^29^ Commercial sources of recombinant purified kinase were used for the selectivity assays: CLK2 (Carna Biosciences 04127); DAPK3 (Carna Biosciences 02–136); HIPK3 (Carna Biosciences 04–137); DYRK2 (Carna Biosciences 04–132). The assay condition for each of the kinases is summarized in Table 10.

**Table 10:** Assay Condition for Each Kinase

Assay	Enzyme Concentration (nM)	Substrate	Substrate Concentration (μM)	Apparent ATP *K*_M_ (μM)	ATP Concentration (μM)
CK2α	0.2	RRRADDSDDDDD	50	15	15
CLK2	20	S6K Substrate	50	180	100
DAPK3	4.0	ZIPtide	50	2.7	2.5
HIPK3	7.5	DYRKtide-F	50	8.2	5
DYRK2	1.3	DYRKtide-F	50	20	15

Enzyme/substrate mixture (1.67×) was prepared in reaction buffer (40 mM Tris, 200 mM NaCl, 20 mM MgCl_2_, 0.1 mg/mL BSA, pH 7.5) and preincubated at room temperature for 30 min. 50 nL per well of test compound or control solution (DMSO for 0% control or 1 mM of silmitasertib for 100% control) was dispensed into a white opaque 384-well assay plate as required. Test compounds were screened over an 11-point concentration range. 3 μL per well of preincubated enzyme/substrate mixture was dispensed into the assay plate, centrifuged, mixed and incubated at room temperature for 15 min. 2 μL of ATP solution (2×) in reaction buffer was dispensed into the assay plate. The plate was centrifuged, mixed, sealed and incubated at room temperature for 2 h. Five μL of ADP-Glo^TM^ reagent per well was added and the plate incubated at room temperature for 1 h. 10 μL of kinase detection reagent per well was added and the assay plate incubated at room temperature for 30 min. The plate was read for luminescence. The percent inhibition for each test well was calculated using DMSO control wells to define 0% inhibition and 1 mM silmitasertib wells to define 100% inhibition. IC_50_ curves were generated from the 11-point concentration response using the standard 4-parameter fit method (Model 205, XL-fit) and IC_50_ values calculated. *K*_i_ values for key compounds were also calculated in GraphPad Prism using the equation for Morrison *K*_i_ (tight inhibition) for CK2α and standard *K*_i_ equation for all selectivity targets.

#### Cellular Inhibition of CK2α in HCT116 Using NanoBRET

The NanoBRET assay was performed according to the manufacturer’s instructions (Promega). Briefly, HCT116 (ECACC 91091005) cells were trypsinized from routine subculture and centrifuged at 800 rpm for 5 min. The cell pellet was resuspended in OptiMEM and cell density adjusted to 2 × 10^5^ cells/mL. DNA complexes were prepared (3.7 μL CSNK2A2-NanoLucfusion DNA, 42 μL FuGENE HD Transfection Reagent, 1396.22 μL OptiMEM) and added to the HCT116 cell suspension at a ratio of 1:20. The cell and DNA mixture was incubated overnight in a humidified, 37 °C, 5% CO_2_ incubator. 30 μL HCT116/DNA complex cell suspension, 5 μL of 8× tracer solution (40 μL NanoBRET Tracer K-5, 160 μL DMSO, 800 μL tracer dilution buffer, 1.5 mL OptiMEM) and 5 μL of test compound (at 8× final test concentration) or control solution (DMSO for 0% control, 1 mM silmitasertib for 100% control) was transferred into each well of a white opaque 384-well assay plate and incubated in a humidified, 37 °C, 5% CO_2_ incubator for 2 h. 20 μL of 3× complete substrate plus inhibitor solution (60 μL NanoBRET Nano-Glo substrate, 20 μL extracellular NanoLuc inhibitor, 9.92 mL OptiMEM) was dispensed per well into the assay plate. The assay plate was read at a donor emission wavelength of 450 nm and acceptor emission wavelength of 610 or 630 nm. The percent inhibition for each test well was calculated using DMSO control wells to define 0% inhibition and 1 mM silmitasertib wells to define 100% inhibition. IC_50_ values were calculated using the standard 4-parameter fit method (Model 205, XL-fit).

#### HCT116 Cell Proliferation Assay

The procedures were performed according to the CellTiter-Glo Luminescent Cell Viability Assay Kit (Promega). Briefly, HCT116 cells were trypsinized from routine subculture and centrifuged at 800 rpm for 5 min. Cells were resuspended, counted and adjusted to an appropriate cell density. Cell suspension (90 μL) was added into each well of a 96-well black flat-bottom plate assay plate and incubated overnight in a humidified incubator at 37 °C, 5% CO_2_. Ten μL per well of test compound (at 10× final test concentration) or control solution (DMSO for 0% control, 1 mM silmitasertib for 100% control) was dispensed into the assay plate and incubated for 72 h. CellTiter-Glo Reagent (50 μL) was added to each well and the plate covered to protect from light. The wells were gently mixed to induce cell lysis then incubated at room temperature for 10 min before reading the luminescence signal. The percent inhibition for each test well was calculated using DMSO control wells to define 0% inhibition and 1 mM silmitasertib wells to define 100% inhibition. IC_50_ values were calculated using the standard 4-parameter fit method (Model 205, XL-fit).

#### p-AKT S129 Western Blot Assay

Cell suspension (either HCT116 cell line or human PBMC isolated from healthy donors) was plated into 100 mm well dishes in 9.5 mL cell culture media. The dishes were incubated at 37 °C, 5% CO_2_ overnight. 500 μL per well of test compound (at 20× final test concentration) or control solution (DMSO for 0% control, 1 mM silmitasertib for 100% control) was transferred onto the plated cells and incubated at 37 °C for 6 h. The medium from the 100 mm dishes was removed and cells washed with 3 mL of ice-cold 1× phosphate buffered saline (PBS). PBS was removed and 600 μL ice-cold 1× RIPA Buffer (containing 1% protease inhibitor cocktail and 1% phosphatase inhibitor cocktail 2) was added into each dish. The cells were collected into 1.5 mL microfuge tubes and incubated on ice for 30 min before centrifugation at 14,000 rpm for 10 min. Protein concentration in the supernatant was measured using a Pierce BCA Protein Assay Kit following the manufacturer’s instructions. All samples were diluted to the same final concentration using RIPA lysis and extraction buffer (Thermo Scientific) plus Pierce LDS Sample Buffer and 10× NuPAGE Sample Reducing Agent. The samples were heated at 100 °C for 10 min. Ten μL of protein sample was loaded into each well of a NuPAGE Novex 4–12% Bis-Tris gel and electrophoresis conducted with MES running buffer. Protein was transferred to nitrocellulose membranes using an iBlot2 Gel Transfer Device and membranes washed with 10 mL 1× tris buffered saline (TBS) for 5 min. Blocking was performed with 10 mL Intercept (TBS) Blocking Buffer at room temperature for 1 h. Following two wash steps with 10 mL 1× TBS, the membrane was incubated with 10 mL primary antibody (1:1000 anti-AKT pS129 antibody (Abcam ab133458); 1:1,000 anti-AKT (CST-9272); 1:2,000 anti-GAPDH (CST-5174)) diluted in Intercept TBS Blocking Buffer containing 0.1% Tween 20, at 4 °C overnight. Following three wash steps with 10 mL 1× TBS, the membrane was incubated with 10 mL of secondary antibody (IRDye 800CW Goat anti-Rabbit IgG Secondary Antibody, LICOR) diluted 1:20,000 in Intercept TBS Blocking Buffer containing 0.1% Tween 20, for 1 h at room temperature, protected from light. The membrane was washed three times with 10 mL 1× TBS before reading the fluorescence signal (Odyssey CLx Imaging System) and quantification of individual band intensity using Image Studio (NIR) software. Protein level in each sample was normalized to a reference protein (GAPDH) and the ratio of p-AKT S129:AKT determined for each sample. The percent inhibition for each test sample was calculated using DMSO control samples to define 0% inhibition and 1 mM silmitasertib samples to define 100% inhibition. IC_50_ values were calculated using the standard 4-parameter fit method (GraphPad Prism).

#### *In Vivo* HCT116 Tumor Xenograft Study

Mice (BALB/c nude mice, female, 6–8 weeks’ old, weighing approximately 20 g) were inoculated subcutaneously in the right flank with HCT116 cells (5 × 10^6^) in 0.1 mL of PBS. When the average tumor size reached approximately 200 mm^3^, mice were assigned into groups using randomized block design based upon their tumor sizes and body weights.

For chronic tumor growth inhibition studies, *n* = 10 mice were treated by once-daily oral gavage administration of either vehicle only (10% DMSO/90% (20%HP-β-CD in water)) or **61f** in vehicle (group 1 = vehicle only; group 2 = 10 mg/kg; group 3 = 30 mg/kg; group 4 = 100 mg/kg) for 21 days. Following the final administration, *n* = 5 mice in each group were sacrificed at 2 and 8 h postdosing and samples taken for assessment of plasma PK, tumor PK and quantification of tumor p-AKT S129 by Western blotting.

#### Broad Kinase Inhibition Screening

Broad kinase inhibition of **61f** was assessed using the KINOME*scan* scanMAX panel (Eurofins). An initial screen was performed at a single concentration of 100 nM. Concentration-response was then performed on selected kinases using the same assay format to determine *K*_d_ values.

#### Surface Plasmon Resonance (SPR) Binding Assay

Double His-tagged CK2α kinase domain (DH_CK2) was expressed and purified as described previously.^29^ SPR was performed on BiaCore T200 instrument using the analyte binding protocol. Test compound was applied with each concentration tested in replicate. Baseline correction and solvent correction were applied to each binding cycle based on signal from the reference channel (NiNTA chip surface loaded with metal ion but lacking CK2α protein). Data was fitted and processed on the Biacore Evaluation Software using nonlinear regression analysis to fit a 1:1 binding model and determine *K*_d_, *K*_on_, *K*_off_ and *R*_max_.

#### X-ray Crystallography

CK2α_KA mutant was expressed and purified as described previously.^29^ Test compound was soaked into CK2α_KA crystals at 1 mM concentration for 1 h in 107 mM MES pH 6.5, 35% glycerol ethoxylate and 1.04 M ammonium acetate, after which the crystals were cryo-cooled in liquid nitrogen for data collection. X-ray diffraction data was collected at Diamond Light Source beamline i04 at wavelength 0.9795 Å. Data were integrated and scaled using the pipedream package (Global Phasing Ltd.); structures were solved by molecular replacement using Phaser crystallographic software.^51^ The structural model was iteratively refined and rebuilt by using AutoBuster (Global Phasing Ltd.) and Coot programs,^52^ respectively. Ligand coordinates and restraints were generated from the SMILES string using the grade software package (Global Phasing Ltd.). Electron densities for all the compounds are shown in Figure S3. All coordinates have been deposited to the Protein Data Bank. Data collection and refinement statistics are shown in Table S2.

### *In Vitro* and *In Vivo* Pharmacokinetics

#### LogD Determination

Measurement of octanol–water partition coefficient (logD) was performed using a miniaturized 1-octanol/buffer (10 mM sodium phosphate pH 7.4) shake-flask method followed by liquid chromatography coupled to tandem mass spectrometry (LC–MS/MS) analysis. Test compound (2 μL of 10 mM in DMSO) was transferred to 96-well polypropylene cluster tubes. Buffer-saturated 1-octanol (150 μL/well) and 1-octanol saturated buffer (150 μL/well) were added to the tubes, respectively. The tubes were vigorously shaken on their sides for 1 min and then shaken at 600 rpm for 1 h. The tubes were centrifuged at 4,000 rpm for 10 min. The buffer-layer sample and 1-octanol-layer samples were diluted with internal standard solution. The concentration of test compound in the octanol and aqueous layers was determined by measuring the integrated sample peak area using LC–MS/MS and logD calculated using the equation: logD = log­[(concentration in octanol/volume of octanol)/(concentration in buffer/volume of buffer)].

#### Determination of Intrinsic Clearance in Liver Microsomes

The following assay method was used for incubations with mouse, rat, dog (Xenotech) and human (Corning) liver microsomes. Five μL test compound (1 μM final concentration) was incubated with 445 μL isolated liver microsomes (0.56 mg protein/mL in 100 mM potassium phosphate buffer (pH 7.4)) and either 50 μL 100 mM potassium phosphate buffer or 50 μL reduced form of nicotinamide adenine dinucleotide phosphate (NADPH) cofactor. Plates were incubated at 37 °C for 60 min. At 0, 5, 10, 20, 30, and 60 min, 60 μL of the incubation mix was removed and quenched with 180 μL of solvent containing internal standard. The samples were analyzed by LC–MS/MS, and the intrinsic clearance values were determined as described previously.^53^

#### Determination of Intrinsic Clearance in Hepatocytes

The following assay method was used for incubations with pooled mouse, rat, dog and human hepatocytes (BioreclamationIVT). Test compound was prepared and prewarmed to 37 °C. Cryopreserved hepatocytes were thawed and a cell suspension prepared at 5 × 10^5^ cells/mL with prewarmed (37 °C) Williams Medium E. 198 μL of prewarmed cell suspension was added to the required number of wells of a 96-well plate. 2 μL of 100 μM test compound was transferred into each well of the 96-well plate in duplicate (final test concentration of 1 μM). The incubations were maintained at 37 °C for the duration of the experiment. At set intervals (0, 15, 30, 60, and 90 min) 20 μL of cell suspension was transferred to a 96-well block containing 100 μL of ice-cold acetonitrile containing IS to terminate the reaction. The samples were centrifuged, and the resulting supernatant analyzed by LC–MS/MS. Intrinsic clearance was calculated by nonlinear regression analysis of peak area ratio vs time using the following equation to determine the first order elimination rate constant (*k*_e_).

% remaining at each time point = (PAR of analyte to IS/PAR of analyte to IS at *T*_0_) × 100

*K*_e_ = −gradient of nonlinear regression

*T*_1/2_ (min) = Ln2 (0.693)/*K*_e_

*V* = incubation volume (μL)/number of cells (× 10^6^)

CL_int (hep)_ (μL/min/10^6^ cells) = *V* × Ln(2)/*T*_1/2_

CL_int (liver)_ = CL_int (hep)_ × liver weight (g/kg body weight) × hepatocellularity

To scale the hepatocyte clearance, a hepatocyte yield of 1.35 × 10^8^ (mouse), 1.17 × 10^8^ (rat), 2.15 × 10^8^ (dog) and 1.39 × 10^8^ (human) cells per gram of liver were used.^54^

#### Assessment of Cell Permeability and Transporter-Mediated Efflux

Caco-2 cells were used to assess bidirectional (apical to basolateral (A–B), and vice versa (B–A)) permeability of test compounds in the presence and absence of BCRP inhibitors (novobiocin and sulfasalazine) or P-gp inhibitors (verapamil and zosuquidar). Cells were seeded onto 96-well plate inserts at 1 × 10^5^ cells/cm^2^ and incubated for 21–28 days at 37 °C, 5% CO_2_. Cells were washed with transport buffer (HBSS containing 10 mM HEPES pH 7.4) and incubated with test compound (2, 5, and 20 μM) at 37 °C, 5% CO_2_ for 2 h. After mixing with acetonitrile containing internal standard, all samples were centrifuged at 3200*g* for 10 min. 100 μL test compound supernatant was diluted with 100 μL ultrapure water for LC–MS/MS analysis. Concentrations of test and control compounds in starting solution, donor solution, and receiver solution were quantified by LC–MS/MS, using peak area ratio of analyte/internal standard (IS).

MDR1-MDCKII cells were used to assess bidirectional (A–B and B–A) permeability of test compounds in the presence and absence of P-gp inhibitors (verapamil and zosuquidar). Cells were seeded onto PET in 96-well insert systems at 2.5 × 10^5^ cells/mL for 4–7 days. Test compound was diluted in transport buffer (HBSS with 10 mM HEPES, pH 7.4) to a concentration of 2 μM and applied to the apical or basolateral side of the cell monolayer. The plate was incubated for 2.5 h in 5% CO_2_ at 37 °C. Test and reference compounds were quantified by LC–MS/MS analysis based on the peak area ratio of analyte/IS.

For both assays, the apparent permeability coefficient *P*_app_ (× 10^–6^ cm/s) was calculated using the equation: *P*_app_ = (d*C*_r_/d*t*) × *V*_r_/(*A* × *C*_0_)

Where: d*C*_r_/d*t* is the cumulative concentration of compound in the receiver chamber as a function of time (μM/s); *V*_r_ is the solution volume in the receiver chamber; *A* is the surface area for the transport, *C*_0_ is the initial concentration in the donor chamber.

The efflux ratio was calculated using the equation: Efflux Ratio = *P*_app_(B–A)/*P*_app_(A–B)

Percent recovery was calculated using the equation: % Solution Recovery = 100 × [(*V*_r_ × *C*_r_) + (*V*_d_ × *C*_d_)]/(*V*_d_ × *C*_0_)

Where *V*_d_ is the volume in the donor chambers (0.075 mL on the apical side, 0.25 mL on the basolateral side); *C*_d_ and *C*_r_ are the final concentrations of transport compound in donor and receiver chambers, respectively.

#### *In Vivo* Pharmacokinetic Studies

Female BALB/c mice (7–9 weeks’ old, supplied by LC, China) were used to assess the intravenous (IV) pharmacokinetic profile for **61f**. Three animals were used in a serial bleeding design with blood samples taken at the tail vein up to 24 h. The animals had free access to food and water throughout. The compound was formulated in 10% DMSO and Hydroxypropyl-Beta Cyclodextrin 20% in water (2:98, v/v) at a concentration of 0.6 mg/mL and the dose was filtered. The compound was administered as an intravenous bolus at 5 mL/kg to achieve a target dose of 3 mg/kg.

Female or male Sprague–Dawley (SD) rats (7–9 weeks’ old, supplied by WLTH-BJ, China) were used to assess the IV and oral pharmacokinetic profile of **61f**. For each route, three animals were used in a serial bleeding design with blood samples taken at the tail vein up to 24 h. The animals had free access to food and water throughout. For both routes, the compound was formulated in 10% DMSO and hydroxypropyl-beta cyclodextrin 20% in water (2:98, v/v) at a concentration of 0.6 mg/mL (IV) or 0.3 mg/mL (PO) and the dose was filtered. For the IV route, the compound was administered as an IV bolus at 5 mL/kg to achieve a target dose of 3 mg/kg. For the oral route the compound was administered by oral gavage at 10 mL/kg to achieve a target dose of 3 mg/kg. A formulation optimization study was also performed in male SD rats to investigate the impact of different formulations of **61f** on oral bioavailability.

Male and female Beagle dogs (>6 months’ old, 8–12 kg, Beijing Marshall Biotechnology Ltd., China) were used to assess the IV and oral pharmacokinetic profile of **61f**. For each route, two or three animals were used with blood samples taken up to 24 h. Dogs were fasted overnight prior to each dose administration and fed approximately 4 h after the start of dosing. The dogs had free access to water throughout. For both routes, the compound was formulated in 10% DMSO and hydroxypropyl-beta cyclodextrin 20% in water (2:98, v/v) at a concentration of 0.6 mg/mL (IV) or 0.3 mg/mL (oral) and the dose was filtered. For both the IV and oral gavage routes, the compound was administered at 3 mL/kg to achieve a target dose of 3 mg/kg. Three male Beagle dogs were also used to assess pharmacokinetics of **61f** formulated into capsules. This study was conducted as a crossover design with three animals dosed with a single capsule for each formulation tested with 2 days between dose administrations. At the end of each study the dogs were returned to the colony.

PK parameters were obtained from the blood concentration–time profiles using noncompartmental analysis with Phoenix WinNonlin 6.3 software.

### Animal Welfare Statement

All animal studies were conducted in accordance with the IACUC standard animal procedures along with the IACUC guidelines that are compliant with the Animal Welfare Act, the Guide for the Care and Use of Laboratory Animals. All animals were acclimatized to the test facility and checked for their general health by veterinary staff at the end of acclimation period. Environment controls maintained a temperature range of 18–26 °C and a relative humidity range of 40–70%, with a 12-h light/12-h dark cycle. Water was provided to all animals ad libitum and periodically analyzed for specified microorganisms and environment contaminants. Certified diet was provided ad libitum (rodents) and twice-daily (dogs) and routinely analyzed for specified microorganisms, nutritional components and environmental contaminants with all results being reviewed and assessed by veterinary staff.

## Supplementary Material





## Notes

Table S1: data from initial screening against CK2α Chemistry experimental details and analytical data for syntheses of compounds **9**-**14**, **18**–**26**, **30**–39, **41**–**46**, **48**–**53**, **54a**–**m**, **55a**–**b**, **58a**–**I**; Figure S1: HPLC trace for lead compound **61f**; Figure S2: linker comparison from crystal structures; Figure S3: electron densities of all compounds in crystal structures; Table S2: crystallographic data collection and refinement statistics; Figure S4: Surface Plasmon Resonance kinetic binding data; experimental methods for *in vitro* ADME and hERG assays (PDF)
Molecular formula strings (CSV)

## Abbreviations

ADME: absorption, distribution, metabolism, and excretion
ADP: adenosine diphosphate
ATP: adenosine triphosphate
BCRP: breast cancer resistance protein
BRET: bioluminescence resonance energy transfer
BSEP: bile salt export pump
CCA: cholangiocarcinoma
CK2α: casein kinase 2α
CLK2: cdc2-like kinase 2
CRC: colorectal cancer
CYP: cytochrome P450
DAPK3: death-associated protein kinase 3
DDI: drug–drug interaction
DYRK2: dual specificity tyrosine-phosphorylation-regulated kinase 2
ER: efflux ratio
F: oral bioavailability
FaFg: fraction absorbed × fraction escaping gut-wall elimination
FaSSIF: fasted state simulated intestinal fluid
FeSSIF: fed state simulated intestinal fluid
GLP: good laboratory practice
GMP: good manufacturing practice
HEK: human embryonic kidney
hERG: human ether-a-go-go related gene
HIPK3: homeodomain interacting protein kinase 3
HLM: human liver microsomes
IV: intravenous
MDCK-MDR1: Madin–Darby canine kidney-multidrug resistance 1
PBMC: peripheral blood mononuclear cells
PD: pharmacodynamic PDB, Protein Data Bank
PDB: Protein Data Bank
P-gp: P-glycoprotein
PK: pharmacokinetic
OAT: organic anion transporter
OCT: organic cation transporter
SAR: structure–activity relationship
SPR: surface plasmon resonance
TGI: tumor-growth inhibition
TPSA: topological polar surface area
UDP: uridine diphosphate
UGT: UDP-glucuronosyltransferase
