# Supplementary material for: Exploiting the Cryptic αD Pocket of Casein Kinase 2α (CK2α) to Deliver Highly Potent and Selective Type 1 Inhibitors
Source: J Med Chem. 2025 Oct 14;68(20):21587–614. doi: 10.1021/acs.jmedchem.5c01807 (PMC12557390; doi:10.1021/acs.jmedchem.5c01807)
Supplement: Supplementary file 1 [file jm5c01807_si_001.pdf]

Supporting Information

Exploiting the Cryptic αD Pocket of Casein Kinase 2α (CK2α) to Deliver Highly Potent and Selective Type 1 Inhibitors

Paul A. Glossop,*,# Paul Brear,◊ Susanne Wright,▽ Neil Flanagan,▽ Melanie S. Glossop,▽ Charlotte A. L. Lane,▽ Richard P. Butt,▽ David R. Spring,Ψ Marko Hyvönen,*,◊ Darren Cawkill,*,▽

#Sandexis Medicinal Chemistry Ltd., Innovation House, Discovery Park, Ramsgate Road, Sandwich, Kent, CT13 9FF, U.K.

◊Department of Biochemistry, University of Cambridge, 80 Tennis Court Road, Cambridge, CB2 1GA, U.K.

ΨYusuf Hamied Department of Chemistry, University of Cambridge, Lensfield Road, Cambridge, CB2 1EW, U.K.

▽Apollo Therapeutics, 50–60 Station Road, Cambridge, CB1 2JH, U.K.

* Correspondence to: PG (paul.glossop@sandexis.co.uk), DC (darren.cawkill@apollotx.com) and MH (mh256@cam.ac.uk)

Table of contents

Supporting Information S1

Chemistry S4

Synthesis and analytical details for **9** S4

Synthesis and analytical details for **10** S4

Synthesis and analytical details for **11** S5

Synthesis and analytical details for **12** S5

Synthesis and analytical details for **13** S6

Synthesis and analytical details for 14 .... S7

Synthesis and analytical details for **18** S8

Synthesis and analytical details for **19** S9

Synthesis and analytical details for **20** S11

Synthesis and analytical details for **21** S13

Synthesis and analytical details for **22** S15

Synthesis and analytical details for **23** S16

Synthesis and analytical details for **24** S18

Synthesis and analytical details for **25** S20

Synthesis and analytical details for **26** S23
Synthesis and analytical details for **27** S24
Synthesis and analytical details for **28** S25
Synthesis and analytical details for **29** S25
Synthesis and analytical details for **30** S26
Synthesis and analytical details for **31** S27
Synthesis and analytical details for **32** S28
Synthesis and analytical details for **33** S29
Synthesis and analytical details for **35** S30
Synthesis and analytical details for **36** S32
Synthesis and analytical details for **37** S33
Synthesis and analytical details for **38** S34
Synthesis and analytical details for **39** S35
Synthesis and analytical details for **41** S36
Synthesis and analytical details for **42** S37
Synthesis and analytical details for **43** S37
Synthesis and analytical details for **44** S38
Synthesis and analytical details for **45** S39
Synthesis and analytical details for **46** S40
Synthesis and analytical details for **48** S41
Synthesis and analytical details for **49** S42
Synthesis and analytical details for **50** S43
Synthesis and analytical details for **51** S44
Synthesis and analytical details for **52** S45
Synthesis and analytical details for **53** S45
General Procedure for the Synthesis of **54a**–**m** and **55a**–**b**. S45
Synthesis and analytical details for **54a-m** S45
Synthesis and analytical details for **55a-b** S51
Synthesis and analytical details for **56a-h** S52
Synthesis and analytical details for **57a-i** S55
General Procedure for the Synthesis of **58a-i.** S58
Synthesis and analytical details for **58a-i** S58
HPLC analysis of **61f** S64
Initial Screening S65
X-ray Crystallography S69
Surface Plasmon Resonance S82

In vitro ADME and hERG S82
Thermodynamic Solubility Measurement S82
pK_a Determination S82
Hepatocyte Metabolite Identification S83
Permeability and Transporter Substrate Studies S84
Plasma Protein Binding Assay S85
Cytochrome P450 and Uridine 5'diphospho-glucuronosyltransferase Phenotyping S85
hERG Patch-clamp Assay S86

Chemistry

Synthesis and analytical details for **9**

3-(((2-chloro-[1,1'-biphenyl]-4-yl)methyl)amino)propanoic acid

A solution of 3-chloro-4-phenylbenzaldehyde[30] (10.0 g, 46.2 mmol) and 3-aminopropanoic acid (12.3 g, 138 mmol) in methanol (200 mL) was stirred at 34 °C for 1 h. $NaBH(OAc)_3$ (19.6 g, 92.3 mmol) was added slowly and the reaction mixture was stirred at 34 °C for 14 h. The reaction mixture was filtered and reduced *in vacuo* to give a residue that was purified by reverse-phase HPLC (column: Phenomenex Gemini-NX C18 75 × 30 mm × 3 µm; eluents: A) 0.1% FA in H_2O (v/v), B) acetonitrile; gradient: 20−80% B, 20 min) to afford the title compound (11.9 g, 89% yield) as a colourless oil.

LCMS (AM3): rt = 0.758 min, (290.1 $[M+H]^+$).

3-((tert-Butoxycarbonyl)((2-chloro-[1,1'-biphenyl]-4-yl)methyl)amino)propanoic acid (9)

To a solution of 3-(((2-chloro-[1,1'-biphenyl]-4-yl)methyl)amino)propanoic acid (11.9 g, 41.1 mmol) in THF (75 mL) and water (75 mL) was added sodium bicarbonate (5.18 g, 61.6 mmol) and $(Boc)_2O$ (10.8 g, 49.3 mmol) at 30 °C and the mixture was stirred for 2 h. The reaction mixture was filtered and concentrated, and the residue was purified by reverse-phase HPLC (column: Phenomenex Gemini-NX C18 75 × 30 mm × 3 µm; eluents: A) 0.1% FA in H_2O (v/v), B) acetonitrile; gradient: 20−80% B, 20 min) to afford title compound **9** (13.8 g, 86% yield) as a yellow oil.

LCMS (AM3): rt = 0.997 min, (412.1 $[M+Na]^+$).

Synthesis and analytical details for **10**

tert-Butyl ((2-chloro-[1,1'-biphenyl]-4-yl)methyl)(3-((3,3-diethoxypropyl)amino)-3-oxopropyl)carbamate

To a solution of acid **9** (10.0 g, 25.7 mmol), 3,3-diethoxypropan-1-amine (4.53 g, 30.8 mmol) and TEA (10.7 mL, 77.0 mmol) in THF (100 mL) was added T_3P (24.5 g, 38.5 mmol, 50% in ethyl acetate) at 0 °C and the mixture was stirred for 2 h. The mixture was diluted with water (500 mL) and extracted with ethyl acetate (200 mL × 2). The combined organic phases were washed with brine (200 mL), dried (Na_2SO_4) and reduced *in vacuo*. The residue was purified by column chromatography on silica gel eluting with petroleum ether/ethyl acetate (4:1) to give the title compound (11.2 g, 84% yield) as a colourless oil.

LCMS (AM3): rt = 1.057 min, (541.2 $[M+Na]^+$).

tert-Butyl ((2-chloro-[1,1'-biphenyl]-4-yl)methyl)(3-oxo-3-((3-oxopropyl)amino)propyl) carbamate (10)

A solution of tert-butyl ((2-chloro-[1,1'-biphenyl]-4-yl)methyl)(3-((3,3-diethoxypropyl)amino)-3-oxopropyl)carbamate (1.0 g, 1.9 mmol) in acetic acid (5 mL) and water (5 mL) was stirred at room temperature for 2 h. The mixture was basified with saturated aq. $NaHCO_3$ solution to pH 8 and extracted with ethyl acetate (50 mL × 2). The combined organic phases were washed with brine (50 mL), dried (Na_2SO_4) and reduced *in vacuo* to give title compound **10** (850 mg, 99% yield) as a colourless oil, which was used directly without further purification.

LCMS (AM3): rt = 0.961 min, (467.2 $[M+Na]^+$).

Synthesis and analytical details for **11**

Methyl 3-amino-4-(1-((2-(trimethylsilyl)ethoxy)methyl)-1H-pyrazol-3-yl)benzoate (11)

To a solution of methyl 3-amino-4-bromobenzoate (1.00 g, 4.35 mmol), 3-(4,4,5,5-tetramethyl-1,3,2-dioxaborolan-2-yl)-1-((2-(trimethylsilyl)ethoxy)methyl)-1H-pyrazole (2.26 g, 4.98 mmol) and K_3PO_4 (2.78 g, 13.1 mmol) in toluene (3 mL), ethanol (1 mL) and water (1.0 mL) was added Pd(dppf)Cl_2 (320 mg, 437 μmol) at room temperature under a nitrogen atmosphere. The reaction mixture was stirred at 90°C for 4 h. The mixture was diluted with water (30 mL), extracted with ethyl acetate (15 mL × 3) and the combined organic phases were washed with brine (15 mL), dried (Na_2SO_4), filtered and concentrated. The residue was purified on silica gel eluting with petroleum ether/ethyl acetate (20:1 → 5:1) to afford title compound **11** (1.50 g, 99% yield) as a yellow oil.

LCMS (AM3): rt = 0.989 min, (348.0 $[M+H]^+$).

Synthesis and analytical details for **12**

Methyl 3-((3-(3-((tert-butoxycarbonyl)((2-chloro-[1,1'-biphenyl]-4-yl)methyl)amino)propanamido)propyl)amino)-4-(1-((2-(trimethylsilyl)ethoxy)methyl)-1H-pyrazol-3-yl)benzoate

To the mixture of aldehyde **10** (150 mg, 337 μmol) and aniline **11** (120 mg, 345 μmol) in methanol (2.0 mL) was added $NaBH_3CN$ (30.0 mg, 477 μmol) and acetic acid (524 μmol, 30. 0 μL) and the mixture was stirred at room temperature for 48 h. The mixture was diluted with

water (10 mL) and extracted with ethyl acetate (15 mL × 3). The combined organic phases were washed with brine (20 mL), dried (Na_2SO_4), filtered and concentrated to afford the title compound (90 mg, 34% yield) as a yellow oil.

LCMS (AM3): rt = 1.215 min, (776.4 $[M+H]^+$).

Methyl 3-((3-(3-(((2-chloro-[1,1'-biphenyl]-4-yl)methyl)amino)propanamido)propyl)amino)-4-(1H-pyrazol-3-yl)benzoate

A mixture of methyl 3-((3-(3-((tert-butoxycarbonyl)((2-chloro-[1,1'-biphenyl]-4-yl)methyl)amino)propanamido)propyl)amino)-4-(1-((2-(trimethylsilyl)ethoxy)methyl)-1H-pyrazol-3-yl)benzoate (90.0 mg, 116 μmol) in a solution of HCl in methanol (4 M, 1.8 mL) was stirred at room temperature for 1 h. The solvent was removed under reduced pressure to afford the title compound (60 mg) as a yellow oil that was used in the next step without further purification.

LCMS (AM3): rt = 0.858 min, (546.3 $[M+H]^+$).

3-((3-(3-(((2-Chloro-[1,1'-biphenyl]-4-yl)methyl)amino)propanamido)propyl)amino)-4-(1H-pyrazol-3-yl)benzoic acid (12)

A mixture of methyl 3-((3-(3-(((2-chloro-[1,1'-biphenyl]-4-yl)methyl)amino)propanamido)propyl)amino)-4-(1H-pyrazol-3-yl)benzoate (60.0 mg, 109 μmol) in methanol (2.0 mL) and aq. NaOH (2.0 M, 2.0 mL) was stirred at room temperature for 4 h. The mixture was adjusted to pH 7 with aq. HCl (2 M) and purified by reverse-phase HPLC (column: Phenomenex Synergi C18 150 × 25 mm × 10 μm; eluents: A) 0.1% TFA in H_2O (v/v), B) acetonitrile; gradient: 15−55% B, 8 min) to afford title compound **12** (49 mg, 83% yield) as a yellow oil.

LCMS (AM3): rt = 1.945 min, (532.3 $[M+H]^+$).

^{1}H NMR (400 MHz, MeOD) δ: 7.78-7.77 (m, 2H), 7.68 (d, *J* = 1.6Hz, 1H), 7.65 (br s, 1H), 7.46-7.44 (m, 1H), 7.42-7.40 (m, 7H), 6.83 (d, *J* = 2.8Hz, 1H), 4.28 (s, 2H), 3.43-3.39 (m, 4H), 3.35-3.33 (m, 2H), 2.73-2.69 (m, 2H), 2.01-1.96 (m, 2H).

Synthesis and analytical details for **13**

Methyl 4-bromo-1-(tetrahydro-2H-pyran-2-yl)-1H-indazole-6-carboxylate

To a mixture of methyl 4-bromo-1H-indazole-6-carboxylate (2.0 g, 7.8 mmol) in THF (20 mL) was added 3,4-dihydro-2H-pyran (1.32 g, 15.7 mmol, 1.43 mL) and 4-methylbenzenesulfonic

acid (149 mg, 0.784 mmol) at room temperature. The mixture was heated to 50 °C and stirred for 12 h. The mixture was diluted with saturated aq. $NaHCO_3$ (30 mL) and extracted with ethyl acetate (30 mL × 3). The combined organic phases were washed with brine (150 mL), dried (Na_2SO_4), filtered and reduced *in vacuo*. The residue was purified by column chromatography on silica gel eluting with petroleum ether/ethyl acetate (50:1) to afford the title compound (2.4 g, 90% yield) as a white solid.

^{1}H NMR (400 MHz, $CHCl_3$-*d*$CHCl_3$-*d*) δ: 8.30 (s, 1H), 8.08 (s, 1H), 8.00 (d, *J* = 1.2 Hz, 1H), 5.78 (dd, *J* = 9.2, 2.8 Hz, 1H), 4.07-3.95 (m, 4H), 3.83-3.73 (m, 1H), 2.60-2.44 (m, 1H), 2.20-2.07 (m, 2H), 1.82-1.70 (m, 3H).

Methyl 4-amino-1-(tetrahydro-2H-pyran-2-yl)-1H-indazole-6-carboxylate (13)

To a mixture of methyl 4-bromo-1-(tetrahydro-2H-pyran-2-yl)-1H-indazole-6-carboxylate (1.9 g, 5.6 mmol) in DMSO (30 mL) was added ammonium hydroxide (2.07 g, 14.8 mmol), CuI (224 mg, 1.18 mmol), (2S,4S)-4-hydroxypyrrolidine-2-carboxylic acid (301 mg, 2.30 mmol) and K_2CO_3 (2.39 g, 17.3 mmol) at room temperature under a nitrogen atmosphere. The mixture was heated to 90 °C and stirred for 16 h. The mixture was diluted with water (40 mL), extracted with ethyl acetate (30 mL × 3) and the combined organic phases were washed with brine (50 mL × 3), dried (Na_2SO_4), filtered and reduced *in vacuo* to afford title compound **13** (1.2 g, 78% yield) as a yellow solid.

LCMS (AM3): rt = 0.727 min, (276.1 $[M+H]^+$).

Synthesis and analytical details for 14

Methyl 4-((3-(3-((tert-butoxycarbonyl)((2-chloro-[1,1'-biphenyl]-4-yl)methyl)amino)propanamido)propyl)amino)-1-(tetrahydro-2H-pyran-2-yl)-1H-indazole-6-carboxylate

To a mixture of amine **13** (1.29 g, 2.91 mmol) and aldehyde **10** (1.0 g, 3.6 mmol) in DCE (30 mL) was added $NaBH(OAc)_3$ (1.54 g, 7.26 mmol) at room temperature and the mixture was stirred for 16 h. The mixture was reduced *in vacuo* and the residue was purified by reverse-phase HPLC (column: Phenomenex Synergi Max-RP 250 × 50 mm × 10 μm; eluents: A) 10 mM NH_4HCO_3 in H_2O, B) acetonitrile; gradient: 60–90% B, 35 min) to afford the title compound (460 mg, 18% yield) as a white solid.

LCMS (AM3): rt = 1.096 min, (704.4 $[M+H]^+$).

Methyl 4-((3-(3-(((2-chloro-[1,1'-biphenyl]-4-yl)methyl)amino)propanamido)propyl)amino)-1H-indazole-6-carboxylate

A mixture of methyl 4-((3-(3-((tert-butoxycarbonyl)((2-chloro-[1,1'-biphenyl]-4-yl)methyl)amino)propanamido)propyl)amino)-1-(tetrahydro-2H-pyran-2-yl)-1H-indazole-6-carboxylate (144 mg, 204 mmol) in a solution of HCl in methanol (4 M, 3 mL) was stirred at room temperature for 1 h. The solvent was removed under reduced pressure to afford the title compound (100 mg, 97% yield) as a yellow oil, which was used directly without purification.

LCMS (AM3): rt = 0.817 min, (520.3 $[M+H]^+$).

4-((3-(3-(((2-Chloro-[1,1'-biphenyl]-4-yl)methyl)amino)propanamido)propyl)amino)-1H-indazole-6-carboxylic acid (14)

A mixture of methyl 4-((3-(3-(((2-chloro-[1,1'-biphenyl]-4-yl)methyl)amino)propanamido)propyl)amino)-1H-indazole-6-carboxylate (100 mg, 192 mmol) in methanol (2 mL) and aq. NaOH (2 M, 3.5 mL) was stirred at room temperature for 4 h. The solvent was removed under reduced pressure and the residue was purified by reverse-phase HPLC (column: Phenomenex Synergi C18 150 × 25 mm × 10 μm; eluents: A) 0.1% TFA in H_2O (v/v), B) acetonitrile; gradient: 15–45% B, 6 min). The product was further purified by reverse-phase HPLC (column: Phenomenex Gemini 150 × 25 mm × 10 μm; eluents: A) 0.05% NH_4OH in H_2O (v/v), B) acetonitrile; gradient: 15–45% B, 12 min) to afford title compound **14** (20 mg, 14% yield) as a white solid.

LCMS (AM6): rt = 1.825 min, (506.2 $[M+H]^+$).

^{1}H NMR (400 MHz, DMSO) δ 8.21 (s, 1H), 8.03 - 8.02 (m, 1H), 7.50 (s, 1H),7.44 - 7.30 (m, 8H), 6.60 (s, 1H), 6.34 (s, 1H), 3.73 (s, 2H), 3.20 - 3.16 (m, 4H), 2.74 - 2.70 (m, 2H), 2.30 - 2.27 (m, 2H), 1.81 - 1.77 (m, 2H).

Synthesis and analytical details for **18**

5-[(2-aminoethyl)amino]benzo[c]2,6-naphthyridine-8-carboxylic acid

A mixture of ester **28** (300 mg, 1.01 mmol) and LiOH monohydrate (212 mg, 5.06 mmol) in methanol (5 mL) was stirred at room temperature for 1 h. The reaction mixture was adjusted to pH 6 with aqueous HCl (1 M), then diluted with water (10 mL) and extracted with ethyl acetate (3 × 10 mL). The combined organic layers were washed with water (2 × 10 mL) and brine (10 mL), dried (Na_2SO_4) and filtered. The filtrate was reduced *in vacuo* to give the title compound

(200 mg, 70% yield) as a dark-brown oil, which was used in the next step without further purification.

5-({2-[(4-{[(tert-butoxy)carbonyl]({2-chloro-[1,1'-biphenyl]-4-yl}methyl)amino}butyl)amino]ethyl}amino)benzo[c]2,6-naphthyridine-8-carboxylic acid

A mixture of aldehyde **27** (200 mg, 516 mmol), 5-[(2-aminoethyl)amino]benzo[c]2,6-naphthyridine-8-carboxylic acid (140 mg, 496 mmol) and $NaBH_3CN$ (48.0 mg, 764 mmol) in methanol (2 mL) was stirred at 70°C for 2 h. The reaction mixture was diluted with water (10 mL) and extracted with ethyl acetate (3 × 10 mL). The combined organic layers were washed with water (2 × 10 mL) and brine (10 mL), dried (Na_2SO_4) and filtered. The filtrate was reduced *in vacuo* to give the title compound (200 mg, = 59% yield) as a dark-brown solid, which was used in the next step without further purification.

5-{[2-({4-[({2-chloro-[1,1'-biphenyl]-4-yl}methyl)amino]butyl}amino)ethyl]amino}benzo[c]2,6-naphthyridine-8-carboxylic acid (18)

A solution of 5-({2-[(4-{[(tert-butoxy)carbonyl]({2-chloro-[1,1'-biphenyl]-4-yl}methyl)amino}butyl)amino]ethyl}amino)benzo[c]2,6-naphthyridine-8-carboxylic acid (200 mg, 306 mmol) in ethyl acetate (20 mL) was treated with a solution of HCl in ethyl acetate (4 M, 20 mL) and stirred at room temperature for 2 h. The reaction mixture was reduced *in vacuo* and purified by reverse-phase HPLC (column: Boston pH-lex 150 × 25 mm × 10 µm; eluents: A) 0.1% TFA in H_2O (v/v), B) acetonitrile; gradient: 21–48% B, 9 min) and the resulting residue was triturated with water (100 mL) to give title compound **18** (8.6 mg, 5% yield) as a yellow solid.

LCMS (AM3): rt = 0.760 min, (554.3 $[M+H]^+$).

^1H NMR (400 MHz, MeOH-d_4) δ: 10.27 (s, 1H), 9.08 (d, J = 6.0 Hz, 1H), 8.85 (d, J = 6.1 Hz, 1H), 8.80 (d, J = 8.5 Hz, 1H), 8.70 (s, 1H), 8.20-8.17 (m, 1H), 7.72 (d, J = 1.6 Hz, 1H), 7.56-7.52 (m, 1H), 7.49-7.37 (m, 6H), 4.31-4.21 (m, 4H), 3.59 (t, J = 5.7 Hz, 2H), 3.17 (s, 4H), 1.96-1.88 (s, 4H).

Synthesis and analytical details for **19**

2-(((2-chloro-[1,1'-biphenyl]-4-yl)methyl)amino)ethanol

A mixture of 3-chloro-4-phenylbenzaldehyde[30] (1.0 g, 4.6 mmol) and 2-aminoethanol (846 mg, 13.9 mmol) in methanol (20 mL) was stirred at room temperature for 1 h. $NaBH(OAc)_3$ (1.96

g, 9.23 mmol) was added, and the reaction mixture was stirred for 4 h. The reaction mixture was poured into water (100 mL) and extracted with ethyl acetate (60 mL × 2). The combined organic phases were washed with brine (100 mL), dried (Na_2SO_4), filtered and reduced *in vacuo* to afford the title compound (1.07 g, 89% yield) as a colourless oil, which was used directly without purification.

LCMS (AM3): rt = 0.745 min, (261.9 $[M+H]^+$).

tert-butyl ((2-chloro-[1,1'-biphenyl]-4-yl)methyl)(2-hydroxyethyl)carbamate

To a mixture of 2-(((2-chloro-[1,1'-biphenyl]-4-yl)methyl)amino)ethanol (1.07 g, 4.09 mmol) and $NaHCO_3$ (687 mg, 8.18 mmol) in THF (10 mL) and water (10 mL) was added Boc_2O (1.07 g, 4.91 mmol) at room temperature, and the reaction mixture stirred for 16 h. The reaction mixture was poured into water (100 mL) and extracted with ethyl acetate (50 mL × 2). The combined organic phases were washed with brine (100 mL), dried (Na_2SO_4), filtered and reduced *in vacuo*. The residue was purified by column chromatography on silica gel eluting with petroleum ether/ethyl acetate (5:1) to afford the title compound (1.24 g, 84% yield) as a colourless oil.

LCMS (AM3): rt = 0.989 min, (384.2 $[M+Na]^+$).

tert-butyl ((2-chloro-[1,1'-biphenyl]-4-yl)methyl)(2-oxoethyl)carbamate

To a solution of tert-butyl ((2-chloro-[1,1'-biphenyl]-4-yl)methyl)(2-hydroxyethyl)carbamate (780 mg, 2.16 mmol) in DCM (15 mL) was added DMP (1.1 g, 2.6 mmol) in portions at room temperature and the reaction mixture then stirred for 18 h. The reaction mixture was filtered, and the filtrate was reduced *in vacuo*. The residue was purified by column chromatography on silica gel eluting with petroleum ether/ethyl acetate (20:1) to afford the title compound (660 mg, 85% yield) as a colourless oil.

^1H NMR (400 MHz, DMSO-d_6) δ: 9.58-9.54 (m, 1H), 7.47-7.32 (m, 7H), 7.23-7.16 (m, 1H), 4.57-4.16 (m, 2H), 3.89-3.50 (m, 2H), 1.52-1.50 (m, 9H).

Methyl 5-((2-((2-((tert-butoxycarbonyl)((2-chloro-[1,1'-biphenyl]-4-yl)methyl)amino)ethyl)amino)ethyl)amino)benzo[c][2,6]naphthyridine-8-carboxylate

To a mixture of tert-butyl ((2-chloro-[1,1'-biphenyl]-4-yl)methyl)(2-oxoethyl)carbamate (112 mg, 0.311 mmol) and NaOAc (102 mg, 1.24 mmol) in methanol (5 mL) was added amine **28** (170 mg, 0.311 mmol, HCl salt) followed by $NaBH_3CN$ (80 mg, 1.3 mmol) at room temperature, and the reaction then stirred for 16 h. The reaction mixture was filtered and

reduced *in vacuo* to give a residue, which was purified by reverse-phase HPLC (column: Phenomenex Gemini-NX C18 75 × 30 mm × 3 μm; eluents: A) 0.1% TFA in H_2O (v/v), B) acetonitrile; gradient: 20–55% B, 20 min) to afford the title compound (40 mg, 20% yield) as a yellow solid.

LCMS (AM1): rt = 0.809 min, (640.2 $[M+H]^+$).

5-((2-((2-(((2-chloro-[1,1'-biphenyl]-4-yl)methyl)amino)ethyl)amino)ethyl)amino)benzo[c][2,6]naphthyridine-8-carboxylic acid (19)

To a mixture of methyl 5-((2-((2-((tert-butoxycarbonyl)((2-chloro-[1,1'-biphenyl]-4-yl)methyl)amino)ethyl)amino)ethyl)amino)benzo[c][2,6]naphthyridine-8-carboxylate (40 mg, 0.063 mmol) in THF (1 mL) and water (1 mL) was added LiOH monohydrate (11 mg, 0.26 mmol) at room temperature and the mixture then stirred for 8 h. The reaction mixture was acidified with conc. HCl (1 mL) and stirred at room temperature for 4 h. The reaction mixture was reduced *in vacuo* and purified by reverse-phase HPLC (column: Phenomenex Synergi C18 150 × 25 mm × 10 μm; eluents: A) 0.05% HCl in H_2O (v/v), B) acetonitrile; gradient: 10–30% B, 9 min) to afford title compound **19** (17.7 mg, 46% yield, HCl salt) as a yellow solid.

LCMS (AM1): rt = 0.620 min, (526.1 $[M+H]^+$).

^{1}H NMR (400 MHz, MeOH-d_4) δ: 10.23 (s, 1H), 9.02 (d, J = 6 Hz, 1H), 8.77 (d, J = 8.8 Hz, 1H), 8.72 (d, J = 6 Hz, 1H), 8.62 (s, 1H), 8.15 (dd, J = 8.8 Hz, 1.6 Hz, 1H), 7.73 (d, J = 1.6 Hz, 1H), 7.56 (dd, J = 8.0 Hz, 1.6 Hz, 1H), 7.47-7.36 (m, 6H), 4.34 (s, 2H), 4.23 (t, J = 5.2 Hz, 2H), 3.67 (t, J = 5.2 Hz, 2H), 3.62-3.57 (m, 4H).

Synthesis and analytical details for **20**

N-((2-chloro-[1,1'-biphenyl]-4-yl)methyl)-3,3-diethoxypropan-1-amine

A mixture of 3-chloro-4-phenylbenzaldehyde[30] (1.0 g, 4.2 mmol), 3,3-diethoxypropan-1-amine (800 mg, 5.43 mmol) and molecular sieves (4 Å, 2 g) in methanol (20 mL) was stirred at room temperature for 2 h, then $NaBH_3CN$ (1.50 g, 23.9 mmol) was added and the mixture was stirred for 4 h. The mixture was poured into water (50 mL) and extracted with ethyl acetate (50 mL × 3). The combined organic phases were washed with brine (100 mL), dried (Na_2SO_4) and reduced *in vacuo* to afford the title compound (1.7 g), which was used in the next step without further purification.

LCMS (AM1): rt = 0.714 min, (348.1 $[M+H]^+$).

tert-Butyl ((2-chloro-[1,1'-biphenyl]-4-yl)methyl)(3,3-diethoxypropyl)carbamate

To a mixture of N-((2-chloro-[1,1'-biphenyl]-4-yl)methyl)-3,3-diethoxypropan-1-amine (0.50 g, 1.4 mmol) and $NaHCO_3$ (0.30 g, 3.6 mmol) in THF (20 mL) and water (7 mL) was added $(Boc)_2O$ (0.65 g, 3.0 mmol) at room temperature and the mixture was stirred for 5 h. The mixture was poured into water (50 mL) and extracted with ethyl acetate (50 mL × 3). The combined organic phases were washed with brine (50 mL), dried (Na_2SO_4) and reduced *in vacuo* to afford the title compound (0.9 g), which was used directly without further purification.

LCMS (AM1): rt = 1.008 min, (470.2 $[M+Na]^+$).

tert-Butyl ((2-chloro-[1,1'-biphenyl]-4-yl)methyl)(3-oxopropyl)carbamate

A mixture of tert-butyl ((2-chloro-[1,1'-biphenyl]-4-yl)methyl)(3,3-diethoxypropyl)carbamate (0.49 g, 1.1 mmol) in acetic acid (5 mL), water (5 mL) and THF (3 mL) was stirred at room temperature for 40 h. The solution was slowly poured into saturated aq. $NaHCO_3$ solution (200 mL) and extracted with ethyl acetate (50 mL × 2). The combined organic phases were washed with brine (100 mL), dried (Na_2SO_4) and reduced *in vacuo*. The residue was purified by column chromatography on silica gel eluting with petroleum ether/ethyl acetate (12:1) to afford the title compound (220 mg, 50% yield) as a colourless oil.

LCMS (AM3): rt = 1.075 min, (318.2 $[M\text{-}tBu+2H]^+$).

Methyl 5-((2-((3-((tert-butoxycarbonyl)((2-chloro-[1,1'-biphenyl]-4-yl)methyl)amino)propyl)amino)ethyl)amino)benzo[c][2,6]naphthyridine-8-carboxylate

A mixture of tert-butyl ((2-chloro-[1,1'-biphenyl]-4-yl)methyl)(3-oxopropyl)carbamate (210 mg, 0.562 mmol), amine **28** (206 mg, 0.618 mmol, HCl salt) and NaOAc (138 mg, 1.69 mmol) in DCE (10 mL) was stirred at room temperature for 1 h, then $NaBH(OAc)_3$ (238 mg, 1.12 mmol) was added and the reaction mixture was stirred for 15 h. The reaction mixture was reduced *in vacuo* and the residue was purified by reverse-phase HPLC (column: Phenomenex Synergi C18 150 × 25 mm × 10 μm; eluents: A) 0.1% FA in H_2O (v/v), B) acetonitrile; gradient: 10–90% B, 20 min) to afford the title compound (110 mg, 29% yield) as light-yellow solid.

LCMS (AM3): rt = 0.921 min, (654.2 $[M+H]^+$).

5-((2-((3-((tert-Butoxycarbonyl)((2-chloro-[1,1'-biphenyl]-4-yl)methyl)amino)propyl)amino)ethyl)amino)benzo[c][2,6]naphthyridine-8-carboxylic acid

To a mixture of methyl 5-((2-((3-((tert-butoxycarbonyl)((2-chloro-[1,1'-biphenyl]-4-yl)methyl)amino)propyl)amino)ethyl)amino)benzo[c][2,6]naphthyridine-8-carboxylate (110 mg, 0.164 mmol) in THF (2 mL), water (2 mL) and methanol (2 mL) was added LiOH monohydrate (14 mg, 0.33 mmol) at room temperature and the resulting mixture was stirred for 1 h. The reaction mixture was acidified with aq. HCl (1 M) to pH 5 and the resulting mixture was extracted with ethyl acetate (20 mL × 3). The combined organic phases were washed with brine (50 mL), dried (Na_2SO_4), filtered and reduced *in vacuo* to afford the title compound (100 mg) as a light-yellow solid, which was used in the next step without further purification.

LCMS (AM3): rt = 0.869 min, (640.2 $[M+H]^+$).

5-((2-((3-(((2-Chloro-[1,1'-biphenyl]-4-yl)methyl)amino)propyl)amino)ethyl)amino)benzo[c][2,6]naphthyridine-8-carboxylic acid (20)

To a mixture of 5-((2-((3-((tert-butoxycarbonyl)((2-chloro-[1,1'-biphenyl]-4-yl)methyl)amino)propyl)amino)ethyl)amino)benzo[c][2,6]naphthyridine-8-carboxylic acid (100 mg, 0.156 mmol) in DCM (10 mL) was added TFA (27 mmol) at room temperature and the mixture was stirred for 16 h. The reaction mixture was reduced *in vacuo* and the residue was purified by reverse-phase HPLC (column: Phenomenex Synergi C18 150 × 25 mm × 10 μm; eluents: A) 0.225% FA in H_2O (v/v), B) acetonitrile; gradient: 10–40% B, 10 min) to afford title compound **20** (40 mg, 41% yield, 2 × TFA salt) as a light-yellow solid.

LCMS (AM1): rt = 0.631 min, (540.2 $[M+H]^+$).

^{1}H NMR (400 MHz, MeOH-d_4) δ: 9.94 (s, 1H), 8.81 (d, J = 5.6 Hz, 1H), 8.57 (d, J = 8.4 Hz, 1H), 8.28 (s, 2H), 8.09 (d, J = 5.6 Hz, 1H), 8.00 (d, J =8.0 Hz, 1H), 7.87 (s, 1H), 7.49 (s, 1H), 7.43–7.28 (m, 7H), 4.00 (t, J = 4.8 Hz, 2H), 3.97 (s, 2H), 3.43 (t, J = 4.8 Hz, 2H), 3.28–3.25 (m, 2H), 3.05 (t, J = 7.2 Hz, 2H), 2.18–2.10 (quin, 2H).

Synthesis and analytical details for **21**

Methyl 5-((3-((tert-butoxycarbonyl)amino)propyl)amino)benzo[c][2,6]naphthyridine-8-carboxylate

A mixture of methyl 5-chlorobenzo [*c*] 2,6-naphthyridine-8 carboxylate[14] (10.0 g, 36.7 mmol), tert-butyl N-(3-aminopropyl)carbamate (8.31 g, 47.7 mmol) and DIPEA (16.0 mL, 91.9 mmol) in DMSO (150 mL) was heated to 70 °C and stirred for 15 h. The reaction mixture was added

to water (1 L) and extracted with ethyl acetate (500 mL × 2). The combined organic phases were washed with brine (500 mL), dried (Na_2SO_4), filtered and reduced *in vacuo*. The crude product was triturated in ethanol (100 mL) and then filtered and dried under vacuum to afford the title compound (10.2 g, 66% yield) as a yellow solid.

LCMS (AM1): rt = 0.833 min, (411.2 $[M+H]^+$).

Methyl 5-[(3-aminopropyl)amino]benzo[c]2,6-naphthyridine-8-carboxylate

To a solution of methyl 5-((3-((tert-butoxycarbonyl)amino)propyl)amino)benzo[c][2,6] naphthyridine-8-carboxylate (155 mg, 0.380 mmol) in 1,4-dioxane (5 mL) was added a solution of HCl in 1,4-dioxane (4 M, 15 mL) at room temperature and the mixture was stirred for 0.5 h. The mixture was reduced *in vacuo* to give a residue which was purified by reverse-phase HPLC (column: Phenomenex Synergi C18 150 × 25 mm × 10 μm; eluents: A) 0.1% HCl in H_2O (v/v), B) acetonitrile; gradient: 10–90% B, 20 min) to afford the title compound (100 mg, 74% yield, HCl salt) as a yellow solid.

LCMS (AM1): rt = 0.628 min, (311.1 $[M+H]^+$).

^{1}H NMR (400 MHz, DMSO-d_6) δ: 10.18 (s, 1H), 9.05 (d, J = 5.6 Hz, 1H), 8.88 (d, J = 8.4 Hz, 2H), 8.20-8.13 (m, 3H), 8.01 (d, J = 7.9 Hz, 1H), 3.94 (s, 3H), 3.51-3.48 (m, 2H), 3.42 (d, J = 5.1 Hz, 2H), 2.15-2.07 (m, 2H).

5-({3-[(4-{[(tert-butoxy)carbonyl]({2-chloro-[1,1'-biphenyl]-4-yl}methyl)amino}butyl)amino]propyl}amino)benzo[c]2,6-naphthyridine-8-carboxylic acid

A mixture of aldehyde **27** (100 mg, 258 mmol), methyl 5-[(3-aminopropyl)amino]benzo[c]2,6-naphthyridine-8-carboxylate (80.0 mg, 258 mmol) and $NaBH_3CN$ (25.0 mg, 398 mmol) in methanol (2 mL) was stirred at 70°C for 2 h. The reaction mixture was cooled and treated with LiOH monohydrate (54.0 mg, 1.29 mmol) in methanol (3 mL) and stirred at 70°C for 1 h. The reaction mixture was adjusted to pH 5 with aq. HCl (1 M), then diluted with water (10 mL) and extracted with ethyl acetate (3 × 10 mL). The combined organic layers were washed with water (2 × 10 mL) and brine (10 mL), dried (Na_2SO_4), filtered and reduced *in vacuo* to give the title compound (150 mg, 88% yield) as a yellow solid, which was used in the next step without further purification.

5-{[3-({4-[({2-chloro-[1,1'-biphenyl]-4-yl}methyl)amino]butyl}amino)propyl]amino}benzo[c]2,6-naphthyridine-8-carboxylic acid (21)

To a solution of 5-({3-[(4-{[(tert-butoxy)carbonyl]({2-chloro-[1,1'-biphenyl]-4-yl}methyl)amino}butyl)amino]propyl}amino)benzo[c]2,6-naphthyridine-8-carboxylic acid (150 mg, 224 mmol) in ethyl acetate (10 mL) was added a solution of HCl in ethyl acetate (4 M, 20 mL) and the mixture stirred at room temperature for 2 h. The reaction mixture was reduced *in vacuo* and purified by reverse-phase HPLC (column: Boston pH-lex 150 × 25 mm × 10 µm; eluents: A) 0.1% TFA in H_2O (v/v), B) acetonitrile; gradient: 21–48% B, 9 min) to give title compound **21** (11 mg, 9% yield) as a yellow solid.

LCMS (AM3): rt = 0.760 min, (568.3 $[M+H]^+$).

^1H NMR (400 MHz, MeOH-d_4) δ: 10.04 (s, 1H), 8.89 (d, J = 5.6 Hz, 1H), 8.73 (d, J = 8.4 Hz, 1H), 8.45 (d, J = 1.3 Hz, 1H), 8.24 (d, J = 5.6 Hz, 1H), 8.07 (d, J = 8.4 Hz, 1H), 7.67 (s, 1H), 7.49-7.38 (m, 7H), 4.25 (s, 2H), 3.93 (t, J = 6.4 Hz, 2H), 3.20-3.16 (t, 2H), 3.15-3.10 (t, 2H), 3.09-3.04 (t, 2H), 2.25-2.17 (quin, J = 6.8 Hz, 2H), 1.94-1.83 (m, 2H), 1.82-1.72 (m, 2H).

Synthesis and analytical details for **22**

Methyl 5-((3-(3-((tert-butoxycarbonyl)((2-chloro-[1,1'-biphenyl]-4-yl)methyl)amino)propanamido)propyl)amino)benzo[c][2,6]naphthyridine-8-carboxylate

To a mixture of 3-((tert-butoxycarbonyl)((2-chloro-[1,1'-biphenyl]-4-yl)methyl)amino)propanoic acid (300 mg, 0.769 mmol), methyl 5-[(3-aminopropyl)amino]benzo[c]2,6-naphthyridine-8-carboxylate (370 mg, 1.07 mmol, HCl salt) and TEA (4.5 mmol, 0.62 mL) in DCM (10 mL) was added T_3P (1.5 mmol, 0.9 mL, 50% in ethyl acetate) dropwise at 0 °C. The reaction mixture was then warmed to room temperature and stirred for 15 h. The mixture was reduced *in vacuo* and the residue was purified by reverse-phase HPLC (column: Phenomenex Synergi C18 150 × 25 mm × 10 µm; eluents: A) 0.1% TFA in H_2O (v/v), B) acetonitrile; gradient: 10–90% B, 20 min) to afford the title compound (130 mg, 20% yield, TFA salt) as a light-yellow solid.

LCMS (AM1): rt = 0.837 min, (682.2 $[M+H]^+$).

5-((3-(3-((tert-Butoxycarbonyl)((2-chloro-[1,1'-biphenyl]-4-yl)methyl)amino)propanamido)propyl)amino)benzo[c][2,6]naphthyridine-8-carboxylic acid

To a solution of methyl 5-((3-(3-((tert-butoxycarbonyl)((2-chloro-[1,1'-biphenyl]-4-yl)methyl)amino)propanamido)propyl)amino)benzo[c][2,6]naphthyridine-8-carboxylate (120 mg, 0.176 mmol) in THF (5 mL), methanol (5 mL) and water (5 mL) was added LiOH monohydrate (14.8 mg, 0.352 mmol) at room temperature and the mixture was stirred for 1 h. The reaction mixture was poured into water (20 mL) and acidified to pH 5 with aq. HCl (1 M). The mixture was extracted with ethyl acetate (20 mL × 2), and the combined organic phases were washed with brine (30 mL), dried (Na_2SO_4), filtered and reduced *in vacuo* to afford the title compound (120 mg, 97% yield) as a light-yellow solid, which was used in the next step without further purification.

LCMS (AM3): rt = 0.918 min, (668.3 $[M+H]^+$).

5-((3-(3-(((2-Chloro-[1,1'-biphenyl]-4-yl)methyl)amino)propanamido)propyl)amino)benzo[c][2,6]naphthyridine-8-carboxylic acid (22)

To a solution of 5-((3-(3-((tert-butoxycarbonyl)((2-chloro-[1,1'-biphenyl]-4-yl)methyl)amino)propanamido)propyl)amino)benzo[c][2,6]naphthyridine-8-carboxylic acid (106 mg, 0.151 mmol) in DCM (10 mL) was added TFA (1.30 g, 11.4 mmol) at room temperature and the mixture was stirred for 16 h. The reaction mixture was reduced *in vacuo* and the residue was purified by reverse-phase HPLC (column: Phenomenex Luna C18 250 × 50 mm × 10 μm; eluents: A) 0.1% TFA in H_2O (v/v), B) acetonitrile; gradient: 15−40% B, 10 min) to afford title compound **22** (100 mg, 82% yield, TFA salt) as a light-yellow solid.

LCMS (AM3): rt = 0.709 min, (568.1 $[M+H]^+$).

^{1}H NMR (400 MHz, MeOH-d_4) δ: 10.07 (s, 1H), 8.97 (d, J = 5.6 Hz, 1H), 8.77 (d, J = 8.4 Hz, 1H), 8.59 (d, J = 1.2 Hz, 1H), 8.37 (d, J = 5.6 Hz, 1H), 8.15 (d, J =8.0 Hz, 1H), 7.67 (d, J = 1.6 Hz, 1H), 7.50–7.47 (m, 1H), 7.46–7.37 (m, 6H), 4.30 (s, 2H), 3.87 (t, J = 6.4 Hz, 2H), 3.42 (t, 2H), 3.38 (t, 2H), 2.78 (t, J = 6.4 Hz, 2H), 2.11–2.04 (quin, 2H).

Synthesis and analytical details for **23**

5-((3-((tert-butoxycarbonyl)amino)propyl)amino)benzo[c][2,6]naphthyridine-8-carboxylic acid

To a mixture of methyl 5-((3-((tert-butoxycarbonyl)amino)propyl)amino)benzo[c][2,6] naphthyridine-8-carboxylate (10.2 g, 24.1 mmol) in THF (50 mL) and water (50 mL) was added LiOH monohydrate (3.46 g, 82.5 mmol) at room temperature. The reaction mixture was

heated to 40 °C and stirred for 15 h. The reaction mixture was added to water (200 mL) and the pH adjusted to pH3 with aq. HCl (1 M). The resulting precipitate was collected by filtration and dried *in vacuo* to afford the title compound (9.5 g, 98% yield) as a yellow solid, which was used in the next step without further purification.

LCMS (AM1): rt = 0.765 min, (397.2 $[M+H]^+$).

tert-butyl (3-((8-carbamoylbenzo[c][2,6]naphthyridin-5-yl)amino)propyl)carbamate

To a mixture of 5-((3-((tert-butoxycarbonyl)amino)propyl)amino)benzo[c][2,6]naphthyridine-8-carboxylic acid (9.50 g, 23.7 mmol), NH_4Cl (7.60 g, 142 mmol), HOBt (6.39 g, 47.3 mmol) and DIPEA (16.5 mL, 94.7 mmol) in DMF (150 mL) was added EDCI (9.07 g, 47.3 mmol) at room temperature. The reaction mixture was heated to 70 °C and stirred for 14 h. The reaction mixture was added to water (600 mL) and extracted with ethyl acetate (200 mL × 5). The combined organic phases were washed with brine (500 mL × 2), dried (Na_2SO_4), filtered and reduced *in vacuo* to afford the title compound (9.1 g, 94% yield) as a yellow solid, which was used directly without further purification.

LCMS (AM1): rt = 0.756 min, (396.2 $[M+H]^+$).

5-((3-aminopropyl)amino)benzo[c][2,6]naphthyridine-8-carboxamide

To a mixture of tert-butyl (3-((8-carbamoylbenzo[c][2,6]naphthyridin-5-yl)amino)propyl)carbamate (9.10 g, 23.0 mmol) in 1,4-dioxane (50 mL) was added a solution of HCl in 1,4-dioxane (4 M, 50 mL) at room temperature and the mixture was stirred for 15 h. The reaction mixture was reduced *in vacuo* to afford the title compound (9.2 g, HCl salt) as a yellow solid, which was used in the next step without further purification.

tert-Butyl (3-((3-((8-carbamoylbenzo[c][2,6]naphthyridin-5-yl)amino)propyl)amino)-3-oxopropyl)((2-chloro-[1,1'-biphenyl]-4-yl)methyl)carbamate

To a mixture of 3-((tert-butoxycarbonyl)((2-chloro-[1,1'-biphenyl]-4-yl)methyl)amino)propanoic acid (117.5 mg, 301.4 mmol) and HATU (171.9 mg, 452.1 mmol) in DMF (5 mL) was added DIPEA (77.9 mg, 603 mmol) followed by 5-((3-aminopropyl)amino)benzo[c][2,6]naphthyridine-8-carboxamide (100 mg, 301 mmol) at room temperature and the mixture was stirred for 2 h. The mixture was poured into water (50 mL) and extracted with ethyl acetate (50 mL × 2). The combined organic phases were washed with brine (50 mL × 3), dried (Na_2SO_4) and reduced *in vacuo* to give the title compound (150 mg) as a brown solid, which was used in the next step without further purification.

LCMS (AM3): rt = 0.861 min, (667.4 $[M+H]^+$).

5-((3-(3-(((2-Chloro-[1,1'-biphenyl]-4-yl)methyl)amino)propanamido)propyl)amino)benzo[c][2,6]naphthyridine-8-carboxamide (23)

To a solution of tert-butyl (3-((3-((8-carbamoylbenzo[c][2,6]naphthyridin-5-yl)amino)propyl)amino)-3-oxopropyl)((2-chloro-[1,1'-biphenyl]-4-yl)methyl)carbamate (150 mg, 225 mmol) in DCM (3 mL) was added TFA (1 mL) at room temperature and the reaction mixture was stirred for 0.5 h. The reaction mixture was reduced *in vacuo* and the residue was purified by reverse-phase HPLC (column: Phenomenex Synergi C18 150 × 25 mm × 10 μm; eluents: A) 0.1% TFA in H_2O (v/v), B) acetonitrile; gradient: 15–45% B, 10 min) to afford title compound **23** (69.5 mg, 45% yield, TFA salt) as a yellow solid.

LCMS (AM3): rt = 0.782 min, (567.2 $[M+H]^+$).

^1H NMR (400 MHz, MeOH-d_4) δ: 10.07 (s, 1H), 8.99 (d, J = 5.7 Hz, 1H), 8.76 (d, J = 8.4 Hz, 1H), 8.53 (d, J = 1.6 Hz, 1H), 8.44 (d, J = 5.6 Hz, 1H), 8.03 (dd, J = 1.7, 8.5 Hz, 1H), 7.69 (d, J = 1.7 Hz, 1H), 7.55–7.37 (m, 7H), 4.33 (s, 2H), 3.90 (t, J = 6.7 Hz, 2H), 3.45–3.38 (m, 4H), 2.81 (t, J = 6.7 Hz, 2H), 2.11 (quin, J = 6.5 Hz, 2H).

Synthesis and analytical details for **24**

Methyl 5-((3-((tert-butoxycarbonyl)(methyl)amino)propyl)amino)benzo[c][2,6]naphthyridine-8-carboxylate

To a solution of methyl 5-chlorobenzo [*c*] 2,6-naphthyridine-8 carboxylate[14] (500 mg, 1.83 mmol) in DMSO (10 mL) was added DIPEA (473 mg, 3.67 mmol) and tert-butyl (3-aminopropyl)(methyl)carbamate (517 mg, 2.75 mmol) at room temperature. The mixture was heated to 75 °C and stirred for 12 h. The mixture was poured into iced-water (50 mL) and the precipitate was collected by filtration and dried under vacuum to afford the title compound (750 mg, 78% yield) as a red solid.

LCMS (AM3): rt = 0.757 min, (425.2 $[M+H]^+$).

5-((3-((tert-butoxycarbonyl)(methyl)amino)propyl)amino)benzo[c][2,6]naphthyridine-8-carboxylic acid

To a solution of methyl 5-((3-((tert-butoxycarbonyl)(methyl)amino)propyl)amino)benzo[c][2,6] naphthyridine-8-carboxylate

(740 mg, 1.41 mmol) in THF (10 mL), methanol (1 mL) and water (1 mL) was added NaOH (564 mg, 14.1 mmol) at room temperature. The mixture was heated to 50 °C and stirred for 2 h. The reaction mixture was reduced *in vacuo* and purified by reverse-phase HPLC (column: Phenomenex Gemini-NX C18 75 × 30 mm × 3 μm; eluents: A) 0.1% HCl in H_2O (v/v), B) acetonitrile; gradient: 20–80% B, 20 min) to afford the title compound (550 mg, 93% yield) as a red solid.

LCMS (AM3): rt = 0.782 min, (411.4 $[M+H]^+$).

tert-butyl (3-((8-carbamoylbenzo[c][2,6]naphthyridin-5-yl)amino)propyl)(methyl)carbamate

To a solution of 5-((3-((tert-butoxycarbonyl)(methyl)amino)propyl)amino)benzo[c][2,6] naphthyridine-8-carboxylic acid (550 mg, 1.34 mmol) in THF (10 mL) was added DIPEA (519 mg, 4.02 mmol), NH_4Cl (143 mg, 2.68 mmol) and HATU (662 mg, 1.74 mmol) at room temperature and the mixture was stirred for 12 h. The reaction mixture was poured into iced-water (50 mL) and the precipitate was collected by filtration and dried under vacuum to afford the title compound (400 mg, 69% yield) as a red solid.

LCMS (AM3): rt = 0.697 min, (410.3 [M+H]+).

5-((3-(methylamino)propyl)amino)benzo[c][2,6]naphthyridine-8-carboxamide

To a solution of tert-butyl (3-((8-carbamoylbenzo[c][2,6]naphthyridin-5-yl)amino)propyl)(methyl)carbamate (380 mg, 0.920 mmol) in 1,4-dioxane (10 mL) was added a solution of HCl in dioxane (4 M, 5 mL) at 0 °C. The reaction mixture was warmed to room temperature and stirred for 0.5 h. The reaction mixture was reduced *in vacuo* to afford the title compound (400 mg, 97% yield, HCl salt) as a light-yellow solid, which was used directly without further purification.

LCMS (AM5): rt = 0.621 min, (310.2 [M+H]+).

tert-butyl (3-((3-((8-carbamoylbenzo[c][2,6]naphthyridin-5-yl)amino)propyl)(methyl)amino)-3-oxopropyl)((2-chloro-[1,1'-biphenyl]-4-yl)methyl)carbamate

To a solution of 3-((tert-butoxycarbonyl)((2-chloro-[1,1'-biphenyl]-4-yl)methyl)amino)propanoic acid (100 mg, 0.25 mmol) in THF (10 mL) was added HATU (117 mg, 0.310 mmol), 5-((3-(methylamino)propyl)amino)benzo[c][2,6]naphthyridine-8-carboxamide (137 mg, 0.310 mmol, HCl salt) and DIPEA (99 mg, 0.77 mmol) at room

temperature and the mixture was stirred for 12 h. The reaction mixture was reduced *in vacuo* and purified by reverse-phase HPLC (column: Phenomenex Gemini-NX C18 75 × 30 mm × 3 μm; eluents: A) 0.1% TFA in H_2O (v/v), B) acetonitrile; gradient: 20–80% B, 20 min) to afford the title compound (130 mg, 74% yield) as a yellow solid.

LCMS (AM3): rt = 0.913 min, (681.3 $[M+H]^+$).

5-((3-(3-(((2-chloro-[1,1'-biphenyl]-4-yl)methyl)amino)-N-methylpropanamido)propyl)amino)benzo[c][2,6]naphthyridine-8-carboxamide (24)

To a solution of tert-butyl (3-((3-((8-carbamoylbenzo[c][2,6]naphthyridin-5-yl)amino)propyl)(methyl)amino)-3-oxopropyl)((2-chloro-[1,1'-biphenyl]-4-yl)methyl)carbamate (110 mg, 0.160 mmol) in DCM (5 mL) was added TFA (1.54 g, 13.5 mmol) at 0 °C. The reaction mixture was then warmed to room temperature and stirred for 0.5 h. The reaction mixture was reduced *in vacuo* and purified by reverse-phase HPLC (column: Phenomenex Luna C18 150 × 25 mm × 10 μm; eluents: A) 0.075% TFA in H_2O (v/v), B) acetonitrile; gradient: 12–42% B, 9 min) to afford title compound **24** (59.2 mg, 51% yield, TFA salt) as a yellow solid.

LCMS (AM3): rt = 0.750 min, (581.2 $[M+H]^+$).

^{1}H NMR (400 MHz, MeOH-d_4) δ: 10.04 (s, 1H), 8.87 (d, J = 5.6 Hz, 1H), 8.67 (d, J = 8.4 Hz, 1H), 8.27 (d, 1H), 8.19 (s, 1H), 7.85 (d, J = 1.2 Hz, 1H), 7.71 (s, 1H), 7.48-7.41 (m, 7H), 4.25-4.22 (m, 2H), 3.71-3.68 (m, 2H), 3.60-3.56 (m, 2H), 3.24-3.20 (m, 2H), 3.22-3.19 (m, 3H), 2.80 - 2.76 (m, 2H), 2.04-2.00 (m, 2H).

Synthesis and analytical details for **25**

Methyl 5-((2-((tert-butoxycarbonyl)amino)ethyl)(methyl)amino)benzo[c][2,6]naphthyridine-8-carboxylate

A mixture of methyl 5-chlorobenzo [*c*] 2,6-naphthyridine-8 carboxylate[14] (300 mg, 1.03 mmol), tert-butyl N-[2-(methylamino)ethyl] carbamate (269 mg, 1.55 mmol) and DIPEA (359 mL, 2.06 mmol) in DMSO (5 mL) was stirred at 70 °C for 15 h. The reaction mixture was poured into water, and the precipitate was collected by filtration to afford the title compound (300 mg) as a brown solid, which was used in the next step without further purification.

LCMS (AM3): rt = 0.845 min, (411.2 [M+H]+).

5-((2-((tert-Butoxycarbonyl)amino)ethyl)(methyl)amino)benzo[c][2,6]naphthyridine-8-carboxylic acid

To a mixture of methyl 5-((2-((tert-butoxycarbonyl)amino)ethyl)(methyl)amino)benzo[c][2,6] naphthyridine-8-carboxylate (300 mg, 731 mmol) in THF (5 mL), methanol (5 mL) and water (5 mL) was added LiOH monohydrate (61.3 mg, 1.46 mmol) at room temperature and the mixture was stirred for 15 h. The solvent was removed *in vacuo* and the mixture was acidified with aq. HCl (1 M) to pH 4. The resulting precipitate was collected by filtration and dried under vacuum to afford the title compound (250 mg) as a brown solid, which was used in the next step without further purification.

LCMS (AM3): rt =0.777 min, (397.2 $[M+H]^+$).

tert-Butyl (2-((8-carbamoylbenzo[c][2,6]naphthyridin-5-yl)(methyl)amino)ethyl)carbamate

To a mixture of 5-((2-((tert-butoxycarbonyl)amino)ethyl)(methyl)amino)benzo[c][2,6] naphthyridine-8-carboxylic acid (250 mg, 6311 mmol), EDCI (181 mg, 946 mmol), HOBt (128 mg, 946 mmol) and DIPEA (122 mg, 946 mmol) in DMF (5 mL) was added NH_4Cl (169 mg, 3.15 mmol) at room temperature and the mixture was stirred for 15 h. The mixture was poured into water (50 mL) and extracted with a mixed solution of DCM and methanol (50 mL × 2, 10:1). The combined organic phases were washed with brine (50 mL), dried over Na_2SO_4 and concentrated *in vacuo* to afford the title compound (120 mg) as a brown solid, which was used in the next step without further purification.

LCMS (AM3): rt = 0.681 min, (396.2 $[M+H]^+$).

5-((2-Aminoethyl)(methyl)amino)benzo[c][2,6]naphthyridine-8-carboxamide

To a solution of tert-butyl (2-((8-carbamoylbenzo[c][2,6]naphthyridin-5-yl)(methyl)amino)ethyl)carbamate (120 mg, 303 mmol) in 1,4-dioxane (5 mL) was added a solution of HCl in 1,4-dioxane (4 M, 3 mL) at room temperature and the mixture was stirred for 1 h. The mixture was reduced *in vacuo* and the residue was triturated with MTBE (10 mL), filtered and dried under reduced pressure to afford the title compound (100 mg, HCl salt) as a yellow solid which was used in the net step without further purification.

LCMS (AM3): rt = 0.234 min, (296.2 $[M+H]^+$).

4-(((2-Chloro-[1,1'-biphenyl]-4-yl)methyl)amino)butanoic acid

To a solution of 4-aminobutanoic acid (5.71 g, 55.4 mmol) in methanol (30 mL) was added 3-chloro-4-phenylbenzaldehyde[30] (3.00 g, 13.9 mmol) at room temperature. After stirring for 2 h, $NaBH(OAc)_3$ (8.80 g, 41.5 mmol) was added, and the mixture was stirred for 12 h. The

mixture was filtered, and the filtrate was concentrated. The residue purified by reverse-phase HPLC (column: Phenomenex Synergi C18 150 × 25 mm × 10 μm; eluents: A) H_2O, B) acetonitrile; gradient: 10–90% B, 20 min) to afford the title compound (1.9 g, 45% yield) as a white solid.

LCMS (AM3): rt = 0.787 min, (304.5 $[M+H]^+$).

4-((tert-Butoxycarbonyl)((2-chloro-[1,1'-biphenyl]-4-yl)methyl)amino)butanoic acid

To a mixture of 4-(((2-chloro-[1,1'-biphenyl]-4-yl)methyl)amino)butanoic acid (1.50 g, 4.94 mmol) in THF (20 mL) and water (2 mL) was added $NaHCO_3$ (830 mg, 9.88 mmol) and Boc_2O (1.19 g, 5.43 mmol) at room temperature and the mixture was stirred for 2 h. The mixture was concentrated and the residue was purified by reverse-phase HPLC (column: Phenomenex Synergi C18 150 × 25 mm × 10 μm; eluents: A) 0.1% NH_4OH in H_2O (v/v), B) acetonitrile; gradient: 10–90% B, 20 min) to afford the title compound (1.8 g, 90% yield) as a white solid.

LCMS (AM3): rt = 0.962 min, (426.1 $[M+Na]^+$).

tert-Butyl (4-((2-((8-carbamoylbenzo[c][2,6]naphthyridin-5-yl)(methyl)amino)ethyl)amino)-4-oxobutyl)((2-chloro-[1,1'-biphenyl]-4-yl)methyl)carbamate

To a solution of 4-((tert-butoxycarbonyl)((2-chloro-[1,1'-biphenyl]-4-yl)methyl)amino)butanoic acid (121.73 mg, 301.39 mmol) and HATU (172 mg, 452 mmol) in DMF (5 mL) was added DIPEA (105 mL, 603 mmol) followed by 5-((2-zminoethyl)(methyl)amino)benzo[c][2,6]naphthyridine-8-carboxamide (100 mg, 301 mmol) at room temperature and the mixture was stirred for 2 h. The mixture was poured into water (50 mL) and extracted with ethyl acetate (50 mL × 2). The combined organic phases were washed with brine (50 mL), dried (Na_2SO_4) and concentrated to afford the title compound (150 mg) as a yellow solid, that was used in the next step without further purification.

LCMS (AM3): rt=0.909 min, (681.4 $[M+H]^+$).

5-((2-(4-(((2-Chloro-[1,1'-biphenyl]-4-yl)methyl)amino)butanamido)ethyl)(methyl)amino)benzo[c][2,6]naphthyridine-8-carboxamide (25)

To a solution of tert-butyl (4-((2-((8-carbamoylbenzo[c][2,6]naphthyridin-5-yl)(methyl)amino)ethyl)amino)-4-oxobutyl)((2-chloro-[1,1'-biphenyl]-4-yl)methyl)carbamate

(150 mg, 220 mmol) in DCM (5 mL) was added TFA (27 mmol, 2.0 mL) at room temperature and the mixture was stirred for 15 h. The mixture was reduced *in vacuo* and the residue was purified by reverse-phase HPLC (column: Phenomenex Synergi C18 150 × 25 mm × 10 μm; eluents: A) 0.1% TFA in H_2O (v/v), B) acetonitrile; gradient: 18–38% B, 10 min) to afford title compound **25** (87.7 mg, , 68% yield) as an orange solid.

LCMS (AM3): rt = 0.830 min, (581.4 $[M+H]^+$).

^1H NMR (400 MHz, MeOH-d_4) δ: 10.12 (s, 1H), 8.90 (d, *J* = 5.7 Hz, 1H), 8.76 (d, *J* = 8.6 Hz, 1H), 8.58 (d, *J* = 1.5 Hz, 1H), 8.37 (d, *J* = 5.9 Hz, 1H), 8.05 (dd, *J* = 1.8, 8.5 Hz, 1H), 7.68 (d, *J* = 1.6 Hz, 1H), 7.51–7.36 (m, 7H), 4.26 (s, 2H), 3.92 (t, *J* = 6.7 Hz, 2H), 3.68 (t, *J* = 6.7 Hz, 2H), 3.51 (s, 3H), 3.16 (t, *J* = 7.5 Hz, 2H), 2.39 (t, *J* = 6.7 Hz, 2H), 2.02–1.95 (quin, 2H).

Synthesis and analytical details for **26**

4-(((2-Chloro-[1,1'-biphenyl]-4-yl)methyl)(methyl)amino)butanoic acid

To a solution of 4-(((2-chloro-[1,1'-biphenyl]-4-yl)methyl)amino)butanoic acid (350 mg, 1.15 mmol) in methanol (35 mL) was added aqueous formaldehyde (93.5 mg, 1.15 mmol) at room temperature. After stirring for 2 h, $NaBH_3CN$ (724 mg, 11.5 mmol) was added and the mixture was stirred for 12 h. The mixture was filtered and concentrated *in vacuo* to give a residue that was purified by reverse-phase HPLC (column: Phenomenex Gemini-NX C18 75 × 30 mm × 3 μm; eluents: A) 0.1% FA in H_2O (v/v), B) acetonitrile; gradient: 20–80% B, 20 min) to afford the title compound (200 mg, 48% yield) as a colorless oil.

^1H NMR (400 MHz, $CDCl_3$) δ: 7.97–7.79 (m, 1H), 7.57–7.36 (m, 7H), 4.23 (s, 2H), 3.14 (t, *J* = 6.0 Hz, 2H), 2.70 (s, 3H), 2.44 (t, *J* = 6.0 Hz, 2H), 1.97–1.94 (m, 2H).

5-((2-(4-(((2-Chloro-[1,1'-biphenyl]-4-yl)methyl)(methyl)amino)butanamido)ethyl)amino)benzo[c][2,6]naphthyridine-8-carboxamide (26)

To a solution of 4-(((2-chloro-[1,1'-biphenyl]-4-yl)methyl)(methyl)amino)butanoic acid (100 mg, 315 μmol) in THF (5 mL) was added DIPEA (81.3 mg, 629 μmol) and HATU (144 mg, 378 μmol). After stirring for 10 min at room temperature, amine **35** (100 mg, 315 μmol) was added and the mixture was stirred for 12 h. The mixture was filtered, concentrated *in vacuo* and the residue was purified by reverse-phase HPLC (column: Phenomenex Synergi C18 150 × 25 mm × 10 μm; eluents: A) 0.1% TFA in H_2O (v/v), B) acetonitrile; gradient: 18–38% B, 10 min) to afford title compound **26** (137.5 mg, 50% yield) as a yellow solid.

LCMS (AM3): rt = 0.773 min, (581.2 $[M+H]^+$).

^{1}H NMR (400 MHz, DMSO) δ: 10.12 (s, 1H), 9.77 (br s, 1H), 8.92 (d, *J* = 2.4 Hz, 1H), 8.75 (d, *J* = 3.6 Hz, 1H), 8.39–8.17 (m, 4H), 7.89 (d, *J* = 4.4 Hz, 1H), 7.76 (s, 1H), 7.59-7.37 (m, 8H), 4.52–4.43 (m, 1H), 4.35–4.21 (m, 1H), 3.77-3.75 (m, 2H), 3.57-3.42 (m, 2H), 3.17 (br d, *J* = 12.8 Hz, 2H), 2.72–2.71 (m, 3H), 2.27–2.23 (m, 2H), 1.97–1.94 (m, 2H).

Synthesis and analytical details for **27**

4-(((2-chloro-[1,1'-biphenyl]-4-yl)methyl)amino)butan-1-ol

A mixture of 3-chloro-4-phenylbenzaldehyde[30] (10 g, 42.00 mmol), 4-aminobutan-1-ol (8.01 g, 89.9 mmol) and molecular sieves (4 Å, 20 g) in methanol (200 mL) was stirred at room temperature for 18 h. $NaBH_3CN$ (9.00 g, 143 mmol) was added and the mixture was stirred for 4 h at room temperature. The reaction mixture was filtered and reduced *in vacuo* to give a residue that was diluted with water (200 mL) and extracted with ethyl acetate (200 mL × 3). The combined organic phases were washed with brine (300 mL), dried (Na_2SO_4), filtered and reduced *in vacuo*. The crude product was purified by column chromatography on silica gel eluting with DCM/methanol (100:1 → 10:1) to give the title compound (6.9 g, 52% yield) as a light-brown gum.

^{1}H NMR ($CHCl_3$-*d*, 400 MHz) *δ*: 7.37-7.34 (m, 6H), 7.25-7.19 (m, 2H), 3.74 (s, 2H), 3.56 (m, 2H), 3.38 (bs, 2H), 2.68 (t, *J* = 5.6 Hz, 2H), 1.63-1.58 (m, 4H).

tert-butyl ((2-chloro-[1,1'-biphenyl]-4-yl)methyl)(4-hydroxybutyl)carbamate

To a mixture of 4-(((2-chloro-[1,1'-biphenyl]-4-yl)methyl)amino)butan-1-ol (6.90 g, 23.8 mmol) in THF (45 mL) and water (15 mL) was added $NaHCO_3$ (4.00 g, 47.6 mmol) and $(Boc)_2O$ (6.00 g, 27.5 mmol) at room temperature and the mixture was stirred for 18 h. The reaction mixture was diluted with water (100 mL), extracted with ethyl acetate (100 mL × 3) and the combined organic phases washed with brine (100 mL), dried (Na_2SO_4), filtered and reduced *in vacuo*. The residue was purified by column chromatography on silica gel eluting with petroleum ether/ethyl acetate (8:1 → 2:1) to afford the title compound (7.0 g, 75% yield) as a light-brown oil.

LCMS (AM1): rt = 0.839 min, (334.1 $[M-^tBu+2H]^+$).

tert-butyl ((2-chloro-[1,1'-biphenyl]-4-yl)methyl)(4-oxobutyl)carbamate (27)

To a solution of tert-butyl ((2-chloro-[1,1'-biphenyl]-4-yl)methyl)(4-hydroxybutyl)carbamate (1.50 g, 3.85 mmol) in DCM (30 mL) was added (1,1,1-trisacetyloxy)-1,1-dihydro-1,2-

benziodoxol-3(1*H*)-one (DMP, 2.0 g, 4.7 mmol) at room temperature. The reaction mixture was stirred for 15 h, then filtered and the filtrate reduced *in vacuo*. The residue was purified by column chromatography on silica gel eluting with petroleum ether/ethyl acetate (100:1 → 30:1) to afford title compound **27** (1.01 g, 63% yield) as a colourless oil.

LCMS (AM1): rt = 0.979 min, (332.0 $[M\text{-}^{t}Bu+2H]^{+}$).

Synthesis and analytical details for **28**

Methyl 5-((2-((tert-butoxycarbonyl)amino)ethyl)amino)benzo[c][2,6]naphthyridine-8-carboxylate

To a stirred mixture of methyl 5-chlorobenzo [*c*] 2,6-naphthyridine-8 carboxylate[14] (6.00 g, 20.7 mmol) and tert-butyl (2-aminoethyl)carbamate (4.55 mL, 29.06 mmol) in DMSO (60 mL) was added DIPEA (9.0 mL, 52 mmol) at room temperature. The mixture was heated to 70 °C and stirred for 17 h. The reaction mixture was added to water (300 mL), precipitating a grey solid that was collected by filtration and dried under vacuum. This crude product was triturated in ethanol (60 mL), filtered and dried under vacuum to afford the title compound (7.2 g, 88% yield) as a grey solid.

^{1}H NMR (400 MHz, DMSO-d_6) δ: 10.07 (s, 1H), 8.90 (d, J = 5.6 Hz, 1H), 8.76 (d, J = 8.4 Hz, 1H), 8.23 (d, J = 5.6 Hz, 1H), 8.17 (s, 1H), 8.05 (t, J = 4.8 Hz, 1H), 7.83 (dd, J = 8.4 Hz, 1.6 Hz, 1H), 7.03 (t, J = 5.6 Hz, 1H), 3.92 (s, 3H), 3.65 (q, J = 5.6 Hz, 2H), 3.33-3.29 (m, 2H), 1.36 (s, 9H).

Methyl 5-((2-aminoethyl)amino)benzo[c][2,6]naphthyridine-8-carboxylate (28)

A mixture of methyl 5-((2-((tert-butoxycarbonyl)amino)ethyl)amino)benzo[c][2,6]naphthyridine -8-carboxylate (1.11 g, 2.88 mmol) in a solution of HCl in 1,4-dioxane (4 M, 10 mL, 40 mmol) was stirred at room temperature for 5 h. The reaction mixture was reduced *in vacuo* to afford title compound **28** (1.33 g, HCl salt) as a yellow solid, which was used in the next step without further purification.

LCMS (AM1): rt = 0.561 min, (297.0 $[M+H]^{+}$).

Synthesis and analytical details for **29**

Methyl 5-((2-((4-((tert-butoxycarbonyl)((2-chloro-[1,1'-biphenyl]-4-yl)methyl)amino)butyl)amino)ethyl)amino)benzo[c][2,6]naphthyridine-8-carboxylate

To a mixture of aldehyde **27** (1.0 g, 2.4 mmol) and NaOAc (1.06 g, 12.9 mmol) in methanol (20 mL) was added amine **28** (2.23 g, 3.35 mmol, HCl salt) at room temperature and the mixture

was stirred for 1 h. $NaBH_3CN$ (324 mg, 5.16 mmol) was added, and the mixture was stirred for 17 h. The mixture was filtered, reduced *in vacuo* and the residue was purified by reverse-phase HPLC (column: Phenomenex Synergi C18 150 × 25 mm × 10 μm; eluents: A) 0.1% TFA in H_2O (v/v), B) acetonitrile; gradient: 20–40% B, 10 min) to afford the title compound (240 mg, 12% yield, TFA salt) as a yellow solid.

LCMS (AM2): rt = 0.879 min, (668.3 $[M+H]^+$).

Methyl 5-((2-((tert-butoxycarbonyl)(4-((tert-butoxycarbonyl)((2-chloro-[1,1'-biphenyl]-4-yl)methyl)amino)butyl)amino)ethyl)amino)benzo[c][2,6]naphthyridine-8-carboxylate

To a mixture of methyl 5-((2-((4-((tert-butoxycarbonyl)((2-chloro-[1,1'-biphenyl]-4-yl)methyl)amino)butyl)amino)ethyl)amino)benzo[c][2,6]naphthyridine-8-carboxylate (240 mg, 0.298 mmol, TFA salt) in THF (10 mL) was added DMAP (20 mg, 0.16 mmol), Boc_2O (143 mg, 0.653 mmol) and TEA (1.8 mmol, 0.25 mL) at room temperature. The mixture was heated to reflux for 18 h. The mixture was reduced *in vacuo* to afford the title compound (330 mg) as a brown solid, which was used in the next step without further purification.

LCMS (AM1): rt = 0.946 min, (768.3 $[M+H]^+$).

5-((2-((tert-Butoxycarbonyl)(4-((tert-butoxycarbonyl)((2-chloro-[1,1'-biphenyl]-4-yl)methyl)amino)butyl)amino)ethyl)amino)benzo[c][2,6]naphthyridine-8-carboxylic acid (29)

A mixture of methyl 5-((2-((tert-butoxycarbonyl)(4-((tert-butoxycarbonyl)((2-chloro-[1,1'-biphenyl]-4-yl)methyl)amino)butyl)amino)ethyl)amino)benzo[c][2,6]naphthyridine-8-carboxylate (330 mg, 0.430 mmol) and LiOH monohydrate (180 mg, 4.29 mmol) in water (8 mL) and THF (16 mL) was stirred at room temperature for 8 h. The mixture was added to aq. HCl (0.1 N, 50 mL) and extracted with ethyl acetate (20 mL × 3). The combined organic phases were washed with brine (30 mL), dried (Na_2SO_4), filtered and reduced *in vacuo* to afford title compound **29** (200 mg) as a yellow solid, which was used directly without further purification.

LCMS (AM1): rt = 0.890 min, (754.3 $[M+H]^+$).

Synthesis and analytical details for **30**

tert-Butyl (4-((tert-butoxycarbonyl)(2-((8-carbamoylbenzo[c][2,6]naphthyridin-5-yl)amino)ethyl)amino)butyl)((2-chloro-[1,1'-biphenyl]-4-yl)methyl)carbamate

To a mixture of acid **29** (200 mg, 0.270 mmol) in DMF (10 mL) was added NH_4Cl (400 mg, 7.48 mmol), HOBt (160 mg, 1.18 mmol), DIPEA (1 mL) and EDCI (230 mg, 1.20 mmol) at

room temperature. The reaction mixture was heated to 70 °C for 8 h. The mixture was poured into water (100 mL) and extracted with ethyl acetate (30 mL × 2). The combined organic phases were washed with brine (30 mL), dried (Na_2SO_4), filtered and reduced *in vacuo* to afford the title compound (200 mg) as a yellow gum, which was used in the next step without further purification.

LCMS (AM1): rt = 0.877 min, (753.3 $[M+H]^+$).

5-((2-((4-(((2-Chloro-[1,1'-biphenyl]-4-yl)methyl)amino)butyl)amino)ethyl)amino)benzo[c][2,6]naphthyridine-8-carboxamide (30)

A mixture of tert-butyl (4-((tert-butoxycarbonyl)(2-((8-carbamoylbenzo[c][2,6]naphthyridin-5-yl)amino)ethyl)amino)butyl)((2-chloro-[1,1'-biphenyl]-4-yl)methyl)carbamate (160 mg, 0.190 mmol) and TFA (5.0 mL, 68 mmol) in DCM (10 mL) was stirred at room temperature for 2 h. The reaction mixture was reduced *in vacuo* and the residue was purified by reverse-phase HPLC (column: Phenomenex Synergi C18 150 × 25 mm × 10 μm; eluents: A) 0.1% TFA in H_2O (v/v), B) acetonitrile; gradient: 15–45% B, 10 min) to afford title compound **30** (87 mg, 58% yield, TFA salt) as a yellow solid.

LCMS (AM4): rt = 0.588 min, (553.2 $[M+H]^+$).

^1H NMR (400 MHz, MeOH-*d₄*) δ: 10.03 (s, 1H), 8.87 (d, *J* = 5.7 Hz, 1H), 8.70 (d, *J* = 8.6 Hz, 1H), 8.28 (d, *J* = 1.7 Hz, 1H), 8.19 (d, *J* = 5.7 Hz, 1H), 7.90 (dd, *J* = 1.8, 8.4 Hz, 1H), 7.68 (d, *J* = 1.6 Hz, 1H), 7.52-7.48 (m, 1H), 7.48-7.37 (m, 6H), 4.26 (s, 2H), 4.10-4.03 (t, 2H), 3.50-3.44 (t, 2H), 3.30-3.25 (m, 2H), 3.18-3.11 (m, 2H), 1.97-1.80 (m, 4H).

Synthesis and analytical details for **31**

tert-Butyl (4-((tert-butoxycarbonyl)(2-((8-methylcarbamoylbenzo[c][2,6]naphthyridin-5-yl)amino)ethyl)amino)butyl)((2-chloro-[1,1'-biphenyl]-4-yl)methyl)carbamate

To a solution of acid **29** (50 mg, 59 μmol) and HATU (33.3 mg, 58.5 μmol) in DMF (1 mL) was added TEA (17.7 mg, 117 μmol) at room temperature. After stirring for 0.5 h, methanamine hydrochloride (3.91 mg, 58.5 μmol) was added and the mixture was stirred for 0.5 h. The mixture was poured into water (25 mL) and extracted with ethyl acetate (30 mL × 2). The combined organic phases were washed with brine (30 mL), dried (Na_2SO_4), filtered and reduced *in vacuo* to afford the title compound (39 mg) as a white solid, which was used in the next step without further purification.

5-((2-((4-(((2-Chloro-[1,1'-biphenyl]-4-yl)methyl)amino)butyl)amino)ethyl)amino)-N-methylbenzo[c][2,6]naphthyridine-8-carboxamide (31)

To a solution of tert-butyl (4-((tert-butoxycarbonyl)(2-((8-methylcarbamoylbenzo[c][2,6]naphthyridin-5-yl)amino)ethyl)amino)butyl)((2-chloro-[1,1'-biphenyl]-4-yl)methyl)carbamate (39 mg, 43 μmol) in DCM (1 mL) was added TFA (250 μL, 3.38 mmol) and the mixture was stirred at room temperature for 8 h. The reaction mixture was reduced *in vacuo* and the residue was purified by reverse-phase HPLC (column: Phenomenex Synergi C18 150 × 25 mm × 10 μm; eluents: A) 0.1% TFA in H_2O (v/v), B) acetonitrile; gradient: 15–45% B, 10 min) to afford title compound **31** (24.5 mg, 70% yield) as a yellow gum.

LCMS (AM4): rt = 0.619 min, (567.2 $[M+H]^+$).

^{1}H NMR (400 MHz, DMSO) δ: 9.97 (s, 1H), 8.85 (d, *J* = 5.6 Hz, 1H), 8.58 (d, *J* = 9.6 Hz, 1H), 8.27 (d, *J* = 5.6 Hz, 1H), 8.23 (d, *J* = 1.6 Hz, 1H), 7.77 (dd, *J* = 5.6 Hz, 2.0 Hz, 1H), 7.67 (s, 1H), 7.47–7.47 (m, 1H), 7.45–7.38 (m, 6H), 4.25 (s, 2H), 4.08 (t, *J* = 5.6 Hz, 2H), 3.51 (t, *J* = 5.6 Hz, 2H), 3.25 (t, *J* = 7.2 Hz, 2H), 3.15 (t, *J* = 5.6 Hz, 2H), 2.97 (s, 3H), 1.92–1.78 (m, 4H).

Synthesis and analytical details for **32**

tert-Butyl(4-((tert-butoxycarbonyl)(2-((8-((methylsulfonyl)carbamoyl)benzo[c][2,6]naphthyridin-5-yl)amino)ethyl)amino)butyl)((2-chloro-[1,1'-biphenyl]-4-yl)methyl)carbamate

To a solution of acid **29** (70 mg, 93 μmol) in DCM (10 mL) was added DCC (29.0 mg, 141 μmol) followed by methanesulfonamide (11.0 mg, 116 μmol) and DMAP (11 mg, 90 μmol) at room temperature and the mixture was stirred for 20 h. The mixture was poured into water (25 mL) and extracted with DCM (30 mL × 2). The combined organic phases were washed with brine (30 mL), dried (Na_2SO_4), filtered and reduced *in vacuo* to afford the title compound (30 mg, 39% yield) as a yellow solid, which was used in the next step without further purification.

LCMS (AM4): rt = 0.913 min, (831.2 $[M+H]^+$).

5-((2-((4-(((2-Chloro-[1,1'-biphenyl]-4-yl)methyl)amino)butyl)amino)ethyl)amino)-N-(methylsulfonyl)benzo[c][2,6]naphthyridine-8-carboxamide (32)

To a solution of tert-butyl(4-((tert-butoxycarbonyl)(2-((8-((methylsulfonyl)carbamoyl)benzo[c][2,6]naphthyridin-5-yl)amino)ethyl)amino)butyl)((2-chloro-[1,1'-biphenyl]-4-yl)methyl)carbamate (30 mg, 27 μmol) in 1,4-dioxane (5 mL) was

added HCl in 1,4-dioxane (4 M, 5 mL) and the mixture was stirred at room temperature for 2 h. The reaction mixture was reduced *in vacuo* and the residue was purified by reverse-phase HPLC (column: Phenomenex Synergi C18 150 × 25 mm × 10 μm; eluents: A) 0.1% TFA in H_2O (v/v), B) acetonitrile; gradient: 15–45% B, 10 min) to afford title compound **32** (13.9 mg, 75% yield) as a yellow solid.

LCMS (AM4): rt = 0.635 min, (631.2 $[M+H]^+$).

^1H NMR (400 MHz, MeOD) δ: 10.27 (s, 1H), 9.05 (d, *J* = 6.0 Hz, 1H), 8.82-8.78 (m, 2H), 8.48 (s, 1H), 8.01 (d, *J* = 2.0 Hz, 1H), 7.72 (d, *J* = 1.6 Hz, 1H), 7.46 (d, *J* = 6.0 Hz, 1H), 7.44–7.39 (m, 6H), 4.28 (s, 2H), 4.21 (t, *J* = 5.6 Hz, 2H), 3.56 (t, *J* = 5.6 Hz, 2H), 3.43 (s, 3H), 3.32-3.10 (m, 2H), 3.27 (t, *J* = 7.2 Hz, 2H), 1.92–1.89 (m, 4H).

Synthesis and analytical details for **33**

tert-Butyl (4-((tert-butoxycarbonyl)(2-((8-cyanobenzo[c][2,6]naphthyridin-5-yl)amino)ethyl)amino)butyl)((2-chloro-[1,1'-biphenyl]-4-yl)methyl)carbamate

To a solution of tert-butyl (4-((tert-butoxycarbonyl)(2-((8-carbamoylbenzo[c][2,6]naphthyridin-5-yl)amino)ethyl)amino)butyl)((2-chloro-[1,1'-biphenyl]-4-yl)methyl)carbamate (195 mg, 0.260 mmol) and TEA (1 mL) in DCM (20 mL) was added TFAA (0.5 mL) dropwise at room temperature and the mixture was stirred for 12 h. The mixture was reduced *in vacuo* and purified by reverse-phase HPLC (column: Phenomenex Gemini-NX C18 75 × 30 mm × 3 μm; eluents: A) 0.1% NH_4OH in H_2O (v/v), B) acetonitrile; gradient: 20–55% B, 10 min) to afford the title compound (130 mg, 68% yield) as a light-yellow solid.

LCMS (AM1): rt = 1.024 min, (735.3 $[M+H]^+$).

tert-Butyl (4-((2-((8-(2H-tetrazol-5-yl)benzo[c][2,6]naphthyridin-5-yl)amino)ethyl)(tert-butoxycarbonyl)amino)butyl)((2-chloro-[1,1'-biphenyl]-4-yl)methyl)carbamate

To a mixture of tert-butyl (4-((tert-butoxycarbonyl)(2-((8-cyanobenzo[c][2,6]naphthyridin-5-yl)amino)ethyl)amino)butyl)((2-chloro-[1,1'-biphenyl]-4-yl)methyl)carbamate (95 mg, 0.12 mmol) and NH_4Cl (66 mg, 1.2 mmol) in DMF (10 mL) was added sodium azide (220 mg, 3.38 mmol) and the reaction mixture was heated to 100 °C and stirred for 23 h. The mixture was diluted with water (60 mL), adjusted to pH 8 with saturated aq. $NaHCO_3$ solution and extracted with ethyl acetate (30 mL × 2). The combined organic phases were washed with brine (30 mL), dried (Na_2SO_4), filtered and reduced *in vacuo*. The residue was purified by reverse-phase

HPLC (column: Phenomenex Synergi C18 150 × 25 mm × 10 μm; eluents: A) 0.05% HCl in H_2O (v/v), B) acetonitrile; gradient: 45−65% B, 10 min) to afford the title compound (60 mg, 63% yield) as a yellow solid.

LCMS (AM1): rt = 0.918 min, (778.3 $[M+H]^+$).

N^1-(2-((8-(2H-tetrazol-5-yl)benzo[c][2,6]naphthyridin-5-yl)amino)ethyl)-N^4-((2-chloro-[1,1'-biphenyl]-4-yl)methyl)butane-1,4-diamine (33)

A mixture of tert-butyl (4-((2-((8-(2H-tetrazol-5-yl)benzo[c][2,6]naphthyridin-5-yl)amino)ethyl)(tert-butoxycarbonyl)amino)butyl)((2-chloro-[1,1'-biphenyl]-4-yl)methyl)carbamate (55 mg, 0.071 mmol) in a solution of HCl in 1,4-dioxane (4 M, 4 mL) was stirred at room temperature for 2 h. The mixture was reduced *in vacuo* and the residue was purified by reverse-phase HPLC (column: Phenomenex Synergi C18 150 × 25 mm × 10 μm; eluents: A) 0.05% HCl in H_2O (v/v), B) acetonitrile; gradient: 12–32% B, 9 min) to afford title compound **33** (10.4 mg, 22% yield, 2 × HCl salt) as a yellow solid.

LCMS (AM1): rt = 0.631 min, (578.2 $[M+H]^+$).

^{1}H NMR (400 MHz, MeOH-d_4) δ:10.22 (s, 1H), 8.99 (d, J = 6.0 Hz, 1H), 8.87 (d, J = 8.8 Hz, 1H), 8.65–8.62 (m, 2H), 8.14 (dd, J = 8.4 Hz, 1.6 Hz, 1H), 7.71 (d, J =1.6 Hz, 1H), 7.54–7.51 (m, 1H), 7.48–7.39 (m, 6H), 4.28 (s, 2H), 4.19 (t, J = 5.2 Hz, 2H), 3.56 (t, J = 5.6 Hz, 2H), 3.29–3.25 (m, 2H), 3.20 (t, J = 6.8 Hz, 2H), 1.96–1.90 (m, 4H).

Synthesis and analytical details for **35**

tert-Butyl (2-(((2-chloro-[1,1'-biphenyl]-4-yl)methyl)amino)ethyl)carbamate

A solution of tert-butyl N-(2-aminoethyl)carbamate (2.96 g, 18.5 mmol) and 3-chloro-4-phenylbenzaldehyde[30] (1.0 g, 4.6 mmol) in methanol (30 mL) was stirred at room temperature for 2 h, then $NaBH(OAc)_3$ (2.93 g, 13.9 mmol) was added and the mixture was stirred for 12 h. The mixture was concentrated to give a residue which was purified by reverse-phase HPLC (column: Phenomenex Synergi C18 150 × 25 mm × 10 μm; eluents: A) 0.1% FA in H_2O (v/v), B) acetonitrile; gradient: 10–90% B, 20 min) to give the title compound (1.1 g, 59% yield, FA salt) as a white solid.

LCMS (AM3): rt = 0.767 min, (361.2 $[M+H]^+$).

(9H-Fluoren-9-yl)methyl (2-((tert-butoxycarbonyl)amino)ethyl)((2-chloro-[1,1'-biphenyl]-4-yl)methyl)carbamate

To a solution of tert-butyl (2-(((2-chloro-[1,1'-biphenyl]-4-yl)methyl)amino)ethyl)carbamate (1.1 g, 2.7 mmol) and DIPEA (942 mL, 5.41 mmol) in DCM (10 mL) was added (9H-fluoren-9-yl)methyl chloroformate (839 mg, 3.24 mmol) at room temperature and the mixture was stirred for 1 h. The reaction mixture was reduced *in vacuo* and the residue purified by reverse-phase HPLC (column: Phenomenex Synergi C18 150 × 25 mm × 10 µm; eluents: A) 0.1% FA in H_2O (v/v), B) acetonitrile; gradient: 10–90% B, 20 min) to afford the title compound (1.4 g, 80% yield) as a white solid.

LCMS (AM3): rt = 1.113 min, (483.1 $[M\text{-}tBuCO_2+2H]^+$).

(9H-Fluoren-9-yl)methyl (2-aminoethyl)((2-chloro-[1,1'-biphenyl]-4-yl)methyl)carbamate

To a solution of (9H-fluoren-9-yl)methyl (2-((tert-butoxycarbonyl)amino)ethyl)((2-chloro-[1,1'-biphenyl]-4-yl)methyl)carbamate (1.4 g, 2.2 mmol) in 1,4-dioxane (6 mL) was added a solution of HCl in 1,4-dioxane (4 M, 6 mL) at room temperature and the mixture was stirred for 1 h. The mixture was reduced *in vacuo* and the residue purified by reverse-phase HPLC (column: Phenomenex Synergi C18 150 × 25 mm × 10 µm; eluents: A) 0.1% NH_4OH in H_2O (v/v), B) acetonitrile; gradient: 10–90% B, 20 min) to give the title compound (1 g, 93% yield) as a white solid.

LCMS (AM3): rt = 0.842 min, (483.1 $[M+H]^+$).

5-((2-((tert-Butoxycarbonyl)amino)ethyl)amino)benzo[c][2,6]naphthyridine-8-carboxylic acid

A mixture of methyl 5-((2-((tert-butoxycarbonyl)amino)ethyl)amino)benzo[c][2,6] naphthyridine-8-carboxylate (7.2 g, 18 mmol) and LiOH monohydrate (7.62 g, 182 mmol) in THF (100 mL) and water (50 mL) was stirred at room temperature for 19 h. The reaction mixture was added to water (200 mL) and the pH of the mixture adjusted to pH 4 with aq. HCl (1 N). The precipitate was collected by filtration and dried under vacuum to afford the title compound (6.44 g, 91% yield) as a light-yellow solid.

^1H NMR (400 MHz, DMSO-d_6) δ: 13.14 (br s, 1H), 10.10 (s, 1H), 8.93 (d, J = 5.2 Hz, 1H), 8.78 (d, J = 8.4 Hz, 1H), 8.32 (br s, 2H), 7.87 (d, J = 1.2 Hz, 1H), 7.07 (s, 1H), 3.69 (m, 2H), 3.36-3.34 (m, 2H), 1.35 (s, 9H).

tert-butyl (2-((8-carbamoylbenzo[c][2,6]naphthyridin-5-yl)amino)ethyl)carbamate

To a mixture of 5-((2-((tert-butoxycarbonyl)amino)ethyl)amino)benzo[c][2,6]naphthyridine-8-carboxylic acid (6.44 g, 16.8 mmol), NH_4Cl (9.00 g, 168 mmol), HOBt (4.55 g, 33.7 mmol) and DIPEA (14.6 mL) in DMF (150 mL) was added EDCI (6.46 g, 33.7 mmol) at room temperature. The reaction mixture was heated to 70 °C and stirred for 3 h. The mixture was added to water (600 mL) and the resulting white precipitate was collected by filtration. The solid was triturated with ethyl acetate:methanol (400 mL, 20:1) and dried under vacuum to afford the title compound (6.39 g, 100% yield) as a white solid.

^{1}H NMR (400 MHz, DMSO-d_6) δ: 10.07 (s, 1H), 8.87 (d, J = 5.2 Hz, 1H), 8.70 (d, J = 8.4 Hz, 1H), 8.22 (d, J = 6.0 Hz, 1H), 8.15 (s, 1H), 8.14 (d, J = 1.6 Hz, 1H), 7.95-7.91 (m, 1H), 7.81 (dd, J = 8.4 Hz, 1.6 Hz, 1H), 7.42 (s, 1H), 7.03 (t, J = 5.2 Hz, 1H), 3.67-3.63 (m, 2H), 3.35-3.33 (m, 2H), 1.37 (s, 9H).

5-((2-Aminoethyl)amino)benzo[c][2,6]naphthyridine-8-carboxamide (35)

A mixture of tert-butyl (2-((8-carbamoylbenzo[c][2,6]naphthyridin-5-yl)amino)ethyl)carbamate (14.4 g, 37.8 mmol) and TFA (1.35 mol, 100 mL) in DCM (50 mL) was stirred at room temperature for 18 h. The reaction mixture was reduced *in vacuo* to afford title compound **35** (21.2 g, TFA salt) as a yellow solid, which was used in the next step without further purification.

LCMS (AM3): rt = 0.666 min, (282.0 $[M+H]^+$).

Synthesis and analytical details for **36**

5-((2-(3-(2-(((2-Chloro-[1,1'-biphenyl]-4-yl)methyl)amino)ethyl)ureido)ethyl)amino)benzo[c][2,6]naphthyridine-8-carboxamide (36)

To a solution of (9H-fluoren-9-yl)methyl (2-aminoethyl)((2-chloro-[1,1'-biphenyl]-4-yl)methyl)carbamate (130 mg, 269 mmol) and TEA (269 mmol, 38 mL) in DMF (5 mL) was added CDI (43.6 mg, 269 mmol) at room temperature under nitrogen. The mixture was stirred for 1 h and then a solution of amine **35** (94.1 mg, 296 mmol) in DMF (0.5 mL) was added. The reaction mixture was stirred at room temperature for 12 h. The mixture was reduced *in vacuo* and the residue purified by reverse-phase HPLC (column: Phenomenex Luna C18 150 × 25 mm × 10 μm; eluents: A) 0.075% TFA in H_2O (v/v), B) acetonitrile; gradient: 10–40% B, 12 min) to afford title compound **36** (21 mg, 10% yield, TFA salt) as a yellow solid.

LCMS (AM3): rt = 0.715 min, (568.1 $[M+H]^+$).

^{1}H NMR (400 MHz, MeOH-d_4) δ: 10.04 (s, 1H), 8.96 (d, J = 5.2 Hz, 1H), 8.73(t, J = 7.2 Hz, 1H), 8.59 (s, 1H), 8.30 (d, J = 5.6 Hz, 1H), 8.02 (d, J = 8.4 Hz, 1H), 7.66 (s, 1H), 7.50–7.25 (m, 7H), 4.36 (s, 2H), 3.89 (t, J = 6.4 Hz, 2H), 3.64 (t, J = 5.6 Hz, 2H), 3.56 (t, J = 6.4 Hz, 2H), 3.35 (t, J = 5.6 Hz, 2H).

Synthesis and analytical details for **37**

tert-Butyl (3-(((2-chloro-[1,1'-biphenyl]-4-yl)methyl)amino)propyl)carbamate

A solution of tert-butyl N-(3-aminopropyl)carbamate (3.22 g, 18.5 mmol) and 3-chloro-4-phenylbenzaldehyde[30] (1.0 g, 4.6 mmol) in methanol (30 mL) was stirred at room temperature for 2 h, then $NaBH(OAc)_3$ (2.93 g, 13.9 mmol) was added and the mixture was stirred for 12 h. The mixture was acidified with TFA (2 mL) and reduced *in vacuo* to give the title compound (2.2 g, TFA salt) as a yellow solid, which was used in the next step without further purification.

(9H-Fluoren-9-yl)methyl (3-((tert-butoxycarbonyl)amino)propyl)((2-chloro-[1,1'-biphenyl]-4-yl)methyl)carbamate

To a solution of tert-butyl (3-(((2-chloro-[1,1'-biphenyl]-4-yl)methyl)amino)propyl)carbamate (2.2 g, 4.5 mmol) and DIPEA (13.5 mmol, 2.35 mL) in DCM (20 mL) was added (9H-fluoren-9-yl) methyl chloroformate (1.40 g, 5.40 mmol) at room temperature and the mixture was stirred for 1 h. The mixture was reduced *in vacuo* and the residue was purified by reverse-phase HPLC (column: Phenomenex Synergi C18 150 × 25 mm × 10 µm; eluents: A) 0.1% TFA in H_2O (v/v), B) acetonitrile; gradient: 10–90% B, 20 min) to afford the title compound (2.6 g, 81% yield) as a white solid.

LCMS (AM3): rt = 1.217 min, (597.2 $[M+H]^+$).

(9H-Fluoren-9-yl) methyl (3-aminopropyl)((2-chloro-[1,1'-biphenyl]-4-yl)methyl)carbamate

To a solution of (9H-fluoren-9-yl)methyl (3-((tert-butoxycarbonyl)amino)propyl)((2-chloro-[1,1'-biphenyl]-4-yl)methyl)carbamate (2.60 g, 3.66 mmol) in 1,4-dioxane (20 mL) was added a solution of HCl in 1,4-dioxane (4 M, 10 mL) at room temperature and the mixture was stirred for 1 h. The mixture was reduced *in vacuo* to give the title compound (1.8 g, 99% yield), which was used in the next step without further purification.

9H-Fluoren-9-ylmethyl N-[3-[2-(tert-butoxycarbonylamino)ethylsulfonylamino]propyl]-N-[(3-chloro-4-phenyl-phenyl)methyl]carbamate

To a solution of (9H-fluoren-9-yl) methyl (3-aminopropyl)((2-chloro-[1,1'-biphenyl]-4-yl)methyl)carbamate (1.0 g, 2.0 mmol) and DIPEA (4.02 mmol, 701 mL) in DMF (15 mL) was added tert-butyl (2-(chlorosulfonyl)ethyl)carbamate (588 mg, 2.41 mmol) at 15 °C and the mixture was stirred for 0.5 h. The mixture was poured into water (100 mL) and extracted with ethyl acetate (100 mL × 2). The combined organic layers were washed with brine (100 mL), dried (Na_2SO_4) and reduced *in vacuo*. The residue was purified by column chromatography on silica gel eluting with petroleum ether/ethyl acetate (2:1) to afford the title compound (250 mg, 18% yield) as a colourless oil.

LCMS (AM3): rt = 1.158 min, (604.3 $[M\text{-}tBuCO_2\text{+}2H]^+$).

(9H-Fluoren-9-yl)methyl (3-(2-aminoethylsulfonamido)propyl)((2-chloro-[1,1'-biphenyl]-4-yl)methyl)carbamate (37)

To a solution of 9H-fluoren-9-ylmethyl N-[3-[2-(tert-butoxycarbonylamino)ethylsulfonylamino]propyl]-N-[(3-chloro-4-phenyl-phenyl)methyl]carbamate (250 mg, 355 mmol) in 1,4-dioxane (10 mL) was added a solution of HCl in 1,4-dioxane (4 M, 5 mL) at room temperature and the mixture was stirred for 1 h. The mixture was reduced *in vacuo* to give a residue that was purified by reverse-phase HPLC (column: Phenomenex Synergi C18 150 × 25 mm × 10 μm; eluents: A) 0.1% NH_4OH in H_2O (v/v), B) acetonitrile; gradient: 10–90% B, 20 min) to afford title compound **37** (100 mg, 47% yield) as a white solid.

LCMS (AM3): rt = 0.910 min, (604.3 $[M+H]^+$).

Synthesis and analytical details for **38**

Methyl 5-((2-(N-(3-(((2-chloro-[1,1'-biphenyl]-4-yl)methyl)amino)propyl)sulfamoyl)ethyl)amino)benzo[c][2,6]naphthyridine-8-carboxylate

A solution of methyl 5-chlorobenzo [*c*] 2,6-naphthyridine-8 carboxylate[14] (40.0 mg, 147 mmol), amine **37** (93.1 mg, 154 mmol) and DIPEA (293 mmol, 51.1 mL) in DMSO (5 mL) was stirred at 80 °C for 12 h. The mixture was poured into water (50 mL) and extracted with ethyl acetate (50 mL × 2). The combined organic phases were washed with brine (50 mL), dried (Na_2SO_4) and reduced *in vacuo*. The residue was purified by reverse-phase HPLC (column: Phenomenex Luna C18 250 × 50 mm × 10 μm; eluents: A) 0.1% TFA in H_2O (v/v), B)

acetonitrile; gradient: 22−52% B, 10 min) to afford the title compound (100 mg, 93% yield, TFA salt) as a yellow solid.

LCMS (AM3): rt = 0.840 min, (618.0 $[M+H]^+$).

5-((2-(N-(3-(((2-chloro-[1,1'-biphenyl]-4-yl)methyl)amino)propyl)sulfamoyl)ethyl)amino)benzo[c][2,6]naphthyridine-8-carboxamide (38)

A solution of methyl 5-((2-(N-(3-(((2-chloro-[1,1'-biphenyl]-4-yl)methyl)amino)propyl)sulfamoyl)ethyl)amino)benzo[c][2,6]naphthyridine-8-carboxylate (100 mg, 137 mmol) in saturated ammonia in methanol (7 M, 10 mL) was stirred at 80 °C for 14 h in a sealed tube. The mixture was reduced *in vacuo* and the residue was purified by reverse-phase HPLC (column: Phenomenex Synergi C18 150 × 25 mm × 10 µm; eluents: A) 0.1% TFA in H_2O (v/v), B) acetonitrile; gradient: 21−51% B, 10 min) to afford title compound **38** (16.8 mg, 17% yield, TFA salt) as a yellow solid.

LCMS (AM3): rt = 0.774 min, (603.1 $[M+H]^+$).

^1H NMR (400 MHz, MeOH-d_4) δ: 10.00 (s, 1H), 8.85 (d, J = 6.0 Hz, 1H), 8.67 (d, J = 8.4 Hz, 1H), 8.29 (d, J = 1.7 Hz, 1H), 8.17 (d, J = 5.6 Hz, 1H), 7.90 (dd, J = 1.8, 8.4 Hz, 1H), 7.67 (d, J = 2.0 Hz, 1H), 7.50–7.37 (m, 7H), 4.28 (s, 2H), 4.17 (t, J = 7.0 Hz, 2H), 3.62 (t, J = 7.0 Hz, 2H), 3.35 (t, J = 6.8 Hz, 2H), 3.26 (t, J = 8.0 Hz, 2H), 2.09–2.02 (quin, 2H).

Synthesis and analytical details for **39**

tert-Butyl (4-(cyanomethoxy)butyl)carbamate

To a mixture of tert-butyl (4-hydroxybutyl)carbamate (7.0 g, 37 mmol) and 2-bromoacetonitrile (8.87 g, 74.0 mmol) in DCM (100 mL) was added silver(I)oxide (18.55 g, 80.05 mmol) and TBAI (2.94 g, 7.96 mmol) at room temperature and the mixture was stirred for 16 h. The mixture was filtered, the filtrate washed with aq. $NaHCO_3$ (100 mL), dried (Na_2SO_4), filtered and concentrated *in vacuo*. The residue was purified by column chromatography on silica gel eluting with petroleum ether/ethyl acetate (10:1) to afford the title compound (1.0 g, 12% yield) as a yellow oil.

^1H NMR (CDCl3, 400 MHz) δ: 4.56 (br s, 1H), 4.24 (s, 2H), 3.62-3.59 (t, 2H), 3.18-3.13 (m, 2H), 1.71-1.63 (m, 2H), 1.58-1.53 (m, 2H), 1.45 (s, 9H) .

tert-Butyl (4-(2-aminoethoxy)butyl)carbamate (39)

To a solution of tert-butyl (4-(cyanomethoxy)butyl)carbamate (1.0 g, 4.4 mmol) in methanol (10 mL) was added ammonium hydroxide (2 mL, 25% wt.) and Raney nickel (100 mg, 1.17 mmol) under nitrogen protection at room temperature. The suspension was degassed under vacuum and purged with hydrogen three times. The mixture was stirred under hydrogen (45 psi) at room temperature for 16 h. The mixture was filtered and the filtrate concentrated *in vacuo* to afford title compound **39** (1 g) as a green oil.

Synthesis and analytical details for **41**

5-((2-(4-((tert-Butoxycarbonyl)amino)butoxy)ethyl)amino)benzo[c][2,6]naphthyridine-8-carboxylic acid

To a solution of ester **51** (5.00 g, 10.7 mmol) in THF (15 mL), methanol (15 mL) and water (15 mL) was added sodium hydroxide (854 mg, 21.3 mmol) at room temperature and the mixture was stirred for 4 h. The organic solvents were concentrated *in vacuo* and the remaining aqueous solution was acidified with aq. HCl (1 M) to pH 5. The resulting precipitate was collected by filtration and dried under vacuum to afford the title compound (4.5 g) as a brown solid.

LCMS (AM3): rt = 0.808 min, (455.3 $[M+H]^+$).

tert-Butyl (4-(2-((8-carbamoylbenzo[c][2,6]naphthyridin-5-yl)amino)ethoxy)butyl) carbamate

To a stirred solution of 5-((2-(4-((tert-butoxycarbonyl)amino)butoxy)ethyl)amino) benzo[c][2,6]naphthyridine-8-carboxylic acid (4.5 g, 9.9 mmol) in DMF (25 mL) was added EDCI (2.85 g, 14.9 mmol), HOBt (2.01 g, 14.9 mmol), DIPEA (1.92 g, 14.9 mmol) and NH_4Cl (2.12 g, 39.6 mmol) at room temperature and the mixture was stirred for 3 h. The reaction mixture was diluted with water (100 mL) and extracted with ethyl acetate (100 mL × 2). The combined organic layers were washed with brine (80 mL × 2), dried (Na_2SO_4), filtered and concentrated *in vacuo*. The residue was purified by reverse-phase HPLC (column: Phenomenex Synergi C18 150 × 25 mm × 10 μm; eluents: A) 0.05% HCl in H_2O (v/v), B) acetonitrile; gradient: 55–75% B, 12 min) to afford the title compound (3.8 g, 68% yield, TFA salt) as a yellow oil.

LCMS (AM3): rt = 0.758 min, (454.4 $[M+H]^+$).

5-((2-(4-Aminobutoxy)ethyl)amino)benzo[c][2,6]naphthyridine-8-carboxamide (41)

To a solution of tert-butyl (4-(2-((8-carbamoylbenzo[c][2,6]naphthyridin-5-yl)amino)ethoxy)butyl) carbamate (3.8 g, 8.4 mmol) in methanol (5 mL) was added a solution

of HCl in methanol (4 M, 2.09 mL) dropwise at 0 °C. The reaction mixture was then warmed to room temperature and stirred for 2 h. The mixture was concentrated *in vacuo* to afford title compound **41** (2.8 g, 86% yield, HCl salt) as a yellow solid.

LCMS (AM3): rt = 0.229 min, (354.1 $[M+H]^+$).

Synthesis and analytical details for **42**

Methyl 5-((3-(3-aminopropoxy)propyl)amino)benzo[c][2,6]naphthyridine-8-carboxylate (42)

A mixture of 3,3'-oxybis(propan-1-amine) (**40**) (1.37 g, 10.3 mmol), methyl 5-chlorobenzo [c] 2,6-naphthyridine-8 carboxylate[14] (600 mg, 2.07 mmol) and DIPEA (535 mg, 4.14 mmol) in DMSO (6 mL) was heated to 65 °C and stirred for 16 h. The mixture was poured into water and extracted with ethyl acetate (50 mL × 2). The combined organic phases were washed with brine (50 mL), dried (Na_2SO_4), filtered and reduced *in vacuo*. The residue was purified by reverse-phase HPLC (column: Phenomenex Synergi C18 150 × 25 mm × 10 μm; eluents: A) 0.1% FA in H_2O (v/v), B) acetonitrile; gradient: 10–90% B, 20 min) to afford title compound **42** (500 mg, 62% yield) as a brown oil.

LCMS (AM3): rt = 0.686 min, (369.1 $[M+H]^+$).

Synthesis and analytical details for **43**

tert-Butyl (4-(2-(((2-chloro-[1,1'-biphenyl]-4-yl)methyl)amino)ethoxy)butyl)carbamate

A mixture of 3-chloro-4-phenylbenzaldehyde[30] (480 mg, 2.22 mmol) and amine **39** (640 mg, 2.75 mmol) in methanol (10 mL) was stirred at room temperature for 6 h. $NaBH(OAc)_3$ (2.82 g, 13.3 mmol) was added, and the mixture was stirred for 12 h. The mixture was reduced *in vacuo* to give a residue, which was purified by reverse-phase HPLC (column: Phenomenex Gemini-NX C18 75 × 30 mm × 3 μm; eluents: A) 0.1% FA in H_2O (v/v), B) acetonitrile; gradient: 20–55% B, 15 min) to afford the title compound (730 mg, 76% yield, FA salt) as a white solid.

LCMS (AM3): rt = 0.865 min, (433.5 $[M+H]^+$).

(9H-Fluoren-9-yl)methyl (2-(4-((tert-butoxycarbonyl)amino)butoxy)ethyl)((2-chloro-[1,1'-biphenyl]-4-yl)methyl)carbamate

To a mixture of tert-butyl (4-(2-(((2-chloro-[1,1'-biphenyl]-4-yl)methyl)amino)ethoxy)butyl)carbamate (600 mg, 1.39 mmol) and $NaHCO_3$ (233 mg, 2.77

mmol) in dioxane (5 mL) and water (5 mL) was added (9H-fluoren-9-yl)methyl carbonochloridate (396 mg, 1.53 mmol) at 0 °C. The mixture was warmed to room temperature and stirred for 1 h. The mixture was diluted with water (20 mL) and extracted with ethyl acetate (20 mL × 3). The combined organic phases were washed with brine (50 mL), dried (Na_2SO_4), filtered and reduced *in vacuo*. The residue was purified by column chromatography on silica gel eluting with petroleum ether/ethyl acetate (1:1) to afford the title compound (800 mg, 88% yield) as a colourless oil.

LCMS (AM3): rt = 1.152 min, (655.4 $[M+H]^+$).

(9H-Fluoren-9-yl)methyl(2-(4-aminobutoxy)ethyl)((2-chloro-[1,1'-biphenyl]-4-yl)methyl)carbamate (43)

A mixture of (9H-fluoren-9-yl)methyl (2-(4-((tert-butoxycarbonyl)amino)butoxy)ethyl)((2-chloro-[1,1'-biphenyl]-4-yl)methyl)carbamate (800 mg, 1.22 mmol) in a solution of HCl in 1,4-dioxane (4 M, 6 mL) was stirred at room temperature for 1 h. The reaction mixture was reduced *in vacuo* to afford title compound **43** (700 mg) as a colourless oil, which was used in the next step without further purification.

LCMS (AM3): rt = 0.886 min, (555.3 $[M+H]^+$).

Synthesis and analytical details for **44**

5-((2-(4-(((2-Chloro-[1,1'-biphenyl]-4-yl)methyl)amino)butoxy)ethyl)amino)benzo[c][2,6] naphthyridine-8-carboxamide (44)

A mixture of amine **41** (150 mg, 0.385 mmol, HCl salt), DIPEA (44.5 mg, 0.344 mmol) and 3-chloro-4-phenylbenzaldehyde[30] (83 mg, 0.38 mmol) in methanol (4 mL) was stirred at room temperature for 12 h, then $NaBH_3CN$ (75 mg, 1.2 mmol) was added and the mixture was stirred for 3 h. The mixture was concentrated *in vacuo* and purified by reverse-phase HPLC (column: Phenomenex Luna C18 250 × 50 mm × 10 μm; eluents: A) 0.1% TFA in H_2O (v/v), B) acetonitrile; gradient: 15–45% B, 10 min) to afford title compound **44** (63.4 mg, 25% yield, TFA salt) as a yellow solid.

LCMS (AM3): rt = 0.786 min, (554.1 $[M+H]^+$).

^{1}H NMR (400 MHz, MeOD) δ: 10.07 (s, 1H), 8.98 (d, J = 5.6 Hz, 1H), 8.77 (d, J = 8.8 Hz, 1H), 8.43-8.37 (m, 2H), 8.03 (dd, J = 8.4, 1.6 Hz, 1H), 7.63 (d, J = 1.6 Hz, 1H), 7.46-7.35 (m, 7H), 4.19 (s, 2H), 4.07 (t, J = 5.2 Hz, 2H), 3.92-3.87 (t, 2H), 3.63 (t, J = 6.0 Hz, 2H), 3.12-3.05 (m, 2H), 1.88-1.77 (m, 2H), 1.75-1.64 (m, 2H).

Synthesis and analytical details for **45**

Methyl 5-((3-(3-(((2-chloro-[1,1'-biphenyl]-4-yl)methyl)amino)propoxy)propyl)amino)benzo[c][2,6]naphthyridine-8-carboxylate

A mixture of amine **42** (440 mg, 1.13 mmol) and 3-chloro-4-phenylbenzaldehyde[30] (220 mg, 1.02 mmol) in methanol (2 mL) was stirred at room temperature for 3 h, then $NaBH_3CN$ (128 mg, 2.03 mmol) was added and the mixture was stirred for 12 h. The mixture was reduced *in vacuo* and purified by reverse-phase HPLC (column: Phenomenex Synergi C18 150 × 25 mm × 10 μm; eluents: A) 0.1% TFA in H_2O (v/v), B) acetonitrile; gradient: 10–90% B, 20 min) to afford the title compound (120 mg, 16% yield, TFA salt) as a yellow solid.

LCMS (AM3): rt = 0.813 min, (569.2 $[M+H]^+$).

Methyl 5-((3-(3-((tert-butoxycarbonyl)((2-chloro-[1,1'-biphenyl]-4-yl)methyl)amino)propoxy)propyl)amino)benzo[c][2,6]naphthyridine-8-carboxylate

To a mixture of methyl 5-((3-(3-(((2-chloro-[1,1'-biphenyl]-4-yl)methyl)amino)propoxy)propyl)amino)benzo[c][2,6]naphthyridine-8-carboxylate (170 mg, 0.299 mmol, TFA salt) and TEA (90.7 mg, 0.896 mmol) in DCM (10 mL) was added Boc_2O (78 mg, 0.36 mmol) at room temperature and the mixture was stirred for 1 h. The mixture was diluted with water (30 mL) and extracted with DCM (30 mL × 2). The combined organic phases were washed with brine (50 mL), dried (Na_2SO_4), filtered and reduced *in vacuo* to afford the title compound (130 mg, 65% yield) as a yellow oil, which was used in the next step without further purification.

LCMS (AM3): rt = 0.942 min, (669.3 $[M+H]^+$).

5-((3-(3-((tert-Butoxycarbonyl)((2-chloro-[1,1'-biphenyl]-4-yl)methyl)amino)propoxy)propyl)amino)benzo[c][2,6]naphthyridine-8-carboxylic acid

To a mixture of methyl 5-((3-(3-((tert-butoxycarbonyl)((2-chloro-[1,1'-biphenyl]-4-yl)methyl)amino)propoxy)propyl)amino)benzo[c][2,6]naphthyridine-8-carboxylate (130 mg, 0.194 mmol) in THF (9 mL), methanol (1 mL) and water (1 mL) was added LiOH monohydrate (81.5 mg, 1.94 mmol) at room temperature and the mixture was stirred for 20 h. The organic solvent was removed *in vacuo*. The remaining aq. phase was acidified with HCl (1 M, 5 mL) and extracted with ethyl acetate (15 mL × 2). The combined organic phases were washed with brine (20 mL), dried (Na_2SO_4), filtered and reduced *in vacuo* to afford the title compound (130 mg) as a yellow oil, which was used directly without further purification.

LCMS (AM3): rt = 0.865 min, (655.3 [M+H]$^+$).

tert-Butyl(3-(3-((8-carbamoylbenzo[c][2,6]naphthyridin-5-yl)amino)propoxy)propyl)((2-chloro-[1,1'-biphenyl]-4-yl)methyl)carbamate

To a mixture of 5-((3-(3-((tert-butoxycarbonyl)((2-chloro-[1,1'-biphenyl]-4-yl)methyl)amino)propoxy)propyl)amino)benzo[c][2,6]naphthyridine-8-carboxylic acid (120 mg, 0.183 mmol) in THF (5 mL) was added HATU (90 mg, 0.24 mmol), DIPEA (74.2 mg, 0.574 mmol) and NH_4Cl (50 mg, 0.94 mmol) at room temperature and the mixture was stirred for 14 h. The mixture was poured into water (20 mL) and the aq. phase was extracted with ethyl acetate (20 mL × 2). The combined organic phases were washed with brine (30 mL), dried (Na_2SO_4), filtered and reduced *in vacuo* to afford the title compound (130 mg) as a yellow oil, which was used in the next step without further purification.

LCMS (AM3): rt = 0.865 min, (654.3 [M+H]$^+$).

5-((3-(3-(((2-Chloro-[1,1'-biphenyl]-4-yl)methyl)amino)propoxy)propyl)amino)benzo[c][2,6]naphthyridine-8-carboxamide (45)

To a mixture of tert-butyl(3-(3-((8-carbamoylbenzo[c][2,6]naphthyridin-5-yl)amino)propoxy)propyl)((2-chloro-[1,1'-biphenyl]-4-yl)methyl)carbamate (125 mg, 0.191 mmol) in DCM (5 mL) was added TFA (1.54 g, 13.5 mmol) at 0 °C. The mixture was warmed to room temperature and stirred for 1 h. The mixture was reduced *in vacuo* and purified by reverse-phase HPLC (column: Phenomenex Synergi C18 150 × 25 mm × 10 µm; eluents: A) 0.1% TFA in H_2O (v/v), B) acetonitrile; gradient: 15–45% B, 10 min) to afford title compound **45** (70.7 mg, 55% yield, TFA salt) as a yellow solid.

LCMS (AM3): rt = 0.773 min, (554.1 [M+H]$^+$).

^{1}H NMR (400 MHz, DMSO-d_6) δ: 10.13 (s, 1H), 9.02-8.88 (m, 3H), 8.78 (d, J = 8.4 Hz, 1H), 8.42-8.38 (d, 1H), 8.37-8.33 (s, 1H), 8.25-8.20 (s, 1H), 7.92 (d, J = 8.4 Hz, 1H), 7.72 (s, 1H), 7.60-7.35 (m, 8H), 4.25-4.20 (m, 2H), 3.75-3.70 (m, 2H), 3.59-3.55 (t, 2H), 3.52-3.48 (t, 2H), 3.10-3.00 (m, 2H), 2.05-1.95 (m, 2H), 1.95-1.85 (m, 2H).

Synthesis and analytical details for **46**

Methyl 5-((4-(2-(((2-chloro-[1,1'-biphenyl]-4-yl)methyl)amino)ethoxy)butyl)amino)benzo[c][2,6]naphthyridine-8-carboxylate

To a mixture of amine **43** (650 mg, 1.17 mmol) and methyl 5-chlorobenzo [c] 2,6-naphthyridine-8 carboxylate[14] (363 mg, 1.17 mmol) in DMSO (4 mL) was added DIPEA (482 mg, 3.73 mmol) at room temperature. The mixture was heated to 70 °C and stirred for 12 h. The reaction mixture was filtered, and the filtrate was reduced *in vacuo*. The residue was purified by reverse-phase HPLC (column: Phenomenex Gemini-NX C18 75 × 30 mm × 3 μm; eluents: A) 0.1% TFA in H_2O (v/v), B) acetonitrile; gradient: 20–55% B, 15 min) to afford the title compound (350 mg, 52% yield) as a yellow oil.

LCMS (AM3): rt = 0.745 min, (569.1 $[M+H]^+$).

5-((4-(2-(((2-Chloro-[1,1'-biphenyl]-4-yl)methyl)amino)ethoxy)butyl)amino)benzo[c][2,6]naphthyridine-8-carboxamide (46)

A mixture of methyl 5-((4-(2-(((2-chloro-[1,1'-biphenyl]-4-yl)methyl)amino)ethoxy)butyl)amino)benzo[c][2,6]naphthyridine-8-carboxylate (300 mg, 0.517 mmol) in a solution of ammonia in methanol (7 M, 10 mL) was heated to 80 °C and stirred for 16 h in an autoclave. The mixture was reduced *in vacuo* and the residue was purified by reverse-phase HPLC (column: Phenomenex Luna C18 150 × 25 mm × 10 μm; eluents: A) 0.1% TFA in H_2O (v/v), B) acetonitrile; gradient: 10–40% B, 10 min) to give title compound **46** (208 mg, 60% yield, TFA salt) as a yellow solid.

LCMS (AM3): rt = 0.754 min, (554.3 $[M+H]^+$).

^{1}H NMR (400 MHz, DMSO-d_6) δ: 10.12 (s, 1H), 9.04 (br s, 2H), 8.94 (d, J = 5.6 Hz, 1H), 8.77 (d, J = 8.4 Hz, 1H), 8.40 (s, 1H), 8.33 (br s, 1H), 8.22 (s, 1H), 7.90 (d, J = 7.8 Hz, 1H), 7.71 (d, J = 1.6 Hz, 1H), 7.55 (s, 1H), 7.51-7.38 (m, 7H), 4.22 (s, 2H), 3.76-3.64 (m, 4H), 3.53 (t, J = 6.4 Hz, 2H), 3.21-3.09 (m, 2H), 1.89-1.76 (m, 2H), 1.75-1.62 (m, 2H).

Synthesis and analytical details for **48**

4-(2-Chloro-[1,1'-biphenyl]-4-carboxamido)butanoic acid

To a mixture of 3-chloro-4-phenyl-benzoic acid (200 mg, 860 μmol) and HATU (327 mg, 860 μmol) in DCM (10 mL) was added TEA (222 mg, 2.20 mmol) and the mixture stirred at 0 °C for 4.5 h. 4-aminobutanoic acid (133 mg, 1.29 mmol) was added and the mixture was stirred at room temperature for 0.5 h. The mixture was poured into water (5 mL) and extracted with ethyl acetate (20 mL × 3). The combined organic phases were washed with brine (10 mL × 2), dried (Na_2SO_4), filtered and concentrated *in vacuo* to afford the title compound (60 mg, 20% yield) as a white solid.

LCMS (AM4): rt = 0.887 min, (318.1 $[M+H]^+$).

5-((2-(4-(2-Chloro-[1,1'-biphenyl]-4-carboxamido)butanamido)ethyl)amino)benzo[c][2,6]naphthyridine-8-carboxamide (48)

To a mixture of 4-(2-chloro-[1,1'-biphenyl]-4-carboxamido)butanoic acid (60.0 mg, 172 μmol), amine **35** (71.0 mg, 223 μmol) and TEA (104 mg, 1.03 mmol) was added T_3P (82.0 mg, 258 μmol, 50% wt in ethyl acetate) in DCM (10 mL). The resulting mixture was stirred at room temperature for 12 h. The mixture was reduced *in vacuo* and the residue was purified by reverse-phase HPLC (column: Phenomenex Synergi C18 150 × 25 mm × 10 μm; eluents: A) 0.1% TFA in H_2O (v/v), B) acetonitrile; gradient: 15–45% B, 10 min) to afford title compound **48** (25.85 mg, 26% yield) as a yellow solid.

LCMS (AM4): rt = 0.713 min, (581.3 $[M+H]^+$).

^{1}H NMR (400 MHz, DMSO) δ: 10.11 (s, 1H), 8.93 (d, *J* = 6.4Hz, 1H), 8.78 (d, *J* = 8.0Hz, 1H), 8.63 (t, *J* = 6.4, 1H), 8.32 (d, *J* =1.6, 2H), 8.29 (d, *J* =1.6, 2H), 7.97 (d, *J* = 1.6, 1H), 7.89 (d, *J* = 4.4Hz, 1H), 7.81-7.76 (m, 1H), 7.49–7.42 (m, 7H), 3.73 (t, *J* = 5.2Hz, 2H), 3.29-3.27 (m, 2H), 2.52–2.51 (m, 2H), 2.19 (t, *J* = 7.2Hz, 2 H), 1.78 (t, *J* = 7.2Hz, 2 H).

Synthesis and analytical details for **49**

4-(((2-Chloro-[1,1'-biphenyl]-4-yl)methyl)amino)-4-oxobutanoic acid

To a mixture of (2-chloro-[1,1'-biphenyl]-4-yl)methanamine (250 mg, 1.15 mmol) and dihydrofuran-2,5-dione (172 mg, 1.72 mmol) in THF (5 mL) was added TEA (232 mg, 2.30 mmol) and the mixture was stirred at room temperature for 1 h. The mixture was filtered, concentrated *in vacuo* and the residue was purified by reverse-phase HPLC (column: Phenomenex Synergi C18 150 × 25 mm × 10 μm; eluents: A) 0.1% TFA in H_2O (v/v), B) acetonitrile; gradient: 15–45% B, 10 min) to afford the title compound (300 mg, 70% yield) as a white solid.

LCMS (AM4): rt = 0.888 min, (317.9 $[M+H]^+$).

N^1-(2-((8-carbamoylbenzo[c][2,6]naphthyridin-5-yl)amino)ethyl)-N^4-((2-chloro-[1,1'-biphenyl]-4-yl)methyl)succinamide (49)

To a mixture of amine **35** (150 mg, 472 μmol) and 4-(((2-chloro-[1,1'-biphenyl]-4-yl)methyl)amino)-4-oxobutanoic acid (175 mg, 472 μmol) in THF (2 mL) was added HATU (215 mg, 566 umol) and DIPEA (183 mg, 1.42 mmol) and the mixture was stirred at room

temperature for 2 h. The mixture was filtered, concentrated *in vacuo* and the residue was purified by reverse-phase HPLC (column: Phenomenex Synergi C18 150 × 25 mm × 10 μm; eluents: A) 0.1% TFA in H_2O (v/v), B) acetonitrile; gradient: 15–45% B, 10 min) to afford title compound **49** (137.5 mg, 50% yield) as a yellow solid.

LCMS (AM4): rt = 0.805 min, (581.1 $[M+H]^+$).

^{1}H NMR (400 MHz, DMSO) δ: 10.12 (s, 1H), 8.93 (d, *J* = 2.8 Hz, 1H), 8.78 (d, *J* = 4.4 Hz, 1H), 8.44 (t, *J* = 4.0 Hz, 1H), 8.44 (t, *J* = 5.2 Hz, 2H), 8.33–8.23 (m, 2H), 7.92 (d, *J* = 6.0 Hz, 1H), 7.56 (s, 1H), 7.42-7.23 (m, 8H), 4.25 (d, *J* = 2.8 Hz, 2H), 3.87-3.75 (m, 2H), 3.37-3.18 (m, 2H), 2.49–2.38 (m, 4H).

Synthesis and analytical details for **50**

(2-Chloro-[1,1'-biphenyl]-4-yl)methanol

To a solution of 3-chloro-4-phenylbenzaldehyde[30] (220 mg, 1.02 mmol) (2.0 g, 9.2 mmol) in methanol (20 mL) was added $NaBH_4$ (1.77 g, 46.8 mmol) slowly at 0 °C. The reaction mixture was warmed to room temperature and stirred for 1 h. The mixture was filtered, diluted with water (10 mL) and extracted with ethyl acetate (20 mL × 3). The combined organic layers were washed with brine (30 mL × 2), dried (Na_2SO_4), filtered and concentrated *in vacuo.* The residue was purified by column chromatography on silica gel eluting with petroleum ether/ethyl acetate (100:1 → 1:1) to afford the title compound (1.4 g, 69% yield) as a colorless oil.

LCMS (AM4): rt = 0.910 min, (201.1 $[M+H-OH]^+$).

tert-Butyl 4-((2-chloro-[1,1'-biphenyl]-4-yl)methoxy)butanoate

To a solution of (2-chloro-[1,1'-biphenyl]-4-yl)methanol (1 g, 4.57 mmol) in acetonitrile (20 mL) was added tert-butyl 4-bromobutanoate (2.04 g, 9.15 mmol), TBAI (365 mg, 988 μmol) and silver(I)oxide (2.29 g, 9.88 mmol) and the mixture was stirred at 50 °C for 12 h. The mixture was filtered, concentrated *in vacuo*, and the residue was purified by column chromatography on silica gel eluting with petroleum ether/ethyl acetate (50:1 → 2:1) to afford the title compound (400 mg, 24% yield) as a colorless oil.

^{1}H NMR (400 MHz, $CDCl_3$) δ: 7.40–7.35 (m, 8H), 5.17–5.06 (m, 2H), 4.17–3.94 (m, 2H), 2.31–2.29 (m, 2H), 2.04–1.98 (m, 2H), 1.40 (s, 9H).

4-((2-Chloro-[1,1'-biphenyl]-4-yl)methoxy)butanoic acid

To a solution of tert-butyl 4-((2-chloro-[1,1'-biphenyl]-4-yl)methoxy)butanoate (350 mg, 970 μmol) in DCM (5 mL) was added TFA (6.16 g, 54.0 mmol) and the mixture was stirred at room temperature for 1 h. The mixture was concentrated *in vacuo* to afford the title compound (130 mg, 44% yield) as a brown oil.

^{1}H NMR (400 MHz, DMSO) δ: 12.07 (br s, 1H), 7.49–7.37 (m, 8H), 4.50 (s, 2H), 3.48 (t, *J* = 6.4 Hz, 2H), 2.32 (t, *J* = 7.2 Hz, 2H), 1.83–1.77 (m, 2H).

5-((2-(4-((2-Chloro-[1,1'-biphenyl]-4-yl)methoxy)butanamido)ethyl)amino)benzo[c][2,6]naphthyridine-8-carboxamide (50)

To a solution of 4-((2-chloro-[1,1'-biphenyl]-4-yl)methoxy)butanoic acid (50.0 mg, 164 μmol) in THF (2 mL) was added HATU (74.9 mg, 197 μmol) and DIPEA (84.8 mg, 656 μmol). After stirring at room temperature for 15 min, amine **35** (64.9 mg, 164 μmol, TFA) was added and the mixture was stirred for 12 h. The mixture was filtered, concentrated *in vacuo* and the residue was purified by reverse-phase HPLC (column: Phenomenex Synergi C18 150 × 25 mm × 10 μm; eluents: A) 0.1% TFA in H_2O (v/v), B) acetonitrile; gradient: 15–45% B, 10 min) to afford title compound **50** (15.1 mg, 16% yield) as a yellow solid.

LCMS (AM4): rt = 0.828 min, (568.3 $[M+H]^+$).

^{1}H NMR (400 MHz, MeOD) δ: 10.07 (s, 1H), 8.97 (d, *J* = 6.8 Hz, 1H), 8.78 (d, *J* = 4.0 Hz, 1H), 8.56 (s, 1H), 8.22 (d, *J* = 2.8 Hz, 1H), 8.05 (d, *J* = 3.2 Hz, 1H), 7.35-7.32 (m, 4H), 7.21-7.20 (m, 3H), 7.19-7.12 (m, 1H), 4.49 (s, 2H), 3.82 (t, *J* = 3.2 Hz, 2H), 3.64 (t, *J* = 2.8 Hz, 2H), 3.55 (t, *J* = 3.2 Hz, 2H), 2.48 (t, *J* = 3.6 Hz, 2H), 2.10–2.05 (m, 2H).

Synthesis and analytical details for **51**

Methyl 5-((2-(4-((tert-butoxycarbonyl)amino)butoxy)ethyl)amino)benzo[c][2,6] naphthyridine-8-carboxylate (51)

To a solution of methyl 5-chlorobenzo [c] 2,6-naphthyridine-8 carboxylate[14] (3.20 g, 11.7 mmol) in DMSO (50 mL) was added DIPEA (3.03 g, 23.5 mmol) and amine **39** (3.0 g, 13 mmol) at room temperature. The reaction mixture was heated to 75 °C and stirred for 12 h. The mixture was diluted with water (100 mL) and extracted with ethyl acetate (100 mL × 2). The combined organic layers were washed with brine (100 mL), dried (Na_2SO_4), filtered and concentrated *in vacuo* to afford title compound **51** (5 g) as a brown solid.

LCMS (AM3): rt = 0.841 min, (469.3 $[M+H]^+$).

Synthesis and analytical details for **52**

5-((2-(4-Aminobutoxy)ethyl)amino)benzo[c][2,6]naphthyridine-8-carboxylic acid (52)

5-((2-(4-((tert-Butoxycarbonyl)amino)butoxy)ethyl)amino)benzo[c][2,6]naphthyridine-8-carboxylic acid (4.0 g, 8.8 mmol) was treated with a solution of HCl in 1,4-dioxane (4 M, 40 mL) and stirred at room temperature for 16 h. The precipitate was collected by filtration and dried under vacuum to afford title compound **52** (2.5 g, HCl salt) as a yellow solid.

LCMS (AM3): rt = 0.501 min, (354.9 $[M+H]^+$).

Synthesis and analytical details for **53**

Methyl 5-((2-(4-aminobutoxy)ethyl)amino)benzo[c][2,6]naphthyridine-8-carboxylate (53)

To solution of methyl 5-((2-(4-((tert-butoxycarbonyl)amino)butoxy)ethyl)amino)benzo[c][2,6] naphthyridine-8-carboxylate (**51**) (200 mg, 427 mmol) in 1,4-dioxane (5 mL) was added a solution of HCl in 1,4-dioxane (4 M, 5 mL) at room temperature and the mixture was stirred for 1 h. The mixture was concentrated *in vacuo* to afford title compound **53** (201 mg, HCl salt) as a yellow oil, which was used directly without purification.

LCMS (AM3): rt = 0.673 min, (369.2 $[M+H]^+$).

General Procedure for the Synthesis of **54a–m** and **55a–b**.

To a solution of the respective amine (**41**, **52** or **53**) (1 eq.) and substituted benzaldehyde (1 eq.) in methanol (1–15 mL) was added either DIPEA (1.5–3 eq.) or sodium acetate (2–3 eq.) at room temperature and the mixture stirred for 2–16 h. $NaBH(OAc)_3$ (3–4 eq.) or $NaBH_3CN$ (1–3 eq.) was added and the mixture was stirred at room temperature for 0.5–12 h. The reaction mixture was concentrated *in vacuo* and the residue was purified by the method indicated to afford the title compound**.**

Synthesis and analytical details for **54a-m**

5-((2-(4-((3-Chlorobenzyl)amino)butoxy)ethyl)amino)benzo[c][2,6]naphthyridine-8-carboxamide (54a)

Reactants: amine **41** and 3-chlorobenzaldehyde

Product purified by reverse-phase HPLC (column: Phenomenex Luna C18 150 × 25 mm × 10 μm; eluents: A) 0.075% TFA in H_2O (v/v), B) acetonitrile; gradient: 5–35% B, 9 min) to afford title compound **54a** (28.33 mg, 25% yield, TFA salt) as a yellow solid.

LCMS (AM3): rt = 0.683 min, (478.0 $[M+H]^+$).

^{1}H NMR (400MHz, MeOH-d_4) δ: 10.04 (s, 1H), 8.92 (d, J = 5.6 Hz, 1H), 8.73 (d, J = 8.6 Hz, 1H), 8.34 (d, J = 1.7 Hz, 1H), 8.29 (d, J = 5.6 Hz, 1H), 7.96 (dd, J = 1.7, 8.3 Hz, 1H), 7.50 (s, 1H), 7.46-7.34 (m, 3H), 4.13 (s, 2H), 4.03-4.01 (t, 2H), 3.88-3.86 (m, 2H), 3.62 (t, J = 6.0 Hz, 2H), 3.07-3.03 (m, 2H), 1.83-1.75 (quintet, 2H), 1.71-1.63 (m, 2H).

5-((2-(4-((3-Chloro-4-cyclopropylbenzyl)amino)butoxy)ethyl)amino)benzo[c][2,6]naphthyridine-8-carboxamide (54b)

Reactants: amine **41** and 3-chloro-4-cyclopropylbenzaldehyde

Product purified by reverse-phase HPLC (column: Phenomenex Synergi C18 150 × 25 mm × 10 µm; eluents: A) 0.1% TFA in H_2O (v/v), B) acetonitrile; gradient: 22−42% B, 10 min) to afford title compound **54b** (20.5 mg, 22% yield, TFA salt) as a yellow solid.

LCMS (AM3): rt = 0.727 min, (518.1 $[M+H]^+$).

^{1}H NMR (400 MHz, MeOH-d_4) δ: 10.06 (s, 1H), 8.95 (d, J = 5.8 Hz, 1H), 8.76 (d, J = 8.5 Hz, 1H), 8.37 (d, J = 1.8 Hz, 1H), 8.33 (d, J = 5.8 Hz, 1H), 8.00 (dd, J = 1.6, 8.4 Hz, 1H), 7.47 (d, J = 2.0 Hz, 1H), 7.26 (dd, J = 1.9, 7.9 Hz, 1H), 7.03 (d, J = 8.0 Hz, 1H), 4.07 (s, 2H), 4.04 (t, J = 5.3 Hz, 2H), 3.90–3.86 (t, 2H), 3.61 (t, J = 6.0 Hz, 2H), 3.05–2.99 (m, 2H), 2.23–2.15 (m, 1H), 1.84–1.74 (m, 2H), 1.72–1.62 (m, 2H), 1.08–1.01 (m, 2H), 0.71–0.66 (m, 2H).

5-((2-(4-((3-Chloro-4-cyclopropoxybenzyl)amino)butoxy)ethyl)amino)benzo[c][2,6]naphthyridine-8-carboxamide (54c)

Reactants: amine **41** and 3-chloro-4-cyclopropoxybenzaldehyde

Product purified by reverse-phase HPLC (column: Phenomenex Synergi C18 150 × 25 mm × 10 µm; eluents: A) 0.1% TFA in H_2O (v/v), B) acetonitrile; gradient: 18−38% B, 10 min) to afford title compound **54c** (46.2 mg, 48% yield, TFA salt) as a yellow solid.

LCMS (AM3): rt = 0.737 min, (534.1 $[M+H]^+$).

^{1}H NMR (400 MHz, MeOH-d_4) δ: 10.06 (s, 1H), 8.95 (d, J = 5.6 Hz, 1H), 8.76 (d, J = 8.6 Hz, 1H), 8.37-8.36 (d, 1H), 8.33-8.31 (d, 1H), 7.99 (dd, J = 1.7, 8.3 Hz, 1H), 7.46 (d, J = 2.2 Hz, 1H), 7.43-7.41 (m, 1H), 7.36-7.35 (m, 1H), 4.05-4.03 (m, 4H), 3.91-3.84 (m, 3H), 3.62 (t, J =

6.0 Hz, 2H), 3.05-2.98 (m, 2H), 1.84-1.74 (m, 2H), 1.74-1.64 (m, 2H), 0.89-0.81 (m, 2H), 0.75-0.70 (m, 2H).

5-((2-(4-((3-Chloro-4-(trifluoromethyl)benzyl)amino)butoxy)ethyl)amino)benzo[c][2,6]naphthyridine-8-carboxamide (54d)

Reactants: amine **41** and 3-chloro-4-(trifluoromethyl)benzaldehyde

Product purified by reverse-phase HPLC (column: Phenomenex Synergi C18 150 × 25 mm × 10 μm; eluents: A) 0.1% TFA in H_2O (v/v), B) acetonitrile; gradient: 20–40% B, 10 min) to afford title compound **54d** (22 mg, 22% yield, TFA) as a yellow solid.

LCMS (AM3): rt = 0.755 min, (546.4 $[M+H]^+$).

^{1}H NMR (400 MHz, MeOH-*d*$_4$) δ: 10.08 (s, 1H), 8.97 (d, *J* = 5.7 Hz, 1H), 8.77 (d, *J* = 8.6 Hz, 1H), 8.38-8.37 (m, 1H), 8.35 (d, *J* = 5.5 Hz, 1H), 8.01 (dd, *J* = 1.8, 8.5 Hz, 1H), 7.85 (d, *J* = 8.2 Hz, 1H), 7.76 (s, 1H), 7.58 (d, *J* = 8.1 Hz, 1H), 4.24 (s, 2H), 4.07-4.05 (t, 2H), 3.90-3.88 (t, 2H), 3.64-3.61 (t, 2H), 3.10-3.06 (dd, 2H), 1.87-1.79 (m, 2H), 1.71-1.63 (m, 2H).

5-((2-(4-((3-Chloro-4-(trifluoromethoxy)benzyl)amino)butoxy)ethyl)amino)benzo[c][2,6]naphthyridine-8-carboxamide (54e)

Reactants: amine **41** and 3-chloro-4-(trifluoromethoxy)benzaldehyde

Product purified by reverse-phase HPLC (column: Phenomenex Luna C18 150 × 25 mm × 10 μm; eluents: A) 0.075% TFA in H_2O (v/v), B) acetonitrile; gradient: 12–42% B, 9 min) to afford title compound **54e** (28.3 mg, 25% yield, TFA salt) as a yellow solid.

LCMS (AM3): rt = 0.739 min, (562.0 $[M+H]^+$).

^{1}H NMR (400MHz, MeOH-*d*$_4$) δ: 10.06 (s, 1H), 8.96 (d, *J* = 5.7 Hz, 1H), 8.75 (d, *J* = 8.6 Hz, 1H), 8.39 (d, 1H), 8.36-8.35 (d, 1H), 8.00 (dd, *J* = 1.7, 8.4 Hz, 1H), 7.73 (s, 1H), 7.51 (s, 2H), 4.18 (s, 2H), 4.05 (t, *J* = 5.1 Hz, 2H), 3.90-3.88 (t, 2H), 3.61 (t, *J* = 6.1 Hz, 2H), 3.09-3.05 (t, 2H), 1.85-1.75 (quintet, 2H), 1.73-1.62 (quintet, 2H).

5-((2-(4-((3-Fluoro-4-(trifluoromethoxy)benzyl)amino)butoxy)ethyl)amino)benzo[c][2,6]naphthyridine-8-carboxamide (54f)

Reactants: amine **41** and 3-fluoro-4-(trifluoromethoxy)benzaldehyde

Product purified by reverse-phase HPLC (column: Phenomenex Synergi C18 150 × 25 mm × 10 µm; eluents: A) 0.1% TFA in H_2O (v/v), B) acetonitrile; gradient: 20–40% B, 10 min) to afford title compound **54f** (35 mg, 35% yield, TFA salt) as a yellow gum.

LCMS (AM3): rt = 0.746 min, (546.4 $[M+H]^+$).

^{1}H NMR (400 MHz, MeOH-*d$_4$*) δ: 10.07 (s, 1H), 8.95 (d, *J* = 5.5 Hz, 1H), 8.77 (d, *J* = 8.4 Hz, 1H), 8.37 (d, *J* = 1.6 Hz, 1H), 8.34-8.32 (d, 1H), 8.00 (dd, *J* = 1.7, 8.5 Hz, 1H), 7.55-7.47 (m, 2H), 7.38-7.36 (m, 1H), 4.19 (s, 2H), 4.06-4.03 (t, 2H), 3.90-3.87 (t, 2H), 3.62 (t, *J* = 6.1 Hz, 2H), 3.09-3.05 (dd, 2H), 1.85-1.77 (m, 2H), 1.70-1.64 (quintet, 2H).

5-((2-(4-((3-Cyano-4-(trifluoromethoxy)benzyl)amino)butoxy)ethyl)amino)benzo[c][2,6]naphthyridine-8-carboxamide (54g)

Reactants: amine **41** and 5-formyl-2-(trifluoromethoxy)benzonitrile

Product purified by reverse-phase HPLC (column: Phenomenex Synergi C18 150 × 25 mm × 10 µm; eluents: A) 0.1% TFA in H_2O (v/v), B) acetonitrile; gradient: 20–40% B, 10 min) to afford title compound **54g** (19.3 mg, 16% yield, TFA salt) as a yellow oil.

LCMS (AM3): rt = 0.715 min, (553.1 $[M+H]^+$).

^{1}H NMR (400 MHz, MeOH-*d$_4$*) δ: 10.06 (s, 1H), 8.94 (d, *J* = 4.6 Hz, 1H), 8.76 (d, *J* = 8.8 Hz, 1H), 8.35 (s, 1H), 8.31-8.29 (d, 1H), 7.99 (d, *J* = 2.2 Hz, 2H), 7.90 (d, *J* = 8.8 Hz, 1H), 7.65 (d, *J* = 8.6 Hz, 1H), 4.25 (s, 2H), 4.04 (t, *J* = 4.8 Hz, 2H), 3.90-3.87 (t, 2H), 3.63 (t, *J* = 6.0 Hz, 2H), 3.11-3.07 (dd, 2H), 1.85-1.78 (quintet, 2H), 1.72-1.63 (quintet, 2H).

5-((2-(4-((3-Cyano-5-(trifluoromethoxy)benzyl)amino)butoxy)ethyl)amino)benzo[c][2,6]naphthyridine-8-carboxamide (54h)

Reactants: amine **41** and 3-formyl-5-(trifluoromethoxy)benzonitrile

Product purified by reverse-phase HPLC (column: Waters Xbridge 150 × 25 mm × 5 µm; eluents: A) 0.05% NH_4OH in H_2O, B) acetonitrile; gradient: 28–58% B, 10 min) to afford title compound **54h** (40.8 mg, 14% yield) as a white solid.

LCMS (AM3): rt = 0.748 min, (553.2 $[M+H]^+$).

1H NMR (400 MHz, MeOH-d4) δ: 9.87 (s, 1H), 8.74 (d, J = 5.6 Hz, 1H), 8.55 (d, J = 8.4 Hz, 1H), 8.17 (d, J = 1.6 Hz, 1H), 8.09 (d, J = 5.6 Hz, 1H), 7.80 (dd, J = 8.4 Hz, 1.6 Hz, 1H), 7.65

(s, 1H), 7.55 (s, 2H), 3.89 (t, J = 5.6 Hz, 2H), 3.80 (t, J = 5.6 Hz, 2H), 3.73 (s, 2H), 3.57 (t, J = 6.0 Hz, 2H), 2.54 (t, J = 6.8 Hz, 2H), 1.65-1.55 (m, 4H).

5-((2-(4-((3-(Cyanomethyl)-5-(trifluoromethoxy)benzyl)amino)butoxy)ethyl)amino)benzo[c][2,6]naphthyridine-8-carboxamide (54i)

Reactants: amine **41** and 2-(3-formyl-5-(trifluoromethoxy)phenyl)acetonitrile[36]

Product purified by reverse-phase HPLC (column: Phenomenex Synergi C18 150 × 25 mm × 10 μm; eluents: A) 0.225% FA in H_2O (v/v), B) acetonitrile; gradient: 19–49% B, 10 min) to afford title compound **54i** (34.5 mg, 27% yield) as a yellow oil.

LCMS (AM3): rt = 0.713 min, (567.3 $[M+H]^+$).

^{1}H NMR (400 MHz, MeOH-d_4) δ: 9.91 (s, 1H), 8.77 (d, *J* = 5.6 Hz, 1H), 8.59 (d, *J* = 8.8 Hz, 1H), 8.46 (br s, 1H), 8.19 (d, *J* = 2.0 Hz, 1H), 8.12 (d, *J* = 5.6 Hz, 1H), 7.82 (dd, *J* = 8.4, 2.0 Hz, 1H), 7.45 (s, 1H), 7.37-7.36 (m, 2H), 4.12 (s, 2H), 4.00 (s, 2H), 3.91 (t, *J* = 5.6 Hz, 2H), 3.82 (t, *J* = 5.6 Hz, 2H), 3.62 (t, *J* = 6.0 Hz, 2H), 3.04 (t, *J* = 7.6 Hz, 2H), 1.84-1.76 (m, 2H), 1.72-1.66 (m, 2H).

5-((2-(4-((3-(2-Hydroxyethoxy)-5-(trifluoromethoxy)benzyl)amino)butoxy)ethyl)amino)benzo[c][2,6]naphthyridine-8-carboxamide (54j)

Reactants: amine **41** and 3-(2-hydroxyethoxy)-5-(trifluoromethoxy)benzaldehyde[36]

Product purified by reverse-phase HPLC (column: Phenomenex Gemini-NX C18 75 × 30 mm × 3 μm; eluents: A) 0.225% FA in H_2O (v/v), B) acetonitrile; gradient: 8–28% B, 7 min) to afford title compound **54j** (85.9 mg, 26% yield, TFA salt) as a yellow gum.

LCMS (AM3): rt = 0.729 min, (588.3 $[M+H]^+$).

^{1}H NMR (400 MHz, MeOH-d_4) δ: 9.94 (s, 1H), 8.78 (d, *J* = 5.6 Hz, 1H), 8.61 (d, *J* = 8.4 Hz, 1H), 8.47 (s, 1H), 8.21 (d, *J* = 1.6 Hz, 1H), 8.13 (dd, *J* = 5.6 Hz, 0.8 Hz, 1H), 7.82 (dd, *J* = 8.4 Hz, 2.0 Hz, 1H), 7.01 (d, *J* = 2.0 Hz, 1H), 6.94 (s, 1H), 6.91 (s, 1H), 4.08-4.06 (m, 4H), 3.92 (t, *J* = 5.6 Hz, 2H), 3.87 (J = 5.6 Hz, 2H), 3.82 (t, *J* = 5.6 Hz, 2H), 3.62 (t, *J* = 6.0 Hz, 2H), 3.03 (t, *J* = 6.8 Hz, 2H), 1.84-1.76 (quin, 2H), 1.72-1.66 (quin, 2H).

5-((2-(4-((3-Carbamoyl-5-(trifluoromethoxy)benzyl)amino)butoxy)ethyl)amino)benzo[c][2,6]naphthyridine-8-carboxamide (54k)

Reactants: amine **41** and 3-formyl-5-(trifluoromethoxy)benzamide[36]

Product purified by reverse-phase HPLC (column: Phenomenex Gemini-NX C18 75 × 30 mm × 3 μm; eluents: A) 0.05% NH_4OH in H_2O (v/v), B) acetonitrile; gradient: 13–43% B, 7 min) to afford title compound **54k** (82.6 mg, 26% yield) as a white solid.

LCMS (AM7): rt = 0.844 min, (571.2 $[M+H]^+$).

^{1}H NMR (400 MHz, MeOH-d_4) δ: 9.92 (s, 1H), 8.76 (d, *J* = 6.0 Hz, 1H), 8.60 (d, *J* = 8.4 Hz, 1H), 8.20 (d, *J* = 1.6 Hz, 1H), 8.13 (d, *J* = 5.6 Hz, 1H), 7.82 (dd, *J* = 8.4, 1.6 Hz, 1H), 7.79 (s, 1H), 7.66 (s, 1H), 7.43 (s, 1H), 3.88 (t, *J* = 5.6 Hz, 2H), 3.80 (t, *J* = 5.6 Hz, 2H), 3.74 (s, 2H), 3.56 (t, *J* = 5.6 Hz, 2H), 2.56 (t, *J* = 7.2 Hz, 2H), 1.64-1.56 (m, 4H).

5-((2-(4-((3-(Oxazol-5-ylmethyl)-5-(trifluoromethoxy)benzyl)amino)butoxy)ethyl)amino)benzo[c][2,6]naphthyridine-8-carboxamide (54l)

Reactants: amine **41** and 3-(oxazol-5-ylmethyl)-5-(trifluoromethoxy)benzaldehyde[36]

Product purified by reverse-phase HPLC (column: Phenomenex Gemini-NX C18 75 × 30 mm × 3 μm; eluents: A) 0.225% FA in H_2O (v/v), B) acetonitrile; gradient: 12–32% B, 7 min) to afford title compound **54l** (650 mg 38% yield) as a yellow oil.

LCMS (AM3): rt = 0.755 min, (609.2 $[M+H]^+$).

^{1}H NMR (400 MHz, MeOH-d_4) δ: 9.93 (s, 1H), 8.78 (d, *J* = 5.6 Hz, 1H), 8.62 (d, *J* = 8.4 Hz, 1H), 8.48 (s, 1H), 8.22 (d, *J* = 1.6 Hz, 1H), 8.13-8.10 (m, 2H), 7.82 (dd, *J* = 8.4 Hz, 2.0 Hz, 1H), 7.31-7.25 (m, 3H), 6.93 (s, 1H), 4.13 (s, 2H), 4.08 (s, 2H), 3.92 (t, *J* = 5.6 Hz, 2H), 3.81 (t, *J* = 5.6 Hz, 2H), 3.62 (t, *J* = 6.0 Hz, 2H), 3.02 (t, *J* = 7.2 Hz, 2H), 1.82-1.75 (m, 2H), 1.71-1.64 (m, 2H).

5-((2-(4-((3-((1H-Pyrazol-4-yl)methyl)-5-(trifluoromethoxy)benzyl)amino)butoxy)ethyl)amino)benzo[c][2,6]naphthyridine-8-carboxamide (54m)

Reactants: amine **41** and tert-butyl 4-(3-formyl-5-(trifluoromethoxy)benzyl)-1H-pyrazole-1-carboxylate[36]

Product purified purified by reverse-phase HPLC (column: Waters Xbridge 150 × 25 mm × 5 μm; eluents: A) 0.05% NH_4OH in H_2O, B) acetonitrile; gradient: 43–73% B, 10 min) to afford tert-butyl 4-(3-(((4-(2-((8-carbamoylbenzo[c][2,6]naphthyridin-5-

yl)amino)ethoxy)butyl)amino)methyl)-5-(trifluoromethoxy)benzyl)-1H-pyrazole-1-carboxylate (100 mg, 24% yield) as a white solid.

LCMS (AM3): rt = 0.809 min, (708.3 $[M+H]^+$).

tert-Butyl 4-(3-(((4-(2-((8-carbamoylbenzo[c][2,6]naphthyridin-5-yl)amino)ethoxy)butyl)amino)methyl)-5-(trifluoromethoxy)benzyl)-1H-pyrazole-1-carboxylate (100 mg, 129 mmol) in DCM (2 mL) was treated with TFA (1.54 g, 13.5 mmol) at room temperature and stirred for 0.5 h. The mixture was concentrated *in vacuo.*

Product purified by reverse-phase HPLC (column: Phenomenex Gemini-NX C18 75 × 30 mm × 3 μm; eluents: A) 0.225% FA in H_2O (v/v), B) acetonitrile; gradient: 8–38% B, 2 min) to afford title compound **54m** (74 mg, 80% yield, FA salt) as a yellow solid.

LCMS (AM3): rt = 0.748 min, (608.1 $[M+H]^+$).

^{1}H NMR (400 MHz, MeOH-d_4) δ: 9.92 (s, 1H), 8.77 (d, J = 6.0 Hz, 1H), 8.59 (d, J = 8.4 Hz, 1H), 8.35 (br s, 1H), 8.20 (d, J = 2.0 Hz, 1H), 8.12 (d, J = 5.6 Hz, 1H), 7.82 (dd, J = 8.4, 1.6 Hz, 1H), 7.44 (s, 2H), 7.27 (s, 1H), 7.20 (d, J = 2.4 Hz, 2H), 4.09 (s, 2H), 3.93-3.90 (m, 4H), 3.81 (t, J = 5.2 Hz, 2H), 3.61 (t, J = 6.0 Hz, 2H), 3.04 (t, J = 7.8 Hz, 2H), 1.85-1.76 (m, 2H), 1.73-1.64 (m, 2H).

Synthesis and analytical details for **55a-b**

5-((2-(4-((3-(Cyanomethyl)-5-(trifluoromethoxy)benzyl)amino)butoxy)ethyl)amino)benzo[c][2,6]naphthyridine-8-carboxylic acid (55a)

Reactants: amine **52** and 2-(3-formyl-5-(trifluoromethoxy)phenyl)acetonitrile[36]

Product purified by reverse-phase HPLC (column: Waters Xbridge 150 × 50 mm × 10 μm; eluents: A) 0.05% NH_4OH in H_2O (v/v), B) acetonitrile; gradient: 10–40% B, 11 min) to afford title compound **55a** (69.4 mg, 19% yield) as a yellow solid.

LCMS (AM7): rt = 0.734 min, (568.3 $[M+H]^+$).

^{1}H NMR (400 MHz, DMSO-d_6) δ: 10.04 (s, 1H), 8.85 (d, J = 5.2 Hz, 1H), 8.68 (d, J = 8.4 Hz, 1H), 8.26 (d, J = 5.6 Hz, 1H), 8.14 (s, 1H), 8.02–7.98 (m, 1H), 7.82 (d, J = 8.0 Hz, 1H), 7.34–7.28 (m, 2H), 7.20 (s, 1H), 4.09 (s, 2H), 3.80–3.77 (m, 2H), 3.72–3.67 (m, 6H), 2.50–2.49 (m, 2H), 1.58–1.42 (m, 4H).

5-((2-(4-((3-Chloro-4-(trifluoromethoxy)benzyl)amino)butoxy)ethyl)amino)benzo[c][2,6] naphthyridine-8-carboxylic acid (55b)

Reactants: amine **53** and 3-chloro-4-(trifluoromethoxy)benzaldehyde

Product purified by reverse-phase HPLC (column: Phenomenex Gemini-NX C18 75 × 30 mm × 3 μm; eluents: A) 0.1% TFA in H_2O (v/v), B) acetonitrile; gradient: 15–45% B, 7 min) to afford methyl 5-((2-(4-((3-chloro-4-(trifluoromethoxy)benzyl)amino)butoxy)ethyl)amino)benzo [c][2,6]naphthyridine-8-carboxylate (103 mg, = 37% yield) as a yellow oil.

LCMS (AM3): rt = 0.812 min, (577.1 $[M+H]^+$).

Methyl 5-((2-(4-((3-chloro-4-(trifluoromethoxy)benzyl)amino)butoxy)ethyl)amino)benzo [c][2,6]naphthyridine-8-carboxylate (103 mg, 178.51 mmol) in THF (3 mL), methanol (3 mL) and water (3 mL) was treated with LiOH monohydrate (18.2 mg, 433 mmol) at room temperature and stirred for 4 h. The mixture was acidified with aq. HCl (1 M) to pH 4 and concentrated *in vacuo.*

Product purified by reverse-phase HPLC (column: Phenomenex Gemini-NX C18 75 × 30 mm × 3 μm; eluents: A) 0.1% TFA in H_2O (v/v), B) acetonitrile; gradient: 12–42% B, 7 min) to afford title compound **55b** (26.8 mg, 46% yield, TFA salt) as a yellow solid.

LCMS (AM3): rt = 0.781 min, (563.1 $[M+H]^+$).

^{1}H NMR (400 MHz, MeOH-*d₄*) δ: 10.07 (s, 1H), 8.95 (d, *J* = 5.6 Hz, 1H), 8.77 (d, *J* = 8.5 Hz, 1H), 8.50 (d, *J* = 1.5 Hz, 1H), 8.33 (d, *J* = 5.8 Hz, 1H), 8.13 (dd, *J* = 1.6, 8.5 Hz, 1H), 7.71 (d, *J* = 1.8 Hz, 1H), 7.50-7.49 (m, 2H), 4.16 (s, 2H), 4.03 (t, *J* = 5.3 Hz, 2H), 3.88 (t, *J* = 5.3 Hz, 2H), 3.64-3.61 (t, 2H), 3.07-3.04 (m, 2H), 1.83-1.75 (m, 2H), 1.75-1.65 (m, 2H).

Synthesis and analytical details for **56a-h**

tert-Butyl N-[(1*S*)-2-[4-(benzyloxycarbonylamino)butoxy]-1-methyl-ethyl]carbamate

A mixture of tert-butyl N-[(1*S*)-2-hydroxy-1-methyl-ethyl]carbamate (2.0 g, 11 mmol), benzyl (4-bromobutyl)carbamate (6.6 g, 23 mmol), NaOH (4.57 g, 114 mmol) and TBAI (0.21 g, 0.57 mmol) in water (11 mL) was stirred at room temperature for 18 h. The mixture was diluted with water (80 mL) and extracted with ethyl acetate (20 mL × 3). The combined organic phases were washed with brine (40 mL), dried (Na_2SO_4), filtered and concentrated *in vacuo*. The residue was purified by reverse-phase HPLC (column: Phenomenex Synergi C18 150 × 25 mm

× 10 μm; eluents: A) 0.1% TFA in H_2O (v/v), B) acetonitrile; gradient: 10–90% B, 20 min) to afford the title compound (0.93 g, 16% yield) as a colorless oil.

LCMS (AM3): rt = 0.968 min, (403.2 $[M+Na]^+$).

(*S*)-Benzyl (4-(2-aminopropoxy)butyl)carbamate (56a)

A mixture of tert-butyl N-[(1S)-2-[4-(benzyloxycarbonylamino)butoxy]-1-methyl-ethyl]carbamate (820 mg, 2.16 mmol) in a solution of HCl in 1,4-dioxane (20 mL, 4 M) was stirred at room temperature for 1 h. The mixture was concentrated *in vacuo* and the residue was purified by reverse-phase HPLC (column: Phenomenex Luna C18 150 × 40 mm × 15 μm; eluents: A) 0.05% HCl in H_2O (v/v), B) acetonitrile; gradient: 10–40% B, 10 min) to afford title compound **56a** (460 mg, 76% yield, HCl salt) as a colorless oil.

LCMS (AM3): rt = 0.658 min, (281.1 $[M+H]^+$).

(*S*)-Methyl 5-((1-(4-(((benzyloxy)carbonyl)amino)butoxy)propan-2-yl)amino)benzo[c][2,6]naphthyridine-8-carboxylate

A mixture of amine **56a** (440 mg, 1.57 mmol, HCl salt), methyl 5-chlorobenzo [*c*] 2,6-naphthyridine-8 carboxylate[14] (480 mg, 1.73 mmol) and DIPEA (609 mg, 4.71 mmol) in DMSO (10 mL) was stirred at 80 °C for 12 h. The mixture was filtered, and the filtrate was concentrated *in vacuo*. The residue was purified by reverse-phase HPLC (column: Phenomenex Synergi C18 150 × 25 mm × 10 μm; eluents: A) 0.1% HCl in H_2O (v/v), B) acetonitrile; gradient: 10–90% B, 20 min) to afford the title compound (400 mg, 46% yield) as a yellow gum.

LCMS (AM3): rt = 0.849 min, (517.4 $[M+H]^+$).

(R)-Benzyl 3-(3-((tert-butoxycarbonyl)amino)propoxy)pyrrolidine-1-carboxylate

To a solution of (*R*)-benzyl 3-hydroxypyrrolidine-1-carboxylate (1.0 g, 4.5 mmol) in DMF (15 mL) was added NaH (199 mg, 4.97 mmol, 60% dispersion in oil) slowly at 0 °C. After stirring for 0.5 h, tert-butyl (3-bromopropyl)carbamate (1.18 g, 4.97 mmol) was added, and the mixture was stirred at 0 °C for 3 h. The mixture was diluted with iced-water (50 mL) and extracted with ethyl acetate (80 mL × 2). The combined organic phases were washed with brine (100 mL), dried (Na_2SO_4), filtered and reduced *in vacuo*. The residue was purified by column chromatography on silica gel eluting with petroleum ether/ethyl acetate (1:1) to afford the title compound (820 mg, 48% yield) as a colourless oil.

^{1}H NMR (400 MHz, $CHCl_3$-*d*) δ: 7.40-7.28 (m, 5H), 5.20-5.09 (m, 2H), 4.77 (br s, 1H), 4.05-3.98 (m, 1H), 3.54-3.42 (m, 6H), 3.24-3.12 (m, 2H), 2.05-1.86 (m, 2H), 1.77-1.70 (quin, 2H), 1.43 (s, 9H).

(R)-tert-Butyl (3-(pyrrolidin-3-yloxy)propyl)carbamate (56e)

To a solution of (R)-benzyl 3-(3-((tert-butoxycarbonyl)amino)propoxy)pyrrolidine-1-carboxylate (700 mg, 1.85 mmol) in methanol (8 mL) was added 10% Pd/C (100 mg) at room temperature under hydrogen and the mixture stirred under hydrogen (15 psi) for 12 h. The catalyst was removed by filtration and the filtrate was reduced *in vacuo* to afford title compound **56e** (460 mg) as a colourless oil, which was used in the next step without further purification.

(R)-methyl 5-(3-(3-((tert-butoxycarbonyl)amino)propoxy)pyrrolidin-1-yl)benzo[c][2,6]naphthyridine-8-carboxylate

A mixture of amine **56e** (460 mg, 1.88 mmol), methyl 5-chlorobenzo [*c*] 2,6-naphthyridine-8 carboxylate[14] (513 mg, 1.88 mmol) and DIPEA (487 mg, 3.77 mmol) in DMSO (10 mL) was stirred at 80 °C for 12 h. The mixture was diluted with water (50 mL) and extracted with ethyl acetate (50 mL × 2). The combined organic layers were washed with brine (80 mL), dried (Na_2SO_4), filtered and reduced *in vacuo*. The residue was purified by reverse-phase HPLC (column: Phenomenex Synergi C18 150 × 25 mm × 10 μm; eluents: A) 0.1% HCl in H_2O (v/v), B) acetonitrile; gradient: 10–90% B, 20 min) to afford the title compound (600 mg, 66% yield) as a dark brown oil.

LCMS (AM3): rt = 0.844 min, (481.1 $[M+H]^+$).

tert-Butyl (4-((2S)-2-((tetrahydro-2H-pyran-2-yl)oxy)propoxy)butyl)carbamate

To a solution of NaOH (13.23 g, 330.81 mmol) in water (33.08 mL) was added (2S)-2-((tetrahydro-2H-pyran-2-yl)oxy)propan-1-ol (5.3 g, 33 mmol), tert-butyl (4-bromobutyl)carbamate (20 g, 79 mmol) and TBAI (611 mg, 1.65 mmol) at room temperature and the mixture was stirred for 12 h. The mixture was diluted with water (100 mL) and extracted with MTBE (100 mL × 2). The combined organic layers were washed with brine (150 mL), dried (Na_2SO_4), filtered and concentrated *in vacuo*. The residue was purified by column chromatography on silica gel eluting with petroleum ether/ethyl acetate (10:1) to afford the title compound (3 g, 27% yield) as a colourless oil, which was used in the next step without further purification

(S)-Tert-butyl (4-(2-hydroxypropoxy)butyl)carbamate (56h)

To a solution of tert-butyl (4-((2S)-2-((tetrahydro-2H-pyran-2-yl)oxy)propoxy)butyl)carbamate (3.0 g, 9.0 mmol) in methanol (25 mL) was added TsOH monohydrate (200 mg, 1.16 mmol) at room temperature and the mixture was stirred for 1 h. The mixture was concentrated *in vacuo* and the residue was purified by column chromatography on silica gel eluting with petroleum ether/ethyl acetate (3:1) to afford title compound **56h** (940 mg, 42% yield) as a colourless oil.

^{1}H NMR (400 MHz, $CHCl_3$-*d*) δ: 4.71 (br s, 1H), 4.00-3.92 (m, 1H), 3.54-3.44 (m, 2H), 3.42-3.39 (m, 1H), 3.24-3.19 (t, 1H), 3.15-3.05 (m, 2H), 1.96 (br s, 1H), 1.65-1.51 (m, 4H), 1.44 (s, 9H), 1.14 (d, J = 6.4 Hz, 3H).

Synthesis and analytical details for **57a-i**

(S)-Methyl 5-((1-(4-((tert-butoxycarbonyl)amino)butoxy)propan-2-yl)oxy)benzo[c][2,6]naphthyridine-8-carboxylate

To a solution of alcohol **56h** (820 mg, 3.32 mmol) in THF (20 mL) was added NaH (199 mg, 4.97 mmol, 60% dispersion in oil) at room temperature. After stirring for 0.5 h, methyl 5-chlorobenzo [*c*] 2,6-naphthyridine-8 carboxylate[14] (904 mg, 3.32 mmol) was added and the mixture was stirred for 2 h. The mixture was diluted with water (80 mL) and extracted with ethyl acetate (60 mL × 2). The combine organic layers were washed with brine (80 mL), dried (Na_2SO_4), filtered and concentrated *in vacuo*. The residue was purified by reverse-phase HPLC (column: Phenomenex Synergi C18 150 × 25 mm × 10 µm; eluents: A) 0.1% TFA in H_2O (v/v), B) acetonitrile; gradient: 10–90% B, 20 min) to afford the title compound (460 mg, 29% yield) as a brown solid.

LCMS (AM3): rt = 1.029 min, (484.2 $[M+H]^+$).

(*S*)-Methyl 5-((1-(4-aminobutoxy)propan-2-yl)amino)benzo[c][2,6]naphthyridine-8-carboxylate (57a)

To a mixture of (*S*)-methyl 5-((1-(4-(((benzyloxy)carbonyl)amino)butoxy)propan-2-yl)amino)benzo[c] [2,6]naphthyridine-8-carboxylate (400 mg, 723 mmol, HCl salt) and aq. ammonium hydroxide solution (1.00 mL, 25%) in methanol (10 mL) was added 10% palladium on charcoal catalyst (0.2 g) under a nitrogen atmosphere. The resulting suspension was hydrogenated under hydrogen (15 psi) at room temperature for 1 h. The catalyst was removed

by filtration and the filtrate was concentrated *in vacuo* to afford title compound **57a** (170 mg, 62% yield) as a yellow solid, which was used directly without further purification.

LCMS (AM3): rt = 0.599 min, (383.3 $[M+H]^+$).

(*R*)-Methyl 5-((1-(4-aminobutoxy)propan-2-yl)amino)benzo[c][2,6]naphthyridine-8-carboxylate (57b)

Synthesized according to the 4-step procedure for (*S*)-methyl 5-((1-(4-aminobutoxy)propan-2-yl)amino)benzo[c][2,6]naphthyridine-8-carboxylate (**57a**), starting with tert-butyl N-[(1*R*)-2-hydroxy-1-methyl-ethyl]carbamate (2.0 g, 11 mmol), to afford title compound **57b** (0.34 g, 98% yield) as a yellow solid.

LCMS (AM3): rt = 0.703 min, (383.3 $[M+H]^+$).

Methyl 5-((1-(4-aminobutoxy)-2-methylpropan-2-yl)amino)benzo[c][2,6]naphthyridine-8-carboxylate (57c)

Synthesized according to the 4-step procedure for (*S*)-methyl 5-((1-(4-aminobutoxy)propan-2-yl)amino)benzo[c][2,6]naphthyridine-8-carboxylate (**57a**), starting with tert-butyl (1-hydroxy-2-methylpropan-2-yl)carbamate (4.0 g, 21 mmol), to afford title compound **57c** (90 mg, 34% yield, TFA salt) as a yellow oil.

LCMS (AM3): rt = 0.726 min, (397.0 $[M+H]^+$).

Methyl 5-(3-(4-aminobutoxy)azetidin-1-yl)benzo[c][2,6]naphthyridine-8-carboxylate (57d)

Synthesized according to the 4-step procedure for (*S*)-methyl 5-((1-(4-aminobutoxy)propan-2-yl)amino)benzo[c][2,6]naphthyridine-8-carboxylate (**57a**), starting with tert-butyl 3-hydroxyazetidine-1-carboxylate (1.0 g, 5.8 mmol), to afford title compound **57d** (0.29 g, 98% yield) as a yellow solid.

LCMS (AM3): rt = 0.690 min, (381.2 [M+H]+), 94.2% purity.

(R)-Methyl 5-(3-(3-aminopropoxy)pyrrolidin-1-yl)benzo[c][2,6]naphthyridine-8-carboxylate (57e)

To a solution of (R)-methyl 5-(3-(3-((tert-butoxycarbonyl)amino)propoxy)pyrrolidin-1-yl)benzo[c][2,6]naphthyridine-8-carboxylate (600 mg, 1.25 mmol) in methanol (5 mL) was added a solution of HCl in methanol (10 mL, 4 M) at room temperature and the mixture was stirred for 1 h. The mixture was reduced *in vacuo* to afford title compound **57e** (600 mg, HCl salt) as a brown solid, which was used directly without purification.

LCMS (AM5): rt = 0.806 min, (381.3 $[M+H]^+$),.

(S)-Methyl 5-(3-(3-aminopropoxy)pyrrolidin-1-yl)benzo[c][2,6]naphthyridine-8-carboxylate (57f)

Synthesized according to the 4-step procedure for (R)-methyl 5-(3-(3-aminopropoxy)pyrrolidin-1-yl)benzo[c][2,6]naphthyridine-8-carboxylate (**57e**), starting with (*S*)-benzyl 3-hydroxypyrrolidine-1-carboxylate (1.0 g, 4.5 mmol), to afford title compound **57f** (800 mg) as a brown solid.

LCMS (AM5): rt = 0.800 min, (381.3 $[M+H]^+$).

Methyl 5-(4-(3-aminopropoxy)piperidin-1-yl)benzo[c][2,6]naphthyridine-8-carboxylate (57g)

Synthesized according to the 4-step procedure for (R)-methyl 5-(3-(3-aminopropoxy)pyrrolidin-1-yl)benzo[c][2,6]naphthyridine-8-carboxylate (**57e**), starting with benzyl 4-hydroxypiperidine-1-carboxylate (5.0 g, 21 mmol), to afford title compound **57g** (450 mg, HCl salt) as a yellow solid.

LCMS (AM3): rt = 0.778 min, (395.3 $[M+H]^+$).

(S)-Methyl 5-((1-(4-aminobutoxy)propan-2-yl)oxy)benzo[c][2,6]naphthyridine-8-carboxylate (57h)

To a solution of (S)-methyl 5-((1-(4-((tert-butoxycarbonyl)amino)butoxy)propan-2-yl)oxy)benzo[c][2,6]naphthyridine-8-carboxylate (220 mg, 455 mmol) in 1,4-dioxane (1 mL) was added a solution of HCl in 1,4-dioxane (4 M, 11 mL) and the mixture was stirred at room temperature for 0.5 h. The mixture was concentrated *in vacuo* to give a residue that was purified by reverse-phase HPLC (column: Phenomenex Synergi C18 150 × 25 mm × 10 µm; eluents: A) 0.1% FA in H_2O (v/v), B) acetonitrile; gradient: 10–90% B, 20 min) to afford title compound **57h** (240 mg, TFA salt) as a yellow solid.

LCMS (AM5): rt = 0.987 min, (384.2 $[M+H]^+$).

(R)-Methyl 5-((1-(4-aminobutoxy)propan-2-yl)oxy)benzo[c][2,6]naphthyridine-8-carboxylate (57i)

Synthesized according to the 4-step procedure for (S)-methyl 5-((1-(4-aminobutoxy)propan-2-yl)oxy)benzo[c][2,6]naphthyridine-8-carboxylate (**57h**), starting with (2R)-2-((tetrahydro-2H-

pyran-2-yl)oxy)propan-1-ol (5.0 g, 31 mmol), to afford title compound **57i** (230 mg, TFA salt) as a yellow solid.

LCMS (AM5): rt = 0.986 min, (384.2 $[M+H]^+$).

General Procedure for the Synthesis of **58a-i.**

Step 1: To a solution of the respective amine **57a-i** (1 eq.) and 3-chloro-4-(trifluoromethoxy)benzaldehyde (1 eq.) in methanol (1−15 mL) was added either DIPEA (1.5−3 eq.) or sodium acetate (2−3 eq.) at room temperature and the mixture stirred for 1−16 h. $NaBH(OAc)_3$ (3−4 eq.) or $NaBH_3CN$ (1−6 eq.) was added and the mixture was stirred at room temperature for 0.5−3.5 h. The reaction mixture was concentrated *in vacuo* and the residue was purified by the method indicated to afford the title compound**.**

Step 2: A solution of the product from Step 1 (1 eq.) in THF (1−10 mL), methanol (0.5−3 mL) and water (1−5 mL) was treated with LiOH monohydrate (2−10 eq.) at room temperature and stirred for 1−20 h. The mixture was acidified with aq. HCl (1 M) to pH 4, concentrated *in vacuo* and purified by the method indicated to afford the title compound.

Synthesis and analytical details for **58a-i**

(*S*)-5-((1-(4-((3-Chloro-4-(trifluoromethoxy)benzyl)amino)butoxy)propan-2-yl)amino)benzo[c][2,6]naphthyridine-8-carboxylic acid (58a)

Step 1: Reactant: amine **57a**. Product purified by reverse-phase HPLC (column: Phenomenex Luna C18 150 × 25 mm × 10 μm; eluents: A) 0.1% TFA in H_2O (v/v), B) acetonitrile; gradient: 23–53% B, 11 min) to afford (S)-methyl 5-((1-(4-((3-chloro-4-(trifluoromethoxy)benzyl)amino)butoxy)propan-2-yl)amino)benzo[c][2,6]naphthyridine-8-carboxylate (50 mg, 29% yield, TFA salt) as a yellow solid.

LCMS (AM3): rt = 0.852 min, (591.2 $[M+H]^+$).

Step 2: Product purified by reverse-phase HPLC (column: Shim-pack C18 150 × 25 mm × 10 μm; eluents: A) 0.225% FA in H_2O (v/v), B) acetonitrile; gradient: 17–47% B, 10 min) to afford title compound **58a** (16.6 mg, 42% yield, FA salt) as a yellow solid.

LCMS (AM3): rt = 0.688 min, (577.1 $[M+H]^+$).

^1H NMR (400 MHz, MeOH-d_4) δ : 9.93 (s, 1H), 8.77 (d, *J* = 5.6 Hz, 1H), 8.55 (d, *J* = 8.4 Hz, 1H), 8.48 (br s, 1H), 8.28 (d, *J* =1.2 Hz, 1H), 8.21 (d, *J* = 5.6 Hz, 1H), 7.95 (dd, *J* = 8.4, 2.0 Hz, 1H), 7.67 (s, 1H), 7.48 (s, 2H), 4.91-4.89 (m, 1H), 4.07 (s, 2H), 3.82-3.76 (m, 2H), 3.64-

3.59 (m, 1H), 3.55-3.51 (m, 1H), 3.02 (t, J =7.2 Hz, 2H), 1.86-1.78 (m, 2H), 1.71-1.63 (m, 2H), 1.40 (d, J = 6.8 Hz, 3H).

(R)-5-((1-(4-((3-Chloro-4-(trifluoromethoxy)benzyl)amino)butoxy)propan-2-yl)amino)benzo[c][2,6]naphthyridine-8-carboxylic acid (58b)

Step 1: Reactant: amine **57b**. Product purified by reverse-phase HPLC (column: Phenomenex Synergi C18 150 × 25 mm × 10 μm; eluents: A) 0.1% TFA in H_2O (v/v), B) acetonitrile; gradient: 25–55% B, 10 min) to afford (R)-methyl 5-((1-(4-((3-chloro-4-(trifluoromethoxy)benzyl)amino)butoxy)propan-2-yl)amino)benzo[c][2,6]naphthyridine-8-carboxylate (0.15 g, 18% yield, TFA salt) as a yellow solid.

LCMS (AM3): rt = 0.838 min, (591.2 $[M+H]^+$).

Step 2: Product purified by reverse-phase HPLC (column: Shim-pack C18 150 × 25 mm × 10 μm; eluents: A) 0.225% FA in H_2O (v/v), B) acetonitrile; gradient: 17–47% B, 10 min) to afford title compound **58b** (35.7 mg, 39% yield) as a yellow solid.

LCMS (AM3): rt = 0.800 min, (577.2 $[M+H]^+$).

^{1}H NMR (400 MHz, MeOH-d_4) δ : 9.84 (s, 1H), 8.69 (d, J = 5.6 Hz, 1H), 8.44 (d, J = 8.4 Hz, 1H), 8.22 (d, J = 1.6 Hz, 1H), 8.12 (d, J = 6.0 Hz, 1 H), 7.89 (dd, J = 8.4 Hz, 1.6 Hz, 1H), 7.63 (d, J = 2.0 Hz, 1H), 7.46-7.38 (m, 2H), 4.83-4.76 (m, 1H), 3.99 (s, 2H), 3.74-3.66 (m, 2H), 3.58-3.53 (m, 1H), 3.50-3.46 (m, 1H), 2.96-2.89 (m, 2H), 1.81-1.74 (m, 2H), 1.67-1.59 (m, 2H), 1.35 (d, J = 6.8 Hz, 3H).

5-((1-(4-((3-Chloro-4-(trifluoromethoxy)benzyl)amino)butoxy)-2-methylpropan-2-yl)amino)benzo[c][2,6]naphthyridine-8-carboxylic acid (58c)

Step 1: Reactant: amine **57c**. Product purified by reverse-phase HPLC (column: Unisil 3-100 C18 Ultra 150 × 50 mm × 3 μm; eluents: A) 0.225% FA in H_2O (v/v), B) acetonitrile; gradient: 25–45% B, 10 min) to afford methyl 5-((1-(4-((3-chloro-4-(trifluoromethoxy)benzyl)amino)butoxy)-2-methylpropan-2-yl)amino)benzo[c][2,6]naphthyridine-8-carboxylate (25 mg, 22% yield, TFA salt) as a white solid.

LCMS (AM3): rt = 0.871 min, (605.4 [M+H]+).

Step 2: Product purified by reverse-phase HPLC (column: Phenomenex Luna C18 150 × 25 mm × 10 μm; eluents: A) 0.225% FA in H_2O (v/v), B) acetonitrile; gradient: 11–41% B, 10 min) to afford title compound **58c** (14.7 mg, 65% yield) as a white solid.

LCMS (AM3): rt = 0.824 min, (591.2 [M+H]+).

1H NMR (400 MHz, MeOH-d4) δ: 9.86 (s, 1H), 8.72 (d, J = 6.0 Hz, 1H), 8.47 (d, J = 8.4 Hz, 1H), 8.26 (s, 1H), 8.08 (d, J = 5.6 Hz, 1H), 7.89 (d, J = 8.4 Hz, 1H), 7.64 (s, 1H), 7.48-7.46 (m, 1H), 7.44-7.41 (m, 1H), 4.01 (s, 2H), 3.97 (s, 2H), 3.54 (t, J = 5.6 Hz, 2H), 2.89 (t, J = 6.8 Hz, 2H), 1.72-1.60 (m, 4H), 1.60 (s, 6H).

5-(3-(4-((3-Chloro-4-(trifluoromethoxy)benzyl)amino)butoxy)azetidin-1-yl)benzo[c][2,6]naphthyridine-8-carboxylic acid (58d)

Step 1: Reactant: amine **57d**. Product purified by reverse-phase HPLC (column: Phenomenex Luna C18 150 × 40 mm × 15 μm; eluents: A) 0.225% FA in H_2O (v/v), B) acetonitrile; gradient: 14–44% B, 11 min) to afford methyl 5-(3-(4-((3-chloro-4-(trifluoromethoxy)benzyl)amino)butoxy)azetidin-1-yl)benzo[c][2,6]naphthyridine-8-carboxylate (0.17 g, 38% yield) as a yellow solid.

LCMS (AM3): rt = 0.815 min, (589.2 $[M+H]^+$).

Step 2: Product purified by reverse-phase HPLC (column: Phenomenex Luna C18 150 × 25 mm × 10 μm; eluents: A) 0.225% FA in H_2O (v/v), B) acetonitrile; gradient: 10–40% B, 10 min) to afford title compound **58d** (124.4 mg, 75% yield) as a yellow solid.

LCMS (AM3): rt = 0.788 min, (575.2 $[M+H]^+$).

^{1}H NMR (400 MHz, DMSO-d_6) δ: 10.12 (s, 1H), 8.83 (d, *J* = 5.6 Hz, 1H), 8.77 (d, *J* = 8.8 Hz, 1H), 8.23–8.21 (m, 1H), 7.96 (d, *J* = 5.2 Hz, 1H), 7.91–7.89 (d, 1H), 7.67 (s, 1H), 7.52–7.42 (q, 2H), 4.73–4.69 (t, *2*H), 4.49–4.43 (m, 1H), 4.33–4.29 (m, 2H), 3.78 (s, 2H), 3.45 (t, *J* = 6.4 Hz, 2H), 2.58–2.55 (m, 2H), 1.62–1.50 (m, 4H).

(R)-5-(3-(3-((3-Chloro-4-(trifluoromethoxy)benzyl)amino)propoxy)pyrrolidin-1-yl)benzo[c][2,6]naphthyridine-8-carboxylic acid (58e)

Step 1: Reactant: amine **57e**. Product purified by reverse-phase HPLC (column: Phenomenex Synergi Max-RP 250 × 50 mm × 10 μm; eluents: A) 0.05% HCl in H_2O, B) acetonitrile; gradient: 15–45% B, 30 min) to afford (R)-methyl 5-(3-(3-((3-chloro-4-

(trifluoromethoxy)benzyl)amino)propoxy)pyrrolidin-1-yl)benzo[c][2,6]naphthyridine-8-carboxylate (200 mg, 26% yield, HCl salt) as a yellow oil.

LCMS (AM3): rt = 0.822 min, (589.3 $[M+H]^+$).

Step 2: Product purified by reverse-phase HPLC (column: Waters Xbridge 150 × 25 mm × 5 µm; eluents: A) 0.05% NH_4OH in H_2O, B) acetonitrile; gradient: 15–45% B, 10 min) to afford title compound **58e** (133 mg, 68% yield) as a yellow solid.

LCMS (AM3): rt = 0.781 min, (575.2 $[M+H]^+$).

^{1}H NMR (400 MHz, DMSO-d_6) δ: 10.08 (s, 1H), 8.81 (d, *J* = 6.0 Hz, 1H), 8.73 (d, *J* = 8.4 Hz, 1H), 8.21-8.18 (m, 2H), 7.85 (dd, *J* = 8.4, 1.6 Hz, 1H), 7.58 (d, *J* = 2.0 Hz, 1H), 7.46-7.34 (m, 2H), 4.20-4.16 (m, 1H), 4.14-4.09 (m, 1H), 4.08-4.00 (m, 1H), 3.87-3.81 (m, 1H), 3.79-3.76 (m, 1H), 3.64 (s, 2H), 3.55-3.45 (s, 2H), 2.53-2.51 (m, 2H), 2.10-2.03 (m, 2H), 1.68-1.60 (quin, 2H).

(S)-5-(3-(3-((3-Chloro-4-(trifluoromethoxy)benzyl)amino)propoxy)pyrrolidin-1-yl)benzo[c][2,6]naphthyridine-8-carboxylic acid (58f)

Step 1: Reactant: amine **57f**. Product purified by reverse-phase HPLC (column: Phenomenex Synergi Max-RP 250 × 50 mm × 10 µm; eluents: A) 0.05% HCl in H_2O, B) acetonitrile; gradient: 17–47% B, 30 min) to afford (S)-methyl 5-(3-(3-((3-chloro-4-(trifluoromethoxy)benzyl)amino)propoxy)pyrrolidin-1-yl)benzo[c][2,6]naphthyridine-8-carboxylate (260 mg, 26% yield, HCl salt) as a yellow oil.

LCMS (AM3): rt = 0.825 min, (589.3 $[M+H]^+$).

Step 2: Product purified by reverse-phase HPLC (column: Waters Xbridge 150 × 25 mm × 5 µm; eluents: A) 0.05% NH_4OH in H_2O, B) acetonitrile; gradient: 28–58% B, 10 min) to afford title compound **58f** (74 mg, 29% yield) as a yellow solid.

LCMS (AM3): rt = 0.781 min, (575.1 $[M+H]^+$).

^{1}H NMR (400 MHz, DMSO-d_6) δ: 10.07 (s, 1H), 8.79 (d, *J* = 5.6 Hz, 1H), 8.71 (d, *J* = 8.4 Hz, 1H), 8.19-8.17 (m, 2H), 7.85 (dd, *J* = 8.4, 1.6 Hz, 1H), 7.59 (d, *J* = 2.0 Hz, 1H), 7.45-7.36 (m, 2H), 4.20-4.16 (m, 1H), 4.1 (dd, *J* =12.0, 4.8 Hz, 1H), 4.06-4.00 (m, 1H), 3.86-3.80 (m, 1H), 3.78-3.75 (m, 1H), 3.67 (s, 2H), 3.57-3.47 (m, 2H), 2.55-2.53 (m, 2H), 2.09-2.02 (m, 2H), 1.70-1.62 (quin, 2H).

5-(4-(3-((3-Chloro-4-(trifluoromethoxy)benzyl)amino)propoxy)piperidin-1-yl)benzo[c][2,6]naphthyridine-8-carboxylic acid (58g)

Step 1: Reactant: amine **57g**. Product purified by reverse-phase HPLC (column: Phenomenex Luna C18 250 × 50 mm × 10 µm; eluents: A) 0.225% FA in H_2O (v/v), B) acetonitrile; gradient: 23–53% B, 10 min) to afford methyl 5-(4-(3-((3-chloro-4-(trifluoromethoxy)benzyl)amino)propoxy)piperidin-1-yl)benzo[c][2,6]naphthyridine-8-carboxylate (100 mg, 36% yield) as a yellow oil.

LCMS (AM3): rt = 0.903 min, (603.2 $[M+H]^+$).

Step 2: Product purified by reverse-phase HPLC (column: Unisil 3-100 C18 Ultra 150 × 50 mm × 3 µm; eluents: A) 0.225% FA in H_2O (v/v), B) acetonitrile; gradient: 20–50% B, 10 min) to afford title compound **58g** (63.4 mg, 64% yield) as an off-white solid.

LCMS (AM3): rt = 0.850 min, (589.2 $[M+H]^+$).

^{1}H NMR (400 MHz, DMSO-*d*$_6$) δ: 10.18 (s, 1H), 8.90 (d, *J* = 5.6 Hz, 2H), 8.33 (d, *J* = 1.6 Hz, 1H), 8.16 (s, 1H), 8.03 (dd, *J* = 8.4, 1.6 Hz, 1H), 7.97 (d, *J* = 5.6 Hz, 1H), 7.67 (d, *J* = 2.0 Hz, 1H), 7.52-7.43 (m, 2H), 3.79 (s, 2H), 3.77-3.69 (m, 2H), 3.63-3.53 (m, 3H), 3.24-3.20 (m, 2H), 2.63 (t, *J* = 8.2 Hz, 2H), 2.08-2.02 (m, 2H), 1.79-1.69 (m, 4H).

(S)-5-((1-(4-((3-Chloro-4-(trifluoromethoxy)benzyl)amino)butoxy)propan-2-yl)oxy)benzo[c][2,6]naphthyridine-8-carboxylic acid (58h)

Step 1: Reactant: amine **57h**. Product purified by reverse-phase HPLC (column: Phenomenex Luna C18 150 × 25 mm × 10 µm; eluents: A) 0.225% FA in H_2O (v/v), B) acetonitrile; gradient: 28–58% B, 10 min) to afford (S)-methyl 5-((1-(4-((3-chloro-4-(trifluoromethoxy)benzyl)amino)butoxy)propan-2-yl)oxy)benzo[c][2,6]naphthyridine-8-carboxylate (200 mg, 70% yield) as a white solid.

LCMS (AM3): rt = 0.907 min, (592.2 $[M+H]^+$).

Step 2: Product purified by reverse-phase HPLC (column: Phenomenex Gemini-NX C18 75 × 30 mm × 3 µm; eluents: A) 0.05% NH_4OH in H_2O (v/v), B) acetonitrile; gradient: 9–39% B, 10 min) to afford title compound **58h** (19.8 mg, 41% yield) as a yellow gum.

LCMS (AM3): rt = 0.851 min, (578.0 $[M+H]^+$).

^{1}H NMR (400 MHz, MeOH-*d*$_4$) δ: 9.94 (s, 1H), 8.78 (d, *J* = 5.6 Hz, 1H), 8.61 (d, *J* = 8.4 Hz, 1H), 8.39 (d, *J* = 1.2 Hz, 1H), 8.14 (d, *J* = 5.6 Hz, 1H), 8.10 (dd, *J* = 8.4, 1.6 Hz, 1H), 7.63 (s,

1H), 7.43 (s, 2H), 5.93-5.85 (m, 1H), 3.99 (s, 2H), 3.86-3.80 (m, 1H), 3.75-3.67 (m, 2H), 3.60-3.55 (m, 1H), 2.90 (t, *J* = 7.6 Hz, 2H), 1.78-1.70 (quin, 2H), 1.68-1.52 (m, 2H), 1.47 (d, *J* = 6.4 Hz, 3H).

(R)-5-((1-(4-((3-Chloro-4-(trifluoromethoxy)benzyl)amino)butoxy)propan-2-yl)oxy)benzo[c][2,6]naphthyridine-8-carboxylic acid (58i)

Step 1: Reactant: amine **57i**. Product purified by reverse-phase HPLC (column: Phenomenex Luna C18 150 × 25 mm × 10 μm; eluents: A) 0.225% FA in H_2O (v/v), B) acetonitrile; gradient: 28−58% B, 10 min) to afford (R)-methyl 5-((1-(4-((3-chloro-4-(trifluoromethoxy)benzyl)amino)butoxy)propan-2-yl)oxy)benzo[c][2,6]naphthyridine-8-carboxylate (210 mg, 74% yield) as a white solid.

LCMS (AM3): rt = 0.906 min, (592.2 $[M+H]^+$).

Step 2: Product purified by reverse-phase HPLC (column: Phenomenex Gemini-NX C18 75 × 30 mm × 3 μm; eluents: A) 0.05% NH_4OH in H_2O (v/v), B) acetonitrile; gradient: 9−39% B, 10 min) to afford title compound **58i** (21.5 mg, 44% yield) as a white solid.

LCMS (AM3): rt = 0.853 min, (578.2 $[M+H]^+$).

^{1}H NMR (400 MHz, CD_3OD) δ: 9.96 (s, 1H), 8.79 (d, *J* = 5.6 Hz, 1H), 8.63 (d, *J* = 8.4 Hz, 1H), 8.39 (d, *J* = 1.6 Hz, 1 H), 8.16 (d, *J* = 5.6 Hz, 1 H), 8.11 (dd, *J* = 8.4, 1.6 Hz, 1 H), 7.65 (s, 1H), 7.47-7.42 (m, 2H), 5.96-5.88 (m, 1H), 4.02 (s, 2H), 3.87-3.83 (m, 1H), 3.76-3.67 (m, 2H), 3.61-3.55 (m, 1H), 2.94 (t, *J* = 7.6 Hz, 2H), 1.79-1.72 (m, 2H), 1.69-1.53 (m, 2H), 1.48 (d, *J* = 6.4 Hz, 3H).

HPLC analysis of **61f**

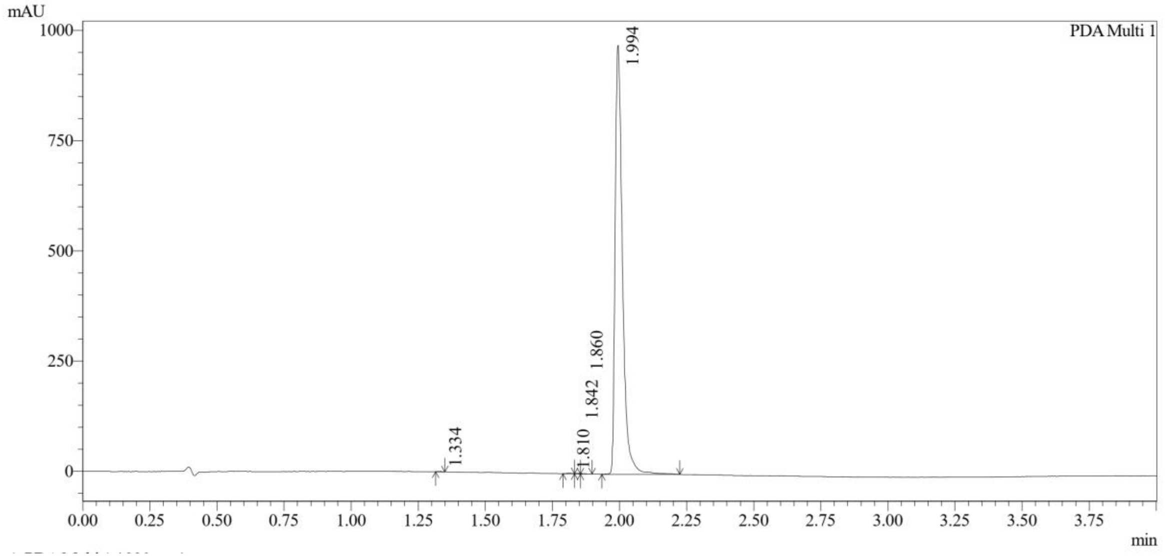

Figure S1. HPLC chromatogram of **61f** (APL5125). Main peak with **61f** is 99.7% of the total peak area.

Initial Screening

Table S1. Structures and activity data for 28 compounds screened initially.

Compound	Structure	CK2α IC_{50} [nM]	CLK2 IC_{50} [nM]	DAPK3 IC_{50} [nM]	NanoBRET™Cellular Assay IC_{50} [nM]
**1** (CX-4945, Silmitasertib)	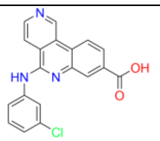	0.678 ± 0.196[b]	4.92 ± 1.44[b]	5.11 ± 1.27[b]	107 ± 45[b]
**4** (AZ 7h)	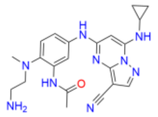	0.348 ± 0.096[b]	2,678 ± 1139[b]	5.23 ± 0.81[b]	5.37 ± 1.09[b]
**6** (CAM4066)	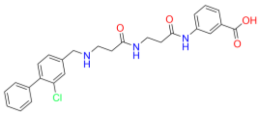	300 ± 80	>50,000[a]	43,550[a]	>50,000[a]
**12**	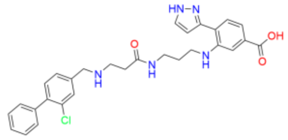	28.8± 5.11[a]	>50,000	32,950 ± 2205	ND
**14**	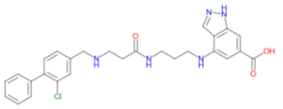	132	21,840 ± 9527	1,383 ± 574	>50,000
**18**	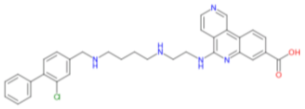	0.386 ± 0.730[a]	216 ± 33[a]	22.6 ± 4.0	1,156 ± 681
**21**	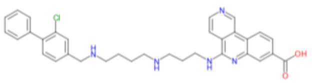	0.184 ± 0.014	164	22.4	ND
	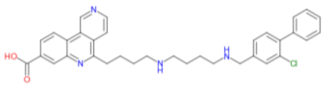	0.214 ± 0.003	23.7	8.38	ND
	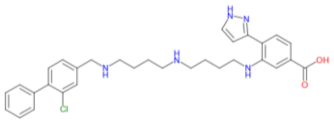	15.9 ± 6.74	25,270	23,430	ND
	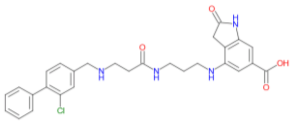	26.2	>50,000	14,780 ± 48	ND

	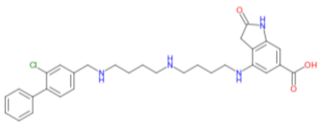	26.9	>50000	2,147	ND
	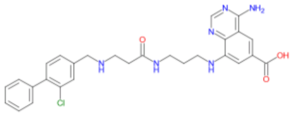	80.4	3,387	2,511	ND
	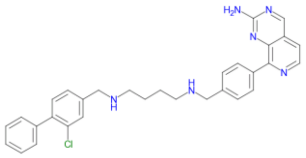	94.1± 12.6	1,407	4,423	2211 ± 768
	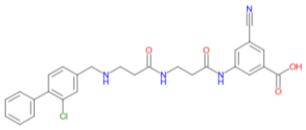	136	>50,000	>50,000	ND
	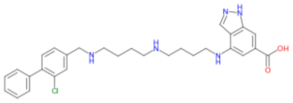	215	30,170	314	ND
	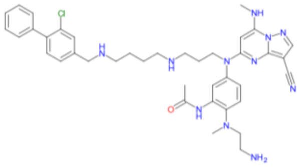	256 ± 83.3[a]	24,250 ± 3173[a]	7,544 ± 4,812	ND
	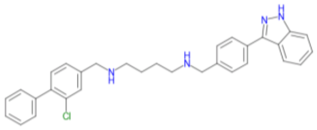	780 ± 316[a]	6,352	5,351	ND
	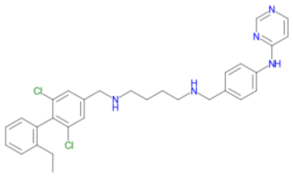	1,819 ± 558[a]	35,540	6,106	ND
	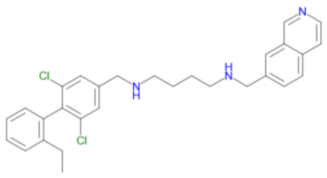	2,936	>50,000	12,390	ND
	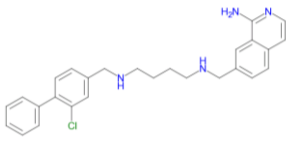	6,158	31,570	20,010	43250

	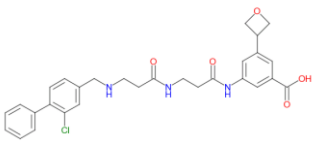	6475	>50,000	>50,000	ND
	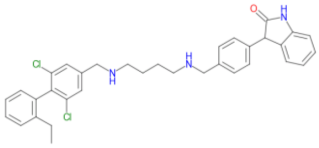	7554	32,990	16,830	ND
	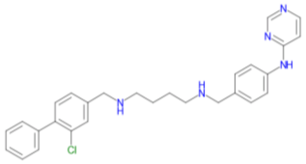	11030	>50,000	10,920	ND
	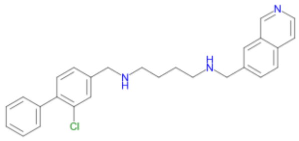	11850	50,000	27,380	ND
	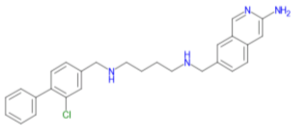	13020	24,990	39,940	ND
	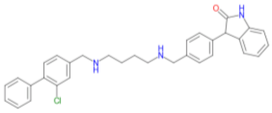	21860	44,360	25,920	ND
	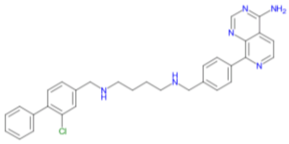	>50000	9,310	32,670	ND
	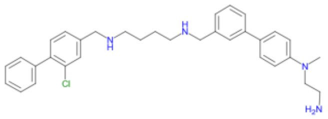	>50000	29,170	47,130	ND
	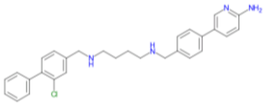	>50,000	>50,000	40,400	>50,000
	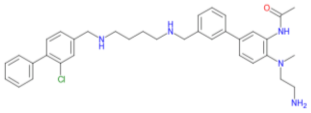	>50,000	>50,000	>50,000	ND

	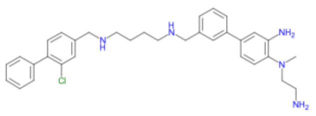	>50,000	>50,000	>50,000	ND

X-ray Crystallography

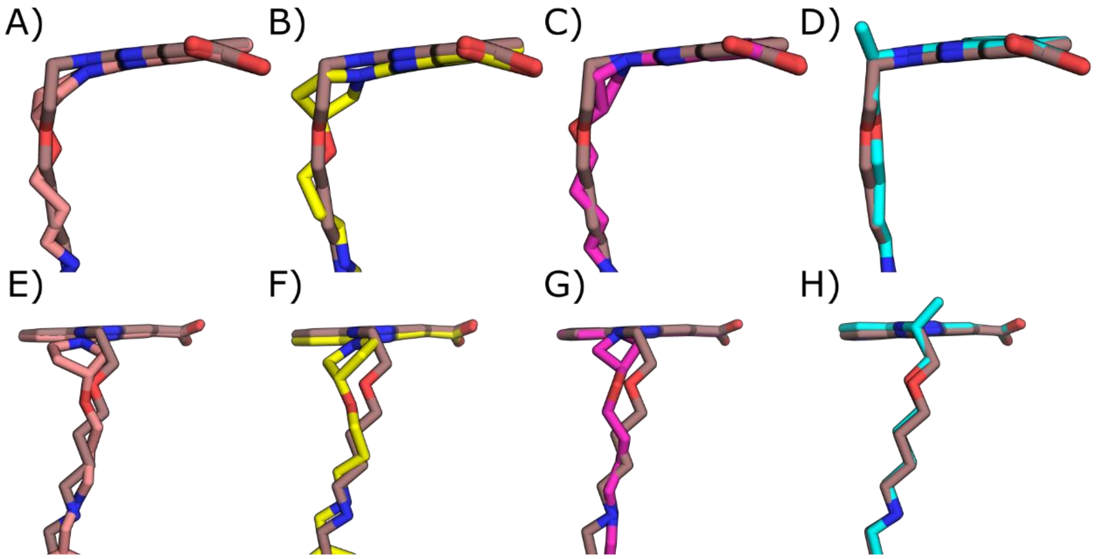

Figure S2. Crystal structures showing compounds with rigidification of the linker. The binding mode of **55b** (brown, PDB: 7I8C) superimposed with the binding modes of: A) and E) **58e** (salmon pink, PDB: 7I8F); B) and F) **58f** (yellow, PDB: 7I8G); C) and G) **58d** (pink, PDB: 7I8E); D) and H) **58b** (blue, PDB: 7I8H).

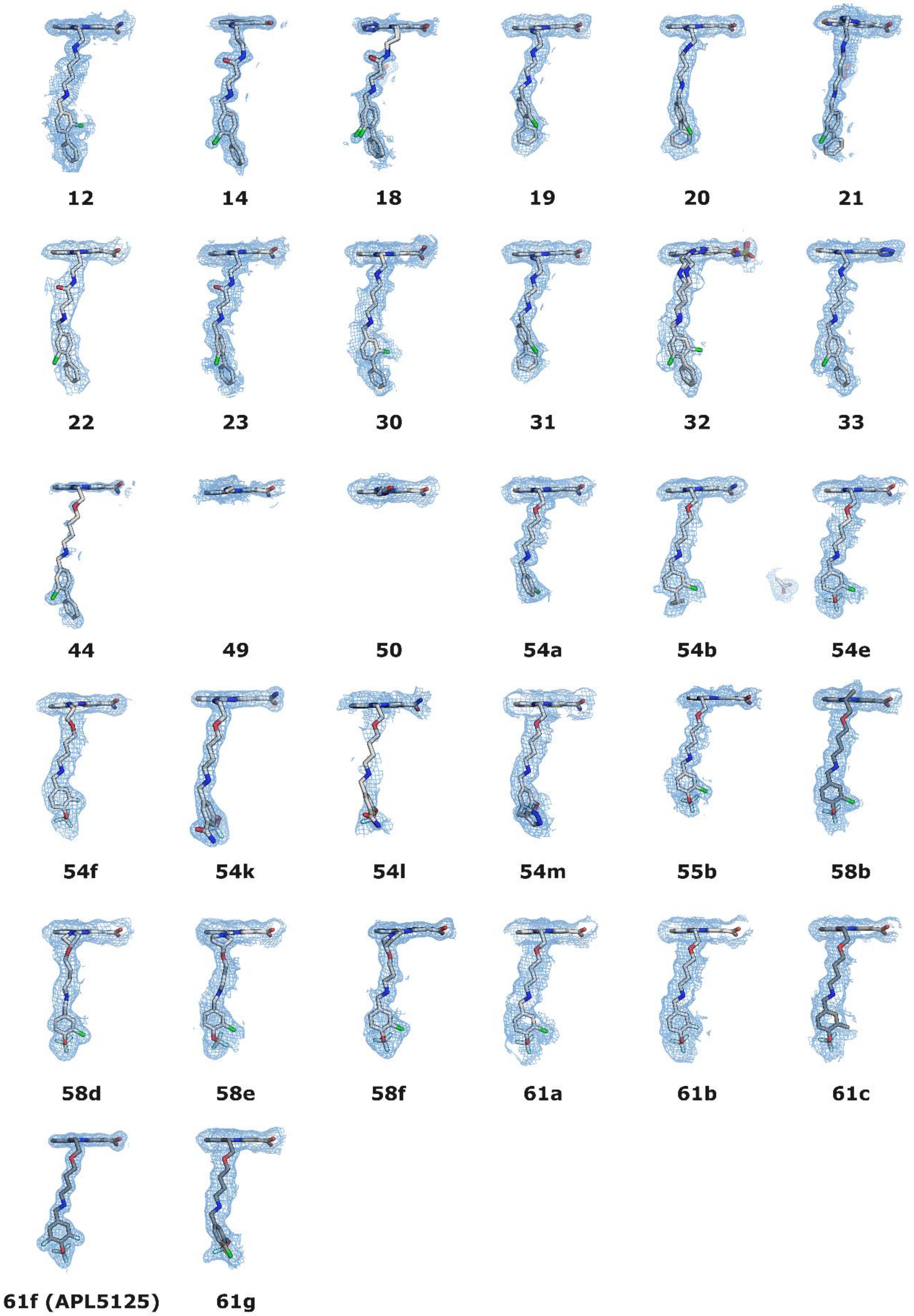

Figure S2. The 2mFo-DFc electron density maps of CK2α inhibitor complexes contoured at 1.0σ before ligand fitting for the crystal structures described.

Table S2. Data collection and refinement statistics.

**Ligand**	**12**	**14**	**18**
PDB code	7I8N	7I8M	7I8O
Data Collection:			
Beamline	DIAMOND BEAMLINE i04-1	DIAMOND BEAMLINE i03	DIAMOND BEAMLINE i04-1
Wavelength (Å)	0.9159	0.9763	0.9159
Resolution range (Å)	54.04 - 1.28 (1.35 - 1.28)	50.23 - 1.31 (1.39 - 1.31)	54.20 - 1.31 (1.38 - 1.31)
Space group	P 1 2_1 1	P 1 2_1 1	P 1 2_1 1
Cell (a b c) (Å)	58.62 46.32 62.66	58.58 46.20 62.72	58.55 46.27 62.80
Cell (α β γ) (°)	90.00 112.82 90.00	90.00 112.24 90.00	90.00 112.41 90.00
Total reflections	331239 (41460)	314591 (44775)	451254 (62903)
Unique reflections	76942 (11451)	73135 (10765)	75448 (10803)
Multiplicity	4.3 (3.6)	4.3 (4.2)	6.0 (5.8)
Completeness (%)	97.1 (99.3)	98.8 (99.9)	99.2 (98.2)
Mean I/sigma(I)	7.7 (0.8)	6.5 (1.0)	11.1 (0.7)
R-merge	0.085 (1.23)	0.117 (1.38)	0.071 (2.40)
R-pim	0.045 (0.74)	0.062 (0.76)	0.031 (1.08)
CC-half	0.996 (0.45)	0.995 (0.37)	0.999 (0.39)
Refinement:			
Resolution range (Å)	54.04 - 1.28 (1.31 - 1.28)	50.23 - 1.31 (1.34 - 1.31)	54.13 - 1.31 (1.34 - 1.31)
No. of reflections	76924 (5161)	73112 (4784)	74058 (5291)
No. of reflections (R_{free})	3876 (237)	3651 (208)	3580 (264)
R-factor	0.225 (0.254)	0.200 (0.213)	0.206 (0.284)
R_{free}	0.246 (0.283)	0.216 (0.237)	0.221 (0.285)
Number of total atoms	2958	3055	3139
No.of atoms for macromolecules	2748	2748	2748
No.of atoms for ligands	46	44	48
No.of atoms for waters	164	263	343
Average B-factor (Å^2)	21.4	21.3	23.5
RMS (bonds)(Å)	0.01	0.01	0.01
RMS (bond angles) (°)	0.99	0.99	0.98
RMS (dihedral angles) (°)	3.75	3.64	3.57

**Ligand**	**19**	**20**	**21**
PDB code	7I80	7I82	7I8P
Data Collection:			
Beamline	DIAMOND BEAMLINE I04	ESRF BEAMLINE ID30B	DIAMOND BEAMLINE i04-1
Wavelength (Å)	0.979	0.9762	0.9159
Resolution range (Å)	54.13 - 1.57 (1.65 - 1.57)	36.33 - 1.51 (1.60 - 1.51)	54.16 - 1.25 (1.31 - 1.25)
Space group	P 1 2_1 1	P 1 2_1 1	P 1 2_1 1
Cell (a b c) (Å)	58.24 45.97 62.90	58.61 46.24 63.32	58.67 46.16 63.19
Cell (α β γ)(°)	90.00 111.66 90.00	90.00 111.93 90.00	90.00 112.61 90.00
Total reflections	236847 (34456)	116149 (6200)	275440 (31310)
Unique reflections	43509 (6334)	34106 (1706)	85553 (12055)
Multiplicity	5.4 (5.4)	3.4 (3.6)	3.2 (2.6)
Completeness (%)	99.9 (100.0)	69.4 (24.5)	98.2 (95.6)
Mean I/sigma(I)	13.8 (0.9)	11.1 (1.4)	8.2 (0.7)
R-merge	0.054 (1.54)	0.054 (0.79)	0.061 (1.24)
R-pim	0.025 (0.72)	0.034 (0.47)	0.040 (0.94)
CC-half	0.999 (0.44)	0.998 (0.588)	0.998 (0.43)
Refinement:			
Resolution range (Å)	22.99 - 1.57 (1.61 - 1.57)	36.33 - 1.73 (1.79 - 1.73)	25.00 - 1.25 (1.28 - 1.25)
No. of reflections	43134 (3189)	27966 (3405)	84814 (5982)
No. of reflections (R_{free})	2176 (149)	1438 (183)	4293 (308)
R-factor	0.191 (0.202)	0.184 (0.246)	0.213 (0.239)
R_{free}	0.222 (0.200)	0.221 (0.312)	0.236 (0.254)
Number of total atoms	3052	2971	2992
No.of atoms for macromolecules	2748	2748	2748
No.of atoms for ligands	50	47	57
No.of atoms for waters	254	176	187
Average B-factor (Å^2)	34.4	34.1	23.4
RMS (bonds) (Å)	0.01	0.01	0.01
RMS (bond angles) (°)	0.97	0.96	0.99
RMS (dihedral angles) (°)	3.29	3.05	3.64

**Ligand**	**22**	**23**	**30**
PDB code	7I81	7I86	7I7Y
Data Collection:			
Beamline	ESRF BEAMLINE ID30B	DIAMOND BEAMLINE I03	ESRF BEAMLINE ID30B
Wavelength (Å)	0.9762	0.9762	0.9762
Resolution range (Å)	36.25 - 1.92 (2.05- 1.92)	54.29 - 1.32 (1.39 - 1.32)	58.91 - 1.16 (1.23 - 1.16)
Space group	P 1 2_1 1	P 1 2_1 1	P 1 2_1 1
Cell (a b c) (Å)	58.25 46.28 62.82	58.35 45.85 63.06	58.68 46.17 63.35
Cell (α β γ)(°)	90.00 111.89 90.00	90.00 111.50 90.00	90.00 111.60 90.00
Total reflections	62387 (3603)	234893 (18915)	285301 (16708)
Unique reflections	16849 (842)	67267 (5803)	82305 (4115)
Multiplicity	3.7 (4.3)	3.5 (3.3)	3.5 (4.1)
Completeness (%)	70.3 (20.4)	91.6 (54.4)	75.2 (21.9)
Mean I/sigma(I)	6.6 (1.3)	9.8 (1.6)	6.5 (1.7)
R-merge	0.186 (2.90)	0.063 (0.90)	0.103 (1.49)
R-pim	0.109 (1.53)	0.038 (0.56)	0.063 (0.83)
CC-half	0.987 (0.202)	0.996 (0.39)	0.989 (0.170)
Refinement:			
Resolution range (Å)	36.25 - 2.33 (2.52 - 2.33)	54.29 - 1.32 (1.35 - 1.32)	58.91 - 1.15 (1.18 - 1.15)
No. of reflections	13182 (2672)	66812 (5079)	82093 (586)
No. of reflections (R_{free})	662 (139)	3379 (284)	4155 (-)
R-factor	0.215 (0.271)	0.188 (0.239)	0.199 (0.227)
R_{free}	0.304 (0.387)	0.217 (0.251)	0.218 (0.224)
Number of total atoms	2856	2997	3057
No.of atoms for macromolecules	2748	2748	2748
No.of atoms for ligands	41	45	44
No.of atoms for waters	67	204	265
Average B-factor (Å^2)	40	28.4	22.5
RMS (bonds) (Å)	0.01	0.01	0.01
RMS (bond angles) (°)	1.11	1.02	1.02
RMS (dihedral angles) (°)	2.91	3.45	3.65

**Ligand**	**31**	**32**	**33**
PDB code	7I7Z	7I84	7I83
Data Collection:			
Beamline	ESRF BEAMLINE ID30B	DIAMOND BEAMLINE I03	DIAMOND BEAMLINE i04
Wavelength (Å)	0.9762	0.9763	0.9795
Resolution range (Å)	59.02 - 1.35 (1.44 - 1.35)	96.59 - 2.32 (2.44 - 2.32)	54.36 - 1.51 (1.59 - 1.51)
Space group	$P\ 1\ 2_1\ 1$	$P\ 1\ 2_1\ 1$	$P\ 1\ 2_1\ 1$
Cell (a b c) (Å)	58.31 46.18 63.44	58.48 46.43 63.25	58.60 46.39 62.95
Cell (α β γ)(°)	90.00 111.50 90.00	90.00 112.03 90.00	90.00 111.93 90.00
Total reflections	288837 (17257)	79552 (5481)	461624 (56577)
Unique reflections	51807 (2591)	21067 (2416)	48869 (6836)
Multiplicity	5.6 (6.7)	3.8 (2.3)	9.4 (8.3)
Completeness (%)	75.7 (23.0)	92.0 (72.6)	98.2 (94.7)
Mean I/sigma(I)	9.1 (1.4)	2.6 (0.4)	16.2 (1.1)
R-merge	0.097 (1.62)	0.576 (2.49)	0.082 (1.65)
R-pim	0.044 (0.68)	0.338 (1.78)	0.028 (0.60)
CC-half	0.994 (0.269)	0.880 (0.50)	0.999 (0.48)
Refinement:			
Resolution range (Å)	54.26 - 1.35 (1.39 - 1.35)	36.40 - 1.97 (2.07 - 1.97)	22.11 - 1.51 (1.55 - 1.51)
No. of reflections	51804 (310)	21230 (2879)	48462 (3405)
No. of reflections (R_{free})	2612 (-)	1076 (124)	2436 (173)
R-factor	0.213 (0.222)	0.186 (0.220)	0.193 (0.216)
R_{free}	0.236 (0.256)	0.237 (0.258)	0.223 (0.239)
Number of total atoms	2959	2975	3079
No.of atoms for macromolecules	2748	2748	2748
No.of atoms for ligands	41	48	50
No.of atoms for waters	170	179	281
Average B-factor (Å^2)	25.6	33.2	27.8
RMS (bonds) (Å)	0.01	0.01	0.01
RMS (bond angles) (°)	1	1.02	0.93
RMS (dihedral angles) (°)	3.45	3.16	3.28

**Ligand**	**44**	**49**	**50**
PDB code	7I87	7I85	7I88
Data Collection:			
Beamline	DIAMOND BEAMLINE I24	DIAMOND BEAMLINE I03	DIAMOND BEAMLINE I24
Wavelength (Å)	0.976	0.9762	0.9686
Resolution range (Å)	58.57 - 1.47 (1.54 - 1.47)	59.48 - 1.44 (1.52 - 1.44)	54.57 - 1.96 (2.06 - 1.96)
Space group	$P\ 1\ 2_1\ 1$	$P\ 1\ 2_1\ 1$	$P\ 1\ 2_1\ 1$
Cell (a b c) (Å)	58.31 46.28 63.03	58.54 46.06 64.01	58.65 45.98 63.54
Cell (α β γ) (°)	90.00 111.69 90.00	90.00 111.69 90.00	90.00 111.50 90.00
Total reflections	398166 (57477)	389644 (57968)	149860 (21768)
Unique reflections	52523 (7494)	55785 (8299)	23027 (3337)
Multiplicity	7.6 (7.7)	7.0 (7.0)	6.5 (6.5)
Completeness (%)	97.4 (95.4)	97.2 (99.7)	99.9 (100.0)
Mean I/sigma(I)	9.4 (1.1)	7.7 (0.6)	7.6 (2.4)
R-merge	0.105 (2.15)	0.149 (6.43)	0.170 (1.18)
R-pim	0.041 (0.82)	0.061 (2.68)	0.073 (0.51)
CC-half	0.998 (0.42)	0.996 (0.42)	0.991 (0.56)
Refinement:			
Resolution range (Å)	21.52 - 1.47 (1.51 - 1.47)	59.48 - 1.44 (1.48 - 1.44)	21.67 - 1.96 (2.06 - 1.96)
No. of reflections	51980 (3752)	55518 (3974)	22827 (2996)
No. of reflections (R_{free})	2629 (208)	2808 (202)	1150 (138)
R-factor	0.181 (0.224)	0.195 (0.252)	0.181 (0.213)
R_{free}	0.209 (0.231)	0.222 (0.269)	0.217 (0.264)
Number of total atoms	3005	2997	2917
No.of atoms for macromolecules	2748	2748	2748
No.of atoms for ligands	44	20	23
No.of atoms for waters	213	229	146
Average B-factor (Å^2)	27.2	27.9	36.1
RMS (bonds) (Å)	0.01	0.01	0.01
RMS (bond angles) (°)	0.96	0.97	0.98
RMS (dihedral angles) (°)	3.43	3.09	2.96

**Ligand**	**54a**	**54b**	**54e**
PDB code	7I89	7I8B	9QY7
Data Collection:			
Beamline	DIAMOND BEAMLINE I04-1	DIAMOND BEAMLINE I04-1	DIAMOND BEAMLINE I04-1
Wavelength (Å)	0.9159	0.9159	0.9159
Resolution range (Å)	54.33 - 1.49 (1.57 - 1.49)	58.33 - 1.73 (1.82- 1.73)	49.96 - 1.39 (1.46 - 1.39)
Space group	P 1 2_1 1	P 1 2_1 1	P 1 2_1 1
Cell (a b c) (Å)	58.45 46.17 63.11	57.96 45.80 62.64	58.30 45.81 63.06
Cell (α β γ) (°)	90.00 111.63 90.00	90.00 111.39 90.00	90.00 111.39 90.00
Total reflections	196085 (30596)	144786 (21498)	275869 (39546)
Unique reflections	48167 (7329)	31685 (4544)	62446 (9036)
Multiplicity	4.1 (4.2)	4.6 (4.7)	4.4 (4.4)
Completeness (%)	93.7 (98.4)	97.9 (96.9)	99.8 (99.9)
Mean I/sigma(I)	7.7 (1.2)	10.2 (0.8)	9.6 (0.7)
R-merge	0.095 (1.55)	0.082 (1.71)	0.075 (2.02)
R-pim	0.053 (0.86)	0.043 (0.87)	0.040 (1.07)
CC-half	0.997 (0.41)	0.999 (0.40)	0.999 (0.37)
Refinement:			
Resolution range (Å)	21.82 - 1.49 (1.53 - 1.49)	58.33 - 1.73 (1.79 - 1.73)	49.96 - 1.39 (1.43 - 1.39)
No. of reflections	47529 (3380)	31375 (2775)	61802 (4064)
No. of reflections (R_{free})	2393 (178)	1593 (139)	3098 (196)
R-factor	0.186 (0.223)	0.190 (0.224)	0.182 (0.264)
R_{free}	0.221 (0.254)	0.215 (0.230)	0.208 (0.280)
Number of total atoms	3039	2955	3117
No.of atoms for macromolecules	2748	2748	2748
No.of atoms for ligands	42	41	47
No.of atoms for waters	249	166	322
Average B-factor (Å^2)	26.6	36.9	26.1
RMS (bonds) (Å)	0.01	0.01	0.01
RMS (bond angles) (°)	0.99	0.98	0.99
RMS (dihedral angles) (°)	3.28	3.15	3.39

**Ligand**	**54f**	**54k**	**54l**
PDB code	7I8A	9QQX	7I8Q
Data Collection:			
Beamline	DIAMOND BEAMLINE I04-1	DIAMOND BEAMLINE I04-1	DIAMOND BEAMLINE I03
Wavelength (Å)	0.9159	0.9179	0.9763
Resolution range (Å)	58.35 - 1.84 (1.94 - 1.84)	58.91 - 1.60 (1.68 - 1.60)	59.48 - 1.44 (1.52 - 1.44)
Space group	P 1 2_1 1	P 1 21 1	P 1 2_1 1
Cell (a b c) (Å)	57.36 45.55 62.51	59.11 46.30 63.59	58.13 46.20 62.76
Cell (α β γ) (°)	90.00 111.00 90.00	90.00 112.12 90.00	90.00 111.66 90.00
Total reflections	116735 (17215)	1013851 (158514)	389644 (57968)
Unique reflections	26216 (3786)	38789 (6120)	55785 (8299)
Multiplicity	4.5 (4.5)	26.1 (25.9)	7.0 (7.0)
Completeness (%)	99.9 (99.8)	91.2 (100.0)	97.2 (99.7)
Mean I/sigma(I)	9.3 (0.6)	17.0 (0.4)	7.7 (0.6)
R-merge	0.086 (2.25)	0.099 (7.19)	0.149 (6.43)
R-pim	0.046 (1.19)	0.020 (1.43)	0.061 (2.68)
CC-half	0.999 (0.41)	1.000 (0.49)	0.996 (0.42)
Refinement:			
Resolution range (Å)	21.72 - 1.84 (1.92 - 1.84)	31.32 - 1.60 (1.61 - 1.60)	35.11 - 1.66 (1.71 - 1.66)
No. of reflections	25909 (2551)	38572 (772)	35602 (2297)
No. of reflections (R_{free})	1304 (134)	1949 (-)	1794 (103)
R-factor	0.194 (0.257)	0.221 (0.343)	0.189 (0.260)
R_{free}	0.234 (0.273)	0.245 (0.353)	0.220 (0.272)
Number of total atoms	2874	2980	3033
No.of atoms for macromolecules	2713	2749	2749
No.of atoms for ligands	43	45	75
No.of atoms for waters	118	186	209
Average B-factor (Å^2)	45.2	37.6	31.8
RMS (bonds) (Å)	0.01	0.008	0.01
RMS (bond angles) (°)	0.99	0.86	1
RMS (dihedral angles) (°)	2.94	3.1	3.06

**Ligand**	**54m**	**55b**	**58b**
PDB code	7I8R	7I8C	7I8H
Data Collection:			
Beamline	DIAMOND BEAMLINE I03	DIAMOND BEAMLINE I04-1	DIAMOND BEAMLINE I24
Wavelength (Å)	0.9763	0.9159	0.9999
Resolution range (Å)	54.18 - 1.72 (1.81 - 1.72)	36.26 - 1.57 (1.59 - 1.57)	50.12 - 1.39 (1.46 - 1.39)
Space group	P 1 2_1 1	P 1 2_1 1	P 1 2_1 1
Cell (a b c) (Å)	58.41 46.31 63.08	58.46 46.18 63.19	58.40 45.74 62.73
Cell (α β γ) (°)	90.00 111.95 90.00	90.00 112.04 90.00	90.00 112.15 90.00
Total reflections	226947 (32014)	405779 (20095)	1565974 (209474)
Unique reflections	32549 (4673)	43499 (2086)	60194 (8533)
Multiplicity	7.0 (6.9)	9.3 (9.6)	26.0 (24.5)
Completeness (%)	97.7 (96.5)	98.8 (94.9)	96.7 (94.5)
Mean I/sigma(I)	7.5 (1.1)	10.3 (1.5)	15.6 (2.0)
R-merge	0.166 (1.88)	0.139 (1.53)	0.207 (3.01)
R-pim	0.067 (0.77)	0.049 (0.52)	0.041 (0.61)
CC-half	0.997 (0.38)	0.996 (0.61)	0.999 (0.51)
Refinement:			
Resolution range (Å)	54.18 - 1.72 (1.78 - 1.72)	22.99 - 1.57 (1.61 - 1.57)	50.12 - 1.39 (1.43 - 1.39)
No. of reflections	32502 (2840)	43185 (3195)	59645 (4255)
No. of reflections (R_{free})	1661 (152)	2168 (179)	2993 (195)
R-factor	0.177 (0.228)	0.190 (0.223)	0.187 (0.235)
R_{free}	0.221 (0.275)	0.217 (0.268)	0.209 (0.263)
Number of total atoms	3025	3076	3008
No.of atoms for macromolecules	2749	2748	2748
No.of atoms for ligands	48	43	44
No.of atoms for waters	228	285	216
Average B-factor (Å^2)	28.6	24.8	23.9
RMS (bonds) (Å)	0.01	0.01	0.01
RMS (bond angles) (°)	1.04	0.96	1.06
RMS (dihedral angles) (°)	3.12	3.38	3.59

**Ligand**	**58d**	**58e**	**58f**
PDB code	7I8E	7I8F	7I8G
Data Collection:			
Beamline	DIAMOND BEAMLINE I04	DIAMOND BEAMLINE I04-1	DIAMOND BEAMLINE I04-1
Wavelength (Å)	0.9795	0.9119	0.9119
Resolution range (Å)	50.16 - 1.66 (1.75 - 1.66)	58.01 - 1.45 (1.53 - 1.45)	58.46 - 1.23 (1.29- 1.23)
Space group	$P\ 1\ 2_1\ 1$	$P\ 1\ 2_1\ 1$	$P\ 2_1 2_1 2_1$
Cell (a b c) (Å)	58.57 45.75 62.91	57.91 45.62 62.45	49.39 62.22 116.92
Cell (α β γ)(°)	90.00 111.78 90.00	90.00 111.75 90.00	90.00 90.00 90.00
Total reflections	248853 (35890)	394662 (58076)	1398606 (148276)
Unique reflections	36961 (5340)	53859 (7811)	105891 (15201)
Multiplicity	6.7 (6.7)	7.3 (7.4)	13.2 (9.8)
Completeness (%)	99.8 (100.0)	99.9 (100.0)	99.9 (99.6)
Mean I/sigma(I)	11.0 (0.8)	15.9 (0.8)	11.9 (1.6)
R-merge	0.072 (2.30)	0.049 (2.19)	0.104 (1.29)
R-pim	0.030 (0.95)	0.019 (0.86)	0.029 (0.43)
CC-half	0.999 (0.41)	1.000 (0.43)	0.998 (0.52)
Refinement:			
Resolution range (Å)	50.16 - 1.66 (1.71 - 1.66)	58.01 - 1.45 (1.49 - 1.45)	18.01 - 1.23 (1.26 - 1.23)
No. of reflections	34804 (3068)	53605 (3742)	105036 (7581)
No. of reflections (R_{free})	1752 (137)	2707 (209)	5301 (395)
R-factor	0.208 (0.207)	0.193 (0.232)	0.191 (0.226)
R_{free}	0.244 (0.235)	0.215 (0.271)	0.201 (0.231)
Number of total atoms	2940	2980	3042
No.of atoms for macromolecules	2748	2748	2754
No.of atoms for ligands	44	44	44
No.of atoms for waters	148	188	244
Average B-factor (Å^2)	39.6	33.8	25.2
RMS (bonds) (Å)	0.01	0.01	0.01
RMS (bond angles) (°)	1	1.03	1
RMS (dihedral angles) (°)	3	3.26	3.62

**Ligand**	**61a**	**61b**	**61c**
PDB code	7I8D	7I8I	7I8L
Data Collection:			
Beamline	DIAMOND BEAMLINE I04	DIAMOND BEAMLINE I03	DIAMOND BEAMLINE I24
Wavelength (Å)	0.9795	0.9763	0.9999
Resolution range (Å)	58.31 - 1.38 (1.46 - 1.38)	58.51 - 1.47 (1.54 - 1.47)	58.70 - 1.44 (1.51 - 1.44)
Space group	P 1 2_1 1	P 1 2_1 1	P 1 2_1 1
Cell (a b c) (Å)	58.22 46.03 62.83	58.45 45.94 63.23	58.49 46.24 63.30
Cell (α β γ) (°)	90.00 111.87 90.00	90.00 112.29 90.00	90.00 112.13 90.00
Total reflections	430485 (62431)	367205 (53133)	1440077 (212429)
Unique reflections	63102 (9185)	51614 (7340)	57622 (8349)
Multiplicity	6.8 (6.8)	7.1 (7.2)	25.0 (25.4)
Completeness (%)	100.0 (100.0)	96.7 (94.5)	100.0 (100.0)
Mean I/sigma(I)	12.6 (0.9)	14.0 (1.0)	11.9 (1.1)
R-merge	0.055 (1.89)	0.065 (1.83)	0.242 (5.24)
R-pim	0.023 (0.78)	0.026 (0.73)	0.049 (1.05)
CC-half	0.999 (0.48)	0.999 (0.50)	0.999 (0.42)
Refinement:			
Resolution range (Å)	27.60 - 1.38 (1.42 - 1.38)	21.52 - 1.47 (1.51 - 1.47)	54.18 - 1.48 (1.52 - 1.48)
No. of reflections	62941 (3996)	51119 (3639)	52417 (3781)
No. of reflections (R_{free})	3160 (182)	2580 (183)	2644 (199)
R-factor	0.193 (0.242)	0.180 (0.234)	0.183 (0.218)
R_{free}	0.216 (0.272)	0.208 (0.276)	0.203 (0.248)
Number of total atoms	2970	3049	3003
No.of atoms for macromolecules	2748	2749	2748
No.of atoms for ligands	47	70	70
No.of atoms for waters	175	230	185
Average B-factor (Å^2)	29.7	29.8	27.4
RMS (bonds) (Å)	0.01	0.01	0.01
RMS (bond angles) (°)	1.01	1	1.02
RMS (dihedral angles) (°)	3.55	3.3	3.44

**Ligand**	**61f (APL-5125)**	**61g**
PDB code	7I8K	7I8J
Data Collection:		
Beamline	DIAMOND BEAMLINE I04	DIAMOND BEAMLINE I24
Wavelength (Å)	0.9795	0.9999
Resolution range (Å)	54.36 - 1.38 (1.45 - 1.38)	58.70 - 1.44 (1.51 - 1.44)
Space group	$P\ 1\ 2_1\ 1$	$P\ 1\ 2_1\ 1$
Cell (a b c) (Å)	58.66 45.91 63.26	58.54 46.28 63.30
Cell (α β γ) (°)	90.00 112.07 90.00	90.00 112.27 90.00
Total reflections	1474046 (216371)	1440077 (212429)
Unique reflections	64679 (9349)	57622 (8349)
Multiplicity	22.8 (23.1)	25.0 (25.4)
Completeness (%)	99.9 (99.6)	100.0 (100.0)
Mean I/sigma(I)	25.0 (0.7)	11.9 (1.1)
R-merge	0.052 (4.48)	0.242 (5.24)
R-pim	0.011 (0.94)	0.049 (1.05)
CC-half	1.000 (0.46)	0.999 (0.42)
Refinement:		
Resolution range (Å)	27.32 - 1.38 (1.39 - 1.38)	54.17 - 1.60 (1.64 - 1.60)
No. of reflections	64395 (1288)	41442 (2917)
No. of reflections (R_{free})	3248 (-)	2098 (153)
R-factor	0.208 (0.338)	0.191 (0.235)
R_{free}	0.230 (0.293)	0.229 (0.272)
Number of total atoms	3022	2960
No.of atoms for macromolecules	2749	2749
No.of atoms for ligands	44	39
No.of atoms for waters	229	172
Average B-factor (Å^2)	32.3	36.7
RMS (bonds) (Å)	0.008	0.01
RMS (bond angles) (°)	0.92	1.01
RMS (dihedral angles) (°)	3.43	3.15

Surface Plasmon Resonance

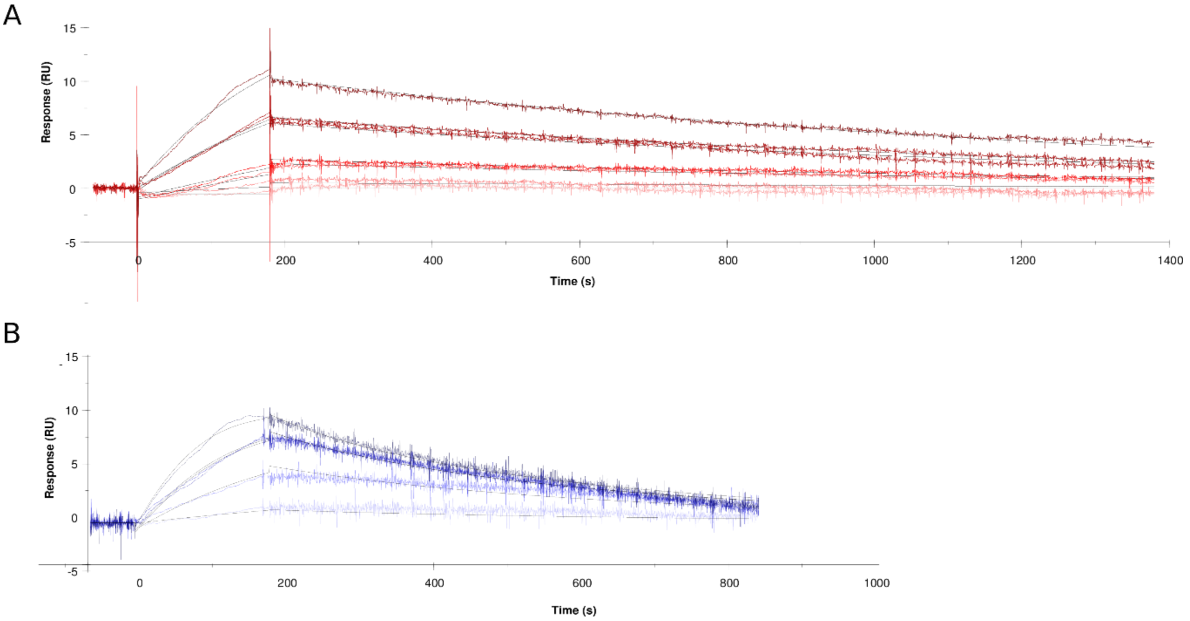

Figure S4. Kinetic binding data from surface plasmon resonance for compounds interacting with CK2α. A) **61f** (red). B) Silmitasertib (**1**) (blue).

In vitro ADME and hERG

Thermodynamic Solubility Measurement

Thermodynamic solubility was determined using the shake-flask method followed by HPLC-UV analysis. An appropriate quantity of test compound was weighed into the lower chamber of Whatman Mini-UniPrep™ vials. 450 µL of 50 mM phosphate buffer (PB, pH 1.0, 3.5, 4.5, 6.5, 7.4, 9.0 and 10.0), Fasted State Simulated Intestinal Fluid (FaSSIF) or Fed State Simulated Intestinal Fluid (FeSSIF) was dispensed into the lower chamber of the Mini-UniPrep™ vials and samples vortexed for 2 min. Samples were incubated at room temperature with shaking at 800 rpm for 24 h then centrifuged at 4000 rpm for 20 min. The Whatman Mini-UniPrep™ vials were compressed to prepare filtrates for injection into a UPLC system and the concentration of each compound determined based on prepared calibration curves using a UV-VIS spectrophotometry method.

pK_a Determination

Final pKa was obtained by pH metric and UV metric at pH 2–12. Sirius T3Dt (Sirius Analytical Instruments Ltd.) fitted with combination Ag/AgCl pH electrode was used for the determination of dissociation constants. The pK_a and psK_a values were calculated by Sirius

T3Dt software (Sirius Analytical Instruments Ltd, Version 1.0.12.120) using the two methods available: potentiometric titration and spectroscopic (UV) titration.

pH metric method: a 1 mg sample of test article was weighed into a sample vial and 1.5 mL 80% v/v MeOH was added. The sample solution was pre-acidified with 0.5 M HCl, then titrated three times from high to low pH. The titrations were carried out at constant ionic strength (I = 0.15 M KCl) and temperature (25.0 ± 0.5 ºC). The aqueous pK_a value of sample was extrapolated from three different psK_a values in the presence of varying amount of cosolvent by Sirius T3Dt software. pK_a values were determined from the shape of the resultant titration curves and fitting a suitable theoretical model for the compound's ionization behaviour onto the titration data.

Ultraviolet (UV) metric method: a 5 µL sample of 10 mmol/L test compound in DMSO and 25 µL fast UV buffer were pipetted into a sample vial. 1.5 mL of 80% v/v MeOH (5.59 g KCl in 100 mL deionized water and made up to 500 mL with analytical grade methanol) was added into the sample vial. The sample solution was pre-acidified with 0.5 M hydrochloric acid (HCl), then titrated three times from low to high pH. pK_a values were determined by monitoring the change in UV absorbance with pH as the compound underwent ionization. This information produced a 3D matrix of pH vs. wavelength vs. absorbance data. Target Factor Analysis was applied to the matrix to produce molar absorption profiles for the different light absorbing species present in solution, and also a Distribution of Species plot showing the proportion of each species with pH.

When co-solvent solutions were employed by pH-metric method or UV-metric method, the psK_a values are extrapolated to 0% organic content using the Yasuda-Shedlovsky extrapolation procedure, which yielded extrapolated aqueous pK_a values and slope information which was used to ascertain the acidic/basic characteristics of the ionisable groups.

Hepatocyte Metabolite Identification

The test compound (10 µM) was incubated with hepatocytes at 37 ºC for 120 min. The positive control 7-ethoxycoumarin (7-EC) was run concurrently to assess Phase I and Phase II metabolic activities in hepatocytes. After incubation, the samples were analyzed by LC-MS. The structures of the metabolites were proposed based on the interpretation of their MS and MS^2 data.

Permeability and Transporter Substrate Studies

A BSEP vesicle assay was used to assess whether test compounds (1, 10 and 100 μM) or cyclosporine (positive control, 50 μM) acted as BSEP substrate. The final assay conditions were 2 μM taurocholic acid, 0.05 mg/mL human BSEP vesicles and 5 mM ATP/AMP in uptake buffer (10 mM HEPES-Tris, 100 mM KNO_3, 12.5 mM $Mg(NO_3)_2$, 50 mM Sucrose, pH 7.4) and 5 min incubation at 37 °C. Reactions were stopped and transferred onto blocked (0.1% BSA) microplate glass fiber filters presoaked with cold stop buffer. The filters were washed and elution buffer containing the appropriate analytical IS added before centrifugation with the collection microplate. Test compound concentrations were determined by LC-MS/MS and expressed as peak area ratio of analyte to IS.

The following formula were used to determine transporter substrate values:

Peak Area Ratio (PAR) = Analyte Peak Area / IS Peak Area × dilution factor

Uptake activity with ATP or AMP (μL/mg/min) = PAR / (time × amount of protein)

Uptake Fold = Uptake activity with ATP (μL/mg/min) / Uptake activity with AMP (μL/mg/min)

Transport activity ratio (R_s/R_i) = Uptake activity without inhibitor ATP (L/mg/min) / Uptake activity with inhibitor ATP (μL/mg/min)

Human embryonic kidney (HEK)293 cells expressing MATE1, OAT1, OAT3, OATP1B1, OATP1B3, OCT1 and OCT2 were used to assess whether test compounds (1, 10 and 100 μM) acted as substrates for these transporters. Cells were seeded into 96well plates at 5×10^4 cells/well and incubated overnight at 37 °C, 5% CO_2. Cells were washed with transport buffer (HBSS containing 25 mM Tricin, pH 8 for MATE1/MATE2K or 10 mM HEPES, pH 7.4 for OAT1/OAT3/OATP1B1/OATP1B3/OCT1/OCT2) and incubated with 20 μM substrate (Metformin for MATE1/MATE2K/OCT2, p-Aminohippurate for OAT1, Estrone 3-sulfate for OAT3/OATP1B1 and β-Estradiol 17-(β-D-glucuronide) for OATP1B3, 1-methyl-4-phenylpyridinium for OCT1) and test compound (50 μM final test concentration) at 37 °C, 5% CO_2 (0.5 min for OATP1B1/OCT2, 2 min for OAT1/OAT3, 4 min for MATE1, 5 min for OATP1B3/OCT1, 15 min for MATE2K). Cells were rinsed three times with cold transporter buffer and lysed with 100 μL cold acetonitrile/methanol (95:5 v:v) containing internal standard and shaken gently for 30 min. 75 μL of cell lysate was pipetted to mix with 75 μL transport buffer and acetonitrile/methanol (95:5 v:v) containing internal standard as a cell sample for

intracellular uptake determination. Samples were centrifuged at 3220 × g for 10 min and supernatant was taken from each well for LC-MS/MS analysis.

The following formula were used to determine transporter substrate values:

Peak Area Ratio (PAR) = Analyte Peak Area / IS Peak Area × dilution factor

Transport activity (μL/mg/min) = PAR / (time × amount of protein)

Uptake Fold = Transport activity in HEK293 transporter cells / Transport activity in HEK293 parent cells

Transport activity ratio (R_s/R_i) = Transport activity without inhibitor / Transport activity with inhibitor

Plasma Protein Binding Assay

The binding of test compound to mouse, rat, dog and human plasma proteins was determined using a Rapid Equilibrium Dialysis (RED) plate technique. Briefly, compound solutions were added to control mouse, rat, dog or human plasma (diluted 1:10 in PBS) to produce final test concentrations of 1, 10, 30 and 100 μM. Buffer chambers were loaded with the relevant volumes of dialysis buffer and spiked sample matrix into the sample chambers of RED insert in triplicate. The unit was sealed and incubated at 37 ºC for 4 h. After incubation 50 μL of plasma or buffer was removed from each well and diluted 1:1 with either buffer (for plasma sample) or control plasma (for buffer sample). The diluted samples were precipitated and the resulting extract analysed by LC-MS/MS.

Cytochrome P450 and Uridine 5'diphospho-glucuronosyltransferase Phenotyping

100 μL human recombinant CYP or UGT (Corning) were incubated with 2 μL test compound for 10 min at 37 °C. The reaction was initiated by transferring 98 μL of cofactor working solutions (3 mM $MgCl_2$, 1.3 mM nicotinamide adenine dinucleotide phosphate, 5 mM glucose 6-phosphate and 1.2 units/mL glucose 6-phosphate dehydrogenase) to the respective well of incubation plates. After 0, 5, 10, 20, 40 and 60 min, reactions were stopped by adding 600 μL quenching/stop solution (100 ng/mL tolbutamide and 100 ng/mL labetalol in acetonitrile) to the respective well of incubation plates. The plates were shaken at 1000 rpm for 10 min and centrifuged at 3220 × g (CYP) or 4000 rpm (UGT) for 20 min. 100 μL supernatant was transferred into 100 μL ultrapure water and the plates shaken for 10 min prior to analysis by LCMS/MS. For data analysis, the relative metabolite formation rate (V) was calculated from

the slope of metabolite formation versus reaction time. Percent contribution from each individual enzyme was calculated by V_x / Total V (Total V is the sum of V_x of each individual expressed CYP isozyme). For CYP enzymes, the percent contribution by each individual CYP in human liver microsomes was normalized with respect to the specific content of the corresponding CYP in native human liver microsomes as listed below.

CYP isoform	1A2	2B6	2C8	2C9	2C19	2D6	3A4
pmol CYP/mg	45	39	64	96	19	10	108

hERG Patch-clamp Assay

HEK293 cells stably expressing the hERG channel were seeded into 35 mm culture dishes at a density allowing single cells to be recorded. All experiments were performed at near physiological temperature (35–37 °C) with each concentration of test compound being tested n = 3 on different cells. For the assay, the culture dishes were continuously perfused (1 mL/min) with the bath solution (137 mM NaCl, 4 mM KCl, 1.8 mM $CaCl_2$, 1 mM $MgCl_2$, 10 mM HEPES, 10 mM D-Glucose, at pH 7.4). After formation of a Gigaohm seal between the patch electrodes and individual hERG stably-transfected HEK293 cells (pipette resistance range: 2.0 MΩ to 7.0 MΩ; seal resistance range: > 1 GΩ) the cell membrane across the pipette tip was ruptured to assure electrical access to the cell interior (whole-cell patch-configuration). hERG outward tail currents were measured upon depolarization of the cell membrane to +20 mV for 2 sec (activation of channels) from a holding potential of -80 mV and upon subsequent repolarization to -40 mV for 3 sec. This voltage protocol was run 10 times at intervals of 10 sec. Once control recordings had been accomplished (less than 5% change of current amplitude within 100 sec), cells were continuously perfused with test compound in bath solution and final concentration of 2% DMSO. During wash-in, the voltage protocol was run continuously at 10 sec intervals until the steady-state level of current block was reached. A concentration–response curve was generated and IC_{50} values calculated using a SigmaPlot 11.0.
